# Selective Dealkenylative Functionalization of Styrenes via C-C Bond Cleavage

**DOI:** 10.34133/2020/7947029

**Published:** 2020-11-10

**Authors:** Jianzhong Liu, Jun Pan, Xiao Luo, Xu Qiu, Cheng Zhang, Ning Jiao

**Affiliations:** ^1^State Key Laboratory of Natural and Biomimetic Drugs, Peking University, 100191 Beijing, China; ^2^State Key Laboratory of Organometallic Chemistry, Chinese Academy of Sciences, Shanghai 200032, China

## Abstract

As a readily available feedstock, styrene with about 25 million tons of global annual production serves as an important building block and organic synthon for the synthesis of fine chemicals, polystyrene plastics, and elastomers. Thus, in the past decades, many direct transformations of this costless styrene feedstock were disclosed for the preparation of high-value chemicals, which to date, generally performed on the functionalization of styrenes through the allylic C-H bond, C(*sp^2^*)-H bond, or the C=C double bond cleavage. However, the dealkenylative functionalization of styrenes via the direct C-C single bond cleavage is so far challenging and still unknown. Herein, we report the novel and efficient C-C amination and hydroxylation reactions of styrenes for the synthesis of valuable aryl amines and phenols via the site-selective C(Ar)-C(alkenyl) single bond cleavage. This chemistry unlocks the new transformation and application of the styrene feedstock and provides an efficient protocol for the late-stage modification of substituted styrenes with the site-directed dealkenylative amination and hydroxylation.

## 1. Introduction

**Figure 1:**
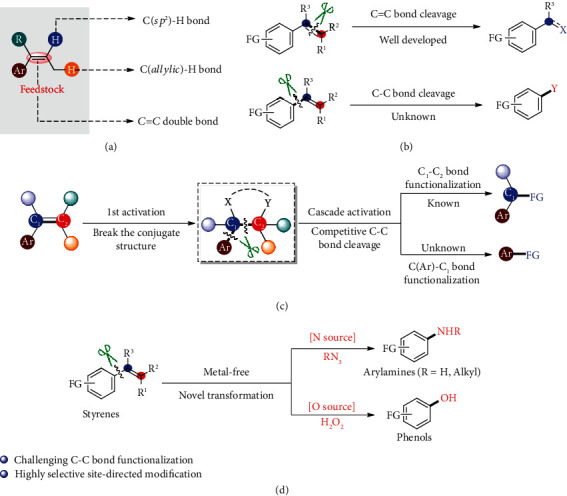
Functionalization based on styrenes. (a) Typical representative transformation patterns of styrene derivatives: C(*sp^2^*)-H activation (for example, Heck-type reaction), C(allylic)-H activation, and C=C double bond activation (for example, wacker oxidation, difunctionalization, polymerization, and metathesis). However, other activation mode is still undeveloped yet very desired to synthetic chemistry. (b) Long-standing unmet challenges in the field of carbon-carbon bond cleavage chemistry of styrenes: although the C=C bond cleavage has been well studied, the dealkenylative C(Ar)-C(alkenyl) single bond cleavage is still unknown. (c) The proposed cascade activation strategy whereby the initial C=C double bond preactivation and the consecutive C(Ar)-C_1_ single bond cleavage sequence may provide a chance to address the above unsolved dealkenylative transformation. However, the C_1_-C_2_ single bond cleavage in conventional styrene chemistry would be a challenging competitive path of the desired process. (d) This work: dealkenylative C-C bond amination and hydroxylation. FG: functional group.

Styrenes are readily available bulk chemicals [1, 2] (produced globally ~25 million tons per year) and widely used in synthesis as a very common building blocks [3, 4]. In the past decades, the development of new direct transformations of styrenes has always been an attractive topic, because it represents the potential industrial application due to the readily available and costless properties of the styrene feedstock. Thus, some classical reactions including the traditional wacker oxidation [5, 6], alkene difunctionalization [7–14], oligomerization or polymerization [15], intramolecular cyclization [16, 17], oxidative cleavage of alkene [18–20], and Heck-type reactions [21, 22], as well as olefin metathesis [23–25], have been well developed and widely applied in chemical synthesis. Generally, these disclosed protocols rely on the functionalization of the C=C double bond [26], the C(*sp^2^*)-H bond [27–30], and the allylic C(*sp^3^*)-H bond [31–35] (Figure 1(a)). Although dealkenylative hydrogenation and thiylation of C(*sp^3^*)–C(*sp^2^*) bonds were significantly developed by Kwon and coworkers [36, 37], the dealkenylative C-C single bond functionalization of styrene is still unknown and remains an unmet challenging issue due to its high thermodynamic stability (the BDE of the C(Ar)-C(alkenyl) single bond is 116.9 kcal/mol [38]) (Figure 1(b)). Thereby, the exploration of a new type of C-C bond activation [39–46] mode and strategy of styrene is undoubtedly very attractive, which may provide an alternative advance in the chemical synthesis and open new avenues for future research of alkene chemistry.

To address the above unsolved dealkenylative C-C single bond functionalization, we proposed a cascade activation strategy via the initial C=C double bond preactivation to break the conjugate structure of styrene and generate the active intermediate for the subsequent C(Ar)-C_1_ bond cleavage. However, the intrinsic C_1_-C_2_ bond cleavage reactivity in styrene chemistry would be a challenging competitive pathway [18–20] (Figure 1(c)). The key point of this strategy is to generate an intermediate with entropic or enthalpic driving force to promote the selective dealkenylative C-C bond cleavage. Herein, we unlock a novel and efficient C-C nitrogenation or hydroxylation reaction of styrenes for the preparation of high-value arylamines and phenols (Figure 1(d)). The significance of this chemistry is trifold: (1) this chemistry provides a new approach to arylamines or phenols under metal-free and simple operation conditions, which are of considerable interest as synthons for the preparation of fine chemicals, pharmaceuticals, agrochemicals, and polymers [47, 48]; (2) compared to the aromatic C-H functionalization approach for the synthesis of arylamines and phenols which suffers from limited substrate scope, harsh conditions, and poor regioselectivity [49–53], the C(Ar)-C(alkenyl) single bond cleavage of styrene contributes a novel site-specific pathway for substituted arylamines and phenols synthesis; and (3) to the best of our knowledge, this chemistry is the first transformation of styrenes via the dealkenylative C-C single bond cleavage, which may inspire further methodology development based on olefins.

## 2. Results

**Figure 2:**
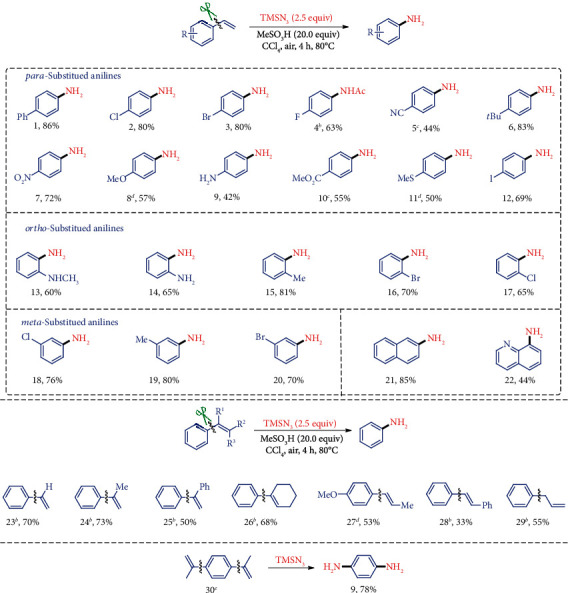
Substrate scope for the aniline synthesis from styrenes. ^a^Standard conditions: reactions were performed with styrene (0.3 mmol), TMSN_3_ (0.75 mmol), and MeSO_3_H (6.0 mmol) in CCl_4_ (1.0 mL) at 80°C for 4 h under atmosphere and isolated yields. ^b^The crude product was acetylated by acetyl chloride. ^c^The reaction was conducted at 40°C instead. ^d^MeSO_3_H (1.5 mmol) was used as the acid. ^e^TMSN_3_ (1.5 mmol) was used instead. CCl_4_: tetrachloromethane.

Although the traditional C=C double bond cleavage leading to the corresponding aldehyde or ketone derivatives [18] provides a great challenge for the desired dealkenylative carbon-carbon functionalization, we investigated the hypothesis by a nucleophilic addition process to initially break the conjugate structure of the substrates. When 4-vinyl-1,1′-biphenyl (1a) was treated with azido nucleophile in the solvents such as DCE and CH_3_CN, unfortunately, the substrate consumed but we did not detect any obvious products except some polymers (see Supplementary Table S1). To our delight, the aniline product 2a was obtained in the solvent of *n*-Hexane or CCl_4_ under acidic conditions (see Supplementary Table S1), which indicated that the two-phase reaction condition generated by the combination of polar acid and the nonpolar solvent was vital to this process. Under the polar acidic conditions, the polymerization of the styrene is a very challenging inherent side reaction, so the choice of the nonpolar solvent such as CCl_4_ is of importance for this dealkenylative transformation due to the formation of the two-phase reaction system with the polar acid to attenuate the side reaction. After the further screening of the acid additives, nitrogenation reagents, and other parameters (see Supplementary Table S3-4), this C-C amination reaction with the conditions of MeSO_3_H (6.0 mmol) and TMSN_3_ (0.75 mmol) in CCl_4_ (1.0 mL) afforded the desired aniline 2a in 86% yield (Figure 2). The subsequent control experiment demonstrates that this chemistry is redox neutral with the acid additive as an essential player.

With the developed optimal reaction conditions, we next investigated the scope of this C-C amination with a series of commercially available or readily prepared styrenes as substrates (Figure 2). As expected, various *para*-substituted styrenes derivatives were compatible with this reaction system, and the corresponding anilines with different electronic properties could be efficiently synthesized. For example, the styrenes bearing electron-donating groups (6, 8, 9, 11, R=OMe, *t*Bu, MeS, NH_2_) underwent the amination process successfully to produce the *para*-substituted anilines in high efficiencies. Substrates containing halogen substituent (2, 3, 12) also performed well to give the corresponding products in good yields, leaving halogens available for the subsequent synthetic transformations. It is noteworthy that substrates with a strong electron-withdrawing group (4, 5, 7, 10, R=F, CN, NO_2_, CO_2_Me) could also deliver the corresponding anilines efficiently using this newly developed method, which is difficult to be prepared through the traditional nitration/reduction sequence or C-H amination pathway. The unprotected amino group is tolerant under these conditions and provides a novel pathway for the synthesis of aryl diamines (9) in moderate yields. The sulfide group which is relatively sensitive to oxidative condition or harsh nitration conditions was not destroyed in this protocol and afforded 4-(methylthio)aniline 11 in 50% yield.

Compared with the traditional nitration/reduction procedure in which the regioselectivity control is very challenging with the substituted arene substrates, the *ortho*-substituted anilines could be synthesized efficiently and selectively using the alkenyl group as a traceless site-directed group, thus without the extra complex isolation of the mixed *ortho-* and *para*-products (13–17). Notably, previously inaccessible *meta*-substituted anilines by the nitration/reduction process could also be prepared in good yields through the present C-C amination process (18–20). In addition, naphthyl and quinoline heterocyclic rings were also compatible, providing the expected product 21 and 22 in 85% and 44% yields, respectively.

To explore the effect of the alkenyl group on the styrenes, 1,1-disubstituted styrenes (24, 25 Figure 2) were first surveyed which produced the aniline products in good yields under the optimized conditions. Besides the terminal styrenes derivatives, the internal styrenes with bulky steric hindrance were also investigated. A natural bioactive molecule 1,2-disubstituted styrene (*trans*-anethole, 27) and (*E*-) stilbene (28) proceeded smoothly to form the target product. Moreover, 1,1,2-trisubstituted styrene, which bears bulker hindrance, was also tolerated affording the aniline in 68% yield (26). To our delight, allylbenzene 29 could also furnish this C-C single bond cleavage due to the isomerization of the allyl group under acidic conditions. When styrene 30 bearing two alkenyls groups was employed as the substrate, two alkenyl groups on the aryl ring were cleavaged simultaneously affording benzene-1,4-diamine 9 in 78% yield.

**Figure 3:**
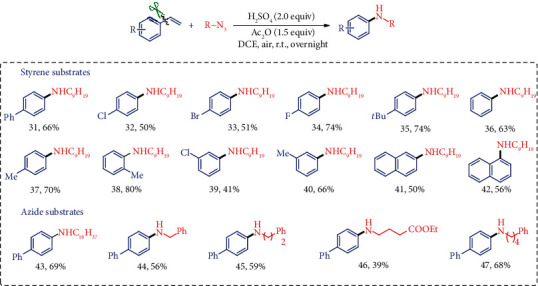
Substrate scope for the arylamine synthesis from styrenes. ^a^Standard conditions: reactions were performed with styrene (0.3 mmol), alkyl azide (0.6 mmol), Ac_2_O (0.45 mmol), and H_2_SO_4_ (0.6 mmol) in DCE (1.0 mL) at room temperature under air atmosphere overnight. Isolated yields. DCE: 1,2-dichloroethane.

Interestingly, this dealkenylative C-C bond nitrogenation chemistry could also be successfully expanded to synthesize alkyl-substituted arylamines with alkyl azides as the *N*-source under the conditions when employed H_2_SO_4_ (2.0 equiv) and Ac_2_O (1.5 equiv) as the additives in DCE (for the results in different conditions, see Supplementary Table S5). During the reaction screening and optimization for the arylamine synthesis, many additives had been tried for the transformation and found that the anhydride had promoted the reaction, but it was not indispensable for the process. Definitely, its actual role in this synthetic route was still not completely clear yet. As shown in Figure 3, a serious of styrenes containing substituents at the *para*-, *ortho*-, and *meta*-positions of the aromatic ring worked well and afforded the corresponding arylamines in moderate to good yields. Moreover, other alkyl azide reagents were tolerated in this transformation leading to various *N*-alkyl-substituted aniline products (43–47).

**Figure 4:**
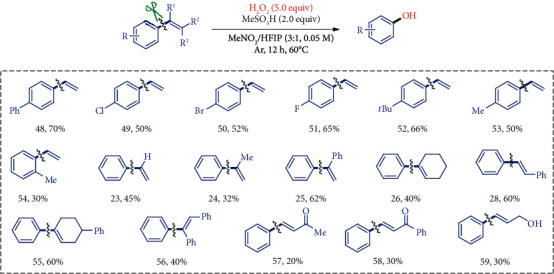
Substrate scope for the phenol synthesis from styrene. ^a^Standard conditions: reactions were performed with styrene (0.3 mmol), H_2_O_2_ (1.5 mmol), and MeSO_3_H (0.6 mmol) in MeNO_2_/HFIP (4.5 : 0.5 mL, 0.05 M) at 60°C for 12 h under Ar atmosphere and isolated yields. MeNO_2_: nitromethane; HFIP: hexafluoroisopropanol.

Although the epoxidation of styrenes was a known and favored process under oxidative conditions, inspired by the dealkenylative C-C bond amination results, we further investigated the C-C hydroxylation process with commercially available aqueous hydrogen peroxide as the oxygen source. Through the careful screening (see Supplementary Table S6), we optimized the conditions as MeSO_3_H (2.0 equiv) and H_2_O_2_ (30%, 5.0 equiv) in MeNO_2_/HFIP (4.5/1.5 mL, 0.05 M) stirring at 60°C. The reaction of styrenes under these conditions could afford the designed phenols by the novel dealkenylative C-C bond oxygenation process. The low reaction concentrations, the type of solvent, and acid were crucial to suppress the undesired by-products such as polymerization and epoxidation. As shown in Figure 4, a series of alkenyl groups on the styrenes (23–26, 28, 55, 56) were successfully replaced by the hydroxyl group to give the phenol products in moderate to good yields. Notably, the very active chalcones (57, 58) and cinnamyl alcohol (59) also worked albeit in low efficiency.

**Figure 5:**
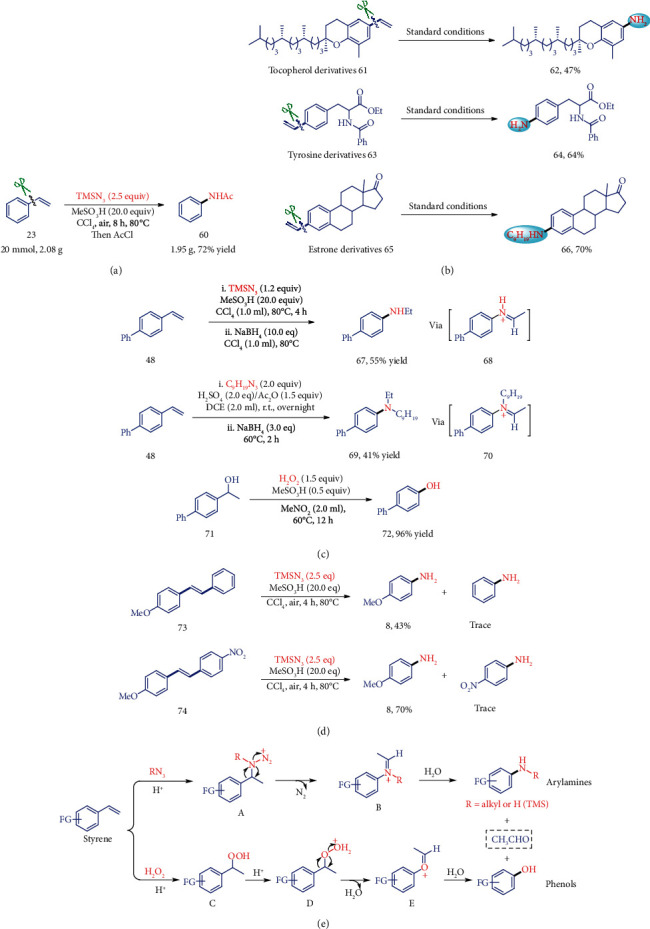
Synthetic applications and mechanism study of the dealkenylative C–C bond functionalization. (a) A gram-scale reaction with substrate 23 under standard conditions. (b) Modification of some pharmaceutical derivatives by this C-C amination protocol. (c) Mechanistic studies for the determination of the reaction intermediate. The formation of 67 and 69 suggests that the imine 68 and 70 are involved as the intermediates. The transformation from 71 to 72 shows the possible benzylic cation intermediate. (d) Regiochemistry exploration for this process. (e) The proposed mechanism of this dealkenylative C-C functionalization.

To further demonstrate the utility of this transformation, we carried out gram-scale reactions with styrene (23) as the substrate which is a bulk chemical from natural sources and coal/petroleum products. The reaction offered the aniline in good yield, indicating its potential industrial application possibility (Figure 5(a)). In addition, the late-stage functionalization of complex bioactive molecules was further evaluated. Interestingly, 61 derived from (+)-*δ*-tocopherol was proven to be tolerated in this carbon-carbon amination process, affording the corresponding 62 in 47% yield. Additionally, the alkene-containing tyrosine derivative (63) and estrone derivative (65) could also furnish this transformation in good efficiency, giving 64 and 66 in 64% and 70% yield, respectively (Figure 5(b)).

Moreover, in order to testify the intermediates of this process and trace the alkenyl group, we first conducted an in situ reduction reaction with regard to the carbon-carbon amination procedure with NaBH_4_ as the hydrogenative reagent, and arylamine 67 and 69 were produced in 55% and 41% yields, respectively, which indicates that the protonated imine 68 and 70 might be the key intermediates of this transformation. The result of the benzyl alcohol 71 under this C-C hydroxylation conditions quantitatively yielding the corresponding phenol (Figure 5(c)) suggests that the benzylic cation is probably involved in this oxygenation process. To explore the regiochemistry for the dealkenylative transformation, substrates of 73 and 74 have been conducted under the standard conditions (Figure 5(d)). High regioselectivities were obtained in these cases which was controlled by the stability of the generated benzylic carbon cation intermediate during the hydroazidation of alkene.

On the basis of the above results and previous reports [46, 54–59], the mechanism of this transformation was described in Figure 5(e). Initially, the acid-assisted hydroazidation of the C=C double-bond of styrenes occurs to generate the intermediate A with Markovnikov's rule [54, 55], which undergoes the subsequent Schmidt-type rearrangement process to afford the imine intermediate B through the cleavage of the C(Ar)-C(alkenyl) single bond [46, 56–59]. The final hydrolysis of species B produces the desired anilines and aldehyde side products. Alternatively, a similar process occurs for the styrene substrate to generate the intermediate C in situ, which undergoes the traditional Hock process [60] to produce the phenol product.

## 3. Conclusions

This chemistry has described a novel carbon-carbon amination and hydroxylation of styrenes for the efficient and site-specific synthesis of arylamines and phenols. Significantly, this protocol provides a highly selective dealkenylative C-C bond activation mode of styrenes under transition-metal free and redox-neutral conditions with azide reagents as the nitrogenaton reagents or aqueous hydrogen peroxide as the oxygen source. Compared to the poor regioselectivity and limited substrate scope in the typical aromatic C-H amination and hydroxylation process, this chemistry features site-directed selectivity and broad substrate scope. The simple and mild conditions make it applicable to the late-stage modification of some bioactive molecules. This strategy may open new avenues for the development of other novel transformations of alkenes through the C-C bond cleavage.

## 4. Methods

### 4.1. General C-C Amination Procedure

The substrate alkenes (0.3 mmol, 1.0 euiv), TMSN_3_ (0.75 mmol, 2.5 equiv), and CCl_4_ (1.0 mL), were added into a 20 mL vial equipped with a stir bar. Then, MeSO_3_H (6.0 mmol, 20.0 equiv) was added. The reaction was refluxed under air at 80°C for 4 h. After cooling down to room temperature, the reaction mixture was quenched by 2 M NaOH (5 mL) and extracted by EA (5 × 2mL), and the combined organic phase was washed with brine and dried over Na_2_SO_4_. Then, the mixture was concentrated and purified by flash chromatography on a short silica gel (eluent: PE/EA = 10/1) to afford the desired anilines.

The substrate alkenes (0.2 mmol, 1.0 equiv), alkyl azide (0.4 mmol, 2.0 equiv), acetic anhydride (0.3 mmol, 1.5 equiv), and DCE (2.0 mL), were added into a 20 mL vial equipped with a stir bar. The mixture was stirred at 25°C. Then, *conc.* H_2_SO_4_ (0.4 mmol, 2.0 equiv) was added to the mixture in 5 seconds. The mixture was stirred at 25°C overnight. The reaction was quenched with 20% NaOH and was extracted with EA, purified by flash chromatography on a short silica gel (eluent: PE/EA = 50/1) to afford the desired arylamines.

### 4.2. General C-C hydroxylation procedure

The substrate alkenes (0.3 mmol, 1.0 euiv), MeNO_2_ (4.5 mL)/HFIP (1.5 mL), were added into a 20 mL vial equipped with a stir bar. Then, 30% aqueous hydrogen peroxide solution (1.5 mmol, 5.0 equiv) and MeSO_3_H (0.6 mmol, 2.0 equiv) were added in order. The reaction was heated under Ar at 60°C for 12 h. After cooling down to room temperature, the reaction mixture was quenched by *sat*. NaHCO_3_ (5 mL) and extracted by EA (5 × 2mL), and the combined organic phase was washed with brine and dried over Na_2_SO_4_. Then, the mixture was concentrated and purified by flash chromatography on a short silica gel (eluent: PE/EA = 10/1) to afford the desired phenols.

## Supplementary Materials

Supplementary Materials Table S1: the effects of solvents for the synthesis of anilines. Table S2: the effects of additives for the synthesis of anilines. Table S3: the effects of acid for the synthesis of anilines. Table S4: the effects of nitrogenation reagents and temperature for the synthesis of anilines. Table S5: the reaction optimization for the synthesis of arylamines. Table S6: the reaction optimization for the synthesis of phenols. The synthesis of substrates. General procedures. 1H NMR and 13C NMR spectra of product.
