# Supplementary material for: Selective Dealkenylative Functionalization of Styrenes via C-C Bond Cleavage
Source: Research (Wash D C). 2020 Nov 10;2020:7947029. doi: 10.34133/2020/7947029 (PMC7676249; doi:10.34133/2020/7947029)
Supplement: Supplementary Materials — Table S1: the effects of solvents for the synthesis of anilines. Table S2: the effects of additives for the synthesis of anilines. Table S3: the effects of acid for the synthesis of anilines. Table S4: the effects of nitrogenation reagents and temperature for the synthesis of anilines. Table S5: the reaction optimization for the synthesis of arylamines. Table S6: the reaction optimization for the synthesis of phenols. The synthesis of substrates. General procedures. 1H NMR and 13C NMR spectra of product. [file 7947029.f1.zip › Description of supplementary material .docx]

Supplementary Materials

Table S1: The effects of solvents for the synthesis of anilines. Table S2: The effects of additives for the synthesis of anilines. Table S3: The effects of acid for the synthesis of anilines. Table S4: The effects of nitrogenation reagents and temperature for the synthesis of anilines. Table S5: The reaction optimization for the synthesis of arylamines. Table S6: The reaction optimization for the synthesis of phenols. The synthesis of substrates. General procedures. ^1^H NMR and ^13^C NMR spectra of product.
