# Supplementary material for: Selective Dealkenylative Functionalization of Styrenes via C-C Bond Cleavage
Source: Research (Wash D C). 2020 Nov 10;2020:7947029. doi: 10.34133/2020/7947029 (PMC7676249; doi:10.34133/2020/7947029)
Supplement: Supplementary Materials — Table S1: the effects of solvents for the synthesis of anilines. Table S2: the effects of additives for the synthesis of anilines. Table S3: the effects of acid for the synthesis of anilines. Table S4: the effects of nitrogenation reagents and temperature for the synthesis of anilines. Table S5: the reaction optimization for the synthesis of arylamines. Table S6: the reaction optimization for the synthesis of phenols. The synthesis of substrates. General procedures. 1H NMR and 13C NMR spectra of product. [file 7947029.f1.zip › Revised Supplemental Material.pdf]

Supplementary Information

Selective dealkenylative functionalization of styrenes via C-C single bond cleavage

Jianzhong Liu,[†,§] Jun Pan,[†,§] Xiao Luo,[†] Xu Qiu,[†] Cheng Zhang,[†] and Ning Jiao*[,†,‡]

[†]State Key Laboratory of Natural and Biomimetic Drugs, Peking University, 100191 Beijing, China.

[‡]State Key Laboratory of Organometallic Chemistry, Chinese Academy of Sciences, Shanghai 200032, China.

[§] These authors contributed equally.

E-mail: jiaoning@pku.edu.cn

Fax: (+86)10-82805297

Table of Contents

A. General information	S2
B. Optimization details for the C-C amination and hydroxylation	S3-S8
C. The synthesis of substrates	S9
D. General procedure for C(Ar)-C(Alkenyl) single bond amination of styrenes	S10-S29
E. General procedure for C(Ar)-C(Alkenyl) single bond hydroxylation of styrenes	S30-S35
F. Synthetic applications of the de-alkenylating C-C bond functionalization	S36-S38
G. Mechanistic experiments	S39-S41
H. References	S42-S43
I. ^{1}H NMR and ^{13}C NMR Spectra of Products	S44-S153

A. General information

All commercially available compounds were purchased from Sigma-Aldrich, TCI, J&K, Alfa-Aesar, Acros, Beijing Ouhe and Beijing Chemical Works, Ltd. Unless otherwise noted, materials obtained from commercial suppliers were used without further purification. Analysis of crude reaction mixture was done on an Agilent 7890 GC System with an Agilent 5975 Mass Selective Detector. Products were purified by flash chromatography on silica gel. ^{1}H-NMR spectra were recorded on Bruker AVANCE III-400 spectrometers. Chemical shifts (in ppm) were referenced $CDCl_3$ (7.27 ppm), DMSO-d_6 (2.50 ppm). ^{13}C-NMR spectra were obtained by using the same NMR spectrometers and were calibrated with $CDCl_3$ (δ = 77.00 ppm), DMSO-d_6 (δ = 39.50 ppm). Mass spectra were recorded using a PE SCLEX QSTAR spectrometer. High resolution mass spectra were obtained with a Bruker APEX IV Fourier transform ion cyclotron resonance mass spectrometer.

Safety caution: We have NOT encountered any explosion during the handling of azide species in all procedures listed in this document. However, for safety concerns, sodium azide (NaN_3) and all organic azide compounds should be treated as explosive and toxic substances. When handling sodium azide, organic azide compounds, care should be taken to avoid strong mechanical shock or friction, and contact with metal apparatus (syringe needle, metal spatula, etc.) should be avoided. Instead, we recommend using a plastic spatula or plastic pipette.

B. Optimization details for the C-C amination and hydroxylation

Table 1. The effects of solvents for the synthesis of anilines[a]

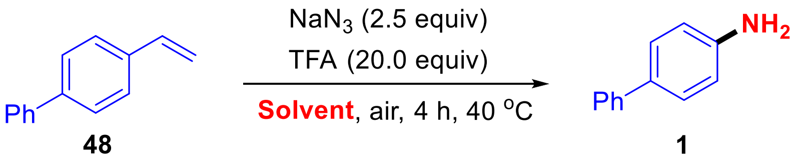

Entry	Solvent	Yield of **1**
1	DCE	0%
2	$CHCl_3$	27%
**3**	**CCl_4**	**40%**
4	TCE	35%
5	Trichloroethane	Trace
6	PhCl	Trace
7	CH_3CN	0%
8	$MeNO_2$	<5%
9	n-Hexane	35%
10	HFIP	Trace
11	TFE	Trace
12	1,4-Dioxane	0%
13	DMF	0%
14	DMSO	0%
15	EA	0%
16	1,2-Dimethoxyethane	0%

[a] Reaction conditions: **48** (0.30 mmol), NaN_3 (0.75 mmol), TFA (6.0 mmol) in Solvent (1.0 mL) was stirred at 40 °C under Air for 4 h. Isolated yield.

Table 2. The effects of additives for the synthesis of anilines[a]

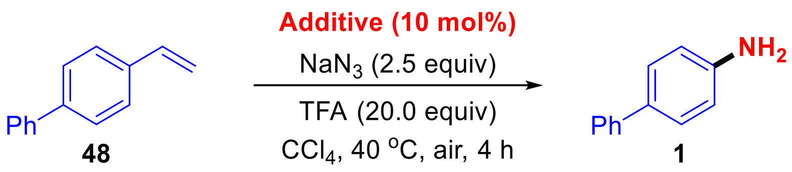

Entry	Additive	Yield of **1**
1	TBAI	23%
2	TBAB	40%
3	TBAC	40%
4	KI	28%
5	NaOAc	33%
6	15-Crown-5	41%
7	Li_2CO_3	38%
8	NaCl	38%
9	PhCOONa	31%
10	PhCOOK	31%
11	H_2O	39%
**12**	**none**	**40%**

[a] Reaction conditions: **48** (0.30 mmol), NaN_3 (0.75 mmol), Additive (0.03 mmol), TFA (6.0 mmol) in CCl_4 (1.0 mL) was stirred at 40 °C under Air for 4 h. Isolated yield.

Table 3. The effects of acid for the synthesis of anilines[a]

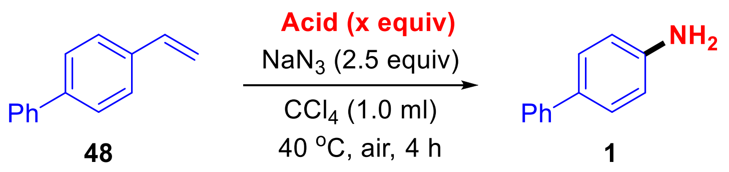

Entry	Acid	X	Yield of **1**
1	TFA	20	40%
**2**	**$MeSO_3H$**	**20**	**67%**
3	TfOH	20	35%
4	H_2SO_4	20	24%
5	$MeSO_3H$	1	0%
6	$MeSO_3H$	2	0%
7	$MeSO_3H$	4	0%
8	$MeSO_3H$	8	24%
9	$MeSO_3H$	16	41%
10	$MeSO_3H$	24	60%

[a] Reaction conditions: **48** (0.30 mmol), NaN_3 (0.75 mmol) in CCl_4 (1.0 mL) was added the indicated acid and continued stirred at 40 °C under Air for 4 h. Isolated yield.

Table 4. The effects of nitrogenation reagents and temperature for the synthesis of anilines[a]

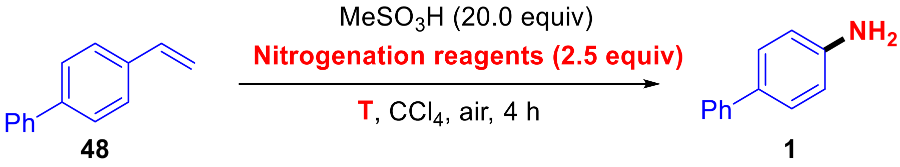

Entry	Nitrogenation reagents	T	Yield of **1**
1	NaN_3	40	67%
2	NaN_3	20	61%
3	NaN_3	60	78%
4	NaN_3	80	80%
**5**	**$TMSN_3$**	**80**	**86%**
6	$NaNO_2$	80	0%
7	NH_2OH	80	0%
8	TBN	80	0%
9	TsN_3	80	0%

[a] Reaction conditions: **48** (0.30 mmol), Nitrogenation reagents (0.75 mmol), $MeSO_3H$ (6.0 mmol) in CCl_4 (1.0 mL) was stirred at 40 °C under Air for 4 h. Isolated yield.

Table 5. The reaction optimization for the synthesis of arylamines[a]

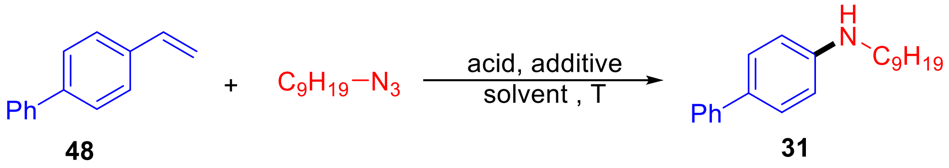

entry	acid	additive	solvent	T	yield (%)[b]
1	CH_3SO_3H (2 equiv)	/	DCE	60 °C	31
2	CF_3SO_3H (2 equiv)	/	DCE	60 °C	42
3	CF_3COOH (2 equiv)	/	DCE	60 °C	10
4	CH_3COOH (2 equiv)	/	DCE	60 °C	0
5	H_2SO_4 (2 equiv)	/	DCE	60 °C	49
6	H_2SO_4 (4 equiv)	/	DCE	60 °C	43
7	H_2SO_4 (2 equiv)	/	DMSO	60 °C	0
8	H_2SO_4 (2 equiv)	/	Cyclohexane	60 °C	34
9	H_2SO_4 (2 equiv)	/	THF	60 °C	0
10	H_2SO_4 (2 equiv)	KI (2 equiv)	DCE	60 °C	33
11	H_2SO_4 (2 equiv)	LiCl (2 equiv)	DCE	60 °C	31
12	H_2SO_4 (2 equiv)	Ac_2O (1.5 equiv)	DCE	60 °C	71 (64)
**13**	**H_2SO_4 (2 equiv)**	**Ac_2O (1.5 equiv)**	**DCE**	**25 °C**	**74 (66)**

[a] Reaction conditions: **48** (0.20 mmol), alkyl azide (0.4 mmol), acid in solvent (2.0 mL) was stirred at 25 °C overnight under Air. Isolated yield. Yield was determined by H[1] NMR using 1,1,2,2-tetrachloroethane as internal standard. The yield in parenthesis was isolated yield.

Table 6. The reaction optimization for the synthesis of phenols[a]

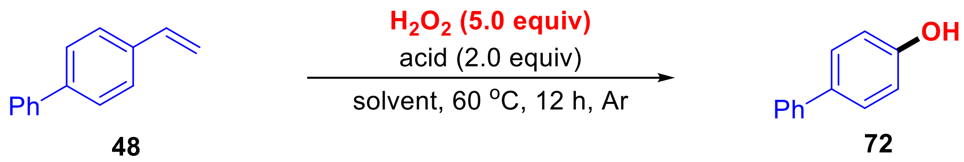

entry	acid	solvent	yield (%)
1	CH_3SO_3H	CH_3CN (2.0 mL)	25
2	CH_3SO_3H	DCE (2.0 mL)	20
3	CH_3SO_3H	n-Hexane (2.0 mL)	trace
4	CH_3SO_3H	TFE (2.0 mL)	ND
5	CH_3SO_3H	$MeNO_2$ (2.0 mL)	30
6	CH_3SO_3H	HFIP (2.0 mL)	ND
7	CH_3SO_3H	$MeNO_2$/HFIP (1.5:0.5 mL)	50
8	HCl	$MeNO_2$/HFIP (1.5:0.5 mL)	ND
9	H_2SO_4	$MeNO_2$/HFIP (1.5:0.5 mL)	30
10	AcOH	$MeNO_2$/HFIP (1.5:0.5 mL)	ND
11	TFA	$MeNO_2$/HFIP (1.5:0.5 mL)	26
12	CF_3SO_3H	$MeNO_2$/HFIP (1.5:0.5 mL)	15
13	CH_3SO_3H	$MeNO_2$/HFIP (1.8:0.6 mL)	58
14	CH_3SO_3H	$MeNO_2$/HFIP (2.4:0.8 mL)	58
15	CH_3SO_3H	$MeNO_2$/HFIP (3.0:1.0 mL)	60
**16**	**CH_3SO_3H**	**$MeNO_2$/HFIP (4.5:1.5 mL)**	**70**

[a] Reaction conditions: **48** (0.30 mmol), H_2O_2 (30% aqueous hydrogen peroxide solution, 1.5 mmol), acid (0.60 mmol) in solvent was stirred at 60 °C under Ar for 12 h. Isolated yield.

C. The synthesis of substrates

The common styrene substrates were commercially available and the substrates of **82**[1], **83**[1], **90**[2], **61**[3] and **63**[4], **65**[4] were synthesized as the corresponding literature. The synthesis of the substrate **30** was described as follows:

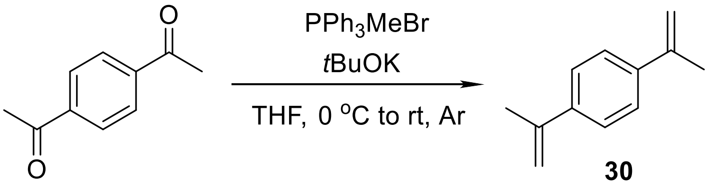

30

1,4-di(prop-1-en-2-yl)benzene (30):[5]

Methyltriphenylphosphonium bromide (8.6 g, 24 mmol), Potassium tert-butoxide (4.4 g, 40 mmol), were dissolved in anhydrous THF (20 mL). The reaction mixture was stirred under Ar for 30 min at 0 °C, then 1,1'-(1,4-phenylene)bis(ethan-1-one) (1.6 g, 10 mmol) was added and stirred at rt for 5 h, poured into sat. NaCl (aq.) and extracted with EA. The organic layer was separated and dried over Na_2SO_4. Then the mixture was concentrated and purified by flash chromatography on silica gel to afford 1.3 g (80%) of **30**, white solid. **^{1}H NMR ($CDCl_3$, 400 MHz)**: δ 7.49 (s, 4H), 5.45 (s, 2H), 5.14 (s, 2H), 2.21 (s, 6H). **^{13}C NMR ($CDCl_3$, 100 MHz)**: δ 142.78, 140.20, 125.30, 112.22, 21.69 ppm.; MS (70 eV): m/z (%) =158.1.

D. General procedure for C(Ar)-C(Alkenyl) single bond amination of styrenes.

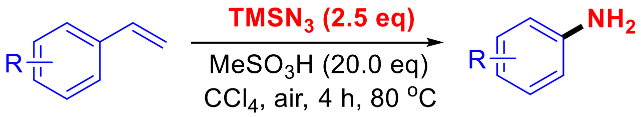

To a 20 mL vial equipped with a stir bar was added alkenes (0.3 mmol), $TMSN_3$ (86.4 mg, 0.75 mmol), CCl_4 (1.0 mL), followed by the addition of $MeSO_3H$ (390 uL, 6.0 mmol). The reaction was refluxed under air at 80 °C for 4 h. After cooling down to room temperature, the reaction mixture was quenched by 2 M NaOH (5 mL), extracted by EA (5 × 2 mL), the combined organic phase were washed with brine and dried over Na_2SO_4. Then the mixture were concentrated and purified by flash chromatography on a short silica gel (eluent: PE/EA = 10/1) to afford the desired anilines.

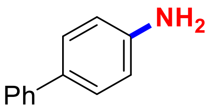

1

4-Aminobiphenyl (2a):[6]

The reaction of 4-vinyl-1,1'-biphenyl **48** (54.1 mg, 0.3 mmol), $TMSN_3$ (86.4 mg, 0.75 mmol) in CCl_4 (1.0 mL) followed by the addition of $MeSO_3H$ (390 uL, 6.0 mmol), then the mixture was stirred under air at 80 °C for 4 h to afford 43.6 mg (86%) of **1.** Yellow solid. **^{1}H NMR ($CDCl_3$, 400 MHz)**: δ 7.59 (d, *J* = 7.2 Hz, 2H), 7.49–7.41 (m, 4H), 7.32 (t, *J* = 7.4 Hz, 1H), 6.79 (d, *J* = 8.5 Hz, 2H);3.69 (s, 2H); **^{13}C NMR ($CDCl_3$, 100 MHz)**: δ 145.78, 141.09, 131.48, 128.60, 127.93, 126.33, 126.19, 115.33 ppm; MS (70 eV): m/z (%) =169.1.

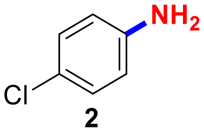

2

4-Chloroaniline (2):[6]

The reaction of 1-chloro-4-vinylbenzene **49** (41.4 mg, 0.3 mmol), $TMSN_3$ (86.4 mg, 0.75 mmol) in CCl_4 (1.0 mL) followed by the addition of $MeSO_3H$ (390 uL, 6.0 mmol), then the mixture was stirred under air at 80 °C for 4 h to afford 30.5 mg (80%) of **2.** Yellow solid. **^{1}H NMR ($CDCl_3$, 400 MHz)**: δ 7.10 (d, *J* = 8.7 Hz, 2H), 6.60 (d, *J* = 8.7 Hz, 2H), 3.62 (s, 2H); **^{13}C NMR ($CDCl_3$, 100 MHz)**: δ 144.91, 129.02, 123.00, 116.15 ppm; MS (70 eV): m/z (%) =127.1.

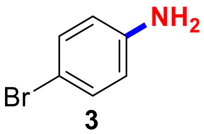

3

4-Bromoaniline (3):[6]

The reaction of 1-bromo-4-vinylbenzene **50** (54.6 mg, 0.3 mmol), $TMSN_3$ (86.4 mg, 0.75 mmol) in CCl_4 (1.0 mL) followed by the addition of $MeSO_3H$ (390 uL, 6.0 mmol), then the mixture was stirred under air at 80 °C for 4 h to afford 41.0 mg (80%) of **3.** Yellow solid. **^{1}H NMR ($CDCl_3$, 400 MHz)**: δ 7.23 (d, J = 8.7 Hz, 2H), 6.56 (d, J = 8.7 Hz, 2H), 3.66 (s, 2H).; **^{13}C NMR ($CDCl_3$, 100 MHz)**: δ 145.37, 131.95, 116.66, 110.12 ppm; MS (70 eV): m/z (%) =171.1.

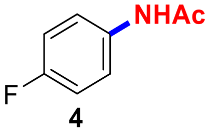

4

N-(4-fluorophenyl)acetamide (4): [7]

The reaction of 1-fluoro-4-vinylbenzene **51** (36.6 mg, 0.3 mmol), $TMSN_3$ (86.4 mg, 0.75 mmol) in CCl_4 (1.0 mL) followed by the addition of $MeSO_3H$ (390 uL, 6.0 mmol), then the mixture was stirred under air at 80 °C for 4 h**.** After cooling down to room temperature, the reaction mixture was quenched by 2 M NaOH (5 mL), extracted by EA (5 × 2 mL), the combined organic phase were washed with brine and dried over Na_2SO_4. Then the mixture were concentrated and AcCl (35.3 mg, 0.45 mmol), Et_3N (45.5 mg, 0.45 mmol) and DCM (5.0 mL) were added and stirred at rt for 6 h. The mixture were concentrated and purified by flash chromatography on a short silica (eluent: PE/EA = 10/1) to afford 31.9 mg (63%) of **4**. Yellow solid. **^{1}H NMR (DMSO-d_6, 400 MHz)**: δ 9.96 (s, 1H), 7.58 (dd, J = 7.5, 5.2 Hz, 2H), 7.11 (t, J = 8.5 Hz, 3H), 2.03 (s, 3H).; **^{13}C NMR (DMSO-d_6, 100 MHz)**: δ 168.08, 157.77 (d, J = 238.0 Hz), 135.67, 120.64 (d, J = 7.7 Hz), 115.11 (d, J = 22.0 Hz), 23.79 ppm; **^{19}F NMR (376 MHz, DMSO-d_6)** δ -119.83. MS (70 eV): m/z (%) =153.1.

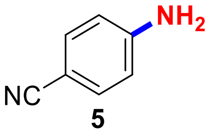

5

4-Aminobenzonitrile (5):[8]

The reaction of 4-vinylbenzonitrile **75** (38.7 mg, 0.3 mmol), $TMSN_3$ (86.4 mg, 0.75 mmol) in CCl_4 (1.0 mL) followed by the addition of $MeSO_3H$ (390 uL, 6.0 mmol), then the mixture was stirred

under air at 40 °C for 4 h to afford 15.6 mg (44%) of **5.** Yellow solid. **^{1}H NMR ($CDCl_3$, 400 MHz)**: δ 7.41 (d, *J* = 8.7 Hz, 2H), 6.64 (d, *J* = 8.7 Hz, 2H), 4.15 (s, 2H). **^{13}C NMR ($CDCl_3$, 100 MHz)**: δ 150.33, 133.78, 120.06, 114.41, 100.24. MS (70 eV): m/z (%) =118.1.

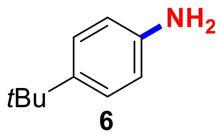

6

4-(Tert-butyl)aniline (6):[8]

The reaction of 1-(tert-butyl)-4-vinylbenzene **52** (48.0 mg, 0.3 mmol), $TMSN_3$ (86.4 mg, 0.75 mmol) in CCl_4 (1.0 mL) followed by the addition of $MeSO_3H$ (390 uL, 6.0 mmol), then the mixture was stirred under air at 80 °C for 4 h to afford 37.1 mg (83%) of **6.** Yellow oil. **^{1}H NMR ($CDCl_3$, 400 MHz)**: δ 7.22 (d, *J* = 8.3 Hz, 2H), 6.68 (d, *J* = 8.3 Hz, 2H), 3.54 (s, 2H), 1.32 (s, 9H); **^{13}C NMR ($CDCl_3$, 100 MHz)**: δ 143.75, 141.33, 125.99, 114.87, 33.84, 31.48 ppm. MS (70 eV): m/z (%) =149.2.

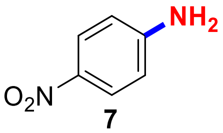

7

4-Nitroaniline (7):[6]

The reaction of 1-nitro-4-vinylbenzene **76** (44.7 mg, 0.3 mmol), $TMSN_3$ (86.4 mg, 0.75 mmol) in CCl_4 (1.0 mL) followed by the addition of $MeSO_3H$ (390 uL, 6.0 mmol), then the mixture was stirred under air at 80 °C for 4 h to afford 29.8 mg (72%) of **7.** Yellow solid. **^{1}H NMR ($CDCl_3$, 400 MHz)**: δ 8.10–8.04 (m, 2H), 6.66–6.60 (m, 2H), 4.39 (s, 2H); **^{13}C NMR ($CDCl_3$, 100 MHz)**: δ 152.46, 126.34, 113.35 ppm; MS (70 eV): m/z (%) =138.1.

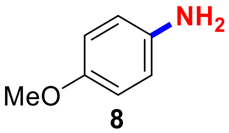

8

4-Methoxyaniline (8):[6]

1) **From 77, Table 1 in Text**:

The reaction of 1-methoxy-4-vinylbenzene **77** (40.2 mg, 0.3 mmol), $TMSN_3$ (86.4 mg, 0.75 mmol) in CCl_4 (1.0 mL) followed by the addition of $MeSO_3H$ (98 uL, 1.5 mmol), then the mixture was stirred under air at 80 °C for 4 h to afford 21.0 mg (57%) of **8.** Yellow solid. **^{1}H NMR ($CDCl_3$, 400 MHz)**: δ 6.75 (d, *J* = 8.8 Hz, 2H), 6.65 (d, *J* = 8.8 Hz, 2H), 3.74 (s, 3H), 3.16 (s, 2H); **^{13}C NMR ($CDCl_3$,**

100 MHz): δ 152.80, 139.84, 116.42, 114.77, 55.70 ppm; MS (70 eV): m/z (%) =123.1.

2) **From 27, Table 1 in Text**:

The reaction of (E)-1-methoxy-4-(prop-1-en-1-yl)benzene **27** (44.4 mg, 0.3 mmol), $TMSN_3$ (86.4 mg, 0.75 mmol) in CCl_4 (1.0 mL) followed by the addition of $MeSO_3H$ (98 uL, 1.5 mmol), then the mixture was stirred under air at 80 °C for 4 h to afford 19.6 mg (53%) of **8.**

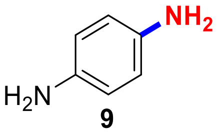

9

Benzene-1,4-diamine (9):[9]

1) **From 78, Table 1 in Text**:

The reaction of 4-vinylaniline **78** (35.7 mg, 0.3 mmol), $TMSN_3$ (86.4 mg, 0.75 mmol) in CCl_4 (1.0 mL) followed by the addition of $MeSO_3H$ (390 uL, 6.0 mmol), then the mixture was stirred under air at 80 °C for 4 h to afford 13.6 mg (42%) of **9.** Yellow solid. **^{1}H NMR ($CDCl_3$, 400 MHz)**: δ 6.57 (s, 4H), 3.33 (s, 4H); **^{13}C NMR ($CDCl_3$, 100 MHz)**: δ 138.56, 116.67 ppm. MS (70 eV): m/z (%) =108.1.

2) **From 30, Table 1 in Text**:

The reaction of 1,4-di(prop-1-en-2-yl)benzene **30** (47.4 mg, 0.3 mmol), $TMSN_3$ (172.8 mg, 1.5 mmol) in CCl_4 (1.0 mL) followed by the addition of $MeSO_3H$ (390 uL, 6.0 mmol), then the mixture was stirred under air at 80 °C for 4 h to afford 25.2 mg (78%) of **9.**

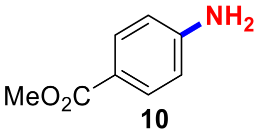

10

Methyl 4-aminobenzoate (10):[6]

The reaction of methyl 4-vinylbenzoate **79** (48.6 mg, 0.3 mmol), $TMSN_3$ (86.4 mg, 0.75 mmol) in CCl_4 (1.0 mL) followed by the addition of $MeSO_3H$ (390 uL, 6.0 mmol), then the mixture was stirred under air at 40 °C for 4 h**.** After cooling down to room temperature, the reaction mixture was quenched by sat. $NaHCO_3$ (aq.) (6 mL), extracted by EA (5 × 2 mL), the combined organic phase were washed with brine and dried over Na_2SO_4. Then the mixture were concentrated and purified by flash chromatography on a short silica gel (eluent: PE/EA = 10/1) to afford 24.9 mg (55%) of **10.** Yellow solid. **^{1}H NMR ($CDCl_3$, 400 MHz)**: δ 7.84 (d, *J* = 8.6 Hz, 2H), 6.62 (d, *J* = 8.6 Hz, 2H), 4.10

(s, 2H), 3.84 (s, 3H); **^{13}C NMR ($CDCl_3$, 100 MHz)**: δ 167.15, 150.85, 131.53, 119.55, 113.71, 51.54 ppm; MS (70 eV): m/z (%) =151.1.

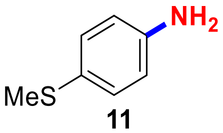

11

4-(Methylthio)aniline (11): [6]

The reaction of methyl(4-vinylphenyl)sulfane **80** (45.1 mg, 0.3 mmol), $TMSN_3$ (86.4 mg, 0.75 mmol) in CCl_4 (1.0 mL) followed by the addition of $MeSO_3H$ (98 uL, 1.5 mmol), then the mixture was stirred under air at 80 °C for 4 h to afford 20.9 mg (50%) of **11.** Yellow oil. **^{11}H NMR ($CDCl_3$, 400 MHz)**: δ 7.18 (d, J = 8.5 Hz, 2H), 6.63 (d, J = 8.5 Hz, 2H), 3.64 (s, 2H), 2.41 (s, 3H); **^{13}C NMR ($CDCl_3$, 100 MHz)**: δ 145.04, 131.00, 125.72, 115.68, 18.73 ppm; MS (70 eV): m/z (%) =139.1.

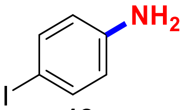

12

4-Iodoaniline (12): [6]

The reaction of 1-iodo-4-vinylbenzene **81** (69.0 mg, 0.3 mmol), $TMSN_3$ (86.4 mg, 0.75 mmol) in CCl_4 (1.0 mL) followed by the addition of $MeSO_3H$ (390 uL, 6.0 mmol), then the mixture was stirred under air at 80 °C for 4 h to afford 45.3 mg (69%) of **12.** Yellow solid. **^{1}H NMR ($CDCl_3$, 400 MHz)**: δ 7.40 (d, J = 8.7 Hz, 2H), 6.47 (d, J = 8.7 Hz, 2H), 3.67 (s, 2H); **^{13}C NMR ($CDCl_3$, 100 MHz)**: δ 146.02, 137.87, 117.26, 79.33 ppm; MS (70 eV): m/z (%) =219.0.

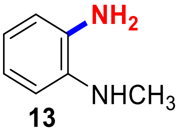

13

***N*^{1}-methylbenzene-1,2-diamine (13):** [10]

The reaction of N-methyl-2-vinylaniline **82** (39.9 mg, 0.3 mmol), $TMSN_3$ (86.4 mg, 0.75 mmol) in CCl_4 (1.0 mL) followed by the addition of $MeSO_3H$ (390 uL, 6.0 mmol), then the mixture was stirred under air at 80 °C for 4 h to afford 22.0 mg (60%) of **13.** Yellow oil. **^{1}H NMR ($CDCl_3$, 400 MHz)**: δ 6.87 (td, J = 7.7, 1.9 Hz, 1H), 6.76–6.65 (m, 3H), 3.33 (s, 3H), 2.87 (s, 3H); **^{13}C NMR ($CDCl_3$, 100 MHz)**: δ 138.96, 134.02, 120.78, 118.38, 116.26, 110.89, 30.92 ppm; MS (70 eV): m/z (%) =122.1

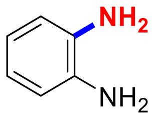

14

Benzene-1,2-diamine (14): [10]

The reaction of 2-vinylaniline **83** (35.7 mg, 0.3 mmol), $TMSN_3$ (86.4 mg, 0.75 mmol) in CCl_4 (1.0 mL) followed by the addition of $MeSO_3H$ (390 uL, 6.0 mmol), then the mixture was stirred under air at 80 °C for 4 h to afford 21.1 mg (65%) of **14.** Yellow solid. **^{1}H NMR ($CDCl_3$, 400 MHz)**: δ 6.77–6.68 (m, 4H), 3.38 (s, 4H); **^{13}C NMR ($CDCl_3$, 100 MHz)**: δ = δ 134.70, 120.22, 116.70 ppm; MS (70 eV): m/z (%) =108.1

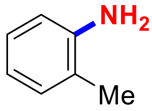

15

***O*-toluidine (15):** [6]

The reaction of 1-methyl-2-vinylbenzene **54** (35.4 mg, 0.3 mmol), $TMSN_3$ (86.4 mg, 0.75 mmol) in CCl_4 (1.0 mL) followed by the addition of $MeSO_3H$ (390 uL, 6.0 mmol), then the mixture was stirred under air at 80 °C for 4 h. After cooling down to room temperature, quenched by 2 M NaOH (5 mL) and extracted by EA, the combined organic phase were concentrated. Owing to the volatilization and low-boiling point of the product, the yield of **15** was determined by HNMR using 1,1,2,2-Tetrachloroethane as internal standard. NMR yield of **15** in this reaction is 81%. Yellow oil. **^{1}H NMR ($CDCl_3$, 400 MHz)**: δ 6.99 (t, J = 7.1 Hz, 2H), 6.67 (t, J = 7.4 Hz, 1H), 6.57 (d, J = 7.6 Hz, 1H), 3.46 (s, 2H), 2.08 (s, 3H); **^{13}C NMR ($CDCl_3$, 100 MHz)**: δ 144.42, 130.19, 126.71, 122.03, 118.29, 114.67, 17.05 ppm; MS (70 eV): m/z (%) =107.2.

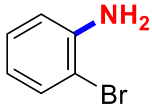

16

2-Bromoaniline (16): [6]

The reaction of 1-bromo-2-vinylbenzene **84** (54.6 mg, 0.3 mmol), $TMSN_3$ (86.4 mg, 0.75 mmol) in CCl_4 (1.0 mL) followed by the addition of $MeSO_3H$ (390 uL, 6.0 mmol), then the mixture was stirred under air at 80 °C for 4 h to afford 35.9 mg (70%) of **16**. Yellow oil. **^{1}H NMR ($CDCl_3$, 400 MHz)**: δ 7.42 (dd, J = 8.0, 1.1 Hz, 1H), 7.14–7.08 (m, 1H), 6.77 (dd, J = 8.0, 1.3 Hz, 1H), 6.68– 6.60

(m, 1H), 4.08 (s, 2H); **^{13}C NMR ($CDCl_3$, 100 MHz)**: δ 144.01, 132.52, 128.28, 119.35, 115.70, 109.26 ppm; MS (70 eV): m/z (%) =171.0.

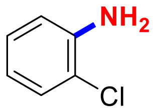

17

2-Chloroaniline (17): [9]

The reaction of 1-chloro-2-vinylbenzene **85** (41.4 mg, 0.3 mmol), $TMSN_3$ (86.4 mg, 0.75 mmol) in CCl_4 (1.0 mL) followed by the addition of $MeSO_3H$ (390 uL, 6.0 mmol), then the mixture was stirred under air at 80 °C for 4 h. After cooling down to room temperature, quenched by 2 M NaOH (5 mL) and extracted by EA, the combined organic phase were concentrated. Owing to the volatilization and low-boiling point of the product, the yield of **17** was determined by HNMR using 1,1,2,2-Tetrachloroethane as internal standard. NMR yield of **17** in this reaction is 65%.Yellow oil. **^{1}H NMR ($CDCl_3$, 400 MHz)**: δ 7.27 (dd, *J* = 8.0, 1.4 Hz, 1H), 7.12–7.05 (m, 1H), 6.78 (dd, *J* = 8.0, 1.5 Hz, 1H), 6.74–6.69 (m, 1H), 4.05 (s, 2H); **^{13}C NMR ($CDCl_3$, 100 MHz)**: δ 142.85, 129.35, 127.57, 119.22, 118.95, 115.81 ppm; MS (70 eV): m/z (%) =127.1.

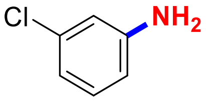

18

3-Chloroaniline (18): [11]

The reaction of 1-chloro-3-vinylbenzene **86** (35.4 mg, 0.3 mmol), $TMSN_3$ (86.4 mg, 0.75 mmol) in CCl_4 (1.0 mL) followed by the addition of $MeSO_3H$ (390 uL, 6.0 mmol), then the mixture was stirred under air at 80 °C for 4 h. After cooling down to room temperature, quenched by 2 M NaOH (5 mL) and extracted by EA, the combined organic phase were concentrated. Owing to the volatilization and low-boiling point of the product, the yield of **18** was determined by HNMR using 1,1,2,2-Tetrachloroethane as internal standard. NMR yield of **18** in this reaction is 76%. Yellow oil. **^{1}H NMR ($CDCl_3$, 400 MHz)**: δ 7.07 (t, *J* = 8.0 Hz, 1H), 6.74 (dd, *J* = 7.9, 1.1 Hz, 1H), 6.67 (t, *J* = 2.0 Hz, 1H), 6.57–6.52 (m, 1H), 3.71 (s, 2H). **^{13}C NMR ($CDCl_3$, 100 MHz)**: δ 147.57, 134.67, 130.22, 118.28, 114.79, 113.11 ppm; MS (70 eV): m/z (%) =127.1.

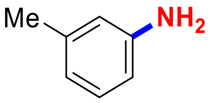

19

***M*-toluidine (19):** [6]

The reaction of 1-methyl-3-vinylbenzene **87** (35.4 mg, 0.3 mmol), $TMSN_3$ (86.4 mg, 0.75 mmol) in CCl_4 (1.0 mL) followed by the addition of $MeSO_3H$ (390 uL, 6.0 mmol), then the mixture was stirred under air at 80 °C for 4 h. After cooling down to room temperature, quenched by 2 M NaOH (5 mL) and extracted by EA, the combined organic phase were concentrated. Owing to the volatilization and low-boiling point of the product, the yield of **19** was determined by HNMR using 1,1,2,2-Tetrachloroethane as internal standard. NMR yield of **19** in this reaction is 80%.Yellow oil. **^{1}H NMR ($CDCl_3$, 400 MHz)**: δ 7.07 (t, *J* = 7.6 Hz, 1H), 6.61 (d, *J* = 7.5 Hz, 1H), 6.53 (dd, *J* = 5.7, 4.8 Hz, 2H), 3.54 (s, 2H), 2.29 (s, 3H); **^{13}C NMR ($CDCl_3$, 100 MHz)**: δ 146.29, 139.06, 129.10, 119.39, 115.86, 112.19, 21.37 ppm; MS (70 eV): m/z (%) =107.1.

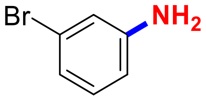

20

3-Bromoaniline (20): [12]

The reaction of 1-bromo-3-vinylbenzene **88** (54.6 mg, 0.3 mmol), $TMSN_3$ (86.4 mg, 0.75 mmol) in CCl_4 (1.0 mL) followed by the addition of $MeSO_3H$ (390 uL, 6.0 mmol), then the mixture was stirred under air at 80 °C for 4 h to afford 35.9 mg (70%) of **20**. Yellow oil. **^{1}H NMR ($CDCl_3$, 400 MHz)**: δ 7.02 (t, *J* = 8.0 Hz, 1H), 6.89 (d, *J* = 8.4 Hz, 1H), 6.83 (s, 1H), 6.58 (dd, *J* = 8.0, 1.7 Hz, 1H), 3.70 (s, 2H). **^{13}C NMR ($CDCl_3$, 100 MHz)**: δ 147.70, 130.47, 122.80, 121.06, 117.58, 113.50 ppm; MS (70 eV): m/z (%) =171.0.

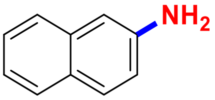

21

Naphthalen-2-amine (21): [13]

The reaction of 2-vinylnaphthalene **89** (46.2 mg, 0.3 mmol), $TMSN_3$ (86.4 mg, 0.75 mmol) in CCl_4 (1.0 mL) followed by the addition of $MeSO_3H$ (390 uL, 6.0 mmol), then the mixture was stirred under air at 80 °C for 4 h to afford 36.5 mg (85%) of **21**. Yellow solid. **^{1}H NMR ($CDCl_3$, 400 MHz)**:

δ 7.71 (dd, J = 14.0, 8.4 Hz, 2H), 7.63 (d, J = 8.2 Hz, 1H), 7.46–7.37 (m, 1H), 7.30–7.23 (m, 1H), 7.03 – 6.94 (m, 2H), 3.86 (s, 2H); **^{13}C NMR ($CDCl_3$, 100 MHz)**: δ 144.07, 134.89, 129.18, 127.94, 127.68, 126.31, 125.76, 122.44, 118.19, 108.55 ppm; MS (70 eV): m/z (%) =143.1

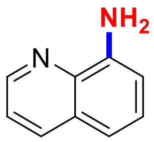

22

Quinolin-8-amine (22): [14]

The reaction of 8-vinylquinoline **90** (46.5 mg, 0.3 mmol), $TMSN_3$ (86.4 mg, 0.75 mmol) in CCl_4 (1.0 mL) followed by the addition of $MeSO_3H$ (390 uL, 6.0 mmol), then the mixture was stirred under air at 80 °C for 4 h to afford 19.0 mg (44%) of **22**. Yellow solid. **^{1}H NMR ($CDCl_3$, 400 MHz)**: δ 8.77 (dd, J = 4.2, 1.7 Hz, 1H), 8.06 (dd, J = 8.3, 1.7 Hz, 1H), 7.39 – 7.31 (m, 2H), 7.15 (dd, J = 8.2, 1.1 Hz, 1H), 6.93 (dd, J = 7.5, 1.2 Hz, 1H), 4.99 (s, 2H); **^{13}C NMR ($CDCl_3$, 100 MHz)**: δ 147.39, 143.93, 138.41, 135.94, 128.82, 127.33, 121.30, 115.99, 109.98 ppm; MS (70 eV): m/z (%) =144.1

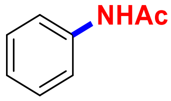

60

N-phenylacetamide (60): [6]

1) **From 23, Table 1 in Text**:

The reaction of styrene **23** (31.2 mg, 0.3 mmol), $TMSN_3$ (86.4 mg, 0.75 mmol) in CCl_4 (1.0 mL) followed by the addition of $MeSO_3H$ (390 uL, 6.0 mmol), then the mixture was stirred under air at 80 °C for 4 h. After cooling down to room temperature, the reaction mixture was quenched by 2 M NaOH (5 mL), extracted by EA (5 × 2 mL), the combined organic phase were washed with brine and dried over Na_2SO_4. Then the mixture were concentrated and AcCl (35.3 mg, 0.45 mmol), Et_3N (45.5 mg, 0.45 mmol) and DCM (5.0 mL) were added and stirred at rt for 6 h. The mixture were concentrated and purified by flash chromatography on a short silica (eluent: PE/EA = 10/1) to afford 28.4 mg (70%) of **60**. Yellow solid. **^{1}H NMR ($CDCl_3$, 400 MHz)**: δ 7.55 (s, 1H), 7.50 (d, J = 7.9 Hz, 2H), 7.30 (t, J = 7.8 Hz, 2H), 7.10 (t, J = 7.4 Hz, 1H), 2.16 (s, 3H); **^{13}C NMR ($CDCl_3$, 100 MHz)**: δ 168.48, 137.90, 128.93, 124.26, 119.92, 24.51 ppm; MS (70 eV): m/z (%) =135.1

2) **From 24, Table 1 in Text**:

The reaction of prop-1-en-2-ylbenzene **24** (35.4 mg, 0.3 mmol), $TMSN_3$ (86.4 mg, 0.75 mmol) in CCl_4 (1.0 mL) followed by the addition of $MeSO_3H$ (390 uL, 6.0 mmol), then the mixture was stirred under air at 80 °C for 4 h. After cooling down to room temperature, the reaction mixture was quenched by 2 M NaOH (5 mL), extracted by EA (5 × 2 mL), the combined organic phase were washed with brine and dried over Na_2SO_4. Then the mixture were concentrated and AcCl (35.3 mg, 0.45 mmol), Et_3N (45.5 mg, 0.45 mmol) and DCM (5.0 mL) were added and stirred at rt for 6 h. The mixture were concentrated and purified by flash chromatography on a short silica (eluent: PE/EA = 10/1) to afford 29.6 mg (73%) of **60**.

3) **From 25, Table 1 in Text**:

The reaction of ethene-1,1-diyldibenzene **25** (54.0 mg, 0.3 mmol), $TMSN_3$ (86.4 mg, 0.75 mmol) in CCl_4 (1.0 mL) followed by the addition of $MeSO_3H$ (390 uL, 6.0 mmol), then the mixture was stirred under air at 80 °C for 4 h. After cooling down to room temperature, the reaction mixture was quenched by 2 M NaOH (5 mL), extracted by EA (5 × 2 mL), the combined organic phase were washed with brine and dried over Na_2SO_4. Then the mixture were concentrated and AcCl (35.3 mg, 0.45 mmol), Et_3N (45.5 mg, 0.45 mmol) and DCM (5.0 mL) were added and stirred at rt for 6 h. The mixture were concentrated and purified by flash chromatography on a short silica (eluent: PE/EA = 10/1) to afford 20.2 mg (50%) of **60**.

4) **From 26, Table 1 in Text**:

The reaction of 2,3,4,5-tetrahydro-1,1'-biphenyl **26** (47.4 mg, 0.3 mmol), $TMSN_3$ (86.4 mg, 0.75 mmol) in CCl_4 (1.0 mL) followed by the addition of $MeSO_3H$ (390 uL, 6.0 mmol), then the mixture was stirred under air at 80 °C for 4 h. After cooling down to room temperature, the reaction mixture was quenched by 2 M NaOH (5 mL), extracted by EA (5 × 2 mL), the combined organic phase were washed with brine and dried over Na_2SO_4. Then the mixture were concentrated and AcCl (35.3 mg, 0.45 mmol), Et_3N (45.5 mg, 0.45 mmol) and DCM (5.0 mL) were added and stirred at rt for 6 h. The mixture were concentrated and purified by flash chromatography on a short silica (eluent: PE/EA = 10/1) to afford 27.6 mg (68%) of **60**.

5) **From 28, Table 1 in Text**:

The reaction of (E)-1,2-diphenylethene **28** (54.0 mg, 0.3 mmol), $TMSN_3$ (86.4 mg, 0.75 mmol) in CCl_4 (1.0 mL) followed by the addition of $MeSO_3H$ (390 uL, 6.0 mmol), then the mixture was

stirred under air at 80 °C for 4 h. After cooling down to room temperature, the reaction mixture was quenched by 2 M NaOH (5 mL), extracted by EA (5 × 2 mL), the combined organic phase were washed with brine and dried over Na_2SO_4. Then the mixture were concentrated and AcCl (35.3 mg, 0.45 mmol), Et_3N (45.5 mg, 0.45 mmol) and DCM (5.0 mL) were added and stirred at rt for 6 h. The mixture were concentrated and purified by flash chromatography on a short silica (eluent: PE/EA = 10/1) to afford 13.4 mg (33%) of **60**.

6) **From 29, Table 1 in Text**:

The reaction of allylbenzene **29** (35.4 mg, 0.3 mmol), $TMSN_3$ (86.4 mg, 0.75 mmol) in CCl_4 (1.0 mL) followed by the addition of $MeSO_3H$ (390 uL, 6.0 mmol), then the mixture was stirred under air at 80 °C for 4 h. After cooling down to room temperature, the reaction mixture was quenched by 2 M NaOH (5 mL), extracted by EA (5 × 2 mL), the combined organic phase were washed with brine and dried over Na_2SO_4. Then the mixture were concentrated and AcCl (35.3 mg, 0.45 mmol), Et_3N (45.5 mg, 0.45 mmol) and DCM (5.0 mL) were added and stirred at rt for 6 h. The mixture were concentrated and purified by flash chromatography on a short silica (eluent: PE/EA = 10/1) to afford 22.3 mg (55%) of **60**.

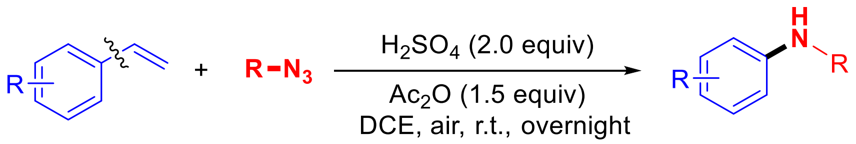

To a 20 mL vial equipped with a stir bar was added alkenes (0.2 mmol, 1.0 equiv), alkyl azide (0.4 mmol, 2.0 equiv), acetic anhydride (0.3 mmol, 1.5 equiv), DCE (2.0 mL) and the mixture was stirred at 25 °C. Then *conc.* H_2SO_4 (0.4 mmol, 2.0 equiv) was added to the mixture in 5 seconds and the mixture was stirred at 25 °C overnight. The reaction was quenched with 20% NaOH and was extracted with EA, isolated by column chromatography (silica gel, petroleum ether/AcOEt = 50/1), affording the desired arylamines**.**

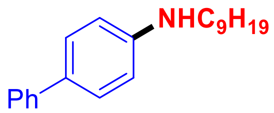

31

N-nonyl-[1,1'-biphenyl]-4-amine (31):

To a 10 mL Schlenk flask containing 4-vinyl-1,1'-biphenyl **48** (36.1 mg, 0.2 mmol), 1-azidononane

(67.7 mg, 0.4 mmol) and a magnetic stir bar, DCE (2 mL) and acetic anhydride (30.6 mg, 0.3 mmol) were added and the mixture was stirred at 25 °C. Conc. H_2SO_4 (39.3 mg, 0.4 mmol) were then added to the mixture in 5 seconds, and the mixture was stirred at 25 °C overnight. The reaction was quenched with 20% NaOH and the mixture was extracted with AcOEt, isolated by column chromatography (silica gel, petroleum ether/AcOEt = 50/1), affording 39 mg (66%) of **31** as colorless liquid. **^{1}H NMR (400 MHz, $CDCl_3$):** δ 7.53 (d, J = 7.6 Hz, 2H), 7.43 (d, J = 8.4 Hz, 2H), 7.37 (t, J = 7.6 Hz, 2H), 7.23 (t, J = 7.6 Hz, 1H), 6.66 (d, J = 8.0 Hz, 2H), 3.70 (brs, 1H), 3.13 (t, J = 7.2 Hz, 2H), 1.58-1.64 (m, 2H), 1.21-1.40 (m, 12H), 0.89 (t, J = 4.6 Hz, 3H).**^{13}C NMR (100 MHz, $CDCl_3$):** δ 147.9, 141.3, 129.9, 128.6, 127.9, 126.2, 125.9, 112.9, 44.0, 31.9, 29.5, 29.4, 29.2, 27.1, 22.6, 14.1. **HRMS *m/z* (ESI)** calcd for $C_{21}H_{30}N$ $[M+H]^+$ 296.2373, found: 296.2373.

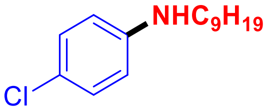

32

4-chloro-N-nonylaniline (32):

To a 10 mL Schlenk flask containing 1-chloro-4-vinylbenzene **49** (27.7 mg, 0.2 mmol), 1-azidononane (67.7 mg, 0.4 mmol) and a magnetic stir bar, DCE (2 mL) and acetic anhydride (30.6 mg, 0.3 mmol) were added and the mixture was stirred at 25 °C. Conc. H_2SO_4 (39.3 mg, 0.4 mmol) were then added to the mixture in 5 seconds, and the mixture was stirred at 25 °C overnight. The reaction was quenched with 20% NaOH and the mixture was extracted with AcOEt, isolated by column chromatography (silica gel, petroleum ether/AcOEt = 50/1), affording 25 mg (50%) of **32** as colorless liquid. **^{1}H NMR (400 MHz, $CDCl_3$):** δ 7.10 (d, J = 8.8 Hz, 2H), 6.51 (d, J = 8.8 Hz, 2H), 3.60 (brs, 1H), 3.06 (t, J = 7.0 Hz, 2H), 1.55-1.64 (m, 2H), 1.27-1.40 (m, 12H), 0.89 (t, J = 6.8 Hz, 3H). **^{13}C NMR (100 MHz, $CDCl_3$):** δ 147.1, 129.0, 121.5, 113.7, 44.1, 31.9, 29.5, 29.4, 29.2, 27.1, 22.6, 14.1. **HRMS *m/z* (ESI)** calcd for $C_{15}H_{25}ClN$ $[M+H]^+$ 254.1670, found: 254.1674.

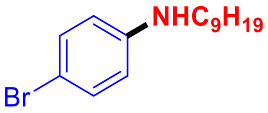

33

4-bromo-N-nonylaniline (33):

To a 10 mL Schlenk flask containing1-bromo-4-vinylbenzene **50** ((36.6 mg, 0.2 mmol), 1-azidononane (67.7 mg, 0.4 mmol) and a magnetic stir bar, DCE (2 mL) and acetic anhydride (30.6

mg, 0.3 mmol) were added and the mixture was stirred at 25 °C. Conc. H_2SO_4 (39.3 mg, 0.4 mmol) were then added to the mixture in 5 seconds, and the mixture was stirred at 25 °C overnight. The reaction was quenched with 20% NaOH and the mixture was extracted with AcOEt, isolated by column chromatography (silica gel, petroleum ether/AcOEt = 50/1), affording 30.1 mg (51%) of **33** as colorless liquid. **^{1}H NMR (400 MHz, CDCl$_3$):** δ 7.23 (d, *J* = 8.8 Hz, 2H), 6.46 (d, *J* = 9.2 Hz, 2H), 3.61 (brs, 1H), 3.05 (t, *J* = 7.0 Hz, 2H), 1.55-1.63 (m, 2H), 1.27-1.43 (m, 12H), 0.88 (t, *J* = 6.8 Hz, 3H). **^{13}C NMR (100 MHz, CDCl$_3$):** δ 147.5, 131.8, 114.2, 108.5, 44.0, 31.8, 29.5, 29.4, 29.3, 29.2, 27.1, 22.6, 14.1. **HRMS *m/z* (ESI)** calcd for $C_{15}H_{25}BrN$ $[M+H]^+$ 298.1165, found: 298.1169.

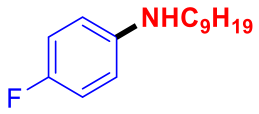

34

4-fluoro-N-nonylaniline (34):

To a 10 mL Schlenk flask containing 1-fluoro-4-vinylbenzene **51** (24.5 mg, 0.2 mmol), 1-azidononane (67.7 mg, 0.4 mmol) and a magnetic stir bar, DCE (2 mL) and acetic anhydride (30.6 mg, 0.3 mmol) were added and the mixture was stirred at 25 °C. Conc. H_2SO_4 (39.3 mg, 0.4 mmol) were then added to the mixture in 5 seconds, and the mixture was stirred at 25 °C overnight. The reaction was quenched with 20% NaOH and the mixture was extracted with AcOEt, isolated by column chromatography (silica gel, petroleum ether/AcOEt = 50/1), affording 35 mg (74%) of **34** as colorless liquid. **^{1}H NMR (400 MHz, CDCl$_3$):** δ 7.87 (d, *J* = 8.6 Hz, 2H), 6.50-6.54 (m, 2H), 3.46 (brs, 1H), 3.05 (t, *J* = 7.0 Hz, 2H), 1.56-1.64 (m, 2H), 1.27-1.40 (m, 12H), 0.88 (t, *J* = 6.6 Hz, 3H). **^{13}C NMR (100 MHz, CDCl$_3$):** δ 155.7 (d, *J* = 328), 144.9, 115.6 (d, *J* = 22.2), 113.4 (d, *J* = 7.4), 44.7, 31.8, 29.6, 29.5, 29.4, 29.2, 27.1, 22.6, 14.1. **HRMS *m/z* (ESI)** calcd for $C_{15}H_{25}FN$ $[M+H]^+$ 238.1966, found: 238.1967.

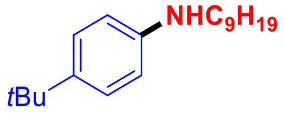

35

4-(tert-butyl)-N-nonylaniline (35):

To a 10 mL Schlenk flask containing 1-(tert-butyl)-4-vinylbenzene **52** (32.1 mg, 0.2 mmol), 1-azidononane (67.7 mg, 0.4 mmol) and a magnetic stir bar, DCE (2 mL) and acetic anhydride (30.6 mg, 0.3 mmol) were added and the mixture was stirred at 25 °C. Conc. H_2SO_4 (39.3 mg, 0.4 mmol)

were then added to the mixture in 5 seconds, and the mixture was stirred at 25 °C overnight. The reaction was quenched with 20% NaOH and the mixture was extracted with AcOEt, isolated by column chromatography (silica gel, petroleum ether/AcOEt = 50/1), affording 40.6 mg (74%) of **35** as colorless liquid. **^{1}H NMR (400 MHz, $CDCl_3$):** δ 7.20 (d, *J* = 8.4 Hz, 2H), 7.56 (d, *J* = 8.4 Hz, 2H), 3.48 (brs, 1H), 3.08 (t, *J* = 7.2 Hz, 2H), 1.57-1.62 (m, 2H), 1.27-1.39 (m, 21H), 0.88 (t, *J* = 6.6 Hz, 3H). **^{13}C NMR (100 MHz, $CDCl_3$):** δ 146.2, 139.8, 125.9, 112.4, 44.2, 33.8, 31.9, 31.5, 29.6, 29.5, 29.4, 29.3, 27.2, 22.7, 14.1. **HRMS *m/z* (ESI)** calcd for $C_{19}H_{34}N$ $[M+H]^+$ 276.2686, found: 276.2692.

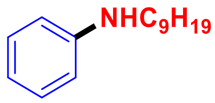

36

N-nonylaniline (36):

To a 10 mL Schlenk flask containing styrene **23** (20.8 mg, 0.2 mmol), 1-azidononane (67.7 mg, 0.4 mmol) and a magnetic stir bar, DCE (2 mL) and acetic anhydride (30.6 mg, 0.3 mmol) were added and the mixture was stirred at 25 °C. Conc. H_2SO_4 (39.3 mg, 0.4 mmol) were then added to the mixture in 5 seconds, and the mixture was stirred at 25 °C overnight. The reaction was quenched with 20% NaOH and the mixture was extracted with AcOEt, isolated by column chromatography (silica gel, petroleum ether/AcOEt = 50/1), affording 27.3 mg (63%) of **36** as colorless liquid.**^{1}H NMR (400 MHz, $CDCl_3$):** δ 7.17 (t, *J* = 7.6 Hz, 2H), 6.68 (d, *J* = 7.4 Hz, 1H), 6.60 (d, *J* = 8.0 Hz, 2H), 3.58 (brs, 1H), 3.01 (t, *J* = 7.2 Hz, 2H), 1.57-1.65 (m, 2H), 1.27-1.42 (m, 12H), 0.88 (t, *J* = 6.8 Hz, 3H). **^{13}C NMR (100 MHz, $CDCl_3$):** δ 148.5, 129.2, 117.0, 112.7, 44.0, 31.9, 29.6, 29.5, 29.4, 29.3, 27.2, 22.6, 14.1. **HRMS *m/z* (ESI)** calcd for $C_{15}H_{26}N$ $[M+H]^+$ 220.2060, found: 220.2062.

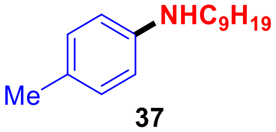

37

4-methyl-N-nonylaniline (37):

To a 10 mL Schlenk flask containing 1-methyl-4-vinylbenzene **53** (23.7 mg, 0.2 mmol), 1-azidononane (67.7 mg, 0.4 mmol) and a magnetic stir bar, DCE (2 mL) and acetic anhydride (30.6 mg, 0.3 mmol) were added and the mixture was stirred at 25 °C. Conc. H_2SO_4 (39.3 mg, 0.4 mmol) were then added to the mixture in 5 seconds, and the mixture was stirred at 25 °C overnight. The

reaction was quenched with 20% NaOH and the mixture was extracted with AcOEt, isolated by column chromatography (silica gel, petroleum ether/AcOEt = 50/1), affording 32.5 mg (70%) of **37** as colorless liquid. **^{1}H NMR (400 MHz, $CDCl_3$):** δ 6.98 (d, J = 8.4 Hz, 2H), 6.53 (d, J = 8.4 Hz, 2H), 3.45 (brs, 1H), 3.07 (t, J = 7.2 Hz, 2H), 2.23 (s, 3H), 1.55-1.63 (m, 2H), 1.27-1.40 (m, 12H), 0.88 (t, J = 6.8 Hz, 3H).**^{13}C NMR (100 MHz, $CDCl_3$):** δ 146.3, 129.7, 126.2, 112.9, 44.3, 31.8, 29.6, 29.5, 29.4, 29.3, 27.2, 22.6, 20.3, 14.1. **HRMS *m/z* (ESI)** calcd for $C_{16}H_{28}N$ $[M+H]^+$ 234.2216, found: 234.2218.

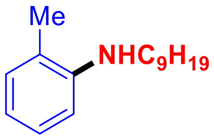

38

2-methyl-N-nonylaniline (38):

To a 10 mL Schlenk flask containing 1-methyl-2-vinylbenzene **54** (23.6 mg, 0.2 mmol), 1-azidononane (67.7 mg, 0.4 mmol) and a magnetic stir bar, DCE (2 mL) and acetic anhydride (30.6 mg, 0.3 mmol) were added and the mixture was stirred at 25 °C. Conc. H_2SO_4 (39.3 mg, 0.4 mmol) were then added to the mixture in 5 seconds, and the mixture was stirred at 25 °C overnight. The reaction was quenched with 20% NaOH and the mixture was extracted with AcOEt, isolated by column chromatography (silica gel, petroleum ether/AcOEt = 50/1), affording 37.2 mg (80%) of **38** as colorless liquid. **^{1}H NMR (400 MHz, $CDCl_3$):** δ 7.12 (d, J = 7.6 Hz, 1H), 7.04 (d, J = 7.2 Hz, 1H), 6.59-6.66 (m, 2H), 3.42 (brs, 1H), 3.14 (t, J = 7.2 Hz, 2H), 2.12 (s, 3H), 1.62-1.70 (m, 2H), 1.27-1.44 (m, 12H), 0.89 (t, J = 6.6 Hz, 3H). **^{3}C NMR (100 MHz, $CDCl_3$):** δ 146.4, 130.0, 127.1, 121.6, 116.6, 109.6, 43.9, 31.9, 29.6, 29.5, 29.4, 29.3, 27.2, 22.6, 17.4, 14.1. **HRMS *m/z* (ESI)** calcd for $C_{16}H_{28}N$ $[M+H]^+$ 234.2216, found: 234.2219.

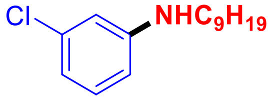

39

3-chloro-N-nonylaniline (39):

To a 10 mL Schlenk flask containing 1-chloro-3-vinylbenzene **86** (27.6 mg, 0.2 mmol), 1-azidononane (67.7 mg, 0.4 mmol) and a magnetic stir bar, DCE (2 mL) and acetic anhydride (30.6 mg, 0.3 mmol) were added and the mixture was stirred at 25 °C. Conc. H_2SO_4 (39.3 mg, 0.4 mmol)

were then added to the mixture in 5 seconds, and the mixture was stirred at 25 °C overnight. The reaction was quenched with 20% NaOH and the mixture was extracted with AcOEt, isolated by column chromatography (silica gel, petroleum ether/AcOEt = 50/1), affording 20.6 mg (41%) of **39** as colorless liquid. **^{1}H NMR (400 MHz, CDCl$_3$):** δ 7.05 (d, *J* = 8.0 Hz, 1H), 6.63 (dd, *J* = 8.0 Hz, 0.8 Hz, 1H), 6.59 (s, 1H), 6.45 (dd, *J* = 8.4 Hz, 1.6Hz, 1H), 3.71 (brs, 1H), 3.07 (t, *J* = 7.2 Hz, 2H), 1.56-1.64 (m, 2H), 1.27-1.38 (m, 12H), 0.88 (t, *J* = 6.6 Hz, 3H). **^{13}C NMR (100 MHz, CDCl$_3$):** δ 149.6, 135.0, 130.1, 116.8, 112.1, 111.0, 43.8, 31.8, 29.5, 29.4, 29.3, 29.2, 27.1, 22.6, 14.1. **HRMS *m/z* (ESI)** calcd for $C_{15}H_{25}ClN$ $[M+H]^+$ 254.1670, found: 254.1677.

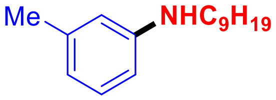

40

3-methyl-N-nonylaniline (40):

To a 10 mL Schlenk flask containing 1-methyl-3-vinylbenzene **87** (23.6 mg, 0.2 mmol), 1-azidononane **2a** (67.7 mg, 0.4 mmol) and a magnetic stir bar, DCE (2 mL) and acetic anhydride (30.6 mg, 0.3 mmol) were added and the mixture was stirred at 25 °C. Conc. H_2SO_4 (39.3 mg, 0.4 mmol) were then added to the mixture in 5 seconds, and the mixture was stirred at 25 °C overnight. The reaction was quenched with 20% NaOH and the mixture was extracted with AcOEt, isolated by column chromatography (silica gel, petroleum ether/AcOEt = 50/1), affording 30.5 mg (66%) of **40** as colorless liquid. **^{1}H NMR (400 MHz, CDCl$_3$):** δ 7.05 (t, *J* = 8.0 Hz, 1H), 6.51 (d, *J* = 7.2 Hz, 1H), 6.42 (s, 1H), 6.41 (d, *J* = 7.2 Hz, 1H), 3.52 (brs, 1H), 3.08 (t, *J* = 7.2 Hz, 2H), 2.27 (s, 3H), 1.56-1.62 (m, 2H), 1.27-1.40 (m, 12H), 0.88 (t, *J* = 6.8 Hz, 3H). **^{13}C NMR (100 MHz, CDCl$_3$):** δ 148.6, 138.9, 129.1, 118.0, 113.4, 109.8, 44.0, 31.9, 29.6, 29.5, 29.4, 29.2, 27.2, 22.6, 21.6, 14.1. **HRMS *m/z* (ESI)** calcd for $C_{16}H_{28}N$ $[M+H]^+$ 234.2216, found: 234.2222.

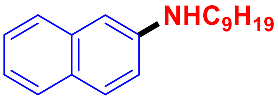

41

N-nonylnaphthalen-2-amine (41):

To a 10 mL Schlenk flask containing 2-vinylnaphthalene **89** (30.8 mg, 0.2 mmol), 1-azidononane (67.7 mg, 0.4 mmol) and a magnetic stir bar, DCE (2 mL) and acetic anhydride (30.6 mg, 0.3 mmol) were added and the mixture was stirred at 25 °C. Conc. H_2SO_4 (39.3 mg, 0.4 mmol) were then added

to the mixture in 5 seconds, and the mixture was stirred at 25 °C overnight. The reaction was quenched with 20% NaOH and the mixture was extracted with AcOEt, isolated by column chromatography (silica gel, petroleum ether/AcOEt = 50/1), affording 26.7 mg (50%) of **41** as colorless liquid. **^{1}H NMR (400 MHz, $CDCl_3$):** δ 7.65 (d, J = 8.0 Hz, 1H), 7.60 (d, J = 8.8 Hz, 2H), 7.34 (t, J = 7.6 Hz, 1H), 7.17 (t, J = 7.4 Hz, 1H), 6.86 (d, J = 8.8 Hz, 1H), 6.79 (s, 1H), 3.86 (brs, 1H), 3.19 (t, J = 7.2 Hz, 2H), 1.63-1.69 (m, 2H), 1.28-1.45 (m, 12H), 0.89 (t, J = 6.8 Hz, 3H). **^{13}C NMR (100 MHz, $CDCl_3$):** δ 146.1, 135.3, 128.8, 127.6, 127.4, 126.2, 125.9, 121.8, 118.0, 104.3, 44.1, 31.9, 29.6, 29.5, 29.4, 29.3, 27.2, 22.7, 14.1. **HRMS *m/z* (ESI)** calcd for $C_{19}H_{28}N$ $[M+H]^+$ 270.2216, found: 270.2219.

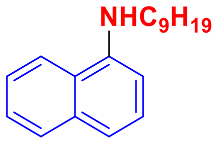

42

N-nonylnaphthalen-1-amine (42):

To a 10 mL Schlenk flask containing 1-vinylnaphthalene **91** (30.8 mg, 0.2 mmol), 1-azidononane (67.7 mg, 0.4 mmol) and a magnetic stir bar, DCE (2 mL) and acetic anhydride (30.6 mg, 0.3 mmol) were added and the mixture was stirred at 25 °C. Conc. H_2SO_4 (39.3 mg, 0.4 mmol) were then added to the mixture in 5 seconds, and the mixture was stirred at 25 °C overnight. The reaction was quenched with 20% NaOH and the mixture was extracted with AcOEt, isolated by column chromatography (silica gel, petroleum ether/AcOEt = 50/1), affording 29.7 mg (56%) of **42** as colorless liquid. **^{1}H NMR (400 MHz, $CDCl_3$):** δ 7.76-7.80 (m, 2H), 7.39-7.45 (m, 2H), 7.34 (t, J = 8.0 Hz, 1H), 7.20 (t, J = 8.0 Hz, 1H), 6.60 (d, J = 7.6 Hz, 1H), 4.28 (brs, 1H), 3.25 (t, J = 7.2 Hz, 2H), 1.72-1.80 (m, 2H), 1.29-1.52 (m, 12H), 0.89 (t, J = 7.0 Hz, 3H). **^{13}C NMR (100 MHz, $CDCl_3$):** δ 143.7, 134.3, 128.6, 126.7, 125.6, 124.5, 123.3, 119.7, 117.0, 104.1, 44.2, 31.9, 29.6, 29.5, 29.4, 29.3, 27.4, 22.7, 14.1. **HRMS *m/z* (ESI)** calcd for $C_{19}H_{28}N$ $[M+H]^+$ 270.2216, found: 270.2220.

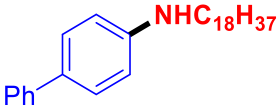

43

N-octadecyl-[1,1'-biphenyl]-4-amine (43):

To a 10 mL Schlenk flask containing 4-vinyl-1,1'-biphenyl **48** (36.1 mg, 0.2 mmol), 1-

azidooctadecane (118.1 mg, 0.4 mmol) and a magnetic stir bar, DCE (2 mL) and acetic anhydride (30.6 mg, 0.3 mmol) were added and the mixture was stirred at 25 °C. Conc. H_2SO_4 (39.3 mg, 0.4 mmol) were then added to the mixture in 5 seconds, and the mixture was stirred at 25 °C overnight. The reaction was quenched with 20% NaOH and the mixture was extracted with AcOEt, isolated by column chromatography (silica gel, petroleum ether/AcOEt = 50/1), affording 58.2 mg (69%) of **43** as colorless liquid. **^{1}H NMR (400 MHz, $CDCl_3$):** δ 7.53 (d, J = 7.6 Hz, 2H), 7.43 (d, J = 8.0 Hz, 2H), 7.38 (t, J = 7.4 Hz, 2H), 7.24 (t, J = 7.2 Hz, 1H), 6.66 (d, J = 7.6 Hz, 2H), 3.69 (brs, 1H), 3.14 (t, J = 7.0 Hz, 2H), 1.59-1.65 (m, 2H), 1.25-1.41 (m, 30H), 0.88 (t, J = 6.6 Hz, 3H). **^{13}C NMR (100 MHz, $CDCl_3$):** δ 147.9, 141.3, 129.9, 128.6, 127.9, 126.2, 125.9, 112.9, 44.0, 31.9, 29.7, 29.65, 29.61, 29.55, 29.5, 29.4, 29.3, 29.1, 27.2, 22.7, 14.1. **HRMS *m/z* (ESI)** calcd for $C_{30}H_{48}N$ $[M+H]^+$ 422.3781, found: 422.3790.

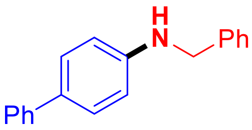

44

N-benzyl-[1,1'-biphenyl]-4-amine (44):

To a 10 mL Schlenk flask containing 4-vinyl-1,1'-biphenyl **48** (36.1 mg, 0.2 mmol), (azidomethyl)benzene (53.3 mg, 0.4 mmol) and a magnetic stir bar, DCE (2 mL) and acetic anhydride (30.6 mg, 0.3 mmol) were added and the mixture was stirred at 25 °C. Conc. H_2SO_4 (39.3 mg, 0.4 mmol) were then added to the mixture in 5 seconds, and the mixture was stirred at 25 °C overnight. The reaction was quenched with 20% NaOH and the mixture was extracted with AcOEt, isolated by column chromatography (silica gel, petroleum ether/AcOEt = 20/1), affording 29 mg (56%) of **44** as colorless liquid. **^{1}H NMR (400 MHz, $CDCl_3$):** δ 7.52 (d, J = 7.6 Hz, 2H), 7.43 (d, J = 8.8 Hz, 2H), 7.32-7.39 (m, 6H), 7.21-7.29 (m, 2H), 6.69 (t, J = 8.4 Hz, 2H), 4.35 (s, 2H), 4.11 (brs, 1H). **^{13}C NMR (100 MHz, $CDCl_3$):** δ 147.5, 141.2, 139.3, 130.4, 128.7, 128.6, 127.9, 127.4, 127.3, 126.3, 126.0, 113.1, 48.3. **HRMS *m/z* (ESI)** calcd for $C_{19}H_{18}N$ $[M+H]^+$ 260.1434, found: 260.1436.

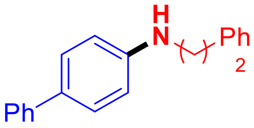

45

N-phenethyl-[1,1'-biphenyl]-4-amine (45):

To a 10 mL Schlenk flask containing 4-vinyl-1,1'-biphenyl **48** (36.1 mg, 0.2 mmol), (2-azidoethyl)benzene (58.9 mg, 0.4 mmol) and a magnetic stir bar, DCE (2 mL) and acetic anhydride (30.6 mg, 0.3 mmol) were added and the mixture was stirred at 25 °C. Conc. H_2SO_4 (39.3 mg, 0.4 mmol) were then added to the mixture in 5 seconds, and the mixture was stirred at 25 °C overnight. The reaction was quenched with 20% NaOH and the mixture was extracted with AcOEt, isolated by column chromatography (silica gel, petroleum ether/AcOEt = 50/1), affording 32 mg (59%) of **45** as colorless liquid. **^{1}H NMR (400 MHz, CDCl$_3$):** δ 7.53 (d, J = 7.2 Hz, 2H), 7.44 (d, J = 8.4 Hz, 2H), 7.38 (t, J = 7.6 Hz, 2H), 7.32 (t, J = 7.4 Hz, 2H), 7.21-7.26 (m, 4H), 6.67 (d, J = 8.4 Hz, 2H), 3.75 (brs, 1H), 3.43 (t, J = 7.0 Hz, 2H), 2.93 (t, J = 7.0 Hz, 2H). **^{13}C NMR (100 MHz, CDCl$_3$):** δ 147.4, 141.2, 139.2, 130.3, 128.8, 128.6, 127.9, 126.4, 126.2, 126.0, 113.2, 45.0, 35.5. **HRMS *m/z* (ESI)** calcd for $C_{20}H_{20}N$ $[M+H]^+$ 274.1590, found: 274.1593.

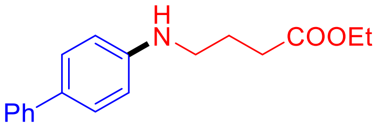

46

ethyl 4-([1,1'-biphenyl]-4-ylamino)butanoate (46):

To a 10 mL Schlenk flask containing 4-vinyl-1,1'-biphenyl **48** (36.1 mg, 0.2 mmol), ethyl 4-azidobutanoate **2r** (62.9 mg, 0.4 mmol) and a magnetic stir bar, DCE (2 mL) and acetic anhydride (30.6 mg, 0.3 mmol) were added and the mixture was stirred at 25 °C. Conc. H_2SO_4 (39.3 mg, 0.4 mmol) were then added to the mixture in 5 seconds, and the mixture was stirred at 25 °C overnight. The reaction was quenched with saturated $NaHCO_3$ and the mixture was extracted with AcOEt, isolated by column chromatography (silica gel, petroleum ether/AcOEt = 5/1), affording 22.1 mg (39%) of **46** as colorless liquid. **^{1}H NMR (400 MHz, CDCl$_3$):** δ 7.53 (d, J = 7.6 Hz, 2H), 7.43 (d, J = 8.8 Hz, 2H), 7.38 (t, J = 7.6 Hz, 2H), 7.23 (t, J = 7.4 Hz, 1H), 6.67 (d, J = 8.4 Hz, 2H), 4.14 (q, J = 7.2 Hz, 2H), 3.85 (brs, 1H), 3.22 (t, J = 6.8 Hz, 2H), 2.44 (t, J = 7.2 Hz, 2H), 1.93-2.01 (m, 2H), 1.26 (t, J = 7.0 Hz, 3H). **^{13}C NMR (100 MHz, CDCl$_3$):** δ 173.4, 147.6, 141.2, 130.2, 128.6, 127.9, 126.3, 126.0, 112.9, 60.5, 43.3, 31.9, 24.6, 14.2. **HRMS *m/z* (ESI)** calcd for $C_{18}H_{22}NO_2$ $[M+H]^+$ 284.1645, found: 284.1654.

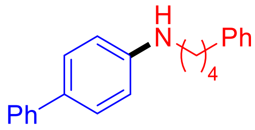

47

N-(4-phenylbutyl)-[1,1'-biphenyl]-4-amine (47):

To a 10 mL Schlenk flask containing 4-vinyl-1,1'-biphenyl **48** (36.1 mg, 0.2 mmol), (4-azidobutyl)benzene (70.0 mg, 0.4 mmol) and a magnetic stir bar, DCE (2 mL) and acetic anhydride (30.6 mg, 0.3 mmol) were added and the mixture was stirred at 25 °C. Conc. H_2SO_4 (39.3 mg, 0.4 mmol) were then added to the mixture in 5 seconds, and the mixture was stirred at 25 °C overnight. The reaction was quenched with 20% NaOH and the mixture was extracted with AcOEt, isolated by column chromatography (silica gel, petroleum ether/AcOEt = 50/1), affording 41 mg (68%) of **47** as colorless liquid. **^{1}H NMR (400 MHz, $CDCl_3$):** δ 7.53 (d, J = 7.2 Hz, 2H), 7.42 (d, J = 8.8 Hz, 2H), 7.37 (t, J = 7.8 Hz, 2H), 7.17-7.30 (m, 6H), 6.64 (d, J = 8.8 Hz, 2H), 3.65 (brs, 1H), 3.15 (t, J = 6.8 Hz, 2H), 2.66 (t, J = 7.4 Hz, 2H), 1.63-1.77 (m, 4H). **^{13}C NMR (100 MHz, $CDCl_3$):** δ 147.8, 142.1, 141.3, 130.0, 128.6, 128.4, 128.3, 127.9, 126.2, 126.0, 125.8, 112.9, 43.8, 35.6, 29.1, 28.9. **HRMS *m/z* (ESI)** calcd for $C_{22}H_{24}N$ $[M+H]^+$ 302.1903, found: 302.1901.

E. General procedure for C(Ar)-C(Alkenyl) single bond hydroxylation of styrenes.

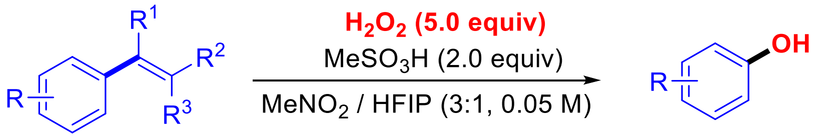

To a 20 mL vial equipped with a stir bar was added alkenes (0.3 mmol, 1.0 euiv), $MeNO_2$ (4.5 mL)/HFIP (1.5 mL), followed by the addition of 30% aqueous hydrogen peroxide solution (170 mg, 1.5 mmol) and $MeSO_3H$ (39 uL, 0.6 mmol) in order. The reaction was heated under Ar at 60 °C for 12 h. After cooling down to room temperature, the reaction mixture was quenched by *sat*. $NaHCO_3$ (5 mL), extracted by EA (5 × 2 mL), the combined organic phase were washed with brine and dried over Na_2SO_4. Then the mixture were concentrated and purified by flash chromatography on a short silica gel (eluent: PE/EA = 10/1) to afford the desired phenols.

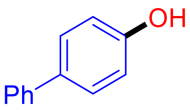

72

[1,1'-biphenyl]-4-ol (72):[15]

The reaction of 4-vinyl-1,1'-biphenyl **48** (54.1 mg, 0.3 mmol) in $MeNO_2$ (4.5 mL)/HFIP (1.5 mL) followed by the addition of 30% aqueous hydrogen peroxide solution (170 mg, 1.5 mmol) and $MeSO_3H$ (39 uL, 0.6 mmol), then the mixture was stirred under Ar at 60 °C for 12 h to afford 35.7 mg (70%) of **72**. yellow solid. **^{1}H NMR (DMSO-d$_6$, 400 MHz)**: δ 9.56 (s, 1H), 7.59 – 7.55 (m, 2H), 7.51 – 7.46 (m, 2H), 7.42-7.37 (m, 2H), 7.29 – 7.24 (m, 1H), 6.90 – 6.85 (m, 2H); **^{13}C NMR (DMSO-d$_6$, 100 MHz)**: δ 157.14, 140.23, 130.94, 128.78, 127.73, 126.33, 125.95, 115.74 ppm; MS (70 eV): m/z (%) =170.1.

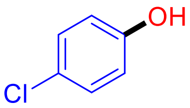

92

4-chlorophenol (92):[15]

The reaction of 1-chloro-4-vinylbenzene **49** (41.4 mg, 0.3 mmol) in $MeNO_2$ (4.5 mL)/HFIP (1.5 mL) followed by the addition of 30% aqueous hydrogen peroxide solution (170 mg, 1.5 mmol) and $MeSO_3H$ (39 uL, 0.6 mmol), then the mixture was stirred under Ar at 60 °C for 12 h to afford 19.2

mg (50%) of **92**. Yellow oil. **^{1}H NMR ($CDCl_3$, 400 MHz)**: δ 7.23 – 7.17 (m, 2H), 6.80 – 6.74 (m, 2H), 4.82 (s, 1H); **^{13}C NMR ($CDCl_3$, 100 MHz)**: δ 154.04, 129.52, 125.72, 116.64 ppm; MS (70 eV): m/z (%) =128.2.

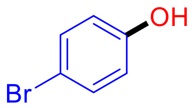

93

4-bromophenol (93): [15]

The reaction of 1-bromo-4-vinylbenzene **50** (54.5 mg, 0.3 mmol) in $MeNO_2$ (4.5 mL)/HFIP (1.5 mL) followed by the addition of 30% aqueous hydrogen peroxide solution (170 mg, 1.5 mmol) and $MeSO_3H$ (39 uL, 0.6 mmol), then the mixture was stirred under Ar at 60 °C for 12 h to afford 26.8 mg (52%) of **93**. yellow solid. **^{1}H NMR ($CDCl_3$, 400 MHz)**: δ 7.37 – 7.31 (m, 2H), 6.76 – 6.69 (m, 2H), 4.85 (s, 1H); **^{13}C NMR ($CDCl_3$, 100 MHz)**: δ 154.52, 132.47, 117.17, 112.93 ppm; MS (70 eV): m/z (%) =172.0.

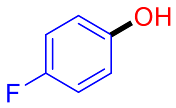

94

4-Fluorophenol (94): [15]

The reaction of 1-fluoro-4-vinylbenzene **51** (36.6 mg, 0.3 mmol) in $MeNO_2$ (4.5 mL)/HFIP (1.5 mL) followed by the addition of 30% aqueous hydrogen peroxide solution (170 mg, 1.5 mmol) and $MeSO_3H$ (39 uL, 0.6 mmol), then the mixture was stirred under Ar at 60 °C for 12 h to afford 21.8 mg (65%) of **94**. yellow oil. **^{1}H NMR ($CDCl_3$, 400 MHz)**: δ 6.98 – 6.87 (m, 2H), 6.84 – 6.68 (m, 2H), 4.92 (s, 1H); **^{13}C NMR ($CDCl_3$, 100 MHz)**: δ 157.30 (d, J = 237 Hz), 151.40 (d, J = 2.0 Hz), 116.22 (d, J = 8.0 Hz), 115.99 (d, J = 23 Hz). **^{19}F NMR (376 MHz, $CDCl_3$)** δ -124.16 ppm; MS (70 eV): m/z (%) =112.1.

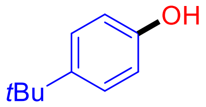

95

4-(tert-butyl)phenol (95): [16]

The reaction of 1-(tert-butyl)-4-vinylbenzene **52** (48.0 mg, 0.3 mmol) in $MeNO_2$ (4.5 mL)/HFIP (1.5 mL) followed by the addition of 30% aqueous hydrogen peroxide solution (170 mg, 1.5 mmol)

and $MeSO_3H$ (39 uL, 0.6 mmol), then the mixture was stirred under Ar at 60 °C for 12 h to afford 29.7 mg (66%) of **95**. yellow solid. **^{1}H NMR ($CDCl_3$, 400 MHz)**: δ 7.32 – 7.26 (m, 2H), 6.83 – 6.77 (m, 2H), 4.73 (s, 1H), 1.33 (s, 9H); **^{13}C NMR ($CDCl_3$, 100 MHz)**: δ 153.07, 143.54, 126.42, 114.72, 34.05, 31.51 ppm; MS (70 eV): m/z (%) =150.1.

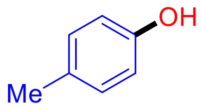

96

***P*-cresol (96):** [16]

The reaction of 1-methyl-4-vinylbenzene **53** (35.4 mg, 0.3 mmol) in $MeNO_2$ (4.5 mL)/HFIP (1.5 mL) followed by the addition of 30% aqueous hydrogen peroxide solution (170 mg, 1.5 mmol) and $MeSO_3H$ (39 uL, 0.6 mmol), then the mixture was stirred under Ar at 60 °C for 12 h to afford 16.2 mg (50%) of **96**. yellow oil. **^{1}H NMR ($CDCl_3$, 400 MHz)**: δ 7.08 (d, J = 8.3 Hz, 2H), 6.80 (d, J = 8.5 Hz, 2H), 5.86 (s, 1H), 2.33 (s, 3H); **^{13}C NMR ($CDCl_3$, 100 MHz)**: δ 152.85, 130.05, 115.15, 20.36 ppm; MS (70 eV): m/z (%) =108.1.

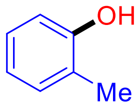

97

***O*-cresol (97):** [15]

The reaction of 1-methyl-2-vinylbenzene **54** (35.4 mg, 0.3 mmol) in $MeNO_2$ (4.5 mL)/HFIP (1.5 mL) followed by the addition of 30% aqueous hydrogen peroxide solution (170 mg, 1.5 mmol) and $MeSO_3H$ (39 uL, 0.6 mmol), then the mixture was stirred under Ar at 60 °C for 12 h to afford 9.7 mg (30%) of **97**. yellow oil. **^{1}H NMR ($CDCl_3$, 400 MHz)**: δ 7.22 (d, J = 7.5 Hz, 1H), 7.17 (t, J = 7.7 Hz, 1H), 6.96 (t, J = 7.3 Hz, 1H), 6.85 (d, J = 8.0 Hz, 1H), 5.41 (s, 1H), 2.35 (s, 3H); **^{13}C NMR ($CDCl_3$, 100 MHz)**: δ 153.51, 131.02, 127.03, 123.92, 120.79, 114.94, 15.64 ppm; MS (70 eV): m/z (%) =108.1.

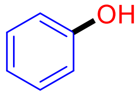

98

Phenol (98): [17]

1) **From 23, Table 3 in Text**:

The reaction of styrene **23** (31.2 mg, 0.3 mmol) in $MeNO_2$ (4.5 mL)/HFIP (1.5 mL) followed by the addition of 30% aqueous hydrogen peroxide solution (170 mg, 1.5 mmol) and $MeSO_3H$ (39 uL, 0.6 mmol), then the mixture was stirred under Ar at 60 °C for 12 h. After cooling down to room temperature, the reaction mixture was quenched by *sat*. $NaHCO_3$ (5 mL), extracted by EA (5 × 2 mL), the combined organic phase were concentrated. Owing to the volatilization and low-boiling point of the product, the yield of **98** was determined by HNMR using 1,1,2,2-Tetrachloroethane as internal standard. NMR yield of **98** in this reaction is 45%. Yellow solid. **^{1}H NMR ($CDCl_3$, 400 MHz)**: δ 7.33 – 7.25 (m, 2H), 6.99 (t, *J* = 7.4 Hz, 1H), 6.94 – 6.86 (m, 2H), 5.58 (s, 1H); **^{13}C NMR ($CDCl_3$, 100 MHz)**: δ 155.27, 129.67, 120.82, 115.32 ppm; MS (70 eV): m/z (%) =94.1.

2) **From 24, Table 3 in Text**:

The reaction of prop-1-en-2-ylbenzene **24** (35.4 mg, 0.3 mmol) in $MeNO_2$ (4.5 mL)/HFIP (1.5 mL) followed by the addition of 30% aqueous hydrogen peroxide solution (170 mg, 1.5 mmol) and $MeSO_3H$ (39 uL, 0.6 mmol), then the mixture was stirred under Ar at 60 °C for 12 h. After cooling down to room temperature, the reaction mixture was quenched by *sat*. $NaHCO_3$ (5 mL), extracted by EA (5 × 2 mL), the combined organic phase were concentrated. Owing to the volatilization and low-boiling point of the product, the yield of **98** was determined by HNMR using 1,1,2,2-Tetrachloroethane as internal standard. NMR yield of **98** in this reaction is 32%.

3) **From 25, Table 3 in Text**:

The reaction of ethene-1,1-diyldibenzene **25** (54.0 mg, 0.3 mmol) in $MeNO_2$ (4.5 mL)/HFIP (1.5 mL) followed by the addition of 30% aqueous hydrogen peroxide solution (170 mg, 1.5 mmol) and $MeSO_3H$ (39 uL, 0.6 mmol), then the mixture was stirred under Ar at 60 °C for 12 h. After cooling down to room temperature, the reaction mixture was quenched by *sat*. $NaHCO_3$ (5 mL), extracted by EA (5 × 2 mL), the combined organic phase were concentrated. Owing to the volatilization and low-boiling point of the product, the yield of **98** was determined by HNMR using 1,1,2,2-Tetrachloroethane as internal standard. NMR yield of **98** in this reaction is 62%.

4) **From 26, Table 3 in Text**:

The reaction of 2,3,4,5-tetrahydro-1,1'-biphenyl **26** (47.4 mg, 0.3 mmol) in $MeNO_2$ (4.5 mL)/HFIP (1.5 mL) followed by the addition of 30% aqueous hydrogen peroxide solution (170 mg, 1.5 mmol) and $MeSO_3H$ (39 uL, 0.6 mmol), then the mixture was stirred under Ar at 60 °C for 12 h. After

cooling down to room temperature, the reaction mixture was quenched by *sat*. $NaHCO_3$ (5 mL), extracted by EA (5 × 2 mL), the combined organic phase were concentrated. Owing to the volatilization and low-boiling point of the product, the yield of **98** was determined by HNMR using 1,1,2,2-Tetrachloroethane as internal standard. NMR yield of **98** in this reaction is 40%.

5) **From 28, Table 3 in Text**:

The reaction of (E)-1,2-diphenylethene **28** (54.0 mg, 0.3 mmol) in $MeNO_2$ (4.5 mL)/HFIP (1.5 mL) followed by the addition of 30% aqueous hydrogen peroxide solution (170 mg, 1.5 mmol) and $MeSO_3H$ (39 uL, 0.6 mmol), then the mixture was stirred under Ar at 60 ºC for 12 h. After cooling down to room temperature, the reaction mixture was quenched by *sat*. $NaHCO_3$ (5 mL), extracted by EA (5 × 2 mL), the combined organic phase were concentrated. Owing to the volatilization and low-boiling point of the product, the yield of **98** was determined by HNMR using 1,1,2,2-Tetrachloroethane as internal standard. NMR yield of **98** in this reaction is 60%.

6) **From 55, Table 3 in Text**:

The reaction of 1',2',3',6'-tetrahydro-1,1':4',1''-terphenyl **55** (70.2 mg, 0.3 mmol) in $MeNO_2$ (4.5 mL)/HFIP (1.5 mL) followed by the addition of 30% aqueous hydrogen peroxide solution (170 mg, 1.5 mmol) and $MeSO_3H$ (39 uL, 0.6 mmol), then the mixture was stirred under Ar at 60 ºC for 12 h. After cooling down to room temperature, the reaction mixture was quenched by *sat*. $NaHCO_3$ (5 mL), extracted by EA (5 × 2 mL), the combined organic phase were concentrated. Owing to the volatilization and low-boiling point of the product, the yield of **98** was determined by HNMR using 1,1,2,2-Tetrachloroethane as internal standard. NMR yield of **98** in this reaction is 60%.

7) **From 56, Table 3 in Text**:

The reaction of ethene-1,1,2-triyltribenzene **56** (76.8 mg, 0.3 mmol) in $MeNO_2$ (4.5 mL)/HFIP (1.5 mL) followed by the addition of 30% aqueous hydrogen peroxide solution (170 mg, 1.5 mmol) and $MeSO_3H$ (39 uL, 0.6 mmol), then the mixture was stirred under Ar at 60 ºC for 12 h. After cooling down to room temperature, the reaction mixture was quenched by *sat*. $NaHCO_3$ (5 mL), extracted by EA (5 × 2 mL), the combined organic phase were concentrated. Owing to the volatilization and low-boiling point of the product, the yield of **98** was determined by HNMR using 1,1,2,2-Tetrachloroethane as internal standard. NMR yield of **98** in this reaction is 40%.

8) **From 57, Table 3 in Text**:

The reaction of (*E*)-4-phenylbut-3-en-2-one **57** (43.8 mg, 0.3 mmol) in $MeNO_2$ (4.5 mL)/HFIP (1.5 mL) followed by the addition of 30% aqueous hydrogen peroxide solution (170 mg, 1.5 mmol) and $MeSO_3H$ (39 uL, 0.6 mmol), then the mixture was stirred under Ar at 60 ºC for 12 h. After cooling down to room temperature, the reaction mixture was quenched by *sat*. $NaHCO_3$ (5 mL), extracted by EA (5 × 2 mL), the combined organic phase were concentrated. Owing to the volatilization and low-boiling point of the product, the yield of **98** was determined by HNMR using 1,1,2,2-Tetrachloroethane as internal standard. NMR yield of **98** in this reaction is 20%.

9) **From 58, Table 3 in Text**:

The reaction of (E)-chalcone **58** (62.4 mg, 0.3 mmol) in $MeNO_2$ (4.5 mL)/HFIP (1.5 mL) followed by the addition of 30% aqueous hydrogen peroxide solution (170 mg, 1.5 mmol) and $MeSO_3H$ (39 uL, 0.6 mmol), then the mixture was stirred under Ar at 60 ºC for 12 h. After cooling down to room temperature, the reaction mixture was quenched by *sat*. $NaHCO_3$ (5 mL), extracted by EA (5 × 2 mL), the combined organic phase were concentrated. Owing to the volatilization and low-boiling point of the product, the yield of **98** was determined by HNMR using 1,1,2,2-Tetrachloroethane as internal standard. NMR yield of **98** in this reaction is 30%.

10) **From 59, Table 3 in Text**:

The reaction of (E)-3-phenylprop-2-en-1-ol **59** (40.2 mg, 0.3 mmol) in $MeNO_2$ (4.5 mL)/HFIP (1.5 mL) followed by the addition of 30% aqueous hydrogen peroxide solution (170 mg, 1.5 mmol) and $MeSO_3H$ (39 uL, 0.6 mmol), then the mixture was stirred under Ar at 60 ºC for 12 h. After cooling down to room temperature, the reaction mixture was quenched by *sat*. $NaHCO_3$ (5 mL), extracted by EA (5 × 2 mL), the combined organic phase were concentrated. Owing to the volatilization and low-boiling point of the product, the yield of **98** was determined by HNMR using 1,1,2,2-Tetrachloroethane as internal standard. NMR yield of **98** in this reaction is 30%.

F. Synthetic applications of the de-alkenylating C-C bond functionalization

1) Gram-scale reaction with O_2 as the oxidant in one-pot reaction

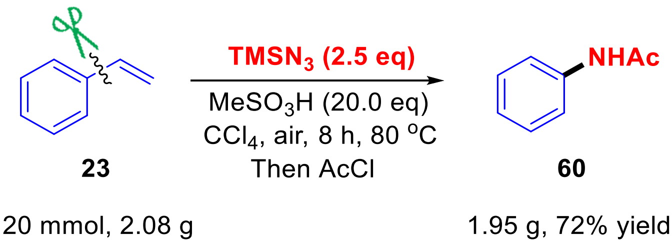

The reaction of styrene **23** (2.08 g, 20 mmol), $TMSN_3$ (5.76 g, 50 mmol) in CCl_4 (50 mL) followed by the addition of $MeSO_3H$ (26 mL, 400 mmol), then the mixture was stirred under air at 80 °C for 8 h. After cooling down to room temperature, the reaction mixture was quenched by 2 M NaOH (5 mL), extracted by EA (5 × 2 mL), the combined organic phase were washed with brine and dried over Na_2SO_4. Then the mixture were concentrated and AcCl (35.3 mg, 0.45 mmol), Et_3N (45.5 mg, 0.45 mmol) and DCM (5.0 mL) were added and stirred at rt for 6 h. The mixture were concentrated and purified by flash chromatography on a short silica (eluent: PE/EA = 10/1) to afford 1.95 g (72%) of **60**. Yellow solid.

2) Late-stage functionalization of complex bioactive molecules

2.1). The C-C amination of tocopherol -derivative:

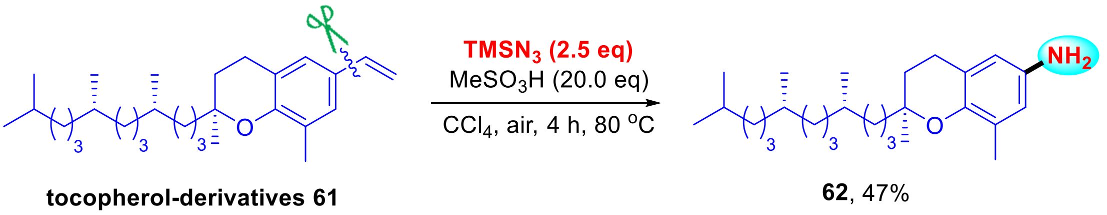

(R)-2,8-Dimethyl-2-((2S,4S)-2,4,6-trimethylheptyl)chroman-6-amine (62)

The reaction of tocopherol-derivatives **61** (82.4 g, 0.2 mmol), $TMSN_3$ (57.6 mg, 0.5 mmol) in CCl_4 (1.0 mL) followed by the addition of $MeSO_3H$ (260 uL, 4 mmol), then the mixture was stirred under air at 80 °C for 4 h to afford 37.8 mg (47%) of **62**. Yellow solid. **^{1}H NMR ($CDCl_3$, 400 MHz)**: δ 6.41 (s, 1H), 6.31 (s, 1H), 3.25 (s, 2H), 2.67 (t, J = 5.6 Hz, 2H), 2.12 (s, 3H), 1.85-1.68 (m, 2H), 1.62-1.28 (m, 15H), 1.21-1.03 (m, 9H), 0.90-0.86 (m, 12H).**^{13}C NMR ($CDCl_3$, 100 MHz)**: δ 145.24, 137.78, 126.98, 121.14, 116.57, 113.40, 75.33, 39.92, 39.35, 37.43, 37.27, 32.78, 32.69, 31.47, 27.97, 24.79, 24.44, 24.12, 22.72, 22.62, 22.45, 20.98, 19.74, 19.64, 16.02 ppm. HRMS m/z (ESI) calcd for $C_{27}H_{48}NO$ $(M + H)^+$, 402.3736, found 402.3731.

2.2). The C-C amination of tyrosine -derivative:

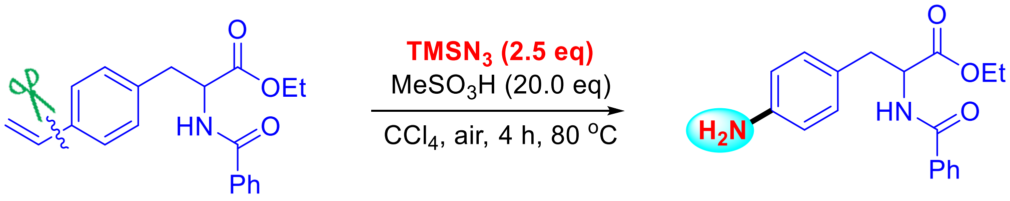

tyrosine derivatives 63 **64**, 64%

Ethyl 3-(4-aminophenyl)-2-benzamidopropanoate (64):

The reaction of tocopherol-derivatives **63** (64.6 g, 0.2 mmol), $TMSN_3$ (57.6 mg, 0.5 mmol) in CCl_4 (1.0 mL) followed by the addition of $MeSO_3H$ (260 uL, 4 mmol), then the mixture was stirred under air at 80 °C for 4 h to afford 40.0 mg (64%) of **64**. Yellow solid. **^{1}H NMR ($CDCl_3$, 400 MHz)**: δ 7.72 (m, 2H), 7.52–7.46 (m, 1H), 7.40 (m, 2H), 6.91 (d, *J* = 8.3 Hz, 2H), 6.60 (dd, *J* = 11.0, 4.5 Hz, 3H), 4.99 (m, *J* = 7.6, 1H), 4.21 (q, *J* = 7.1 Hz, 2H), 3.64 (s, 2H), 3.14 (t, *J* = 5.5 Hz, 2H), 1.28 (t, *J* = 7.1 Hz, 3H); **^{13}C NMR ($CDCl_3$, 100 MHz)**: δ 171.72, 166.70, 145.39, 133.96, 131.58, 130.18, 128.49, 126.93, 125.39, 115.19, 61.44, 53.62, 36.98, 14.11 ppm. HRMS *m/z* (ESI) calcd for $C_{18}H_{21}N_2O_3$ $(M + H)^+$, 313.1552, found 313.1551.

2.2). The C-C amination of tyrosine -derivative:

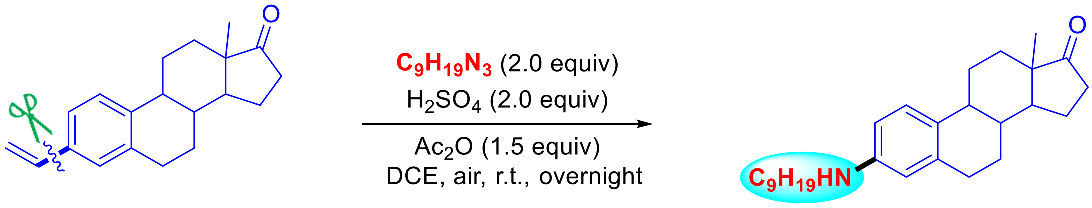

Estrone derivatives 65 **66**, 70%

13-methyl-3-(nonylamino)-6,7,8,9,11,12,13,14,15,16-decahydro-17Hcyclopenta[a]phenanthren-17-one (66):

To a 10 mL Schlenk flask containing 13-methyl-3-vinyl-6,7,8,9,11,12,13,14,15,16-decahydro-17H-cyclopenta[a]phenanthren-17-one **65** (56.1 mg, 0.2 mmol), 1-azidononane (67.7 mg, 0.4 mmol) and a magnetic stir bar, DCE (2 mL) and acetic anhydride (30.6 mg, 0.3 mmol) were added and the mixture was stirred at 25 °C. Conc. H_2SO_4 (39.3 mg, 0.4 mmol) were then added to the mixture in 5 seconds, and the mixture was stirred at 25 °C overnight. The reaction was quenched with 20% NaOH and the mixture was extracted with AcOEt, isolated by column chromatography (silica gel, petroleum ether/AcOEt = 50/1), affording 55 mg (70%) of **66** as colorless liquid. **^{1}H NMR (400 MHz, $CDCl_3$):** δ 7.09 (d, *J* = 8.4 Hz, 1H), 6.44 (dd, *J* = 8.4 Hz, 2.4 Hz, 1H), 6.35 (d, *J* = 1.6 Hz,

1H), 3.47 (brs, 1H), 3.07 (t, *J* = 7.0 Hz, 2H), 2.80-2.90 (m, 2H), 2.45-2.52 (m, 1H), 2.34-2.38 (m, 1H), 1.91-2.34 (m, 5H), 1.27-1.64 (m, 20H), 0.90 (s, 3H), 0.88 (t, *J* = 6.0 Hz, 3H). **^{13}C NMR (100 MHz, $CDCl_3$):** δ 221.0, 146.6, 137.1, 128.5, 126.0, 112.7, 110.8, 50.3, 48.0, 44.1, 43.9, 38.5, 35.8, 31.8, 31.5, 29.7, 29.6, 29.5, 29.4, 29.2, 27.1, 26.6, 25.9, 22.6, 21.5, 14.0, 13.8. **HRMS *m/z* (ESI)** calcd for $C_{27}H_{42}NO$ $[M+H]^+$ 396.3261, found: 396.3261.

G. Mechanistic experiments

In order to capture the intermediates of this process, we conducted an in situ reduction reaction with $NaBH_4$ as the hydrogenation reagent and arylamine **67** and **69** was produced in this process, which indicates that the protonated imine **68** and **70** was the intermediate of this reaction.

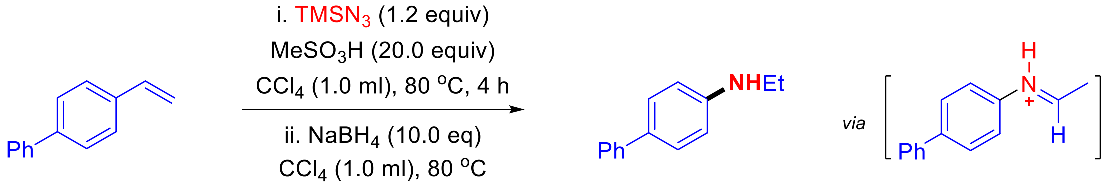

48 **67**, 55% yield **68**

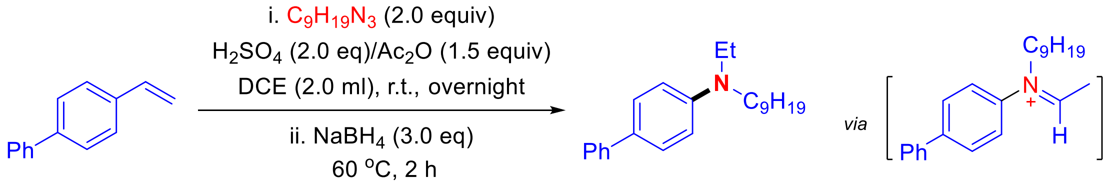

48 **69**, 41% yield **70**

The reaction of 4-vinyl-1,1'-biphenyl **48** (54.1 mg, 0.3 mmol), $TMSN_3$ (41.5 mg, 0.36 mmol) in CCl_4 (1.0 mL) followed by the addition of $MeSO_3H$ (390 uL, 6.0 mmol), then the mixture was stirred under air at 80 °C for 4 h. Then $NaBH_4$ (113 mg, 3.0 mmol), CCl_4 (1.0 mL) was added and stirred for 10 h. After cooling down to room temperature, quenched by 2 M NaOH (5 mL), extracted by EA, washed with brine and dried over Na_2SO_4. The combined organic phase were concentrated and purified by flash chromatography on a short silica gel (eluent: PE/EA = 3/1) to afford the 32.5 mg (55%) of **67.** Yellow solid.

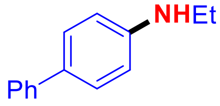

67

N-ethyl-[1,1'-biphenyl]-4-amine (67):[18]

^{1}H NMR ($CDCl_3$, 400 MHz): δ 7.60 (d, J = 7.8 Hz, 2H), 7.50 (d, J = 8.4 Hz, 2H), 7.44 (t, J = 7.6 Hz, 1H), 7.31 (t, J = 8.1 Hz, 2H), 6.73 (d, J = 8.4 Hz, 2H), 3.69 (s, 1H), 3.25 (q, J = 7.1 Hz, 2H), 1.33 (t, J = 7.1 Hz, 3H).; **^{13}C NMR ($CDCl_3$, 100 MHz)**: δ 147.81, 141.26, 130.01, 128.59, 127.87, 126.22, 125.95, 112.91, 38.44, 14.86. ppm; MS (70 eV): m/z (%) =197.2.

To a 10 mL Schlenk flask containing 4-vinyl-1,1'-biphenyl **48** (36.1 mg, 0.2 mmol), 1-azidononane (67.7 mg, 0.4 mmol) and a magnetic stir bar, DCE (2 mL) and acetic anhydride (30.6 mg, 0.3 mmol) were added and the mixture was stirred at 25 °C. Conc. H_2SO_4 (39.3 mg, 0.4 mmol) were then added

to the mixture in 5 seconds, and the mixture was stirred at 25 °C overnight. $NaBH_4$ (22.7 mg, 0.6 mmol) were added and the mixture was stirred at 60 °C for 2 h. The reaction was quenched with saturated $NaHCO_3$ and the mixture was extracted with AcOEt, isolated by column chromatography (silica gel, petroleum ether/AcOEt = 50/1), affording 26.2 mg (41%) of **69** as colorless liquid.

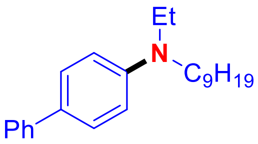

69

N-ethyl-N-nonyl-[1,1'-biphenyl]-4-amine (69):

^{1}H NMR (400 MHz, $CDCl_3$): δ 7.54 (d, *J* = 7.2 Hz, 2H), 7.47 (d, *J* = 8.8 Hz, 2H), 7.37 (t, *J* = 7.8 Hz, 2H), 7.22 (t, *J* = 7.4 Hz, 1H), 6.72 (d, *J* = 9.2 Hz, 2H), 3.39 (q, *J* = 7.0 Hz, 2H), 3.27 (t, *J* = 7.8 Hz, 2H), 1.55-1.63 (m, 2H), 1.27-1.33 (m, 12H), 1.17 (t, *J* = 7.0 Hz, 3H), 0.89 (t, *J* = 6.6 Hz, 3H). **^{13}C NMR (100 MHz, $CDCl_3$):** δ 147.4, 141.3, 128.6, 127.9, 126.1, 125.7, 111.8, 50.5, 44.9, 31.9, 29.7, 29.6, 29.5, 29.3, 27.6, 27.2, 22.7, 14.1, 12.3. **HRMS *m/z* (ESI)** calcd for $C_{23}H_{34}N$ $[M+H]^+$ 324.2686, found: 324.2690.

What's more, when using the benzyl alcohol as substrate under the C-C hydroxylation conditions, the corresponding phenol could be provided in quantitative yield, suggesting that the benzylic cation is probably involved in this oxygenation process.

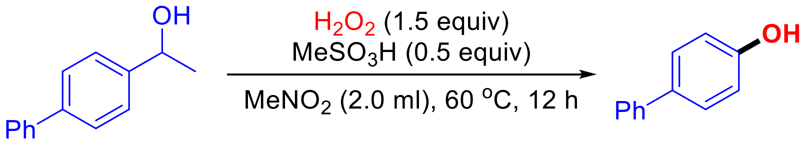

71 **72**, 96% yield

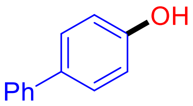

72

[1,1'-biphenyl]-4-ol (72):

The reaction of 1-([1,1'-biphenyl]-4-yl)ethan-1-ol **71** (59.4 mg, 0.3 mmol) in $MeNO_2$ (2.0 mL) followed by the addition of 30% aqueous hydrogen peroxide solution (51 mg, 0.45 mmol) and $MeSO_3H$ (10 uL, 0.15mmol), then the mixture was stirred under air at 60 °C for 12 h to afford 48.5 mg (95%) of **72**. yellow solid.

In order to explore the regiochemistry for the dealkenylative transformation. Substrates of **73** and **74** (**73** was commercially available and **74** was synthesized as the reported literature[19]) have been conducted under the standard conditions. The results follow the rule that the electron-rich benzylic carbon cation intermediate is more stable than the electron-deficient benzylic carbon cation intermediate during the hydroazidation of the hydroazidation of alkene.

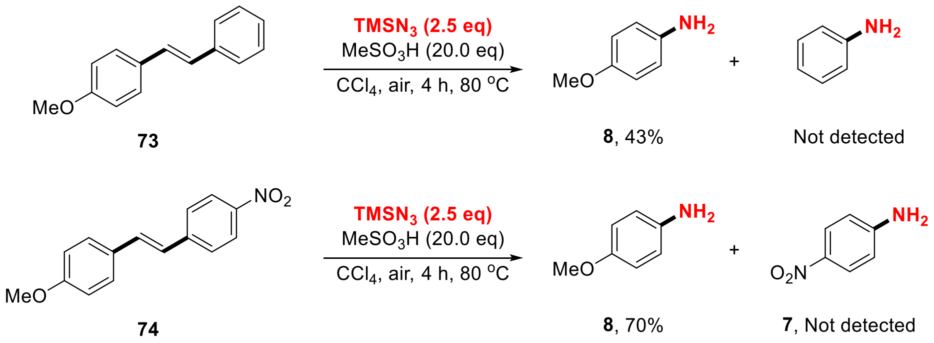

The reaction of (*E*)-1-methoxy-4-styrylbenzene **73** (63.1 mg, 0.3 mmol), $TMSN_3$ (41.5 mg, 0.36 mmol) in CCl_4 (1.0 mL) followed by the addition of $MeSO_3H$ (390 uL, 6.0 mmol), then the mixture was stirred under air at 80 °C for 4 h. The mixture was detected by HNMR using 1,1,2,2-tetrachloroethane as internal standard, the NMR yield of **8** in this reaction was 43% and the other substrate of aniline was not determined.

The reaction of (*E*)-1-methoxy-4-(4-nitrostyryl)benzene **74** (76.6 mg, 0.3 mmol), $TMSN_3$ (41.5 mg, 0.36 mmol) in CCl_4 (1.0 mL) followed by the addition of $MeSO_3H$ (390 uL, 6.0 mmol), then the mixture was stirred under air at 80 °C for 4 h. The mixture was detected by HNMR using 1,1,2,2-tetrachloroethane as internal standard, the NMR yield of **8** in this reaction was 70% and the other substrate of **7** was not determined.

H. References:

(1) Li, B.; Park, Y.; Chang, S. Regiodivergent Access to Five- and Six-Membered Benzo-Fused Lactams: Ru-Catalyzed Olefin Hydrocarbamoylation. *J. Am. Chem. Soc.* **2014,** *136*, 1125-1131.

(2) Beck, R.; Camadanli, S.; Flörke, U.; Klein, H.-F. Reactivity Diversification – Synthesis and Exchange Reactions of Cobalt and Iron 2-Alkenylpyridine/-pyrazine Complexes Obtained by Vinylic C(sp2)–H Activation. *Eur. J. Inorg. Chem.* **2015,** *2015*, 2543-2559.

(3) Song, S.; Huang, X.; Liang, Y.-F.; Tang, C.; Li, X.; Jiao, N. From simple organobromides or olefins to highly value-added bromohydrins: a versatile performance of dimethyl sulfoxide. *Green Chemistry* **2015,** *17*, 2727-2731.

(4) Yasu, Y.; Koike, T.; Akita, M. Intermolecular Aminotrifluoromethylation of Alkenes by Visible-Light-Driven Photoredox Catalysis. *Org. Lett.* **2013,** *15*, 2136-2139.

(5) Goseki, R.; Onuki, S.; Tanaka, S.; Ishizone, T.; Hirao, A. Living Anionic Polymerization of 1,4-Diisopropenylbenzene. *Macromolecules* **2015,** *48*, 3230-3238.

(6) Liu, J.; Qiu, X.; Huang, X.; Luo, X.; Zhang, C.; Wei, J.; Pan, J.; Liang, Y.; Zhu, Y.; Qin, Q.; Song, S.; Jiao, N. From alkylarenes to anilines via site-directed carbon–carbon amination. *Nat. Chem.* **2018**. DOI: org/10.1038/s41557-018-0156-y.

(7) Mo, X.; Morgan, T. D. R.; Ang, H. T.; Hall, D. G. Scope and Mechanism of a True Organocatalytic Beckmann Rearrangement with a Boronic Acid/Perfluoropinacol System under Ambient Conditions. *J. Am. Chem. Soc.* **2018,** *140*, 5264-5271.

(8) Qi, H.-L.; Chen, D.-S.; Ye, J.-S.; Huang, J.-M. Electrochemical Technique and Copper-Promoted Transformations: Selective Hydroxylation and Amination of Arylboronic Acids. *J. Org. Chem* **2013,** *78*, 7482-7487.

(9) Uberman, P. M.; García, C. S.; Rodríguez, J. R.; Martín, S. E. PVP-Pd nanoparticles as efficient catalyst for nitroarene reduction under mild conditions in aqueous media. *Green Chemistry* **2017,** *19*, 739-748.

(10) Mukhina, O. A.; Kuznetsov, D. M.; Cowger, T. M.; Kutateladze, A. G. Amino Azaxylylenes Photogenerated from o-Amido Imines: Photoassisted Access to Complex Spiro-Poly-Heterocycles. *Angew. Chem. Int. Ed.* **2015,** *54*, 11516-11520.

(11) García, N.; García-García, P.; Fernández-Rodríguez, M. A.; Rubio, R.; Pedrosa, M. R.; Arnáiz, F. J.; Sanz, R. Pinacol as a New Green Reducing Agent: Molybdenum- Catalyzed Chemoselective Reduction of Sulfoxides and Nitroaromatics. *Adv. Synth. Catal.* **2012,** *354*, 321-327.

(12) Gawande, M. B.; Rathi, A. K.; Branco, P. S.; Nogueira, I. D.; Velhinho, A.; Shrikhande, J. J.; Indulkar, U. U.; Jayaram, R. V.; Ghumman, C. A. A.; Bundaleski, N.; Teodoro, O. M. N. D. Regio- and Chemoselective Reduction of Nitroarenes and Carbonyl Compounds over Recyclable Magnetic Ferrite-Nickel Nanoparticles (Fe_3O_4-Ni) by Using Glycerol as a Hydrogen Source. *Chem. - Eur. J.* **2012,** *18*, 12628-12632.

(13) Lundgren, R. J.; Peters, B. D.; Alsabeh, P. G.; Stradiotto, M. A P,N-Ligand for Palladium-Catalyzed Ammonia Arylation: Coupling of Deactivated Aryl Chlorides, Chemoselective Arylations, and Room Temperature Reactions. *Angew. Chem. Int. Ed.* **2010,** *49*, 4071-4074.

(14) Feng, J.; Handa, S.; Gallou, F.; Lipshutz, B. H. Safe and Selective Nitro Group Reductions Catalyzed by Sustainable and Recyclable Fe/ppm Pd Nanoparticles in Water at Room Temperature. *Angew. Chem. Int. Ed.* **2016,** *55*, 8979-8983.

(15) Tlili A.; Xia, N.; Monnier F.; Taillefer, M. A Very Simple Copper-Catalyzed Synthesis of Phenols Employing Hydroxide Salts. *Angew. Chem. Int. Ed.* **2009**, *48*, 8725 –8728.

(16) Xia, S.; Gan, L.; Wang, K.; Li, Z.; Ma, D. Copper-Catalyzed Hydroxylation of (Hetero)aryl Halides under Mild Conditions. *J. Am. Chem. Soc*. **2016**, *138*, 13493−13496.
(17) Zheng, Y-W.; Chen, B.; Ye, P,; Feng, K.; Wang, W.; Meng, Q-Y.; Wu, L-Z.; Tung, C-H. Photocatalytic Hydrogen-Evolution Cross-Couplings: Benzene C−H Amination and Hydroxylation. J. *Am. Chem. Soc*. **2016**, *138*, 10080−10083.
(18) Pedersen, L.; Mady, M. F.; Sydnes, M. O. One-pot Suzuki–Miyaura cross-coupling followed by reductive monoalkylation of the resulting nitro biaryl system utilizing Pd/C as catalyst. *Tetrahedron Lett.* **2013,** *54*, 4772-4775.
(19) Zhang J-Z.; Tang, Y. Iron-Catalyzed Regioselective Oxo- and Hydroxy-Phthalimidation of Styrenes: Access to a-Hydroxyphthalimide Ketones. *Adv. Synth. Catal* **2016,** *358*, 752-764.

I. H NMR and ^{13}C NMR spectra of product

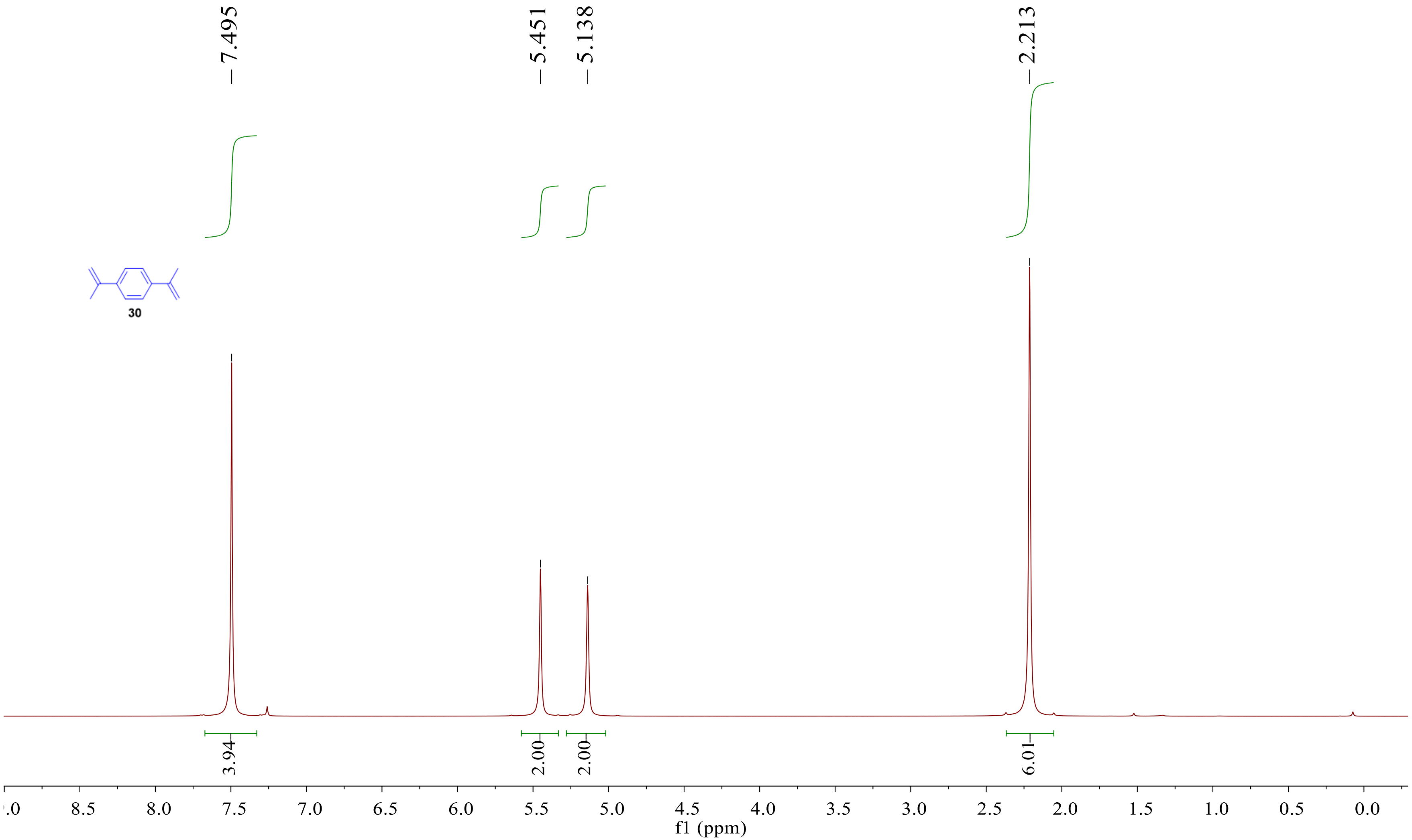

7.495
5.451
5.138
2.213
30
3.94
2.00
2.00
6.01
9.0 8.5 8.0 7.5 7.0 6.5 6.0 5.5 5.0 4.5 4.0 3.5 3.0 2.5 2.0 1.5 1.0 0.5 0.0
f1 (ppm)

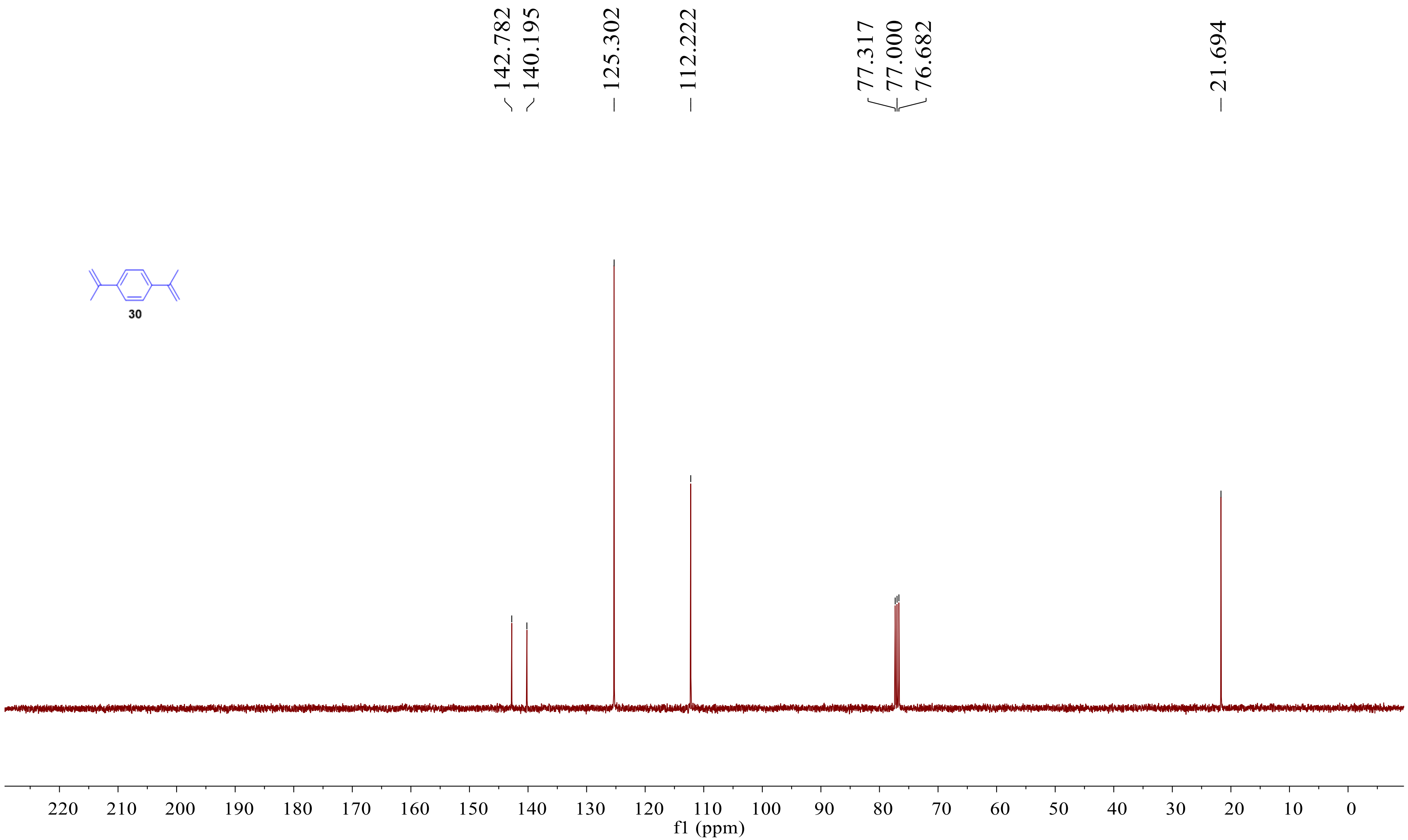
142.782
140.195
125.302
112.222
77.317
77.000
76.682
21.694
30
220 210 200 190 180 170 160 150 140 130 120 110 100 90 80 70 60 50 40 30 20 10 0
f1 (ppm)

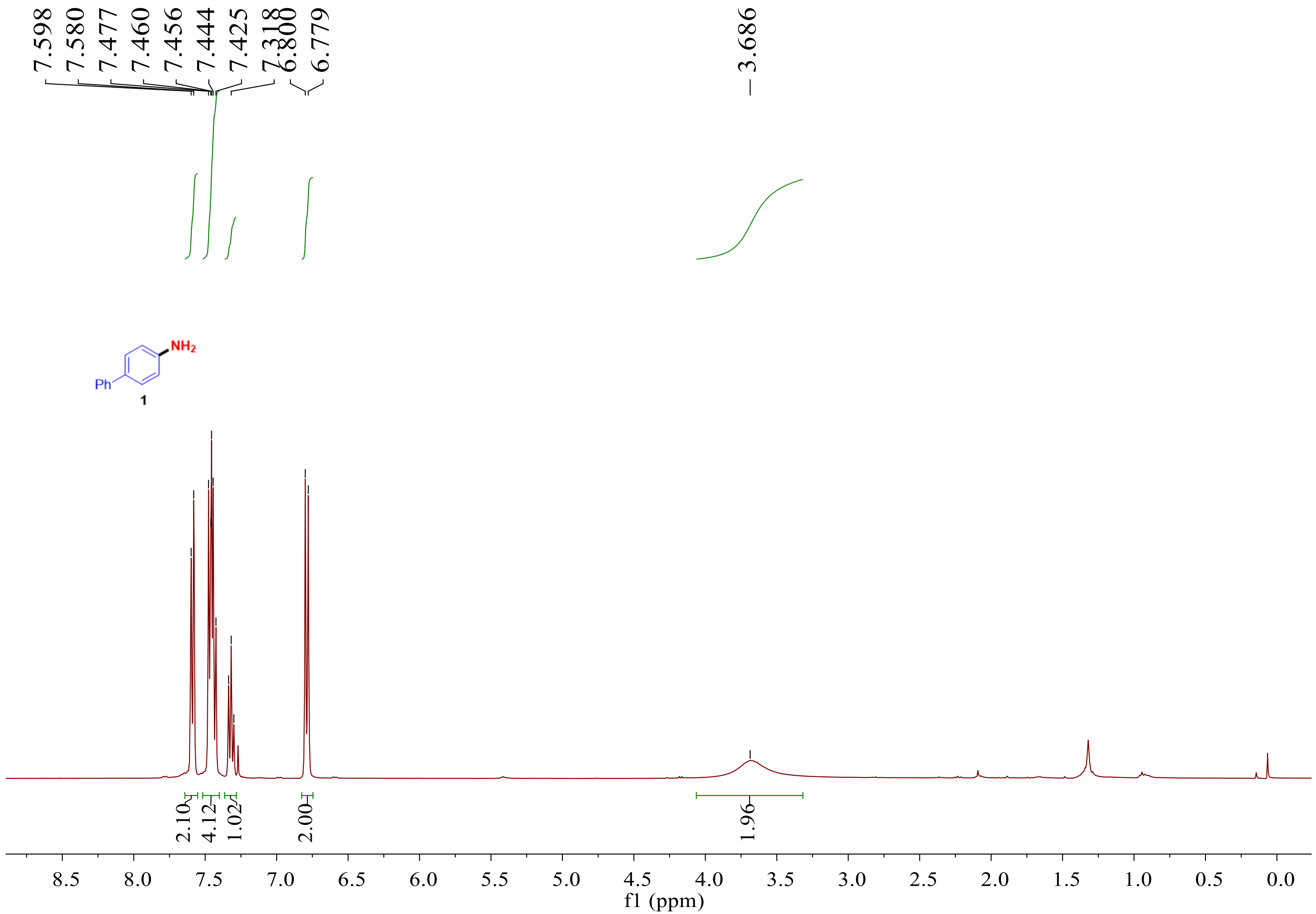
7.598
7.580
7.477
7.460
7.456
7.444
7.425
7.318
6.800
6.779
3.686
NH2
Ph
1
2.10
4.12
1.02
2.00
1.96
8.5
8.0
7.5
7.0
6.5
6.0
5.5
5.0
4.5
4.0
3.5
3.0
2.5
2.0
1.5
1.0
0.5
0.0
f1 (ppm)

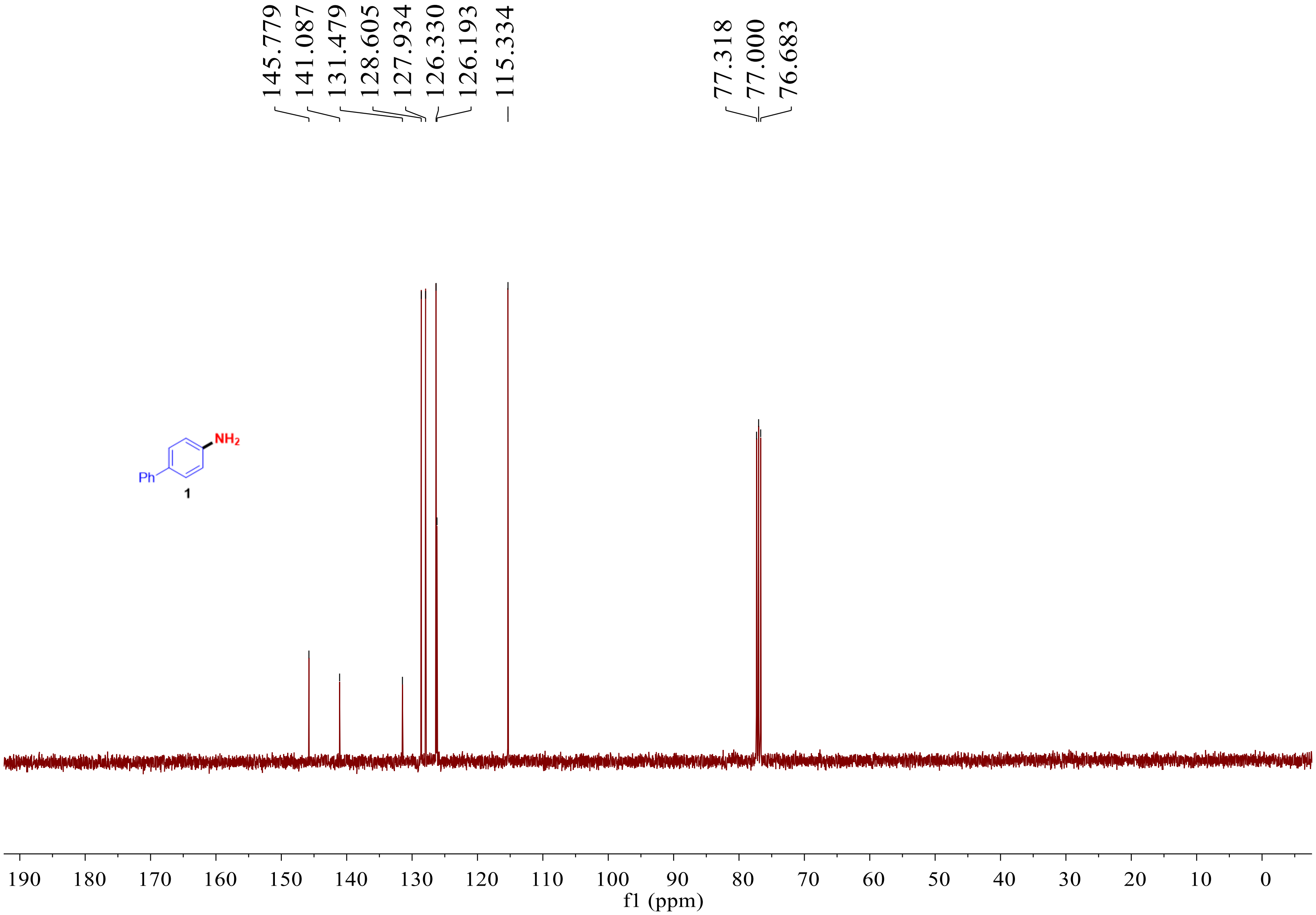
145.779
141.087
131.479
128.605
127.934
126.330
126.193
115.334
77.318
77.000
76.683
NH2
Ph
1
190 180 170 160 150 140 130 120 110 100 90 80 70 60 50 40 30 20 10 0
f1 (ppm)

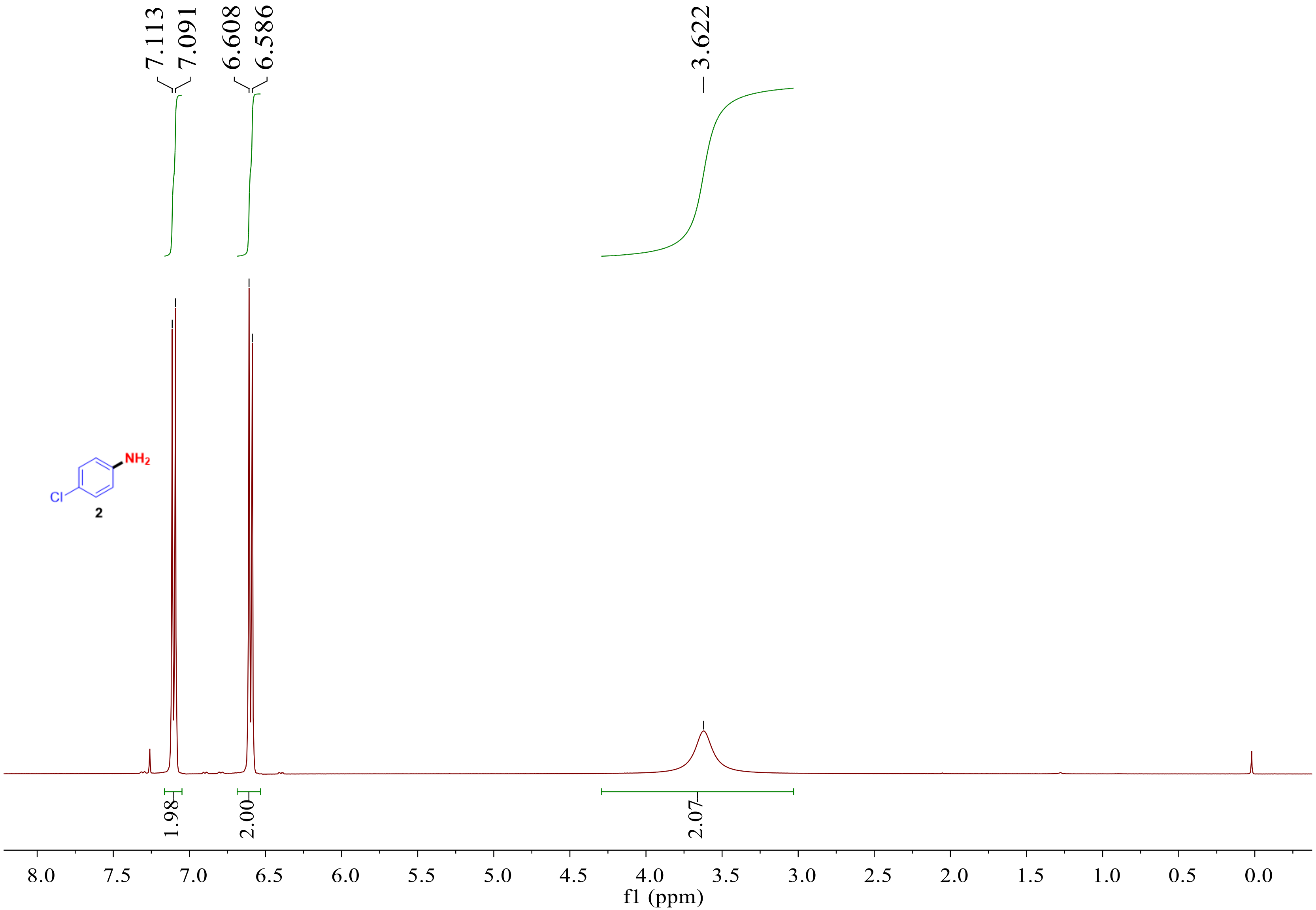
7.113
7.091
6.608
6.586
3.622
NH2
Cl
2
1.98
2.00
2.07
8.0
7.5
7.0
6.5
6.0
5.5
5.0
4.5
4.0
3.5
3.0
2.5
2.0
1.5
1.0
0.5
0.0
f1 (ppm)

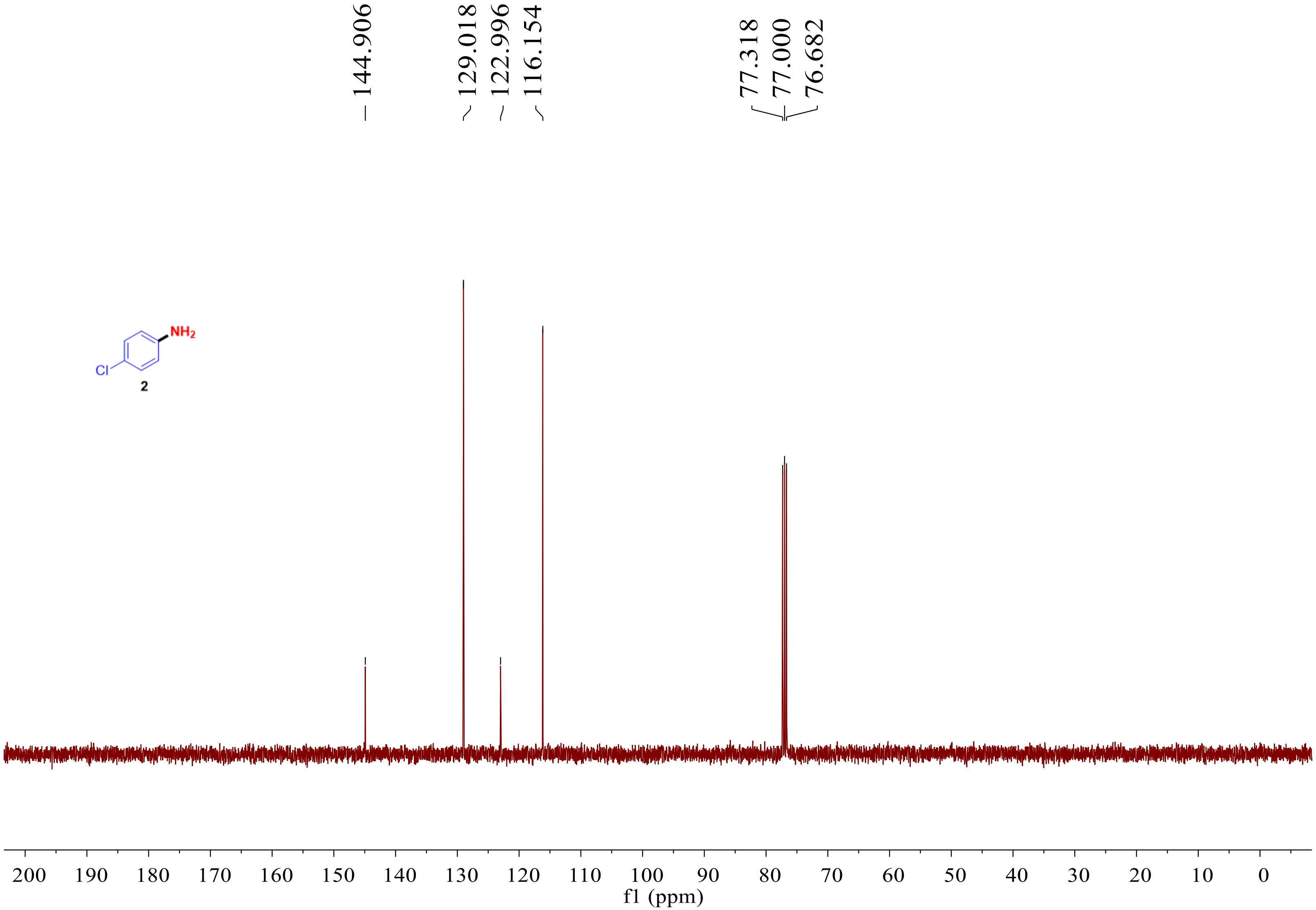
144.906
129.018
122.996
116.154
77.318
77.000
76.682
NH2
Cl
2
200 190 180 170 160 150 140 130 120 110 100 90 80 70 60 50 40 30 20 10 0
f1 (ppm)

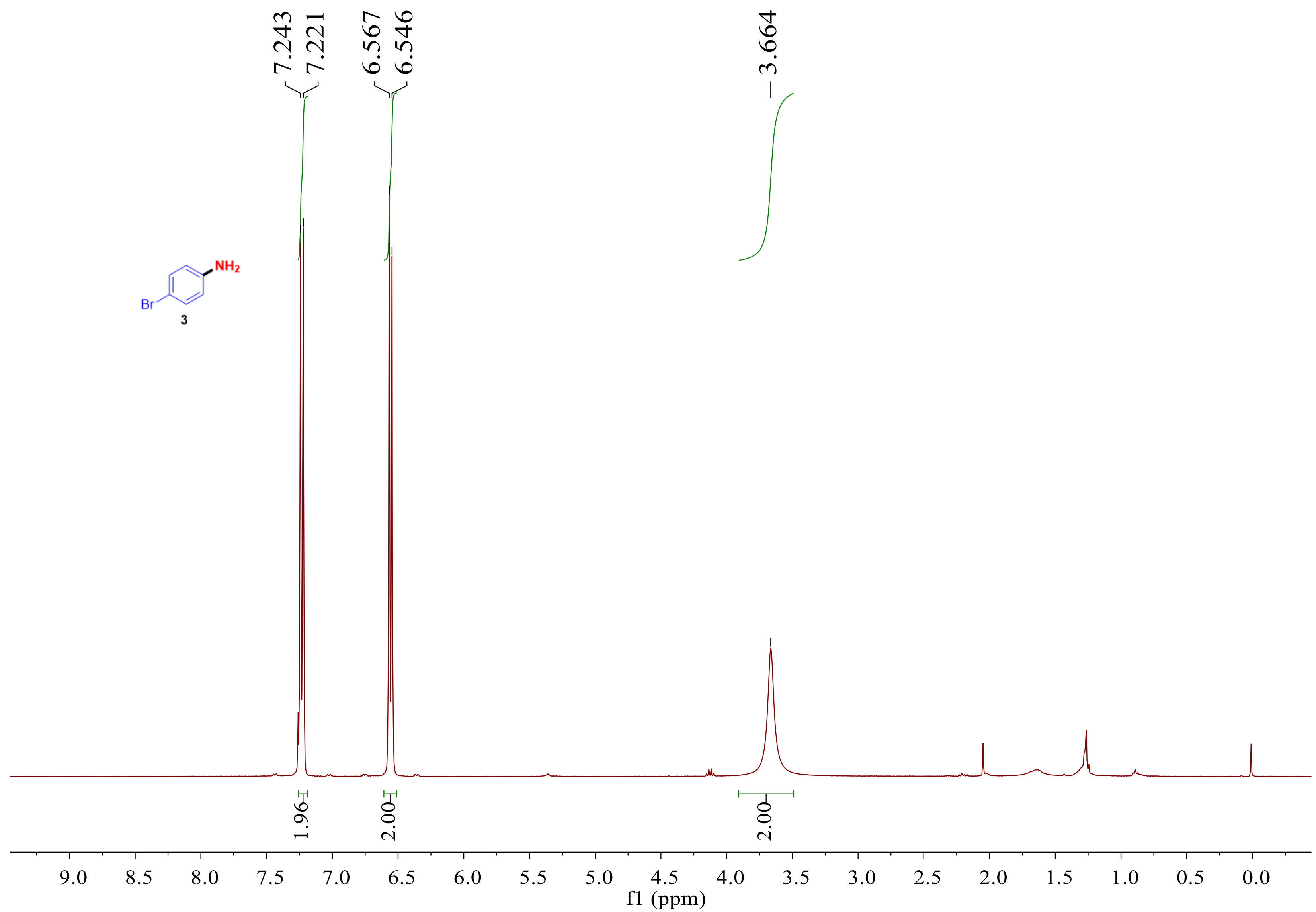

7.243
7.221
6.567
6.546
3.664
NH2
Br
3
1.96
2.00
2.00
9.0
8.5
8.0
7.5
7.0
6.5
6.0
5.5
5.0
4.5
4.0
3.5
3.0
2.5
2.0
1.5
1.0
0.5
0.0
f1 (ppm)

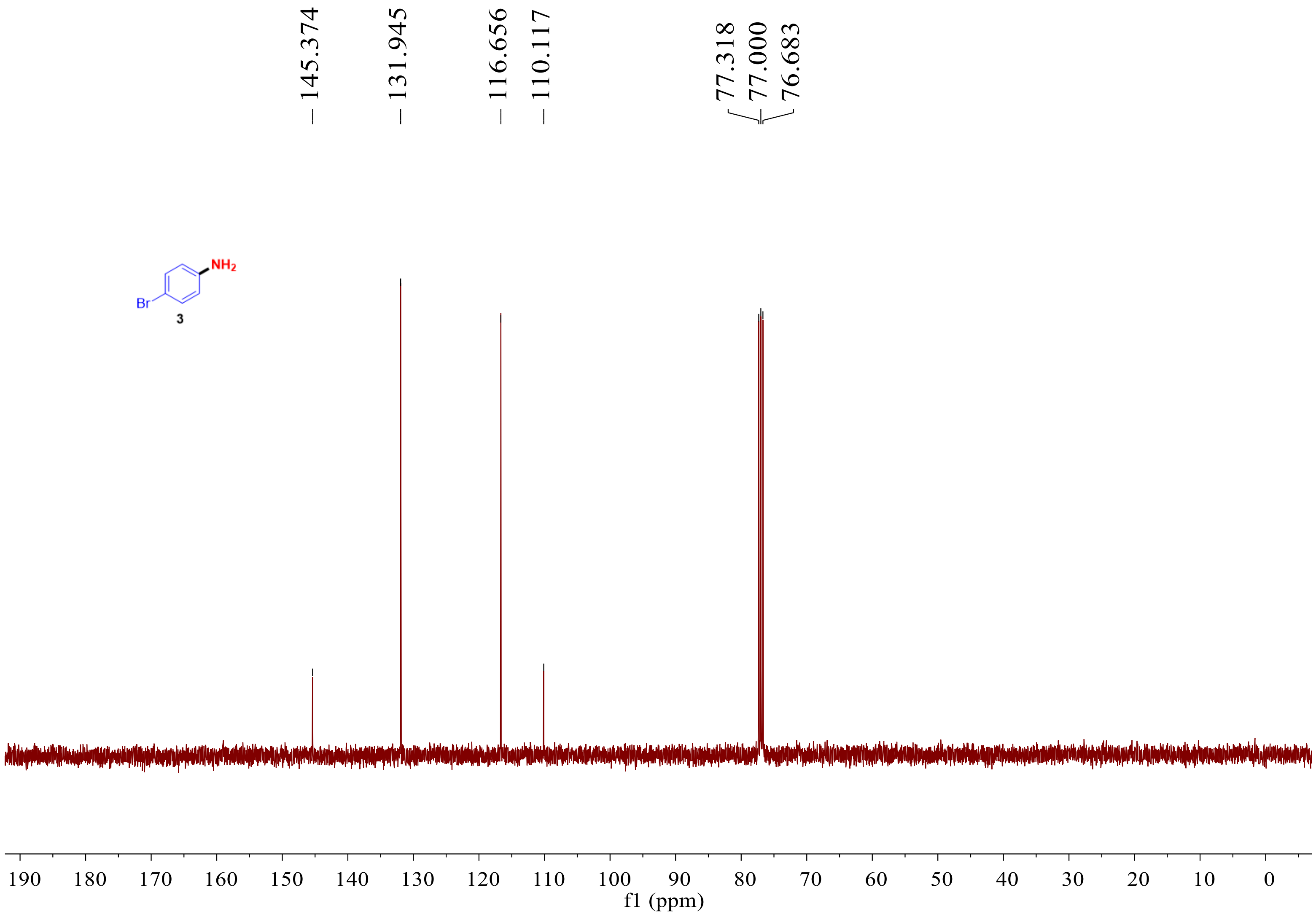
145.374
131.945
116.656
110.117
77.318
77.000
76.683
NH2
Br
3
190 180 170 160 150 140 130 120 110 100 90 80 70 60 50 40 30 20 10 0
f1 (ppm)

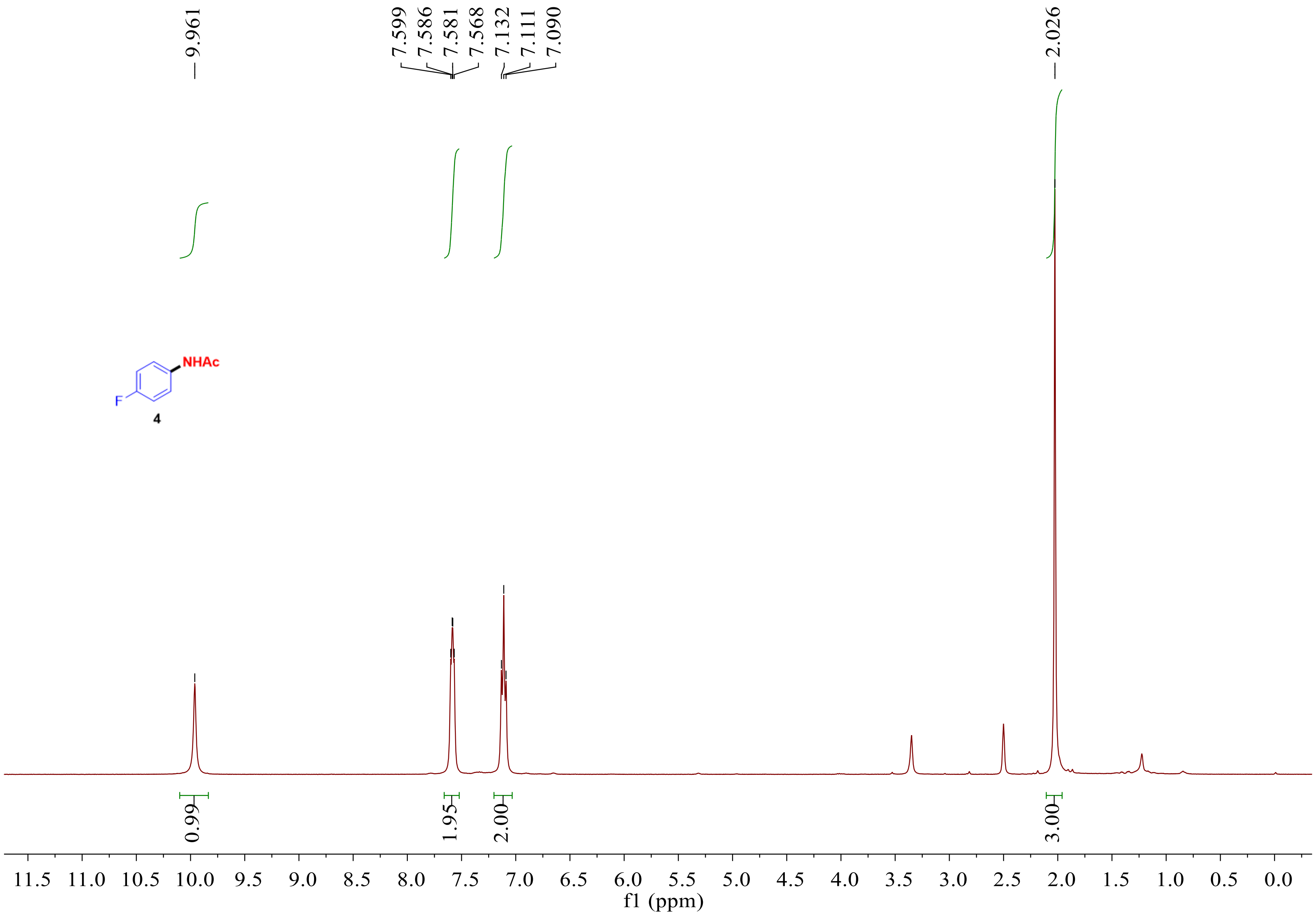
9.961
7.599
7.586
7.581
7.568
7.132
7.111
7.090
2.026
NHAc
F
0.99
1.95
2.00
3.00
11.5 11.0 10.5 10.0 9.5 9.0 8.5 8.0 7.5 7.0 6.5 6.0 5.5 5.0 4.5 4.0 3.5 3.0 2.5 2.0 1.5 1.0 0.5 0.0
f1 (ppm)

4

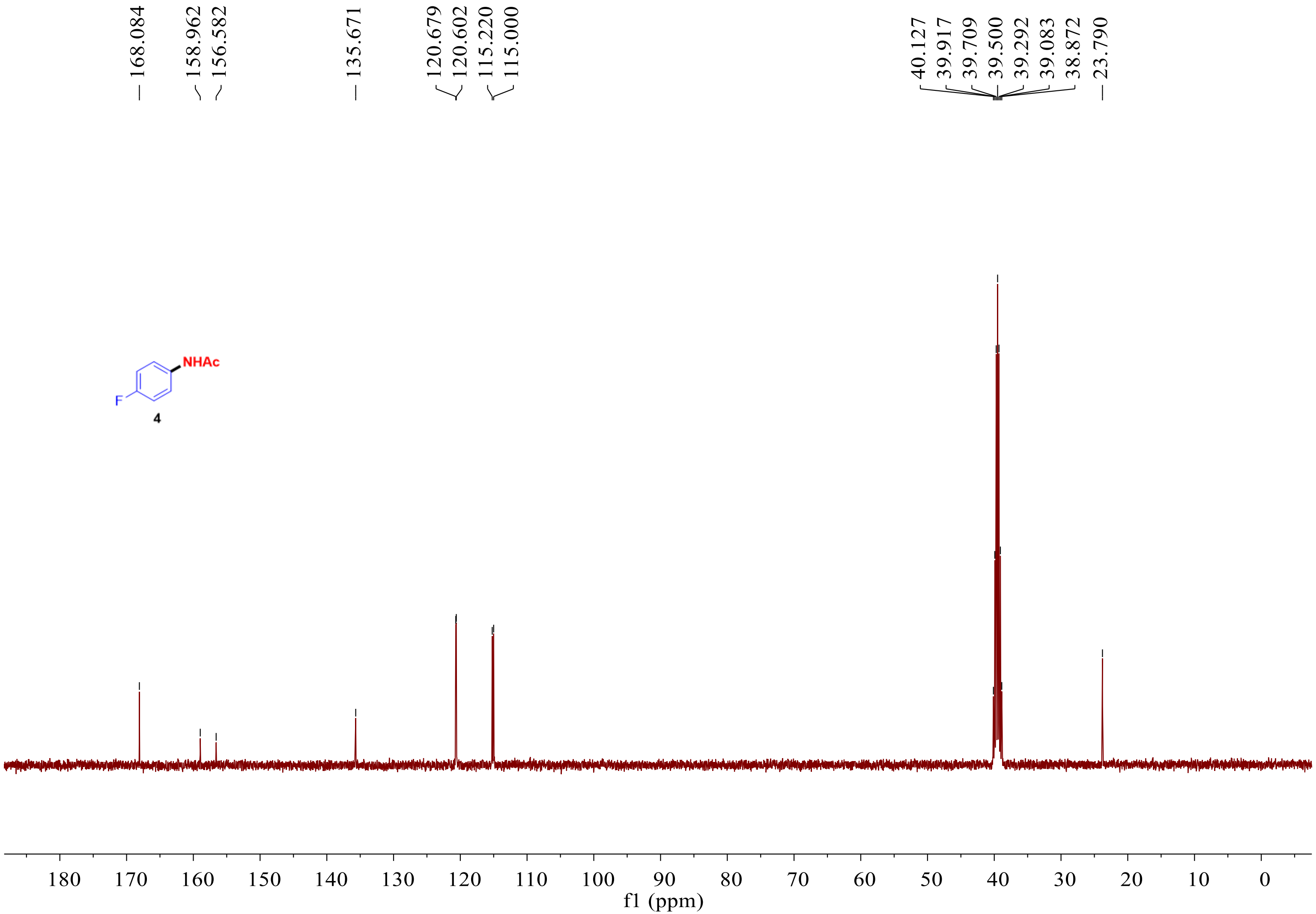
168.084
158.962
156.582
135.671
120.679
120.602
115.220
115.000
40.127
39.917
39.709
39.500
39.292
39.083
38.872
23.790
NHAc
F
4
180
170
160
150
140
130
120
110
100
90
80
70
60
50
40
30
20
10
0
f1 (ppm)

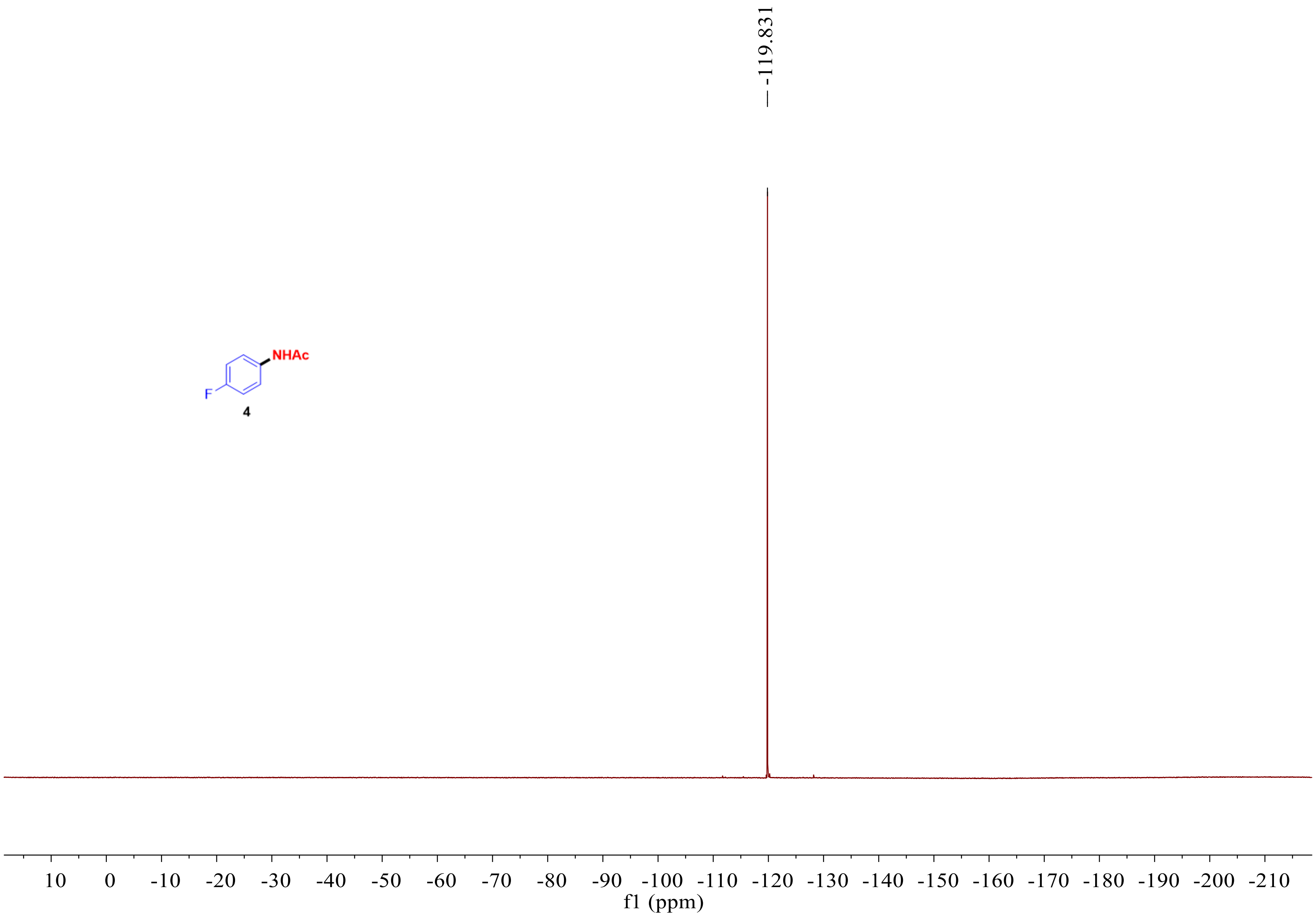

-119.831
NHAc
F
10
0
-10
-20
-30
-40
-50
-60
-70
-80
-90
-100
-110
-120
-130
-140
-150
-160
-170
-180
-190
-200
-210
f1 (ppm)

4

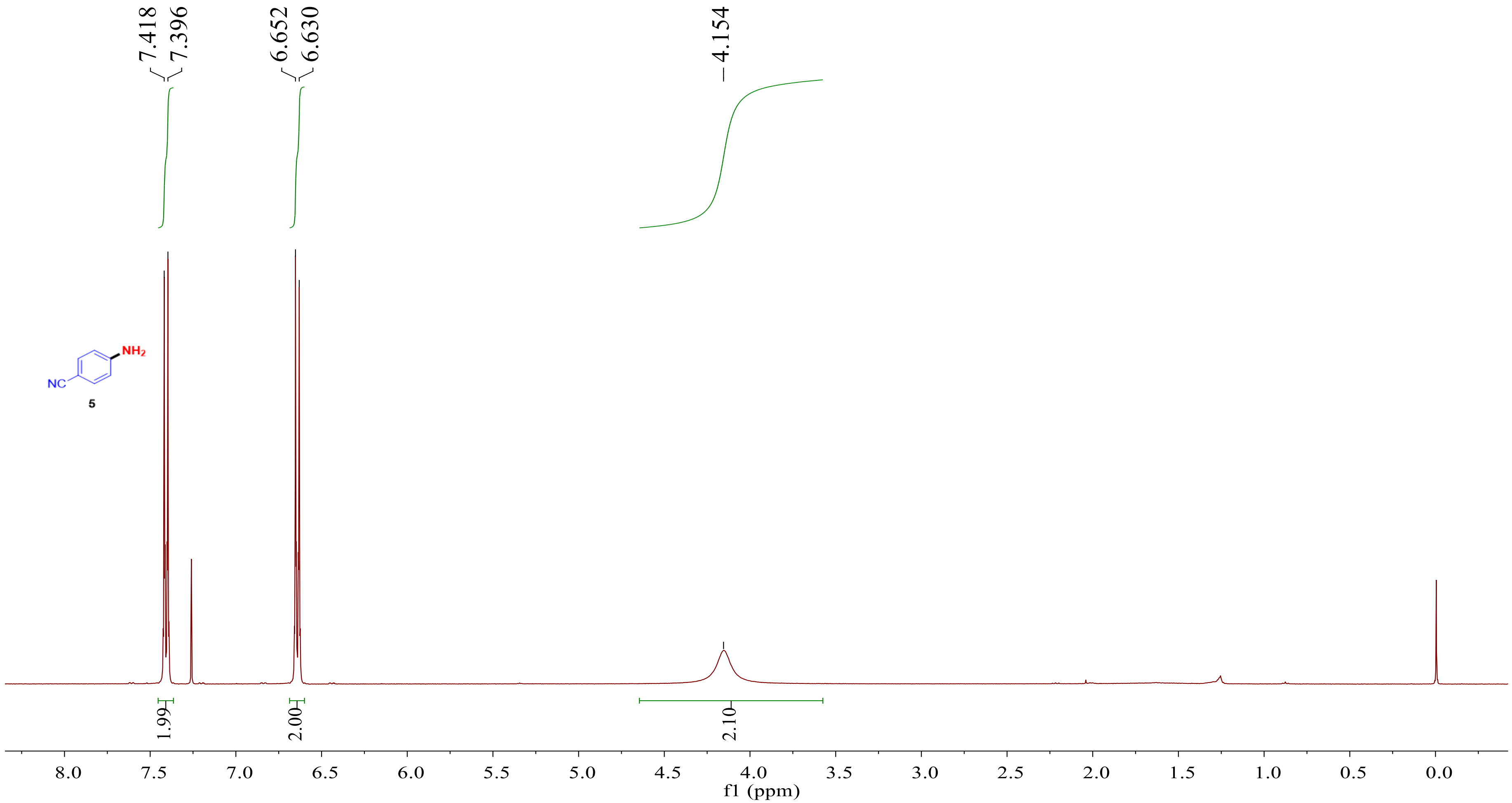

7.418
7.396
6.652
6.630
4.154
NH2
NC
5
1.99
2.00
2.10
8.0 7.5 7.0 6.5 6.0 5.5 5.0 4.5 4.0 3.5 3.0 2.5 2.0 1.5 1.0 0.5 0.0
f1 (ppm)

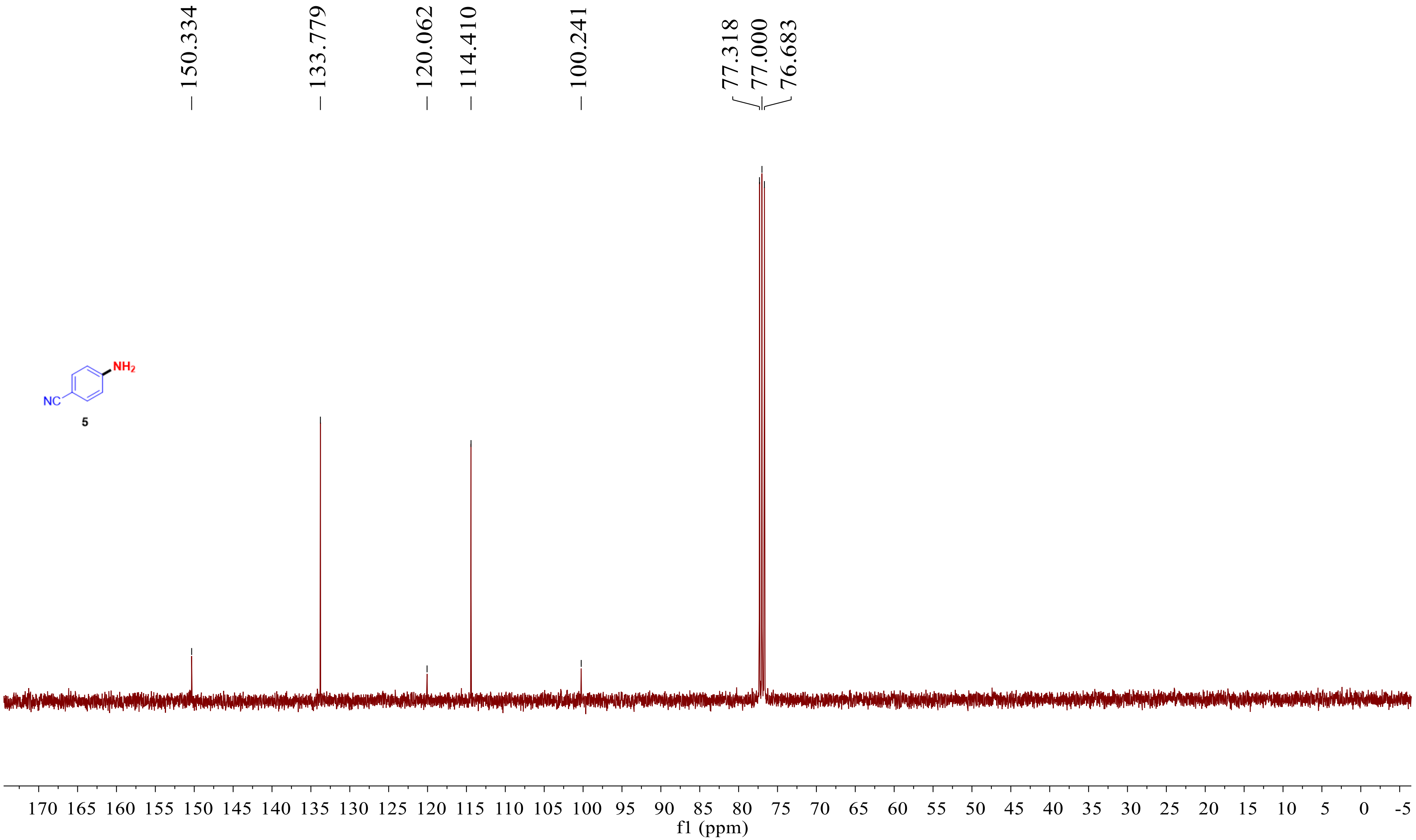
150.334
133.779
120.062
114.410
100.241
77.318
77.000
76.683
NH2
NC
5
170 165 160 155 150 145 140 135 130 125 120 115 110 105 100 95 90 85 80 75 70 65 60 55 50 45 40 35 30 25 20 15 10 5 0 -5
f1 (ppm)

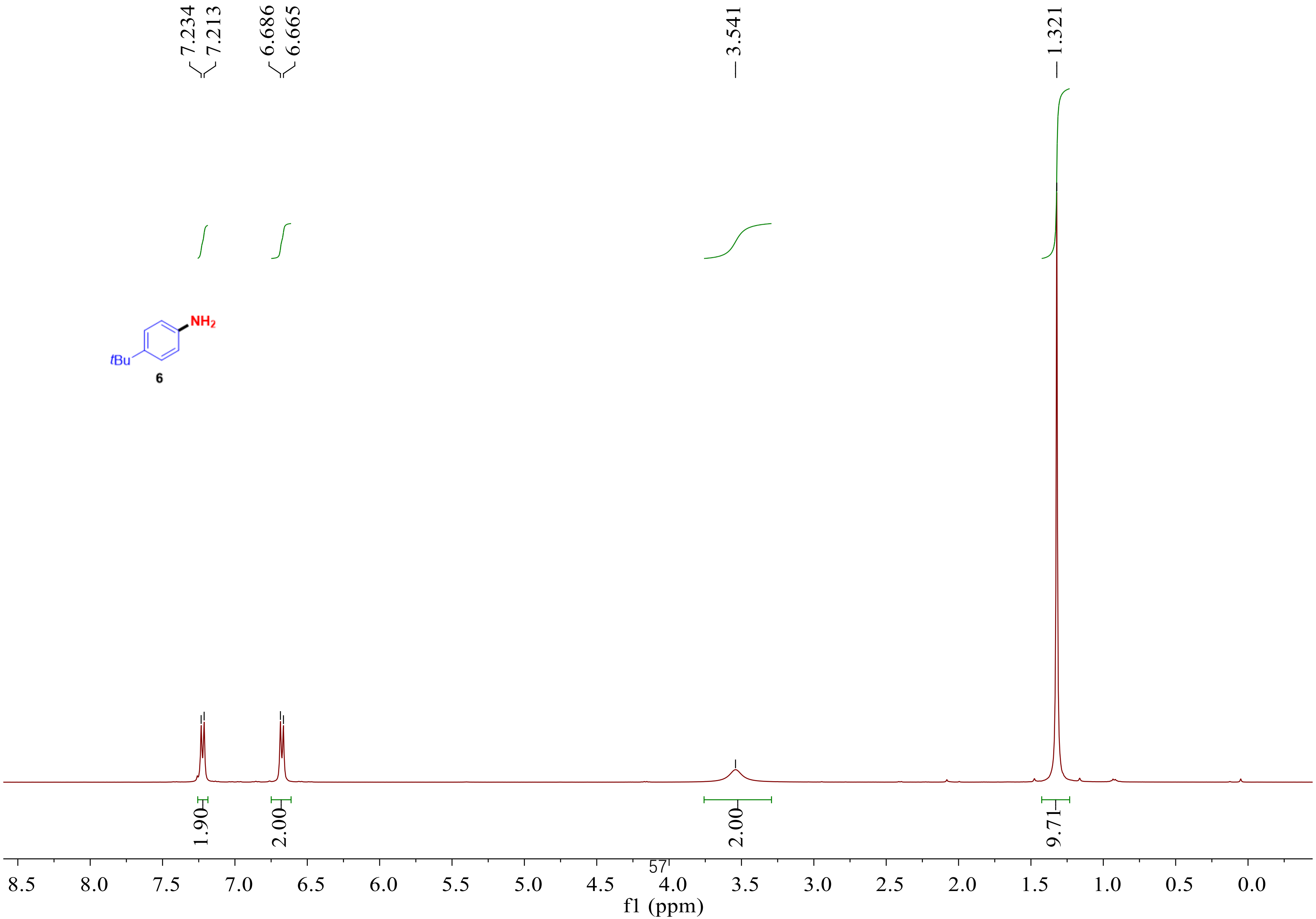

7.234
7.213
6.686
6.665
3.541
1.321
NH2
tBu
6
1.90
2.00
2.00
9.71
8.5 8.0 7.5 7.0 6.5 6.0 5.5 5.0 4.5 4.0 3.5 3.0 2.5 2.0 1.5 1.0 0.5 0.0
f1 (ppm)

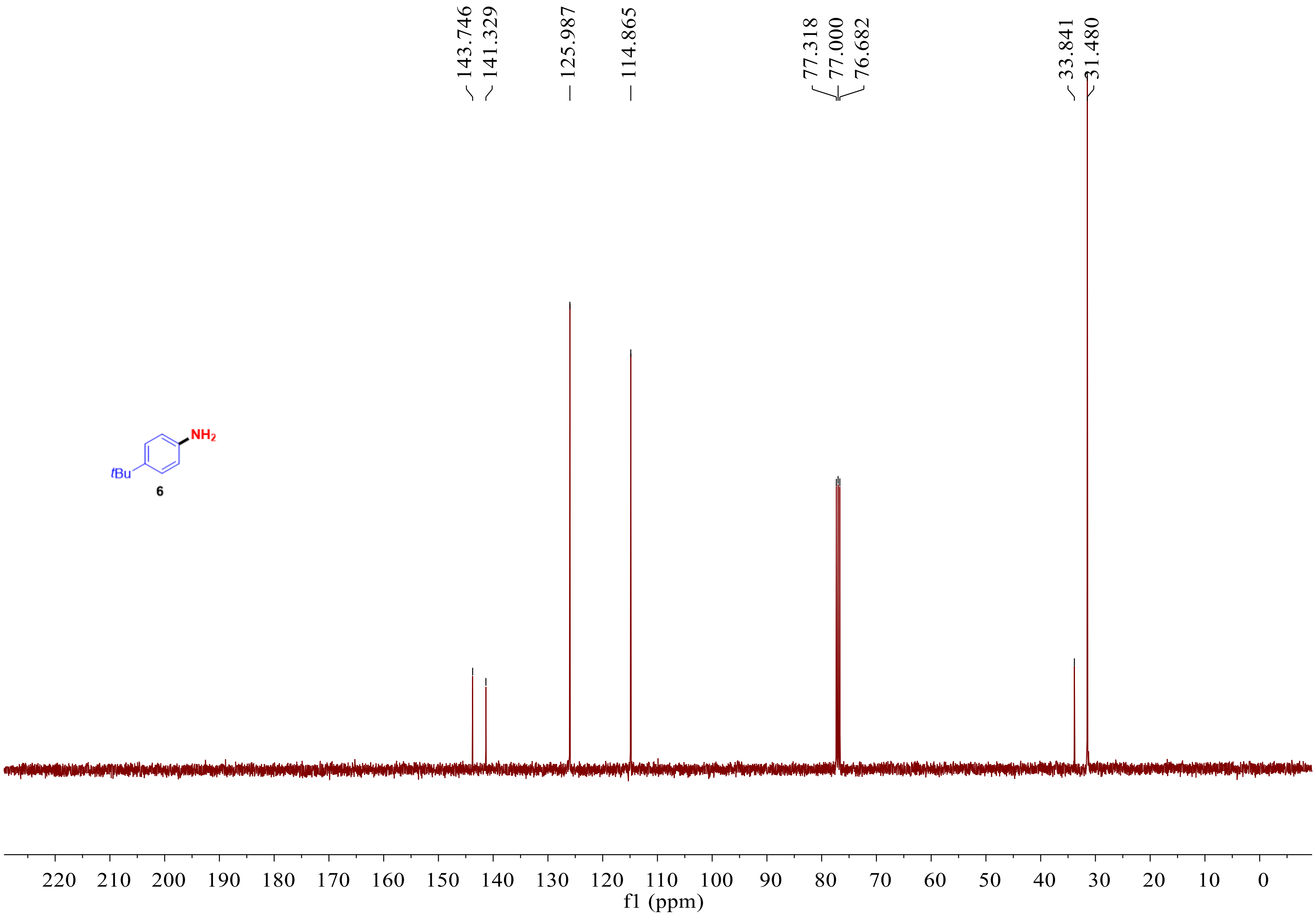
143.746
141.329
125.987
114.865
77.318
77.000
76.682
33.841
31.480
NH2
tBu
6
220 210 200 190 180 170 160 150 140 130 120 110 100 90 80 70 60 50 40 30 20 10 0
f1 (ppm)

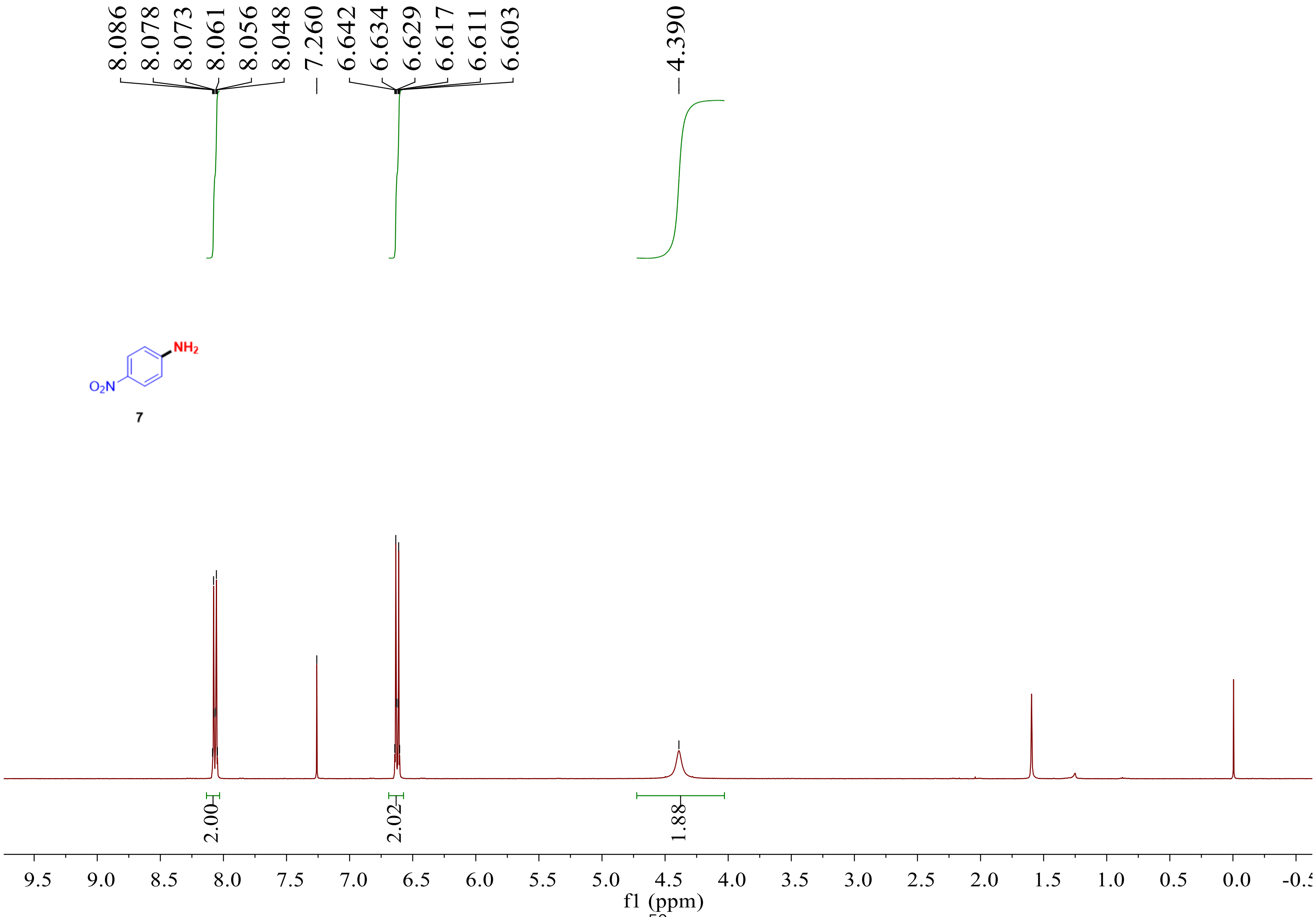
8.086
8.078
8.073
8.061
8.056
8.048
7.260
6.642
6.634
6.629
6.617
6.611
6.603
4.390
NH2
O2N
7
2.00
2.02
1.88
9.5
9.0
8.5
8.0
7.5
7.0
6.5
6.0
5.5
5.0
4.5
4.0
3.5
3.0
2.5
2.0
1.5
1.0
0.5
0.0
f1 (ppm)

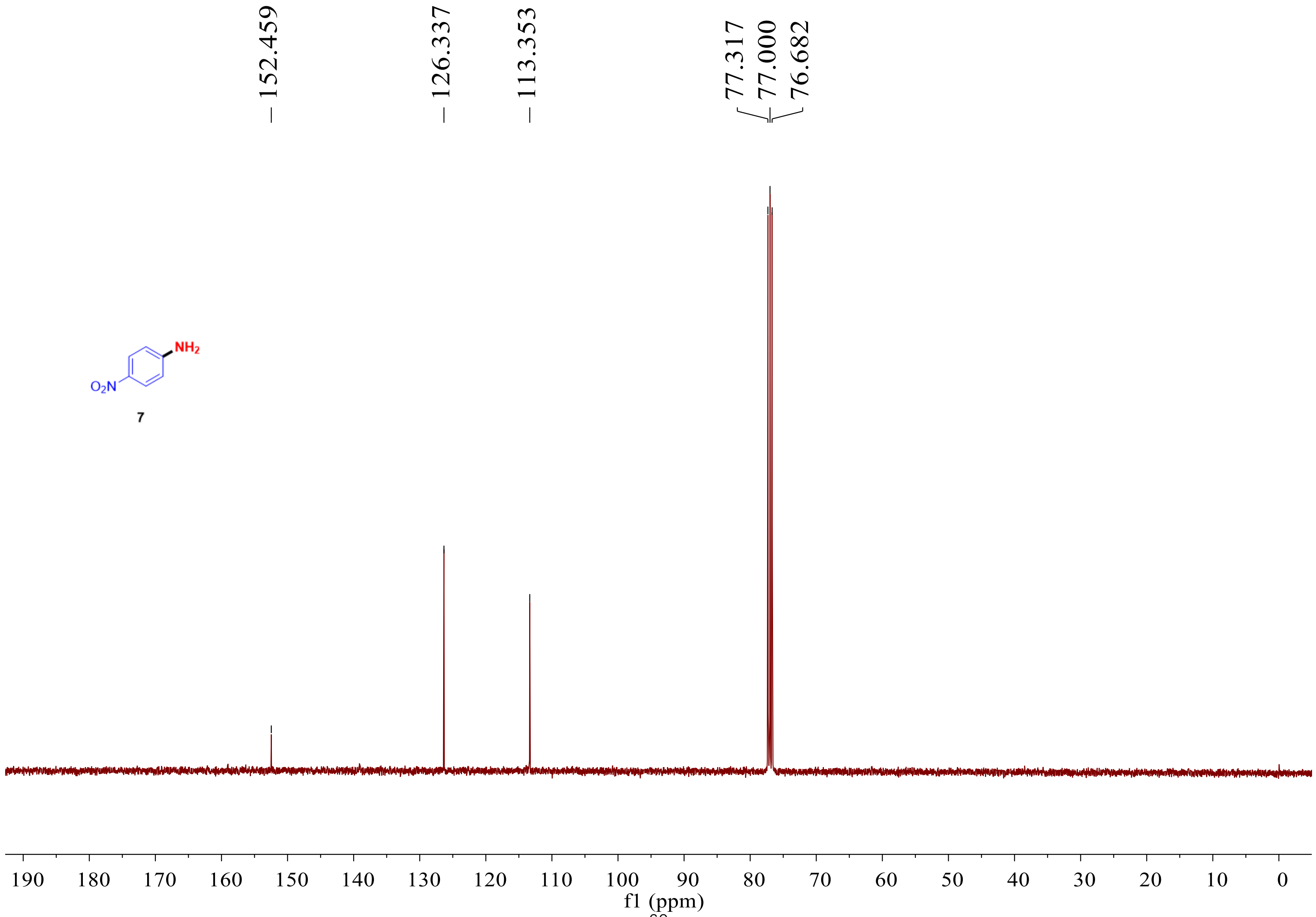
152.459
126.337
113.353
77.317
77.000
76.682
NH2
O2N
7
190 180 170 160 150 140 130 120 110 100 90 80 70 60 50 40 30 20 10 0
f1 (ppm)

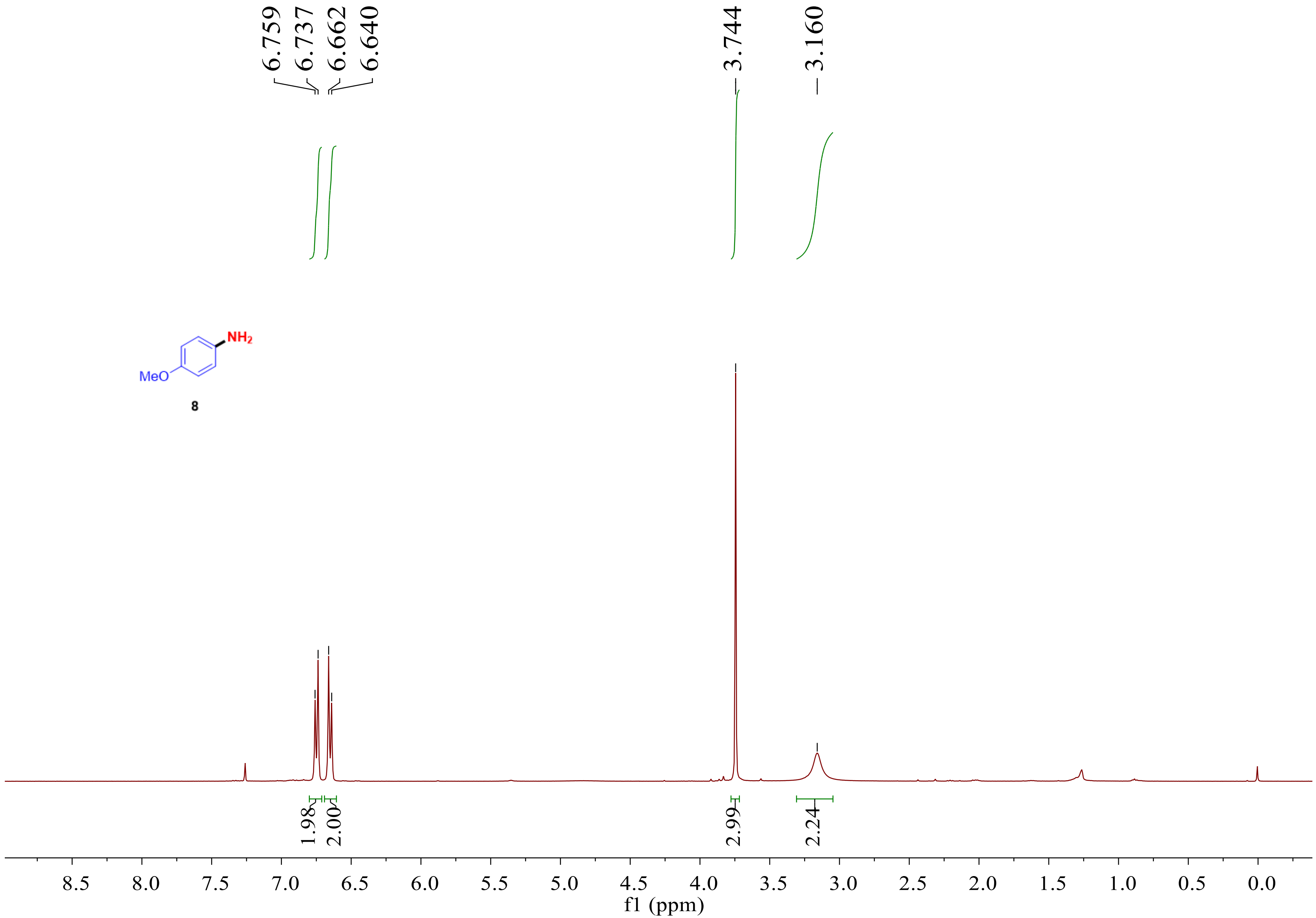
6.759
6.737
6.662
6.640
3.744
3.160
NH2
MeO
8
1.98
2.00
2.99
2.24
8.5
8.0
7.5
7.0
6.5
6.0
5.5
5.0
4.5
4.0
3.5
3.0
2.5
2.0
1.5
1.0
0.5
0.0
f1 (ppm)

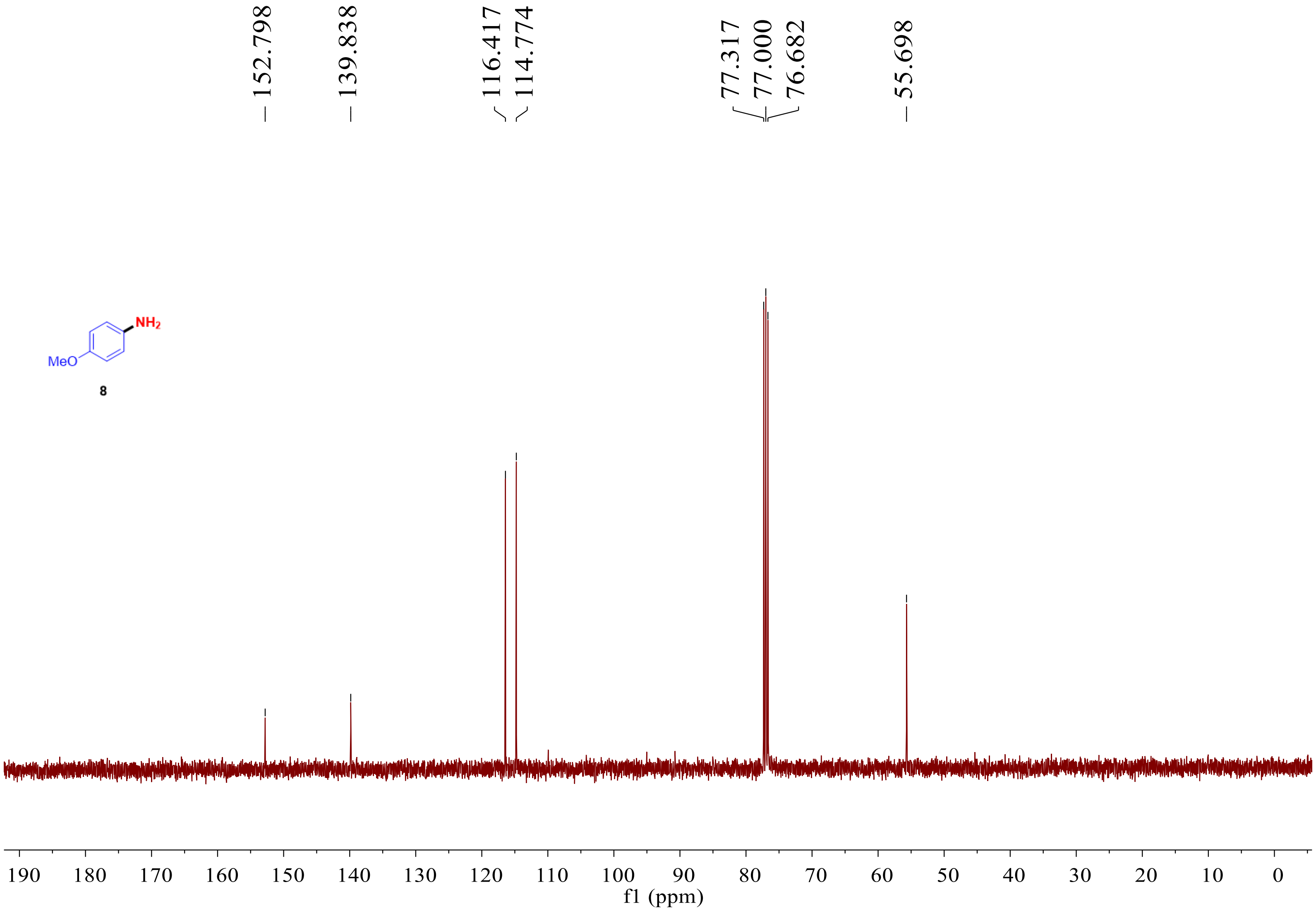
152.798
139.838
116.417
114.774
77.317
77.000
76.682
55.698
NH2
MeO
8
190 180 170 160 150 140 130 120 110 100 90 80 70 60 50 40 30 20 10 0
f1 (ppm)

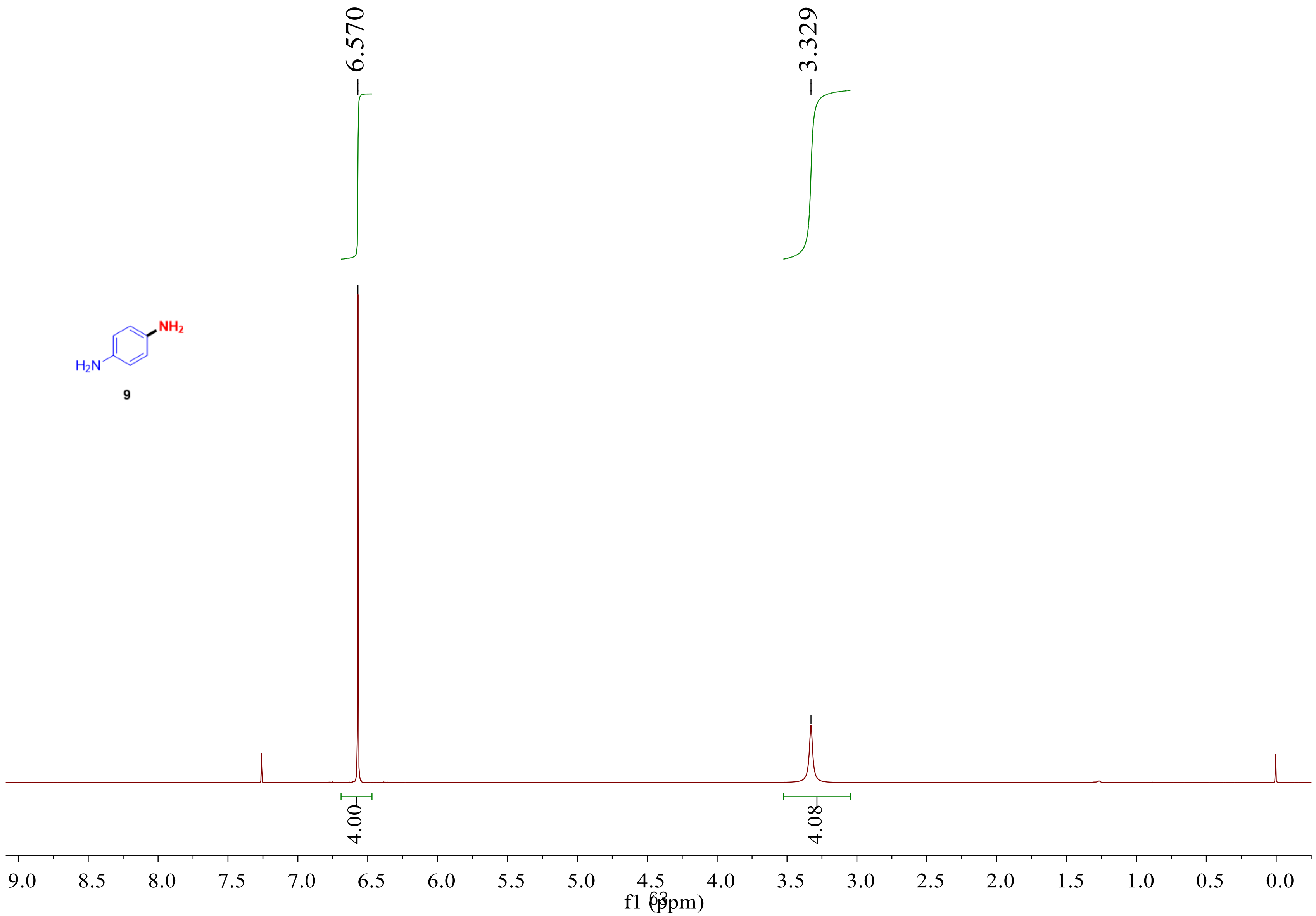
6.570
3.329
NH2
H2N
9
4.00
4.08
9.0 8.5 8.0 7.5 7.0 6.5 6.0 5.5 5.0 4.5 4.0 3.5 3.0 2.5 2.0 1.5 1.0 0.5 0.0
f1 (ppm)

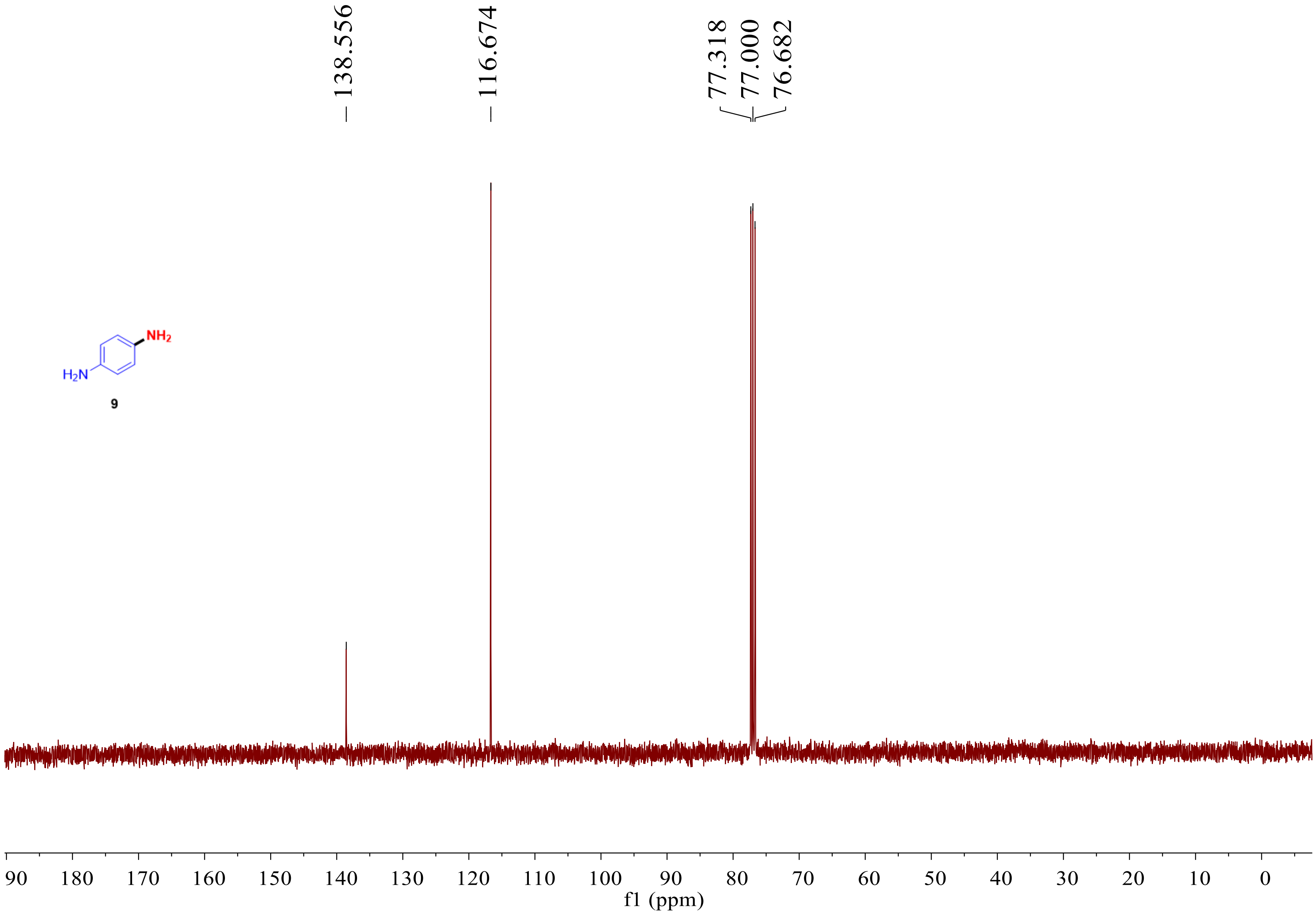
138.556
116.674
77.318
77.000
76.682
H2N
NH2
9
90
180
170
160
150
140
130
120
110
100
90
80
70
60
50
40
30
20
10
0
f1 (ppm)

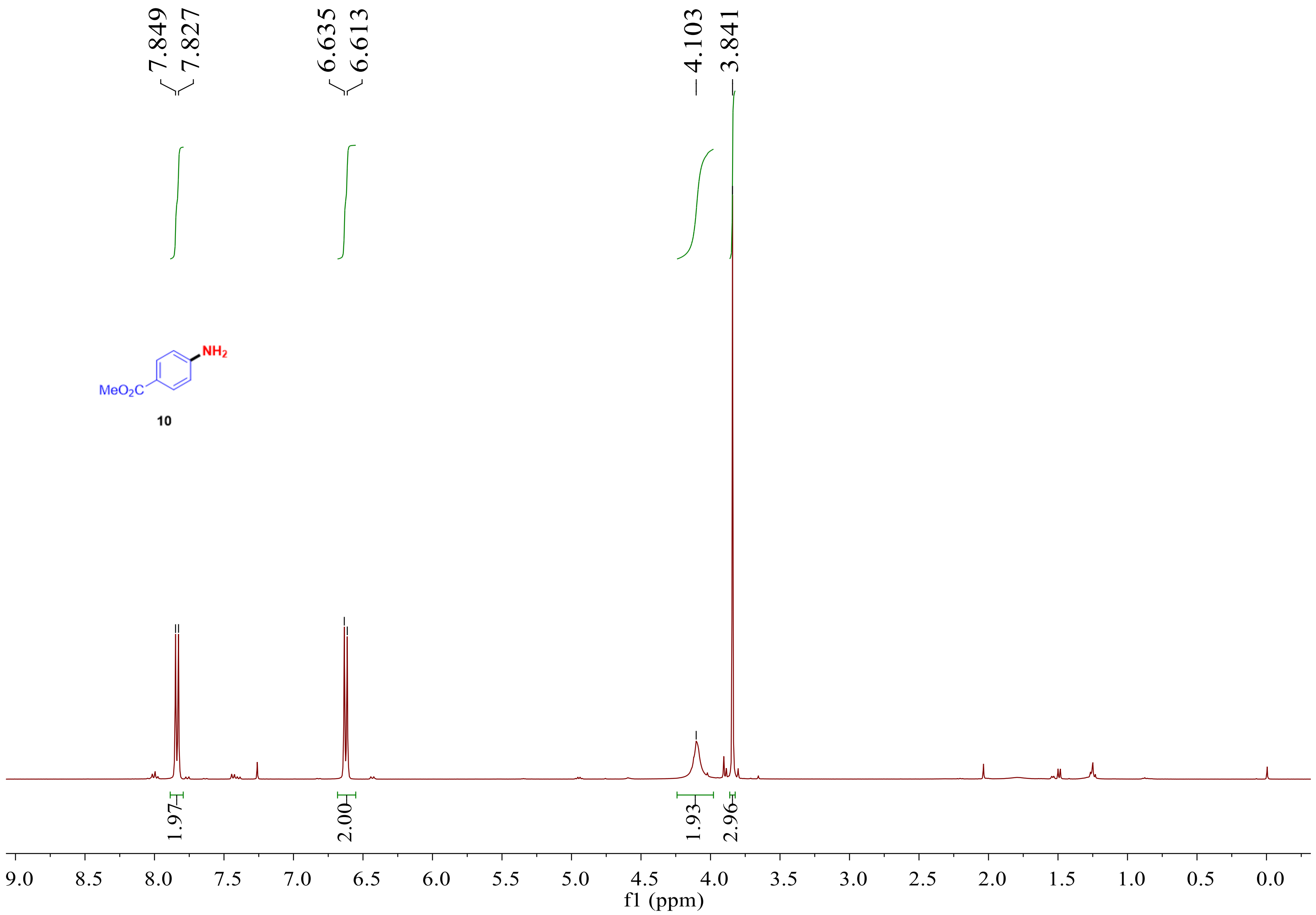
7.849
7.827
6.635
6.613
4.103
3.841
NH2
MeO2C
10
1.97
2.00
1.93
2.96
9.0
8.5
8.0
7.5
7.0
6.5
6.0
5.5
5.0
4.5
4.0
3.5
3.0
2.5
2.0
1.5
1.0
0.5
0.0
f1 (ppm)

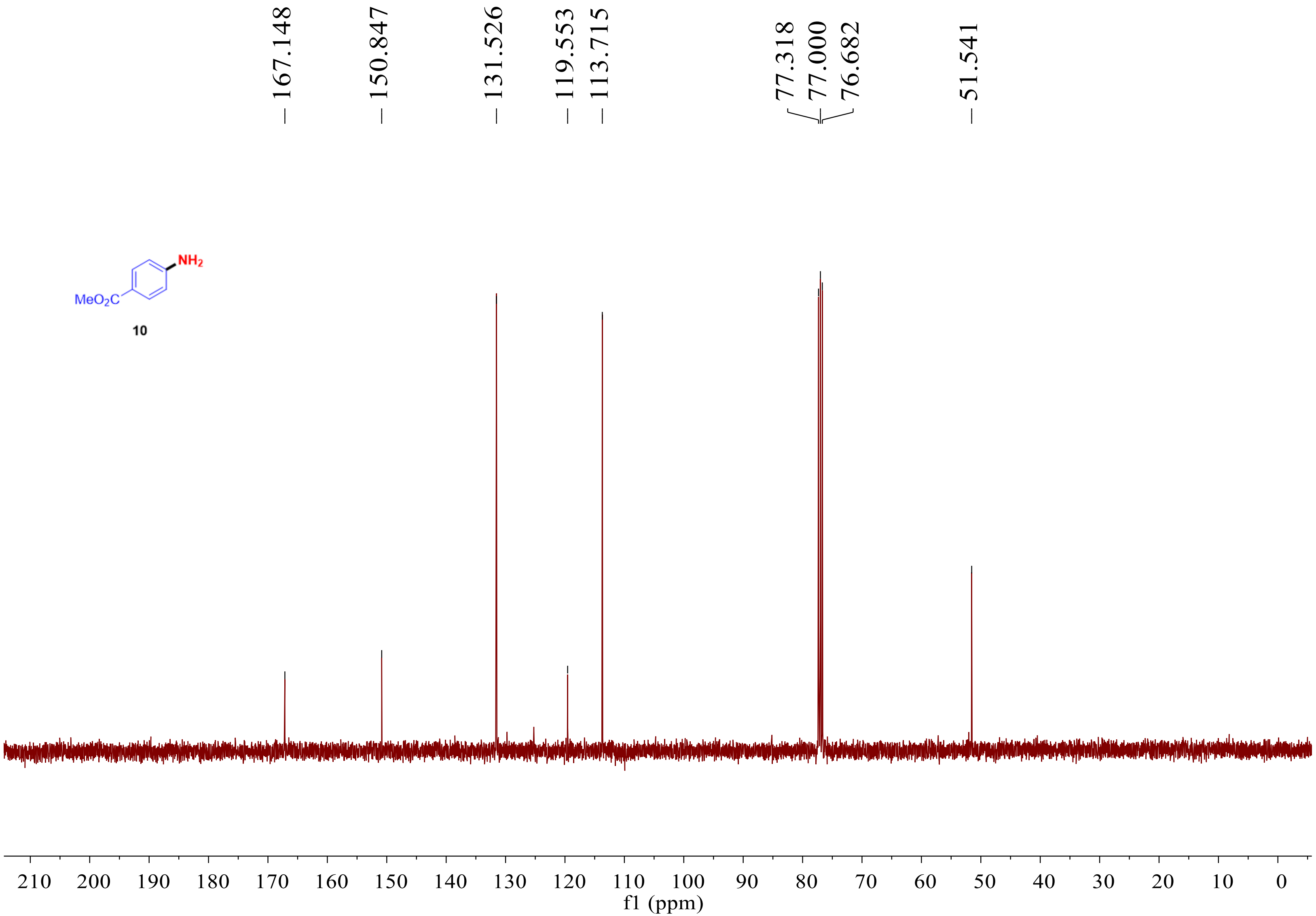
167.148
150.847
131.526
119.553
113.715
77.318
77.000
76.682
51.541
NH2
MeO2C
10
210 200 190 180 170 160 150 140 130 120 110 100 90 80 70 60 50 40 30 20 10 0
f1 (ppm)

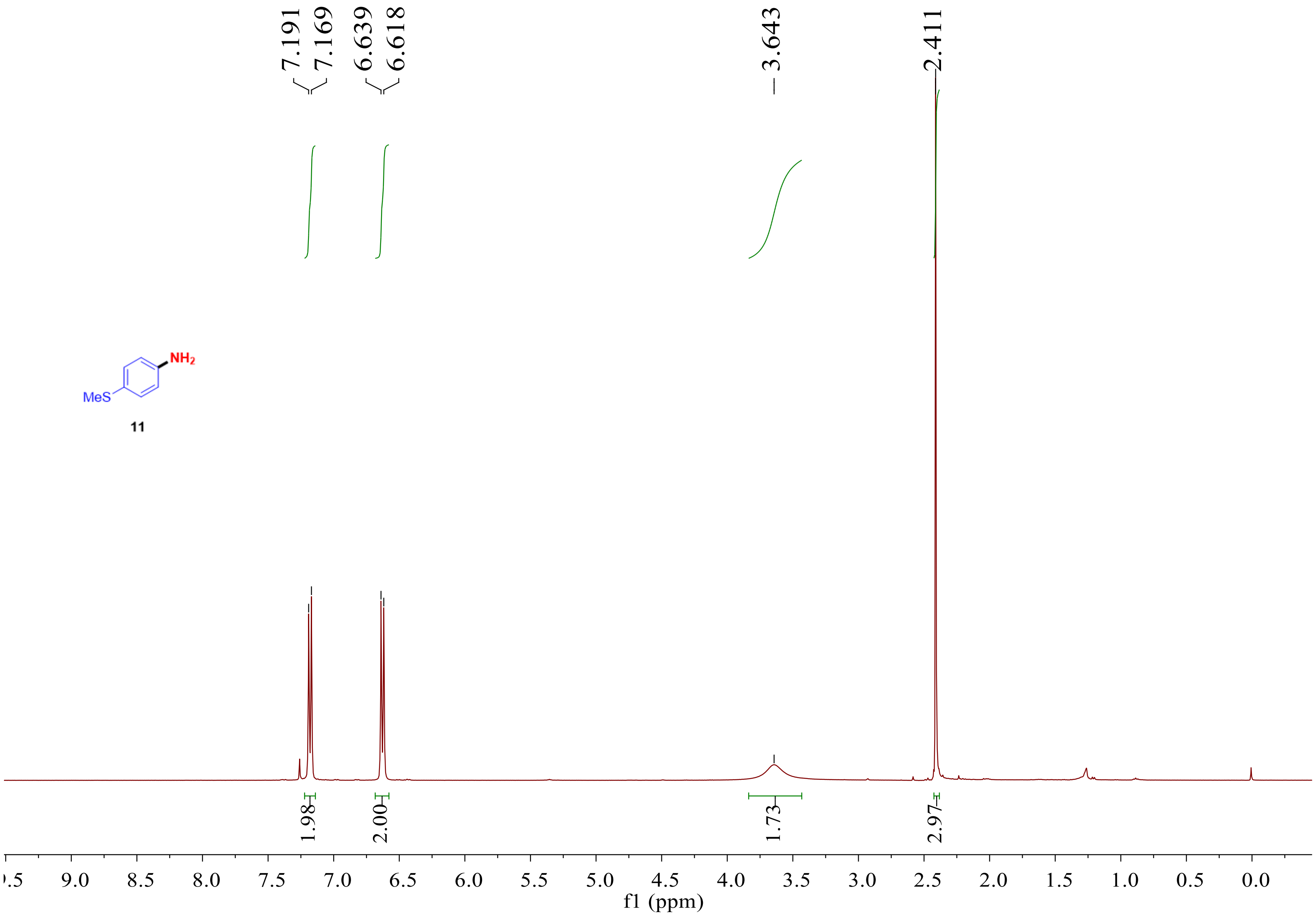
7.191
7.169
6.639
6.618
3.643
2.411
MeS
NH2
11
1.98
2.00
1.73
2.97
9.0
8.5
8.0
7.5
7.0
6.5
6.0
5.5
5.0
4.5
4.0
3.5
3.0
2.5
2.0
1.5
1.0
0.5
0.0
f1 (ppm)

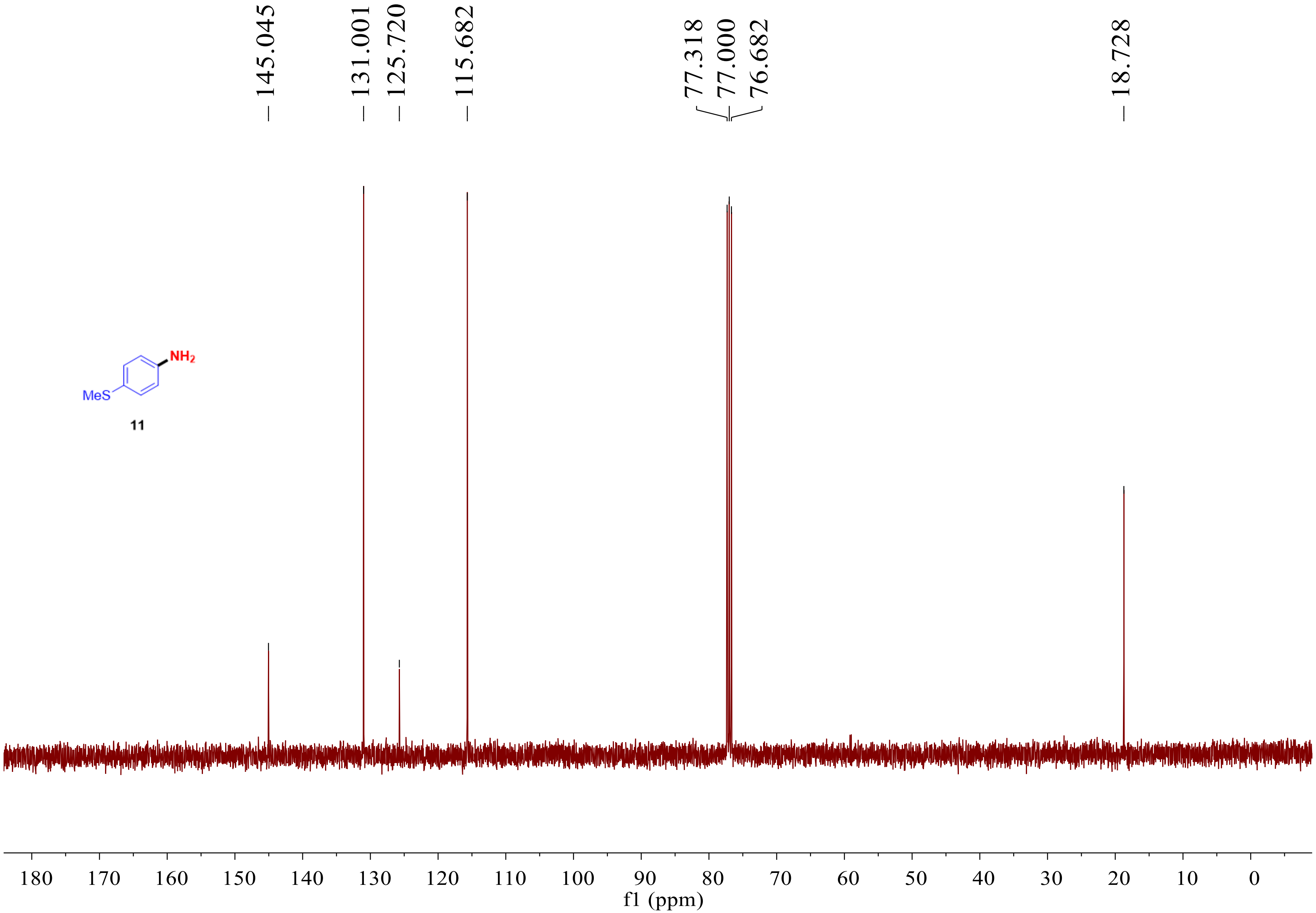

145.045
131.001
125.720
115.682
77.318
77.000
76.682
18.728
NH2
MeS
11
180
170
160
150
140
130
120
110
100
90
80
70
60
50
40
30
20
10
0
f1 (ppm)

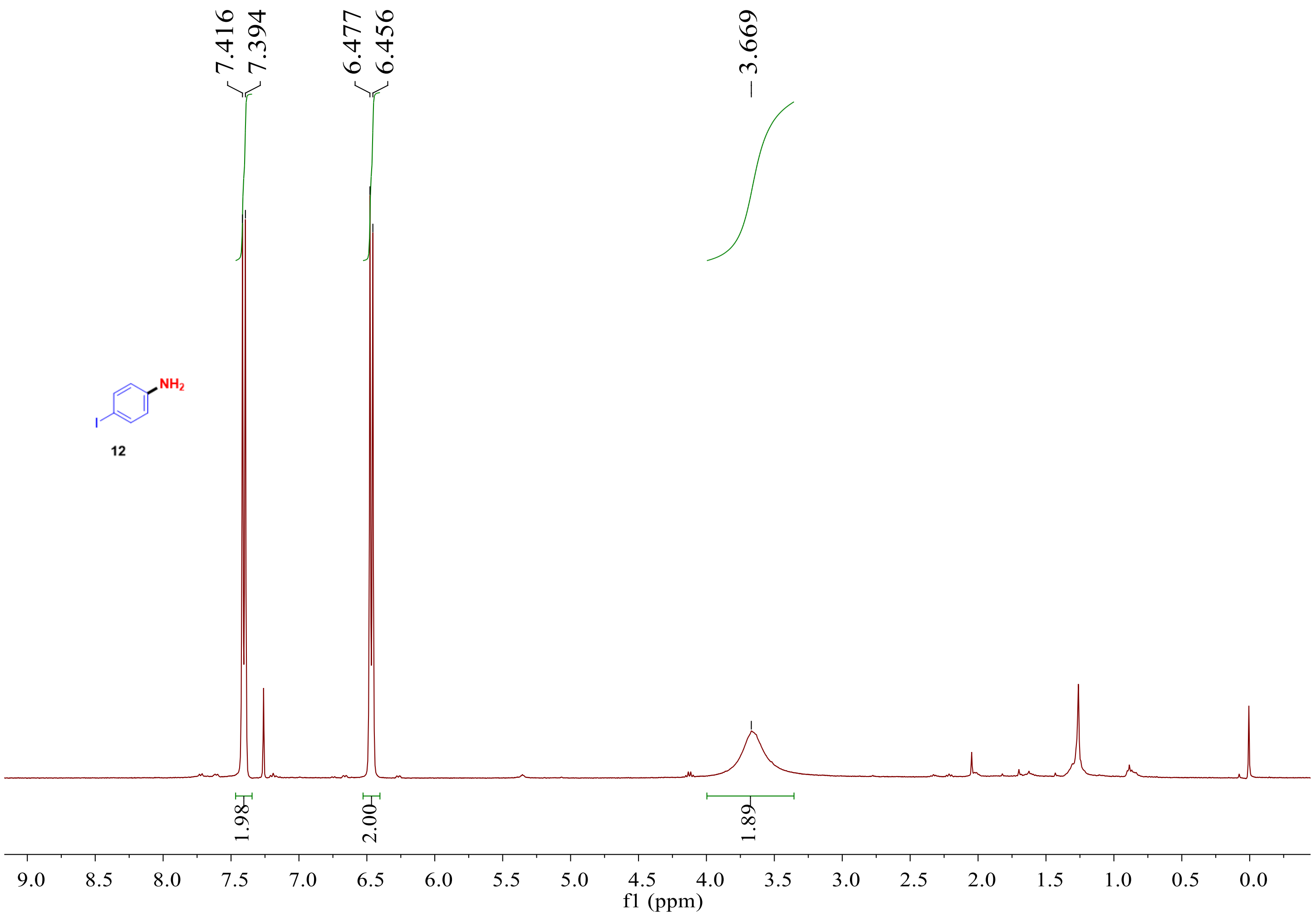
7.416
7.394
6.477
6.456
3.669
NH2
I
12
1.98
2.00
1.89
9.0 8.5 8.0 7.5 7.0 6.5 6.0 5.5 5.0 4.5 4.0 3.5 3.0 2.5 2.0 1.5 1.0 0.5 0.0
f1 (ppm)

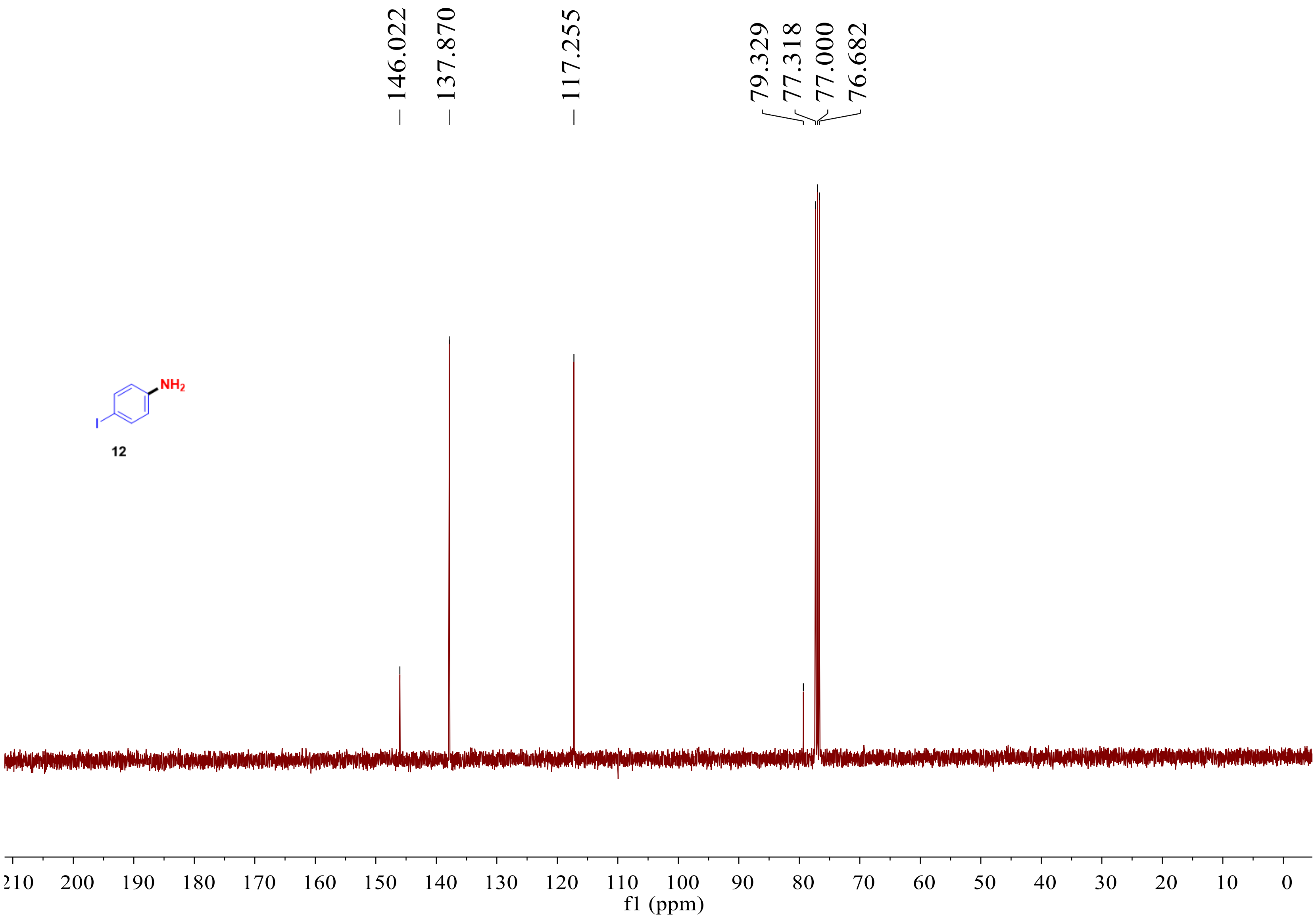
146.022
137.870
117.255
79.329
77.318
77.000
76.682
NH2
I
12
210 200 190 180 170 160 150 140 130 120 110 100 90 80 70 60 50 40 30 20 10 0
f1 (ppm)

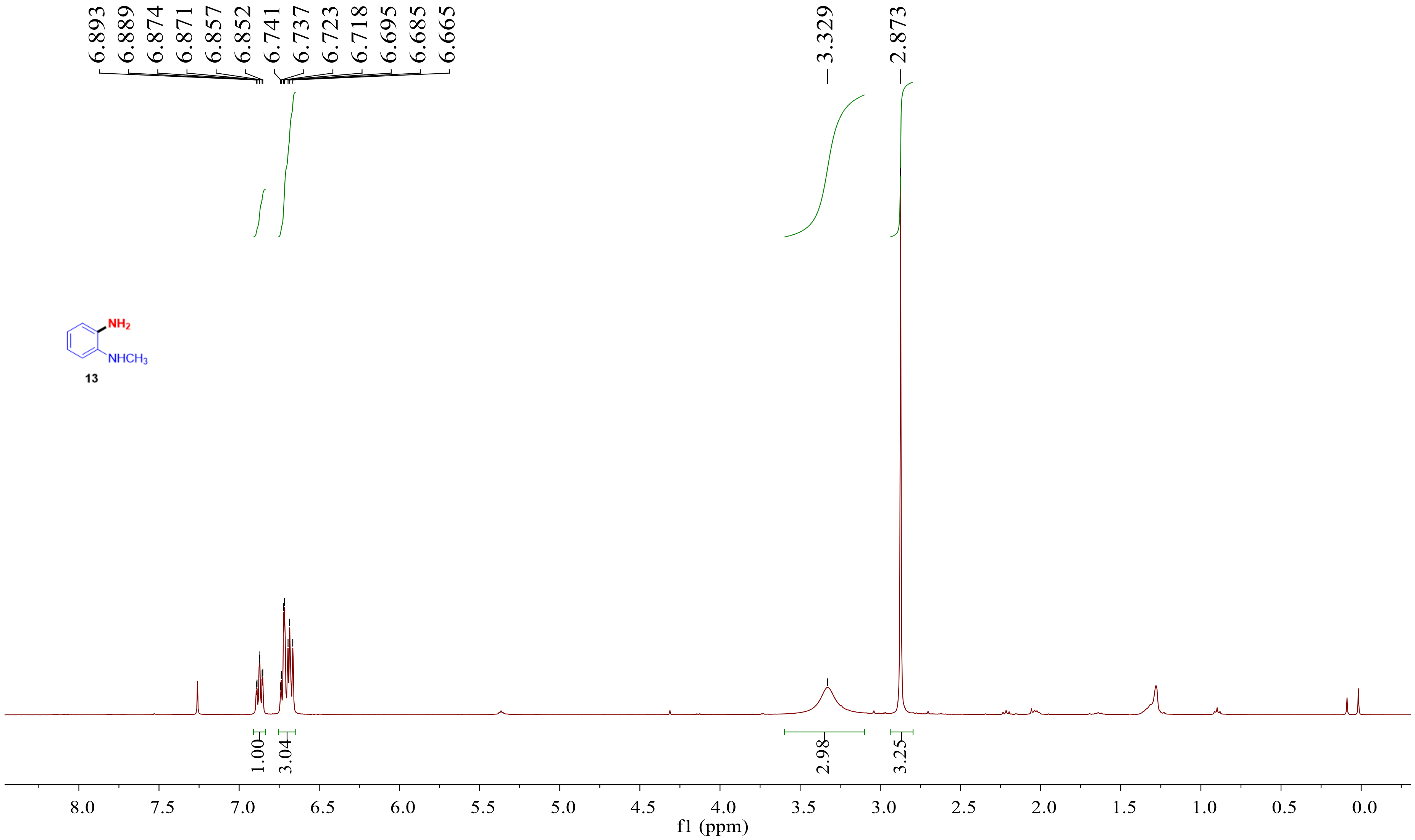
6.893
6.889
6.874
6.871
6.857
6.852
6.741
6.737
6.723
6.718
6.695
6.685
6.665
3.329
2.873
NH2
NHCH3
13
1.00
3.04
2.98
3.25
8.0
7.5
7.0
6.5
6.0
5.5
5.0
4.5
4.0
3.5
3.0
2.5
2.0
1.5
1.0
0.5
0.0
f1 (ppm)

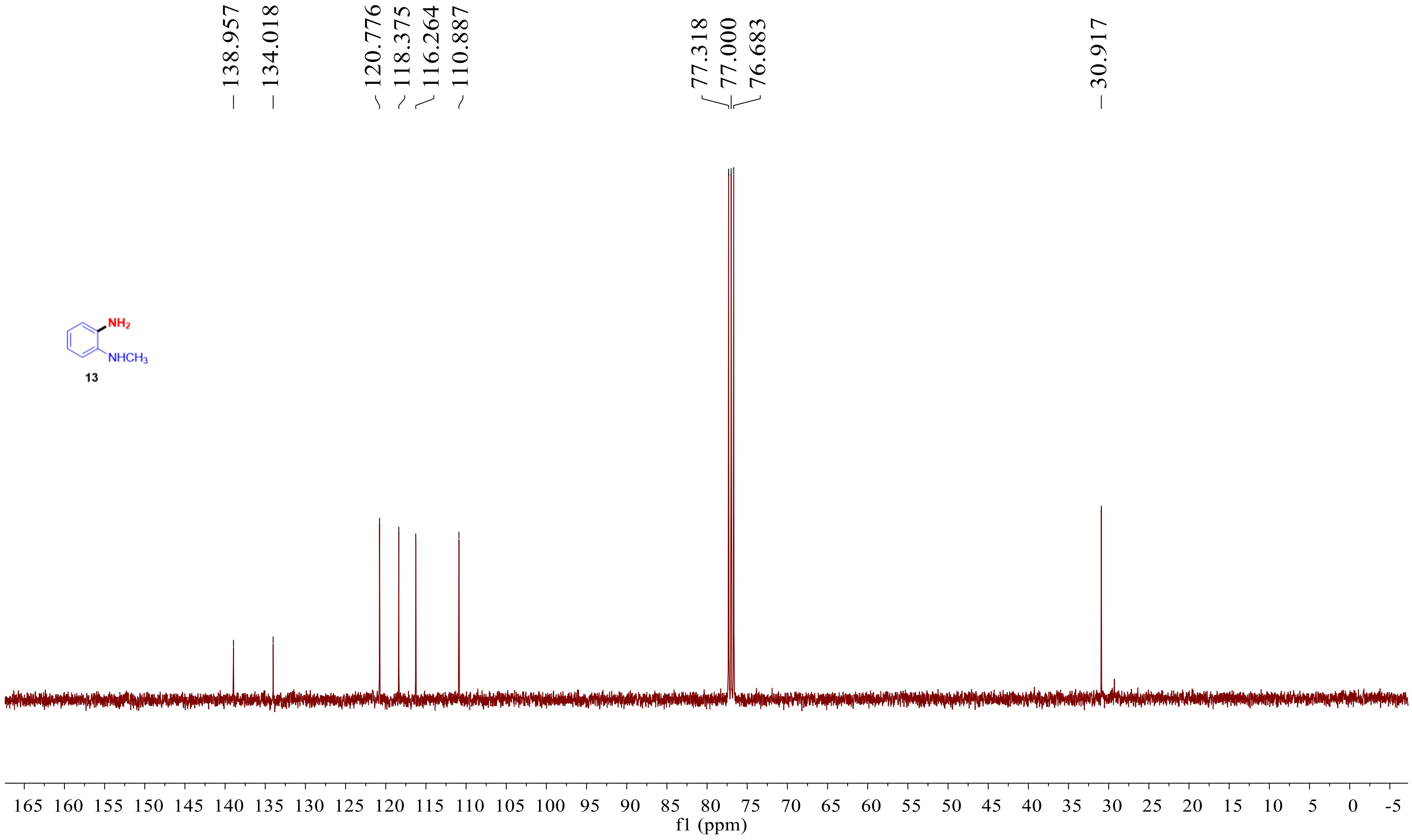
138.957
134.018
120.776
118.375
116.264
110.887
77.318
77.000
76.683
30.917
NH2
NHCH3
13
165 160 155 150 145 140 135 130 125 120 115 110 105 100 95 90 85 80 75 70 65 60 55 50 45 40 35 30 25 20 15 10 5 0 -5
f1 (ppm)

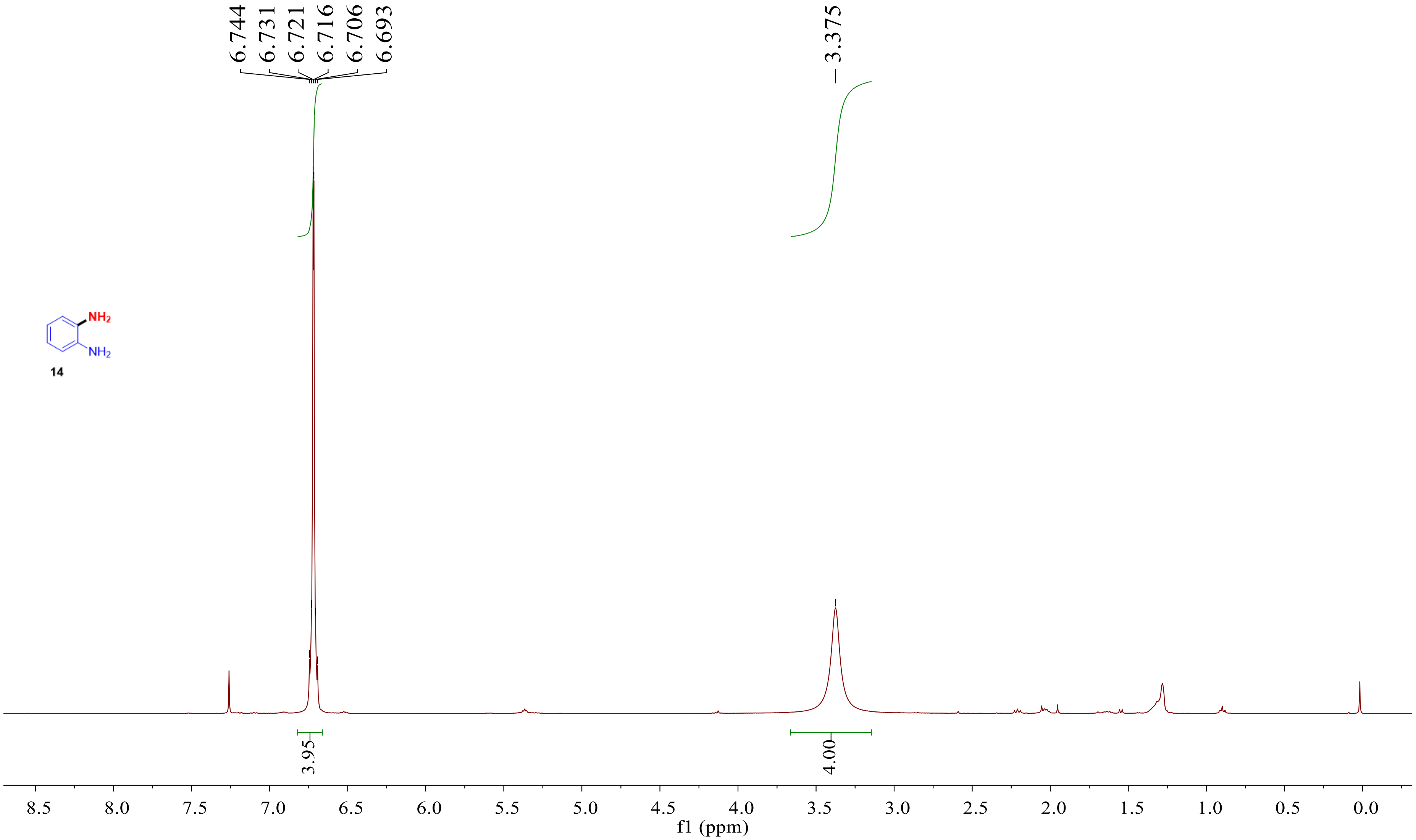

6.744
6.731
6.721
6.716
6.706
6.693
3.375
NH2
NH2
14
3.95
4.00
8.5
8.0
7.5
7.0
6.5
6.0
5.5
5.0
4.5
4.0
3.5
3.0
2.5
2.0
1.5
1.0
0.5
0.0
f1 (ppm)

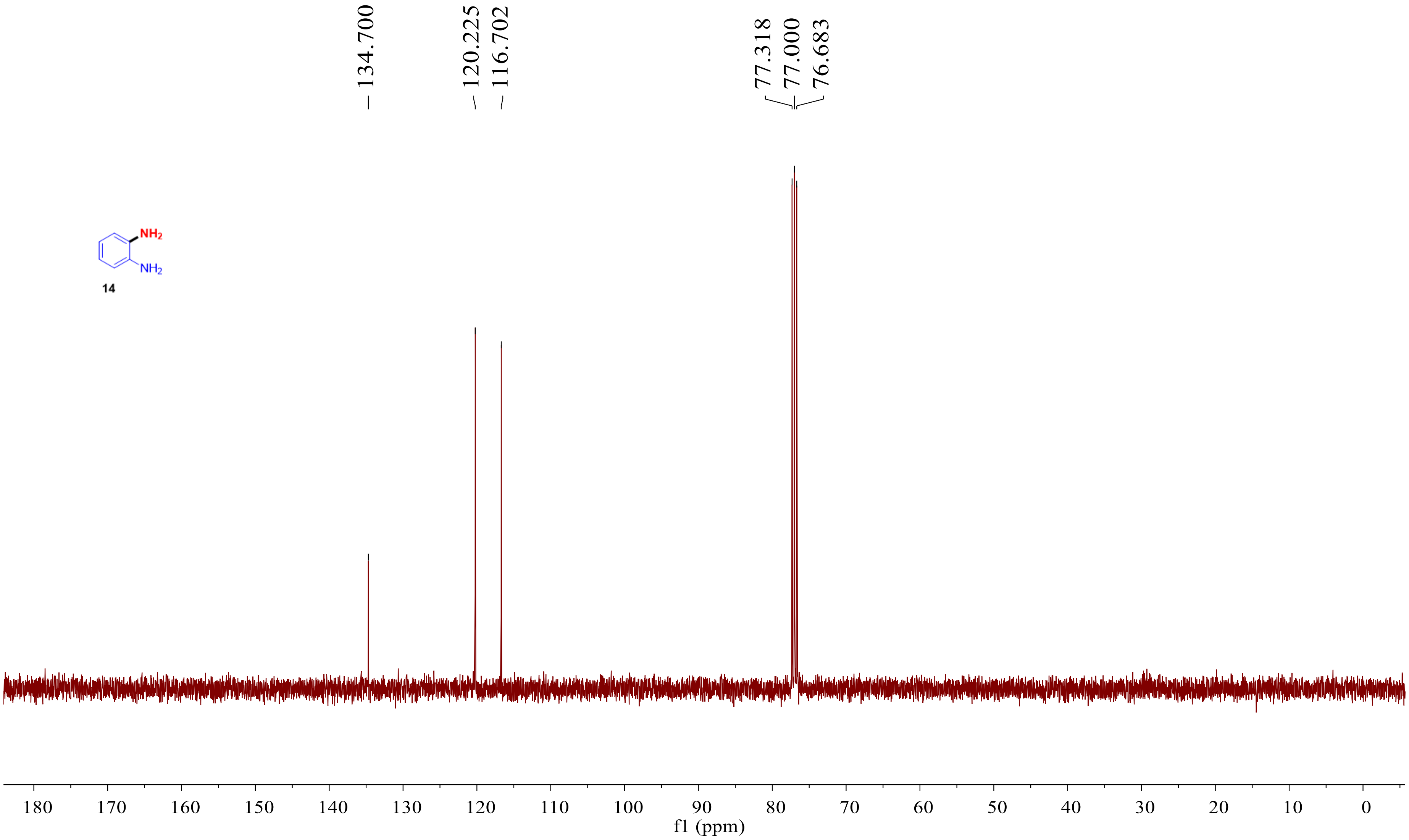

134.700
120.225
116.702
77.318
77.000
76.683
NH2
NH2
14
180
170
160
150
140
130
120
110
100
90
80
70
60
50
40
30
20
10
0
f1 (ppm)

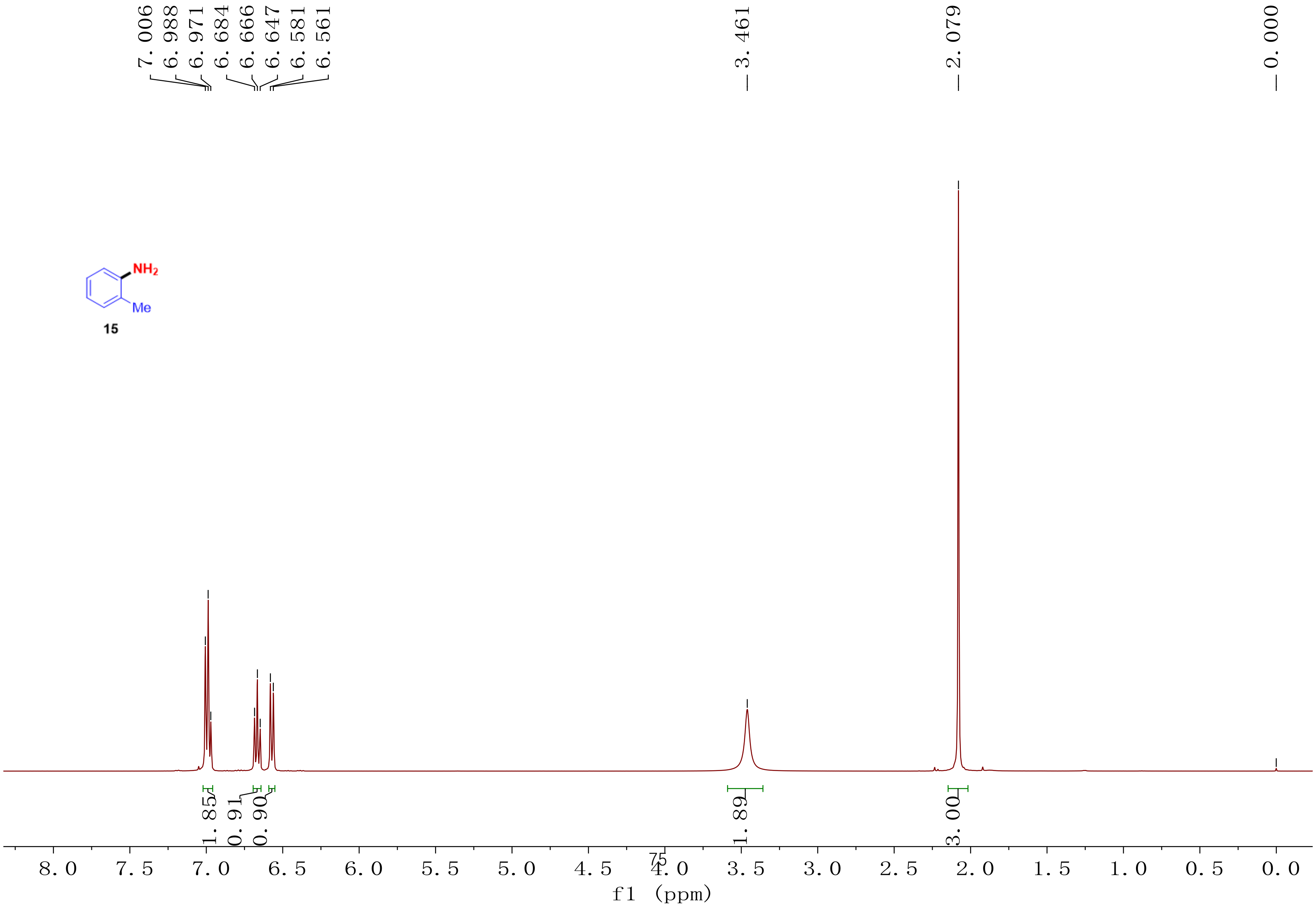
NH2
Me
15
7.006
6.988
6.971
6.684
6.666
6.647
6.581
6.561
3.461
2.079
0.000
1.85
0.91
0.90
1.89
3.00
8.0
7.5
7.0
6.5
6.0
5.5
5.0
4.5
4.0
3.5
3.0
2.5
2.0
1.5
1.0
0.5
0.0
f1 (ppm)

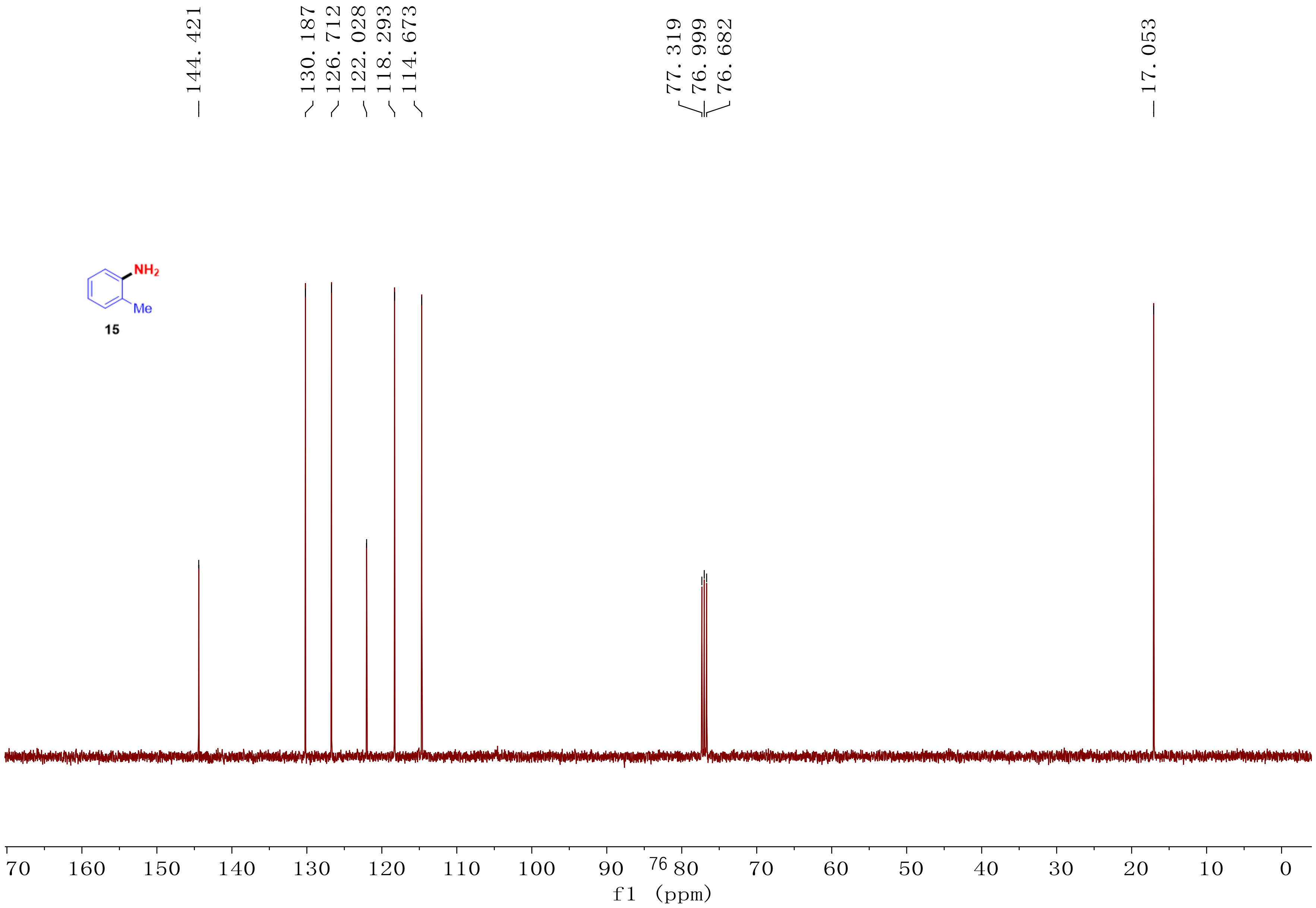
144.421
130.187
126.712
122.028
118.293
114.673
77.319
76.999
76.682
17.053
NH2
Me
15
70
160
150
140
130
120
110
100
90
80
70
60
50
40
30
20
10
0
f1 (ppm)

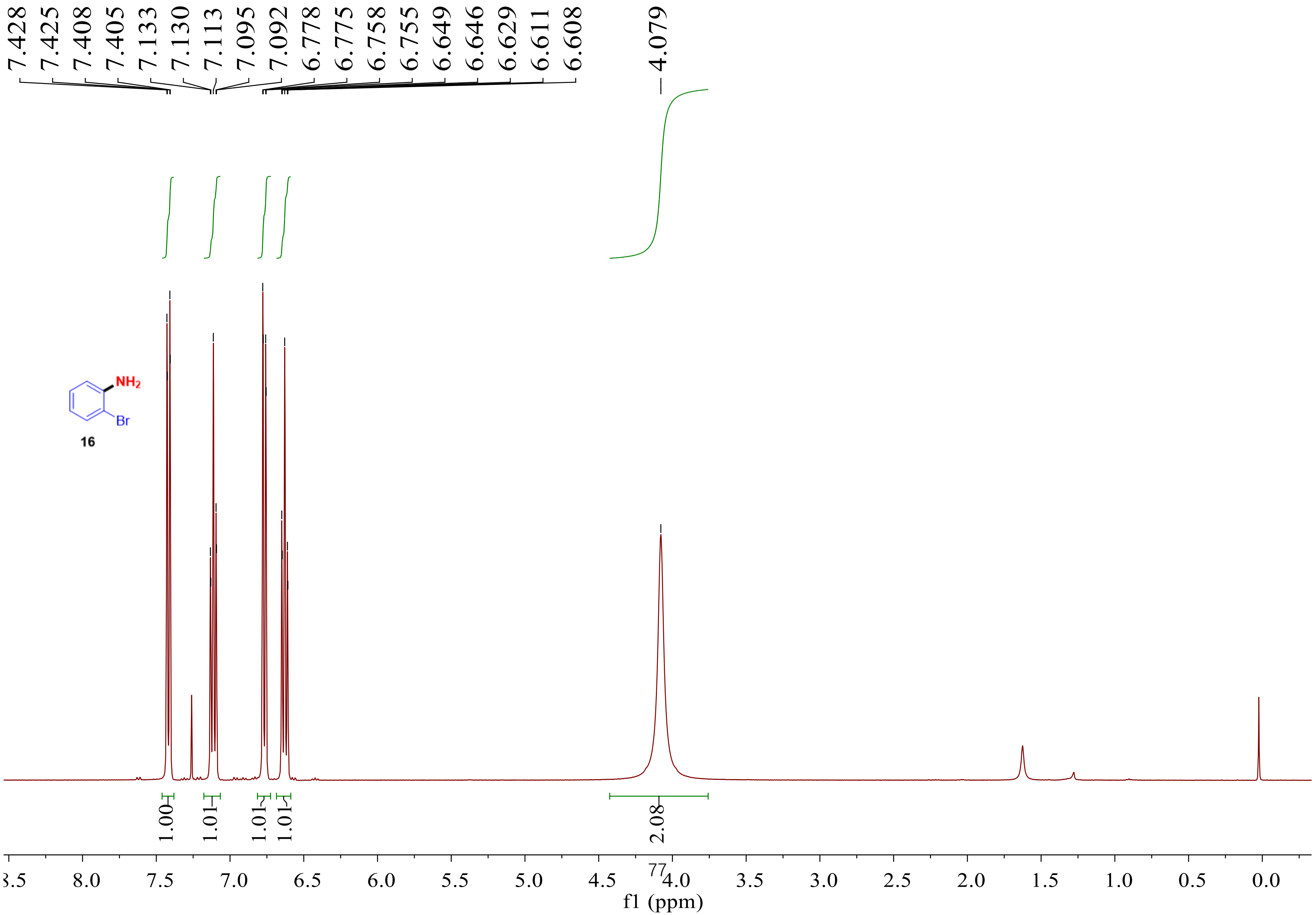

7.428
7.425
7.408
7.405
7.133
7.130
7.113
7.095
7.092
6.778
6.775
6.758
6.755
6.649
6.646
6.629
6.611
6.608
4.079
NH2
Br
16
1.00
1.01
1.01
1.01
2.08
8.5
8.0
7.5
7.0
6.5
6.0
5.5
5.0
4.5
4.0
3.5
3.0
2.5
2.0
1.5
1.0
0.5
0.0
f1 (ppm)

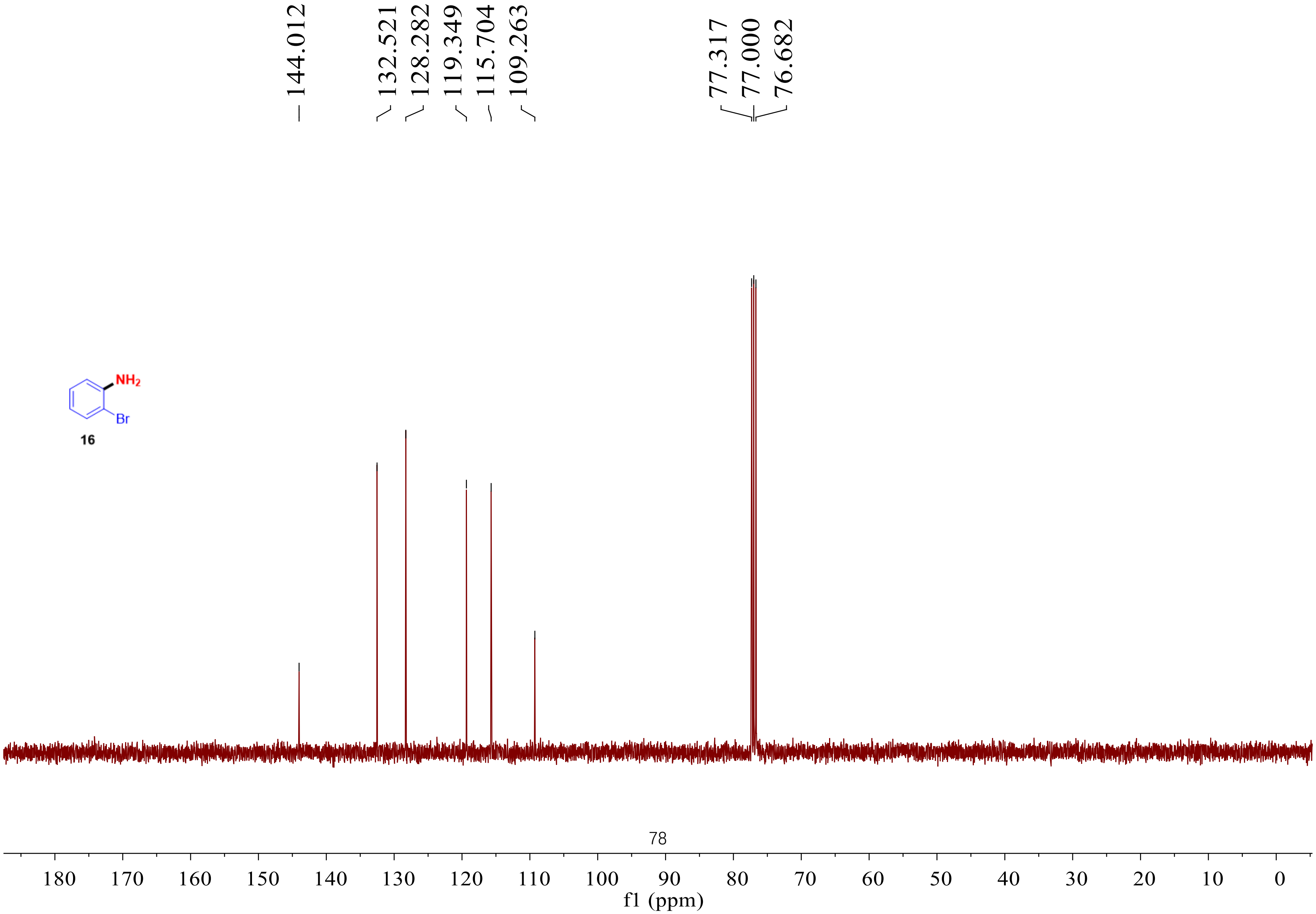

144.012
132.521
128.282
119.349
115.704
109.263
77.317
77.000
76.682
NH2
Br
16
180 170 160 150 140 130 120 110 100 90 80 70 60 50 40 30 20 10 0
f1 (ppm)

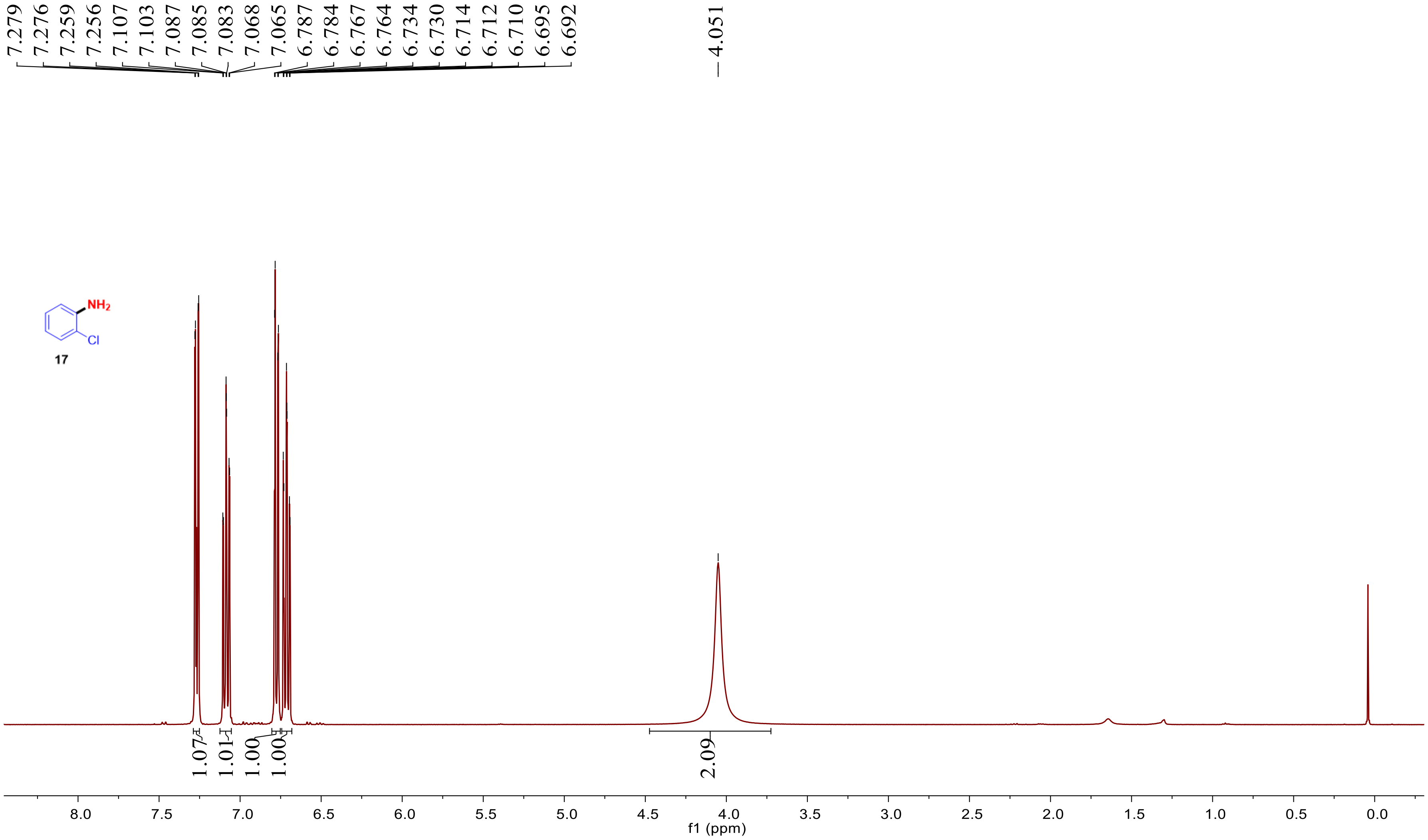
7.279
7.276
7.259
7.256
7.107
7.103
7.087
7.085
7.083
7.068
7.065
6.787
6.784
6.767
6.764
6.734
6.730
6.714
6.712
6.710
6.695
6.692
4.051
NH2
Cl
17
1.07
1.01
1.00
1.00
2.09
8.0
7.5
7.0
6.5
6.0
5.5
5.0
4.5
4.0
3.5
3.0
2.5
2.0
1.5
1.0
0.5
0.0
f1 (ppm)

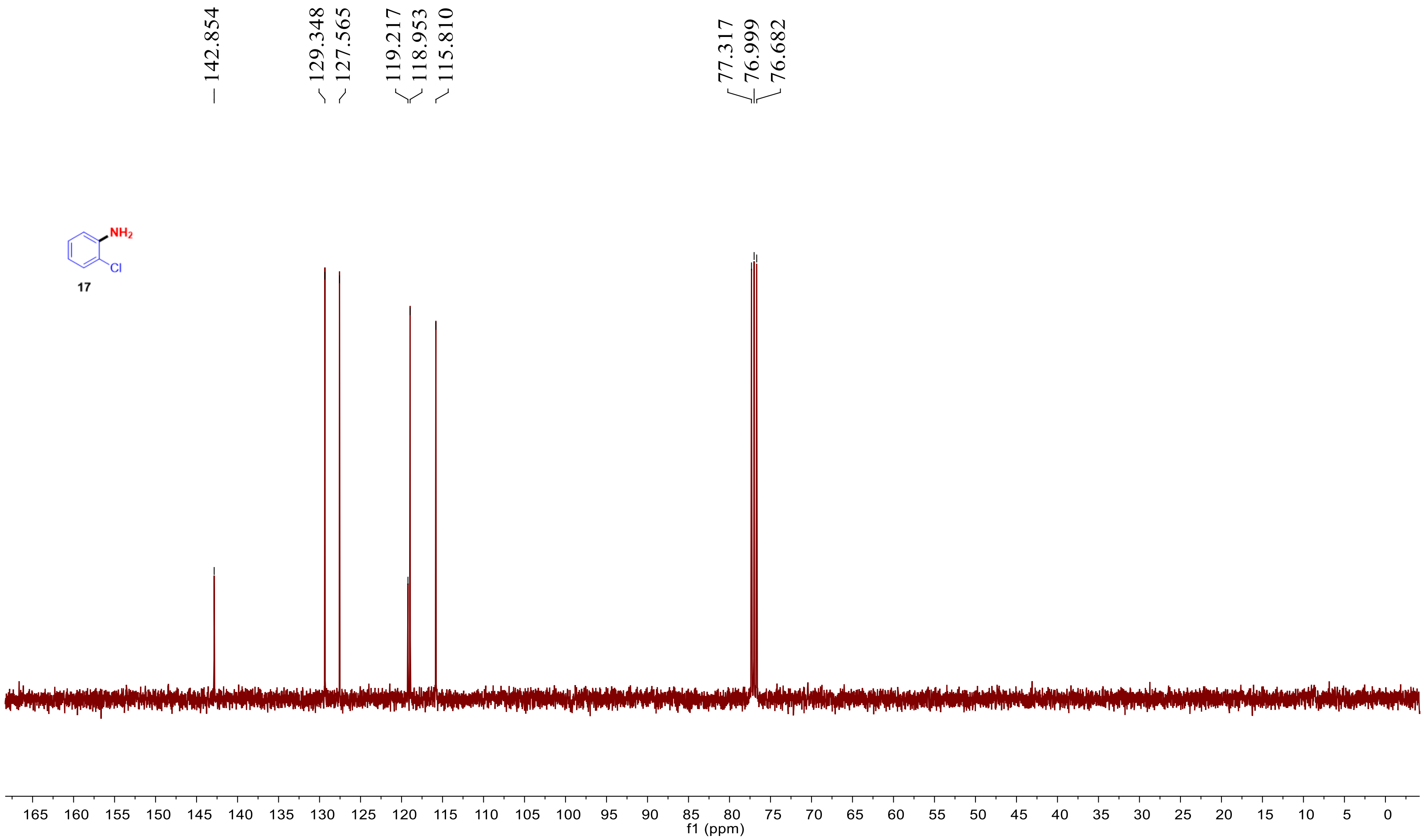

142.854
129.348
127.565
119.217
118.953
115.810
77.317
76.999
76.682
NH2
Cl
17
165 160 155 150 145 140 135 130 125 120 115 110 105 100 95 90 85 80 75 70 65 60 55 50 45 40 35 30 25 20 15 10 5 0
f1 (ppm)

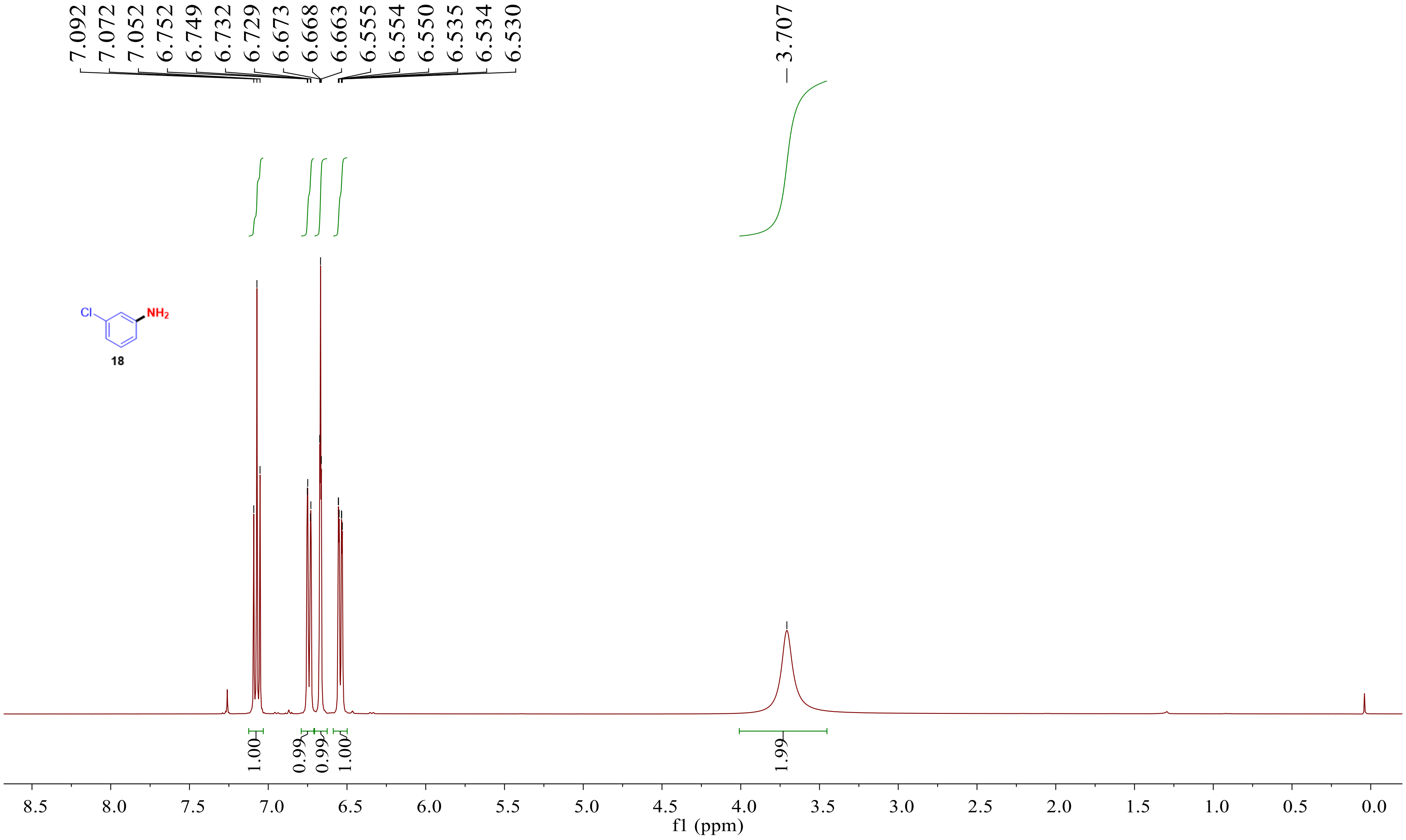
7.092
7.072
7.052
6.752
6.749
6.732
6.729
6.673
6.668
6.663
6.555
6.554
6.550
6.535
6.534
6.530
3.707
Cl
NH2
1.00
0.99
0.99
1.00
1.99
8.5
8.0
7.5
7.0
6.5
6.0
5.5
5.0
4.5
4.0
3.5
3.0
2.5
2.0
1.5
1.0
0.5
0.0
f1 (ppm)

18

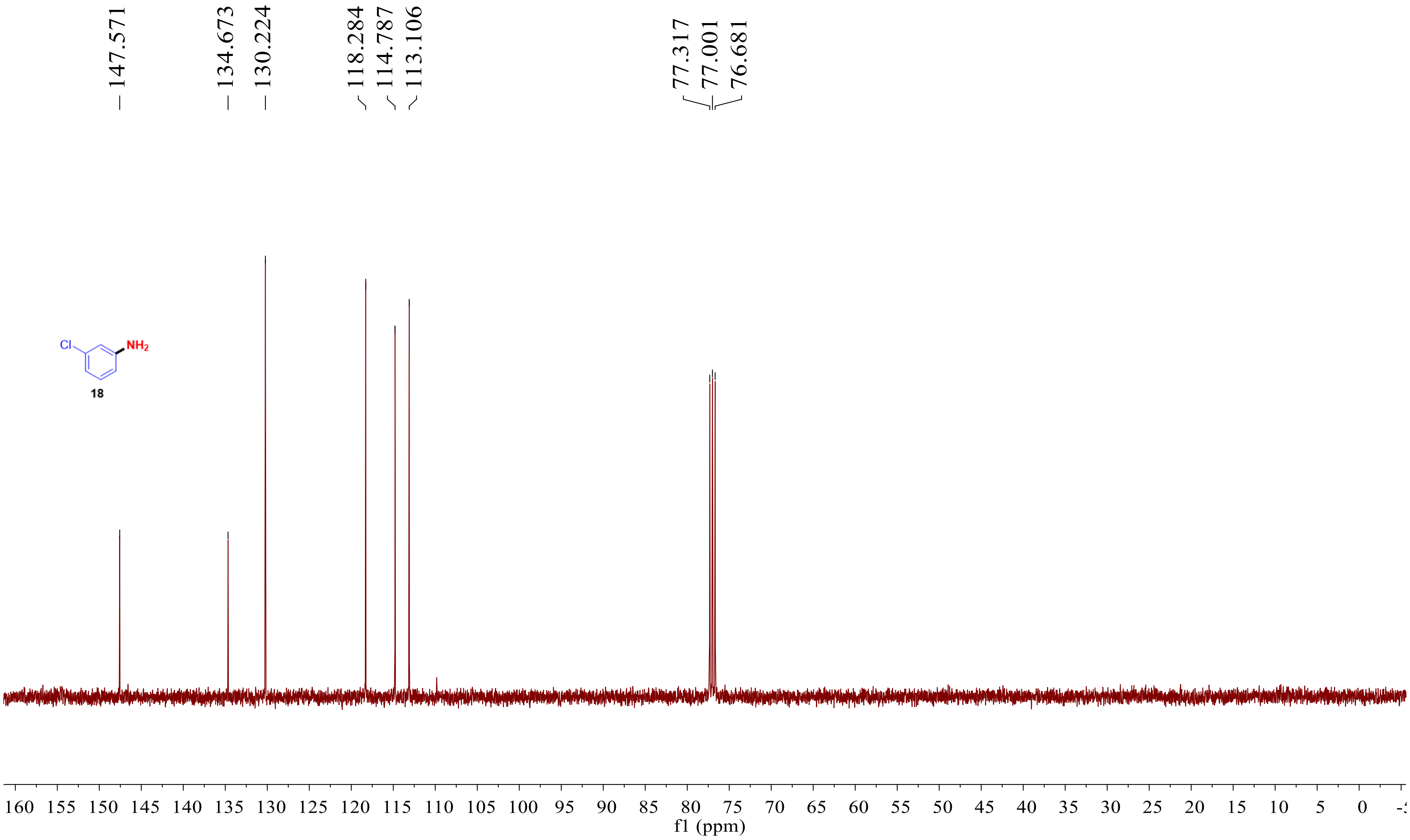
147.571
134.673
130.224
118.284
114.787
113.106
77.317
77.001
76.681
Cl
NH2
18
160 155 150 145 140 135 130 125 120 115 110 105 100 95 90 85 80 75 70 65 60 55 50 45 40 35 30 25 20 15 10 5 0
f1 (ppm)

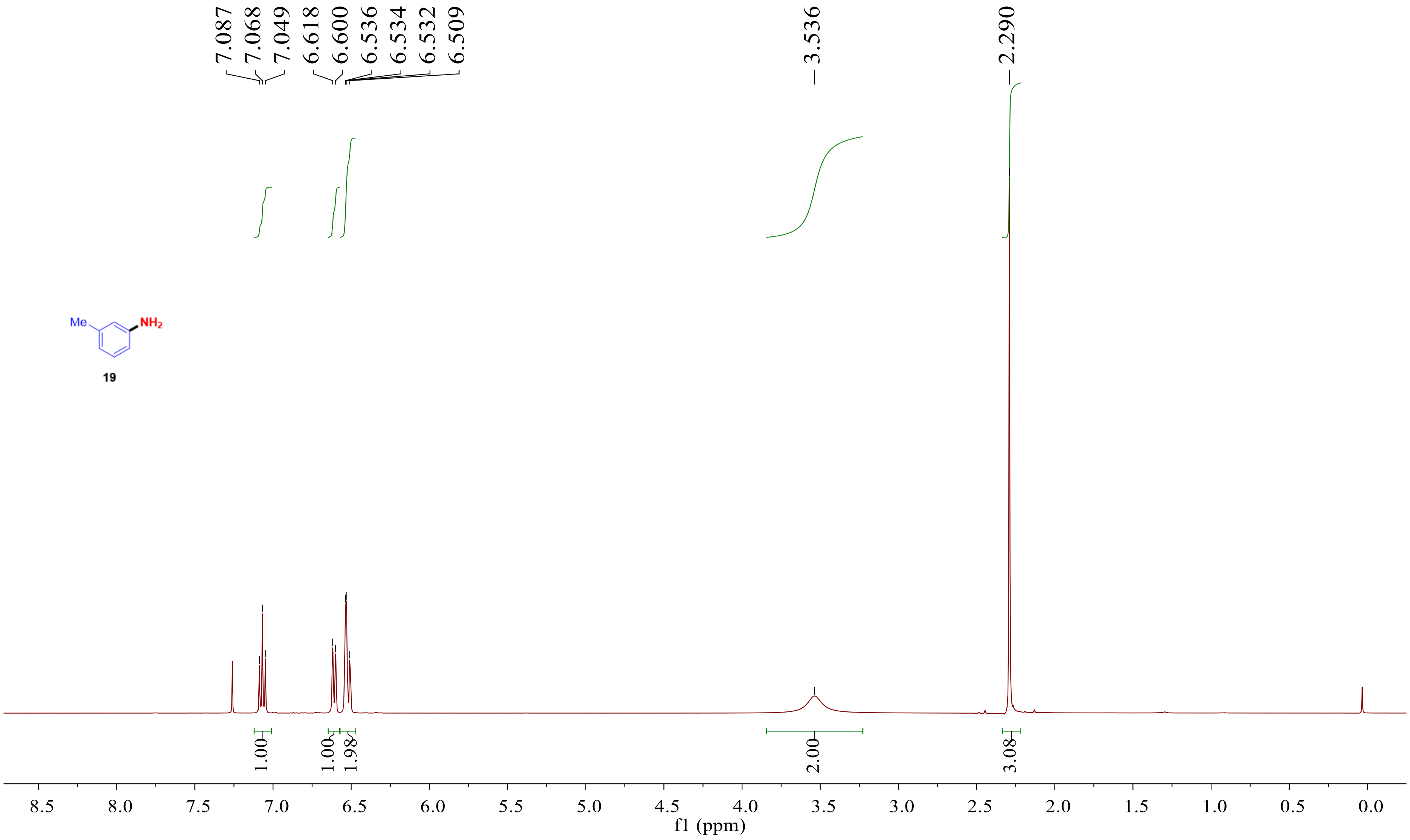
7.087
7.068
7.049
6.618
6.600
6.536
6.534
6.532
6.509
3.536
2.290
Me
NH2
19
1.00
1.00
1.98
2.00
3.08
8.5
8.0
7.5
7.0
6.5
6.0
5.5
5.0
4.5
4.0
3.5
3.0
2.5
2.0
1.5
1.0
0.5
0.0
f1 (ppm)

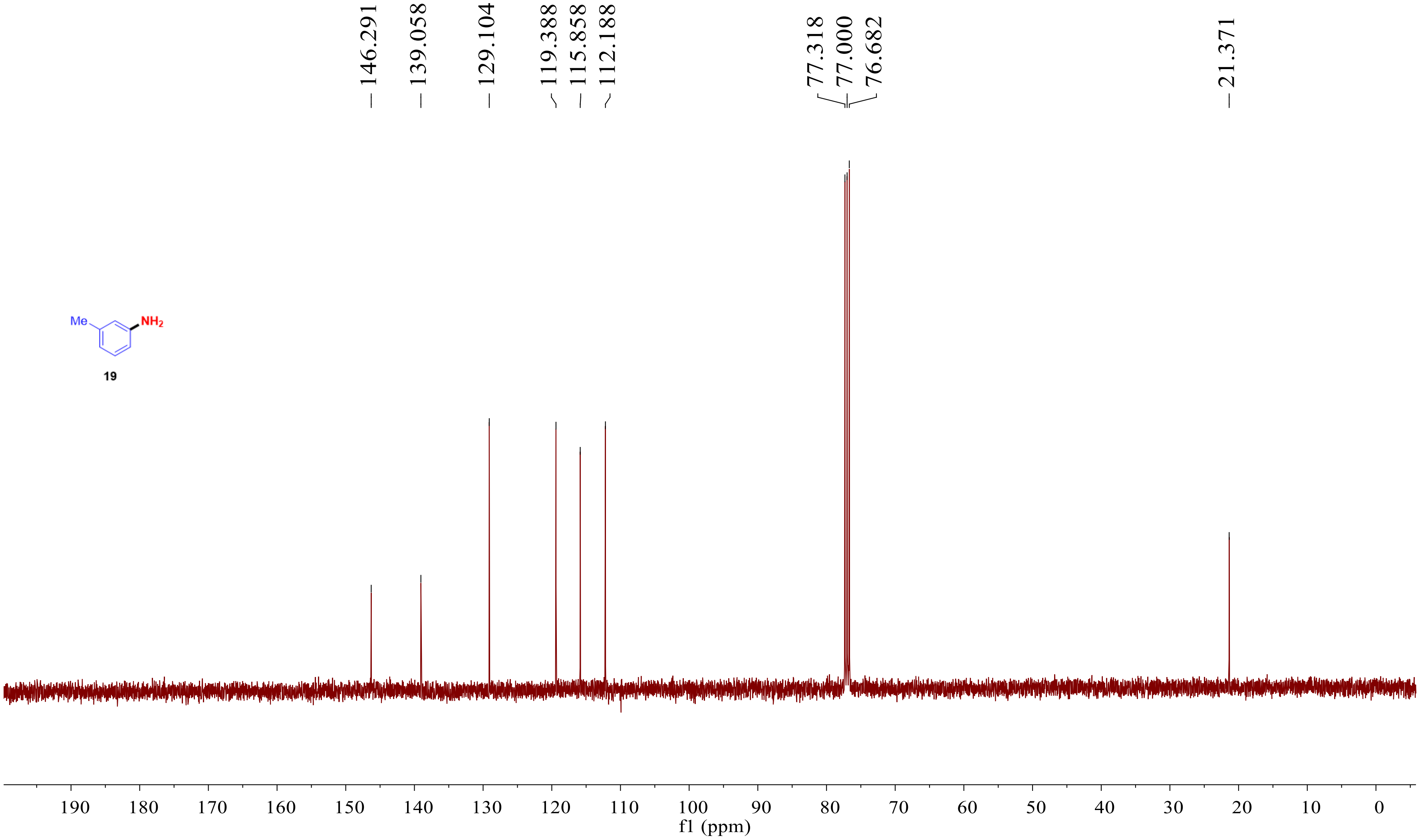
Me
NH2
19
146.291
139.058
129.104
119.388
115.858
112.188
77.318
77.000
76.682
21.371
190
180
170
160
150
140
130
120
110
100
90
80
70
60
50
40
30
20
10
0
f1 (ppm)

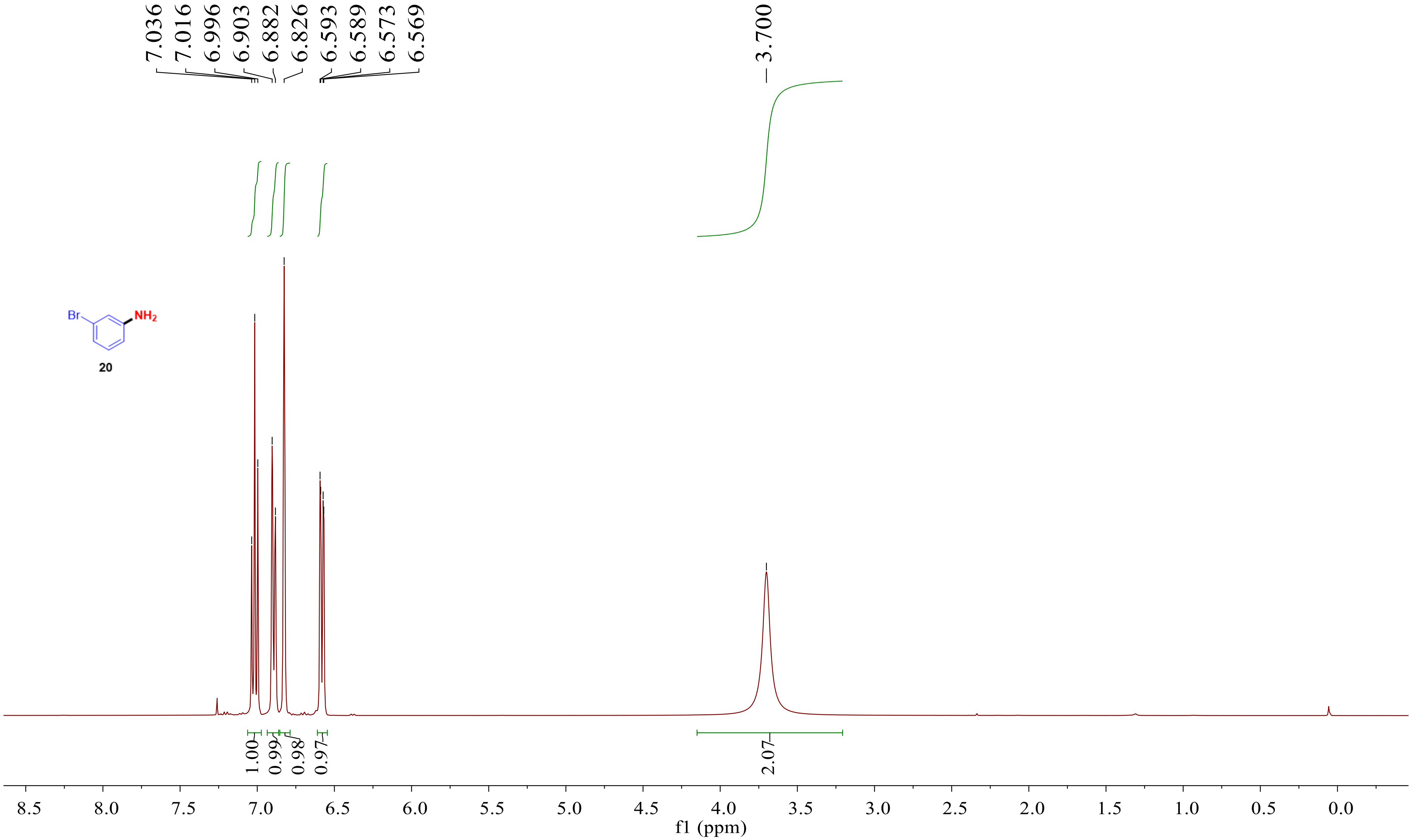
7.036
7.016
6.996
6.903
6.882
6.826
6.593
6.589
6.573
6.569
3.700
Br
NH2
20
1.00
0.99
0.98
0.97
2.07
8.5 8.0 7.5 7.0 6.5 6.0 5.5 5.0 4.5 4.0 3.5 3.0 2.5 2.0 1.5 1.0 0.5 0.0
f1 (ppm)

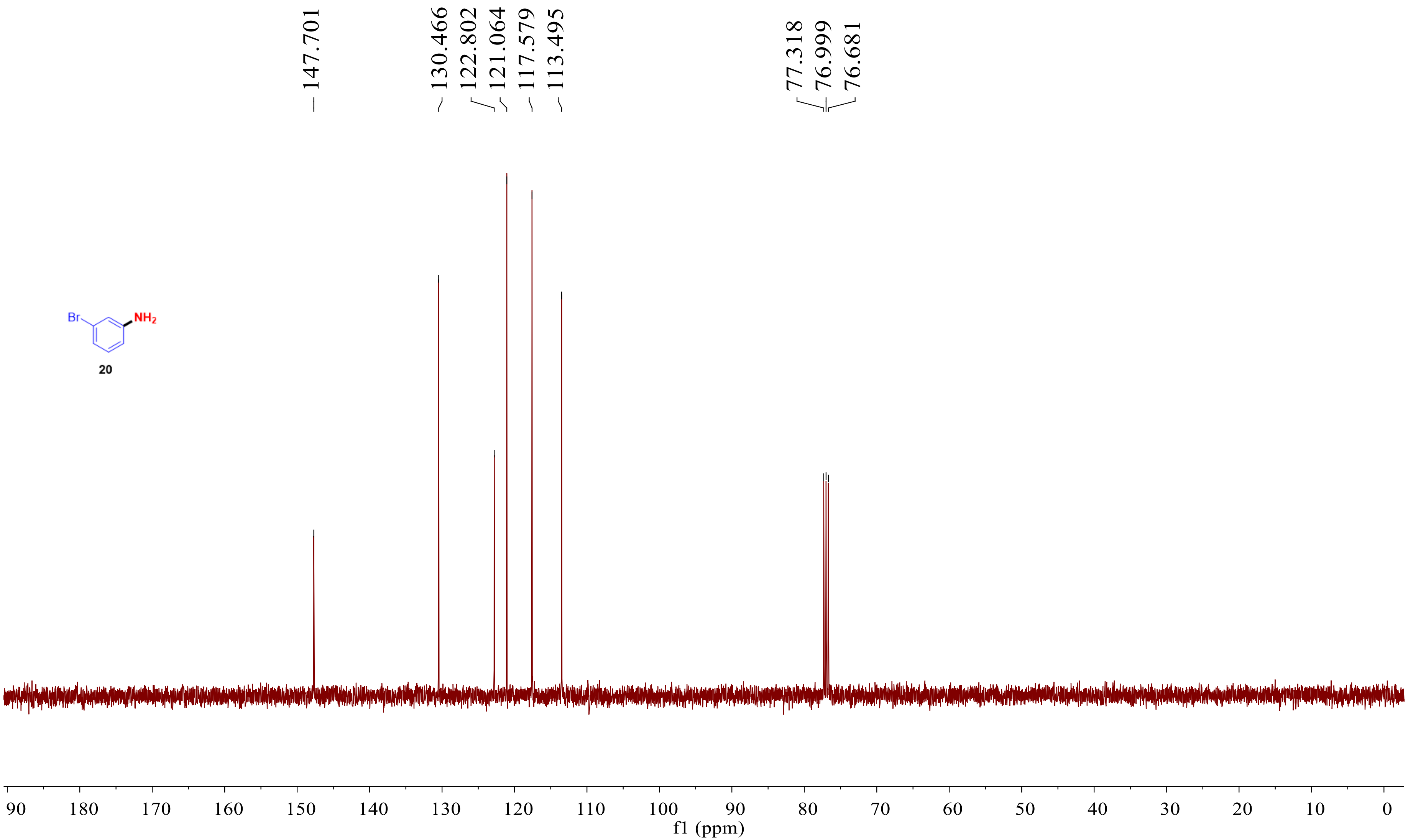
147.701
130.466
122.802
121.064
117.579
113.495
77.318
76.999
76.681
Br
NH2
20
90 180 170 160 150 140 130 120 110 100 90 80 70 60 50 40 30 20 10 0
f1 (ppm)

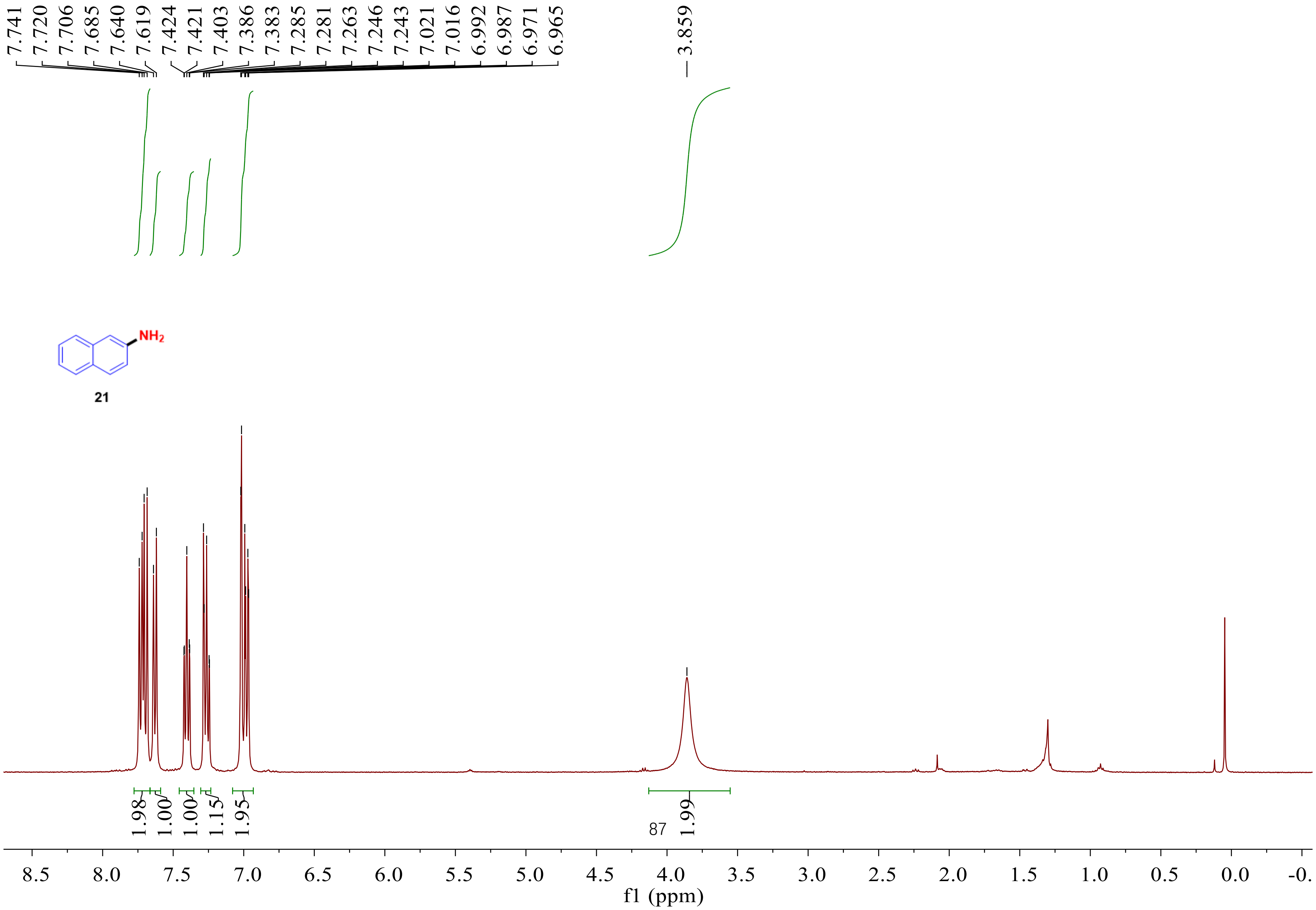

7.741
7.720
7.706
7.685
7.640
7.619
7.424
7.421
7.403
7.386
7.383
7.285
7.281
7.263
7.246
7.243
7.021
7.016
6.992
6.987
6.971
6.965
3.859
NH2
21
1.98
1.00
1.00
1.15
1.95
1.99
8.5
8.0
7.5
7.0
6.5
6.0
5.5
5.0
4.5
4.0
3.5
3.0
2.5
2.0
1.5
1.0
0.5
0.0
-0.
f1 (ppm)

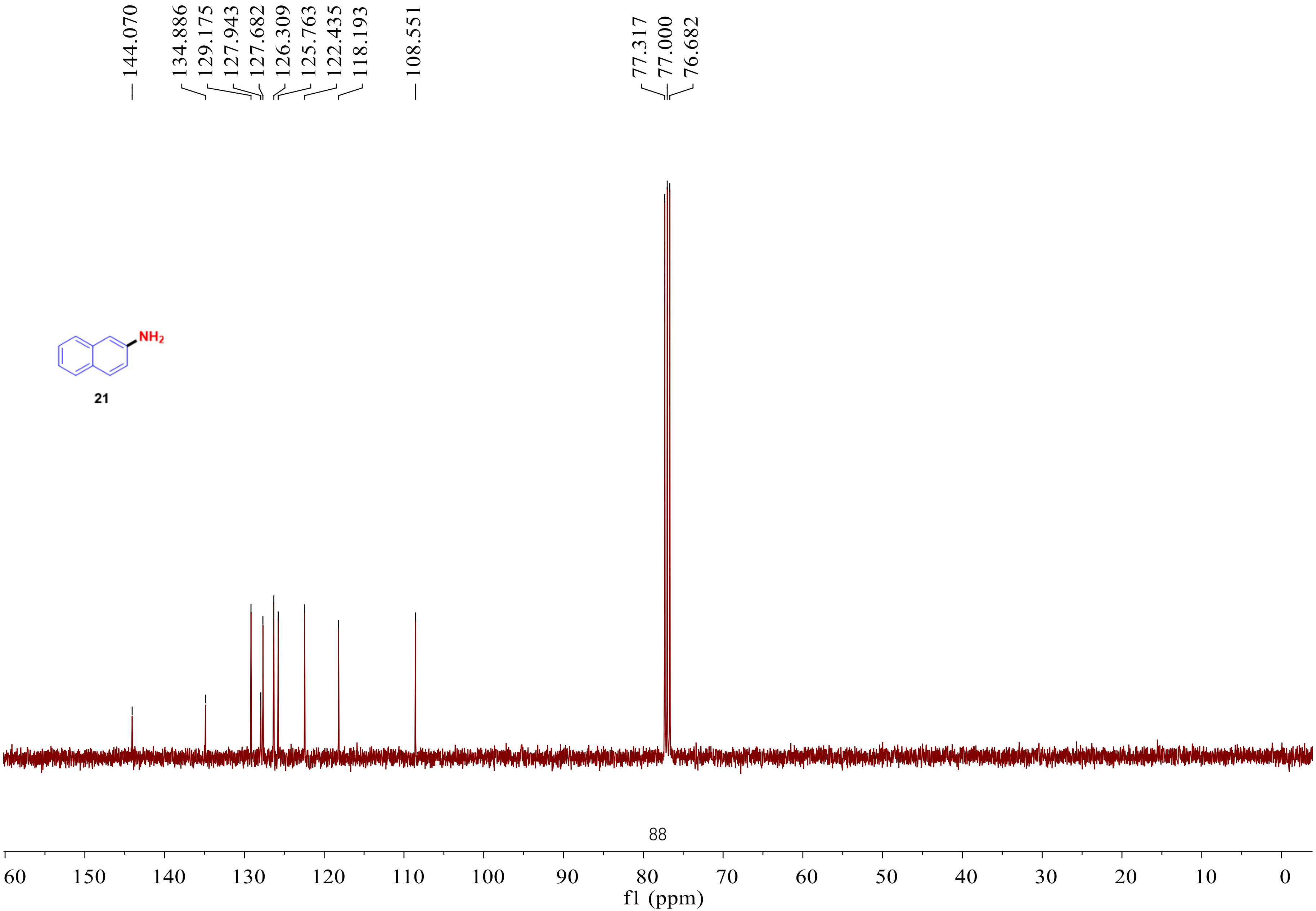

144.070
134.886
129.175
127.943
127.682
126.309
125.763
122.435
118.193
108.551
77.317
77.000
76.682
60
150
140
130
120
110
100
90
80
70
60
50
40
30
20
10
0
f1 (ppm)
NH2

21

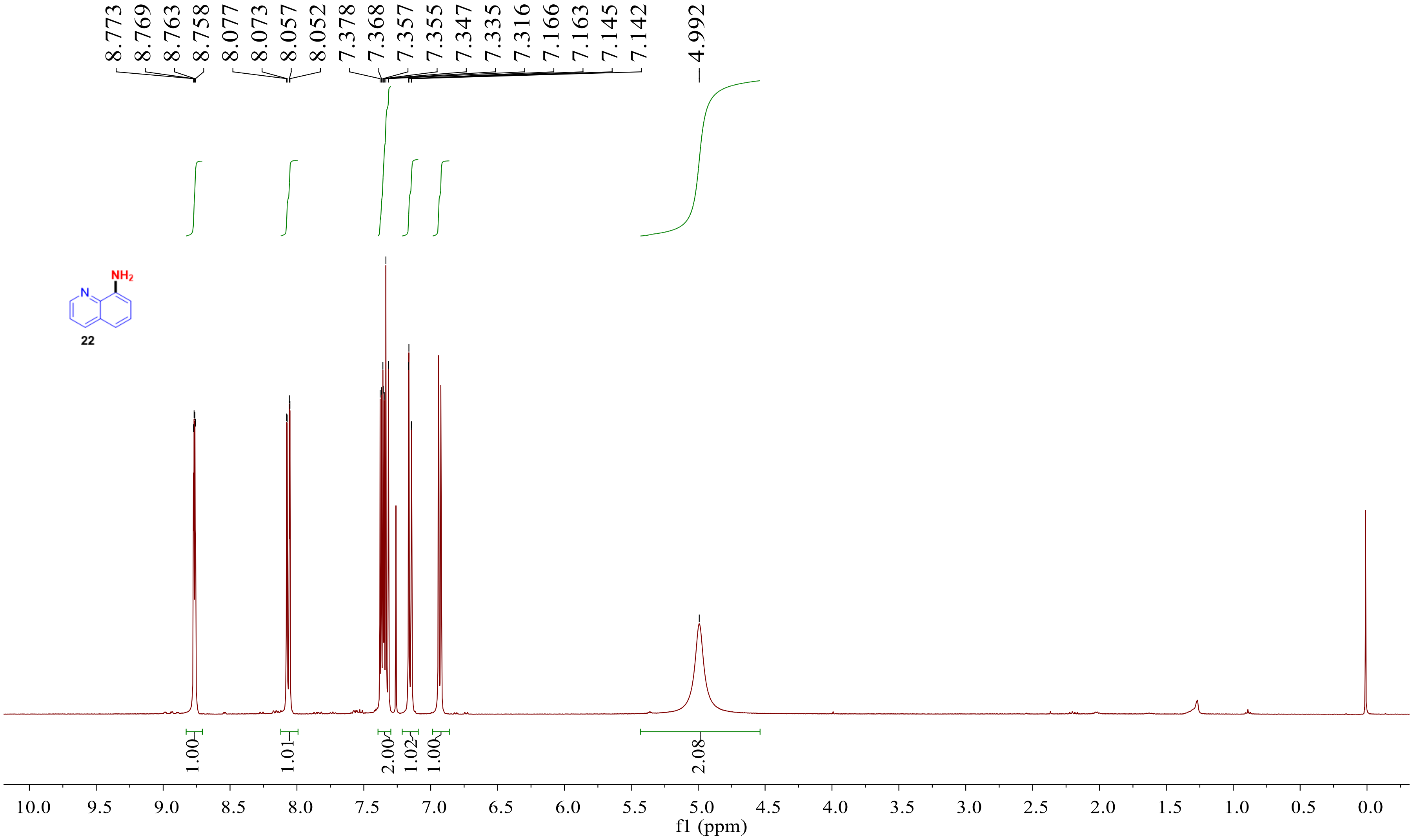
8.773
8.769
8.763
8.758
8.077
8.073
8.057
8.052
7.378
7.368
7.357
7.355
7.347
7.335
7.316
7.166
7.163
7.145
7.142
4.992
NH2
N
22
1.00
1.01
2.00
1.02
1.00
2.08
10.0
9.5
9.0
8.5
8.0
7.5
7.0
6.5
6.0
5.5
5.0
4.5
4.0
3.5
3.0
2.5
2.0
1.5
1.0
0.5
0.0
f1 (ppm)

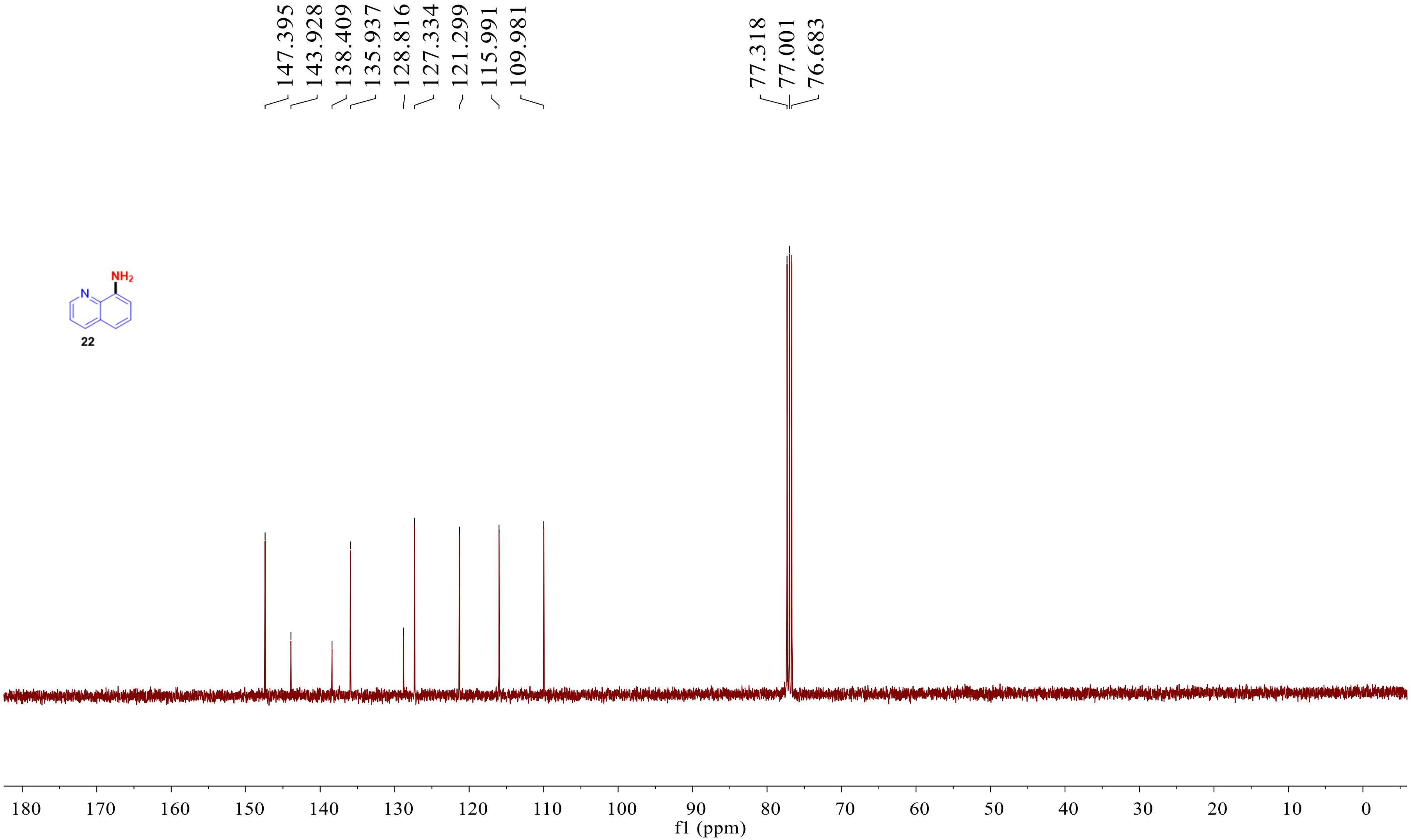
147.395
143.928
138.409
135.937
128.816
127.334
121.299
115.991
109.981
77.318
77.001
76.683
NH2
N
22
180 170 160 150 140 130 120 110 100 90 80 70 60 50 40 30 20 10 0
f1 (ppm)

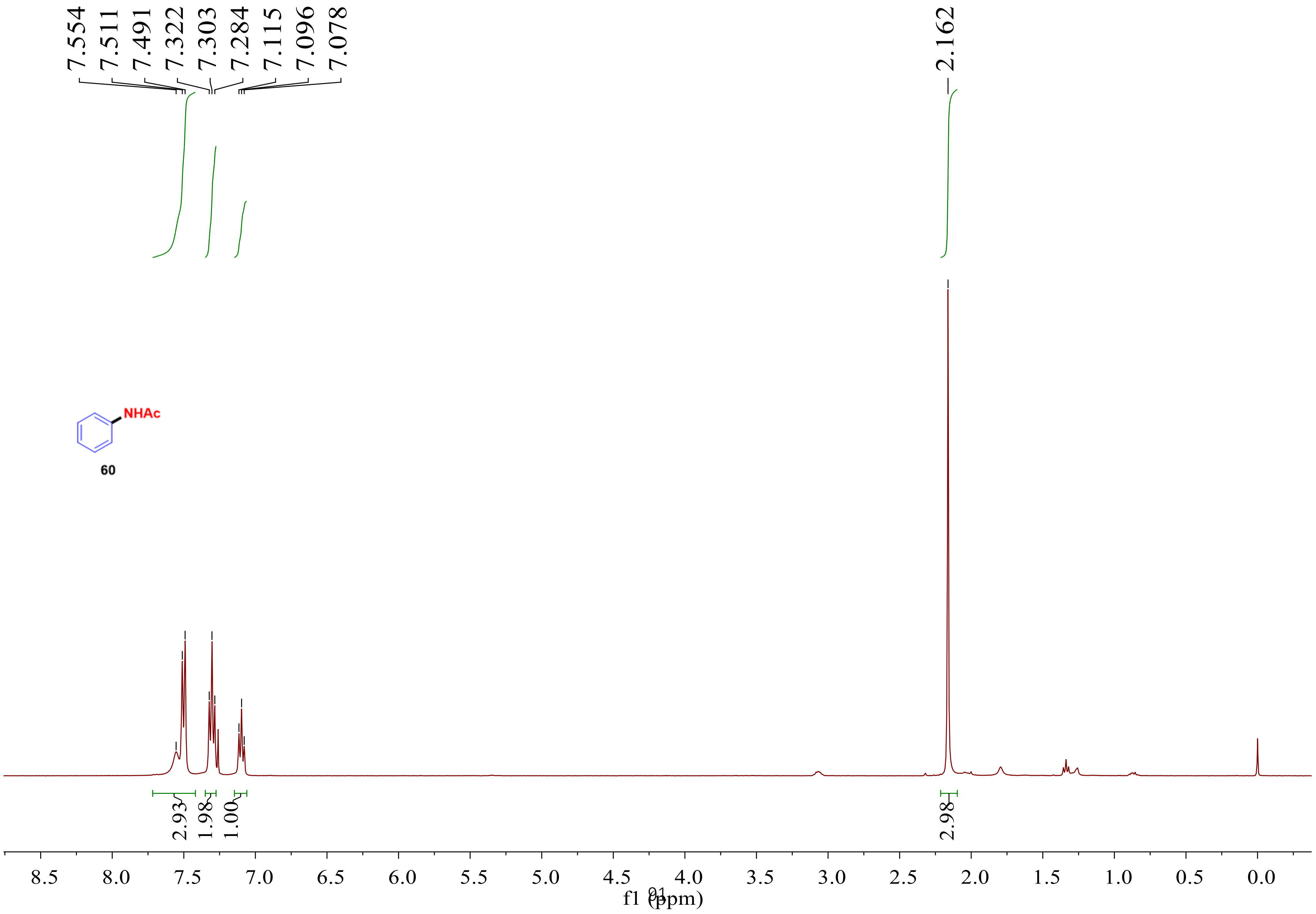

7.554
7.511
7.491
7.322
7.303
7.284
7.115
7.096
7.078
2.162
NHAc
60
2.93
1.98
1.00
2.98
8.5 8.0 7.5 7.0 6.5 6.0 5.5 5.0 4.5 4.0 3.5 3.0 2.5 2.0 1.5 1.0 0.5 0.0
f1 (ppm)

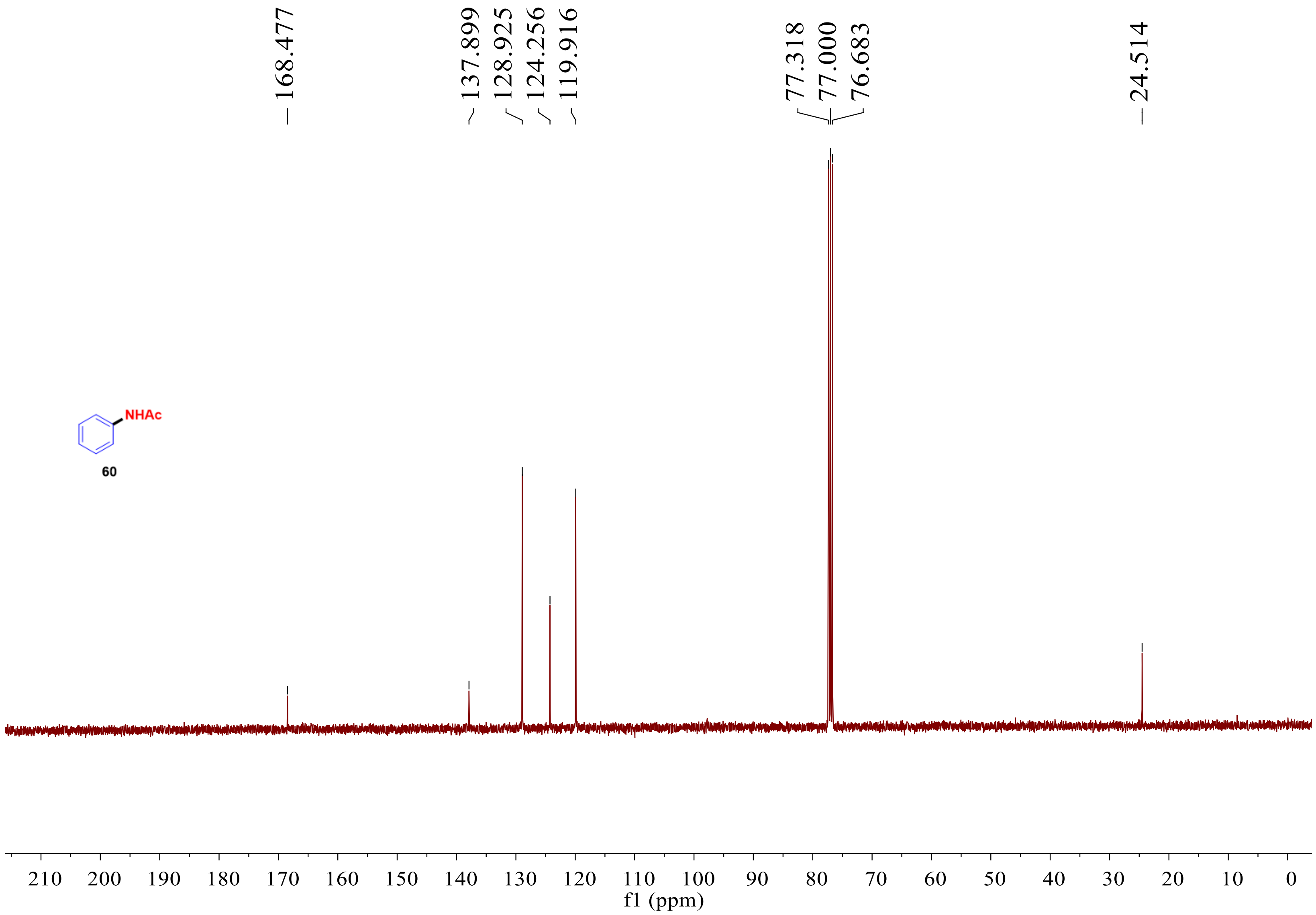
168.477
137.899
128.925
124.256
119.916
77.318
77.000
76.683
24.514
NHAc
60
210 200 190 180 170 160 150 140 130 120 110 100 90 80 70 60 50 40 30 20 10 0
f1 (ppm)

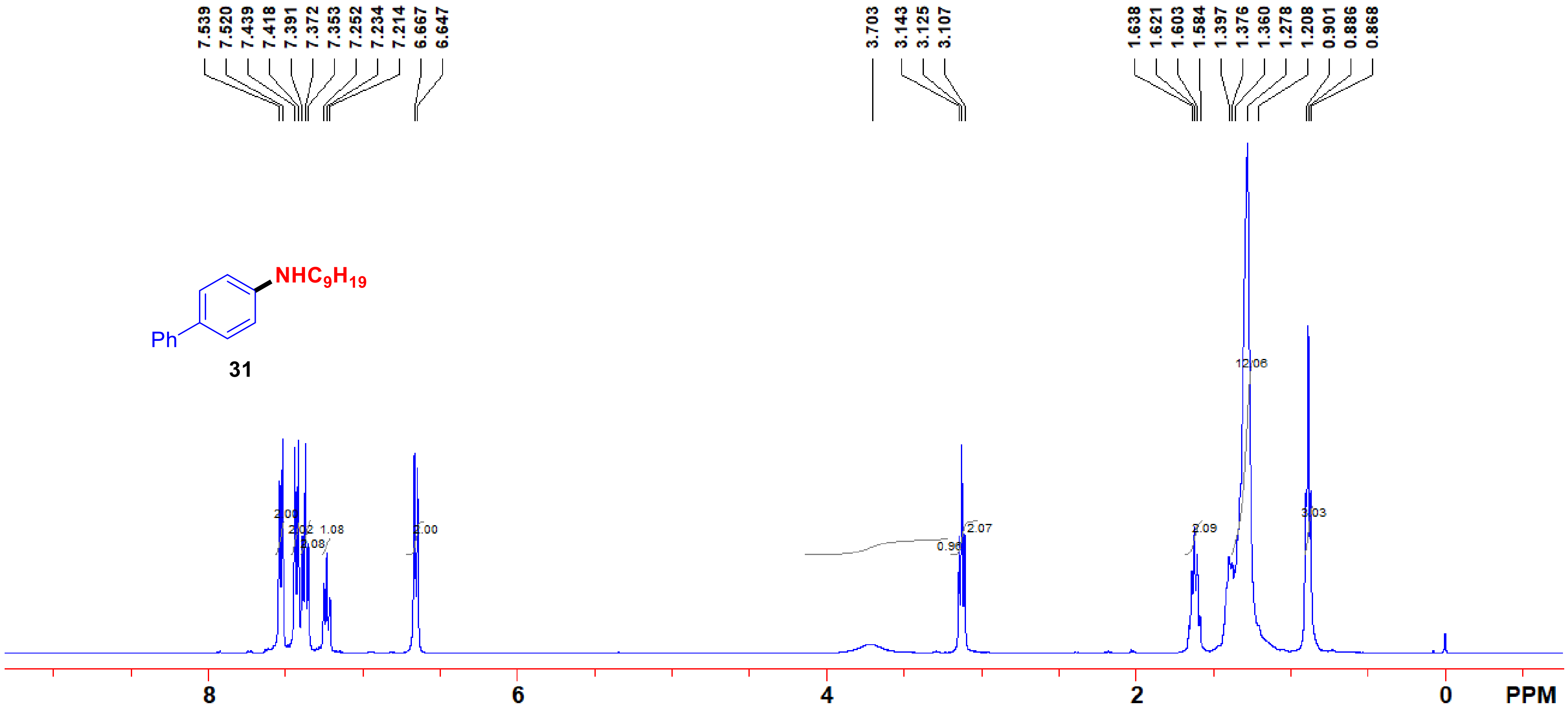
7.539
7.520
7.439
7.418
7.391
7.372
7.353
7.252
7.234
7.214
6.667
6.647
3.703
3.143
3.125
3.107
1.638
1.621
1.603
1.584
1.397
1.376
1.360
1.278
1.208
0.901
0.886
0.868
NHC9H19
Ph
31
2.00
2.02
1.08
1.08
2.00
0.96
2.07
2.09
12.06
3.03
8
6
4
2
0
PPM

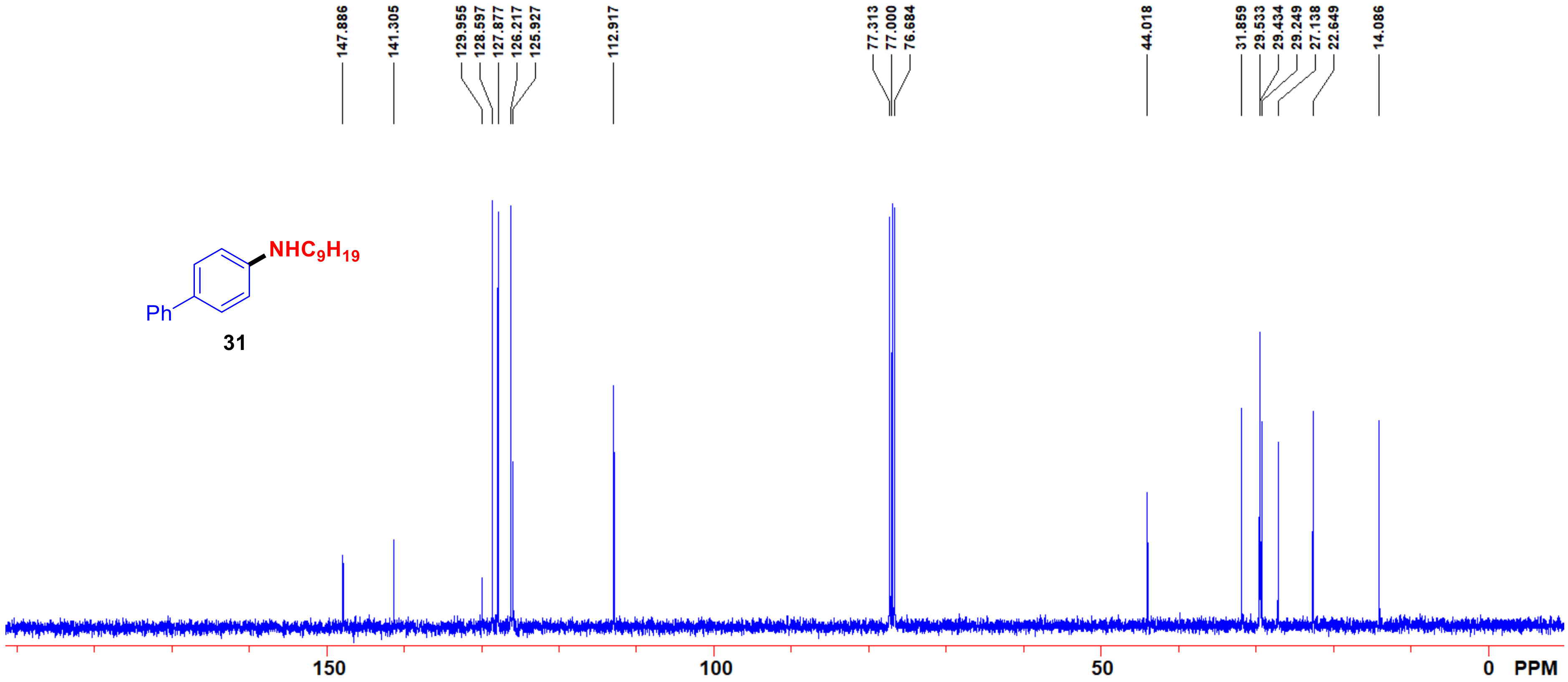
NHC9H19
Ph
31
147.886
141.305
129.955
128.597
127.877
126.217
125.927
112.917
77.313
77.000
76.684
44.018
31.859
29.533
29.434
29.249
27.138
22.649
14.086
150
100
50
0
PPM

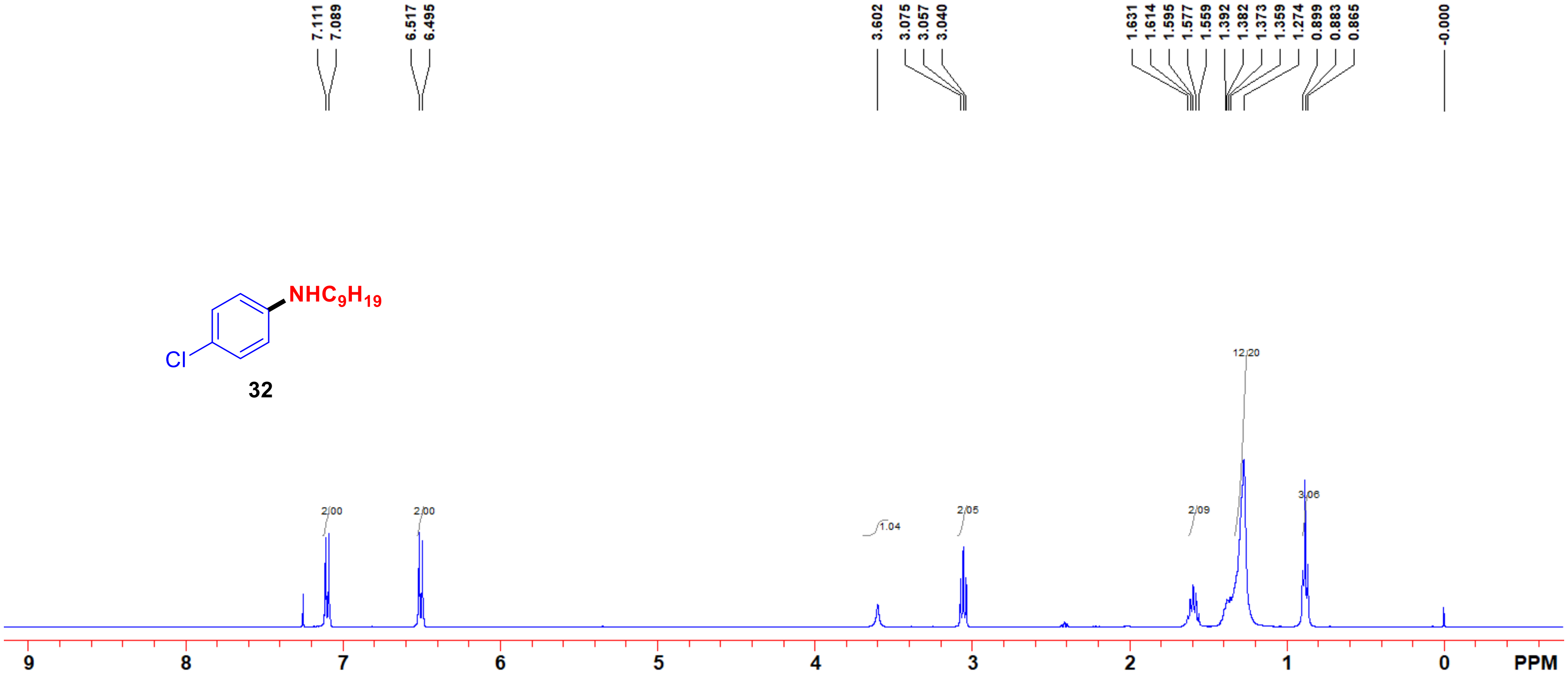
NHC9H19
Cl

32

7.111
7.089
6.517
6.495
3.602
3.075
3.057
3.040
1.631
1.614
1.595
1.577
1.559
1.392
1.382
1.373
1.359
1.274
0.899
0.883
0.865
-0.000
2.00
2.00
1.04
2.05
2.09
12.20
3.06
9
8
7
6
5
4
3
2
1
0
PPM

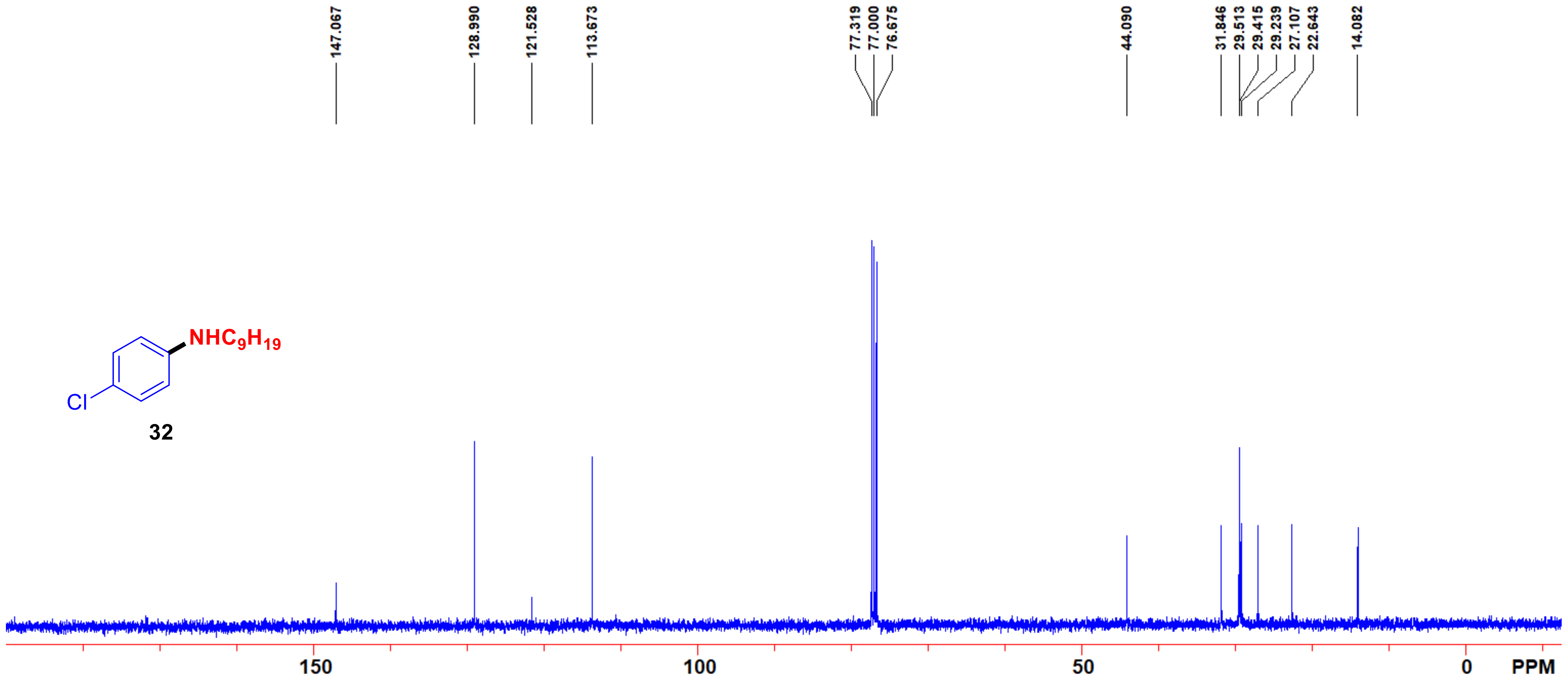

147.067
128.990
121.528
113.673
77.319
77.000
76.675
44.090
31.846
29.513
29.415
29.239
27.107
22.643
14.082
NHC9H19
Cl
32
150
100
50
0
PPM

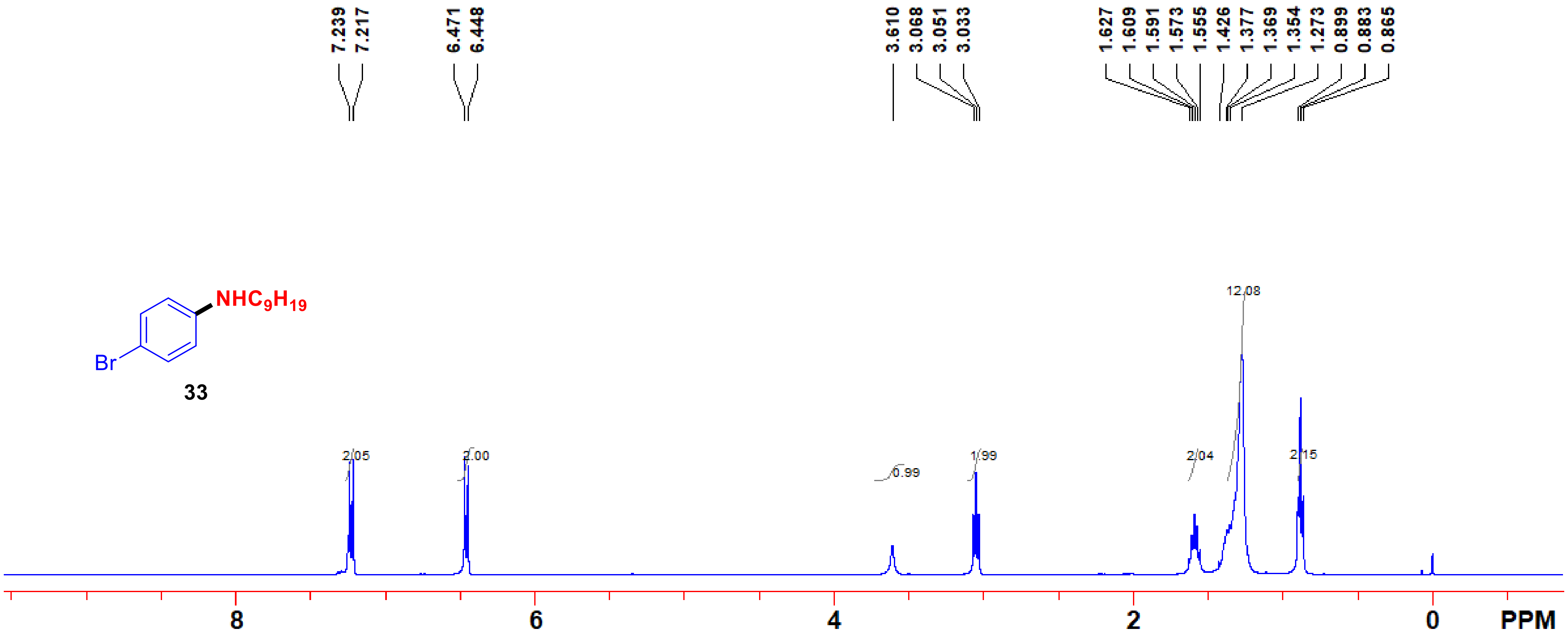

7.239
7.217
6.471
6.448
3.610
3.068
3.051
3.033
1.627
1.609
1.591
1.573
1.555
1.426
1.377
1.369
1.354
1.273
0.899
0.883
0.865
NHC9H19
Br
33
2.05
2.00
0.99
1.99
2.04
12.08
2.15
8
6
4
2
0
PPM

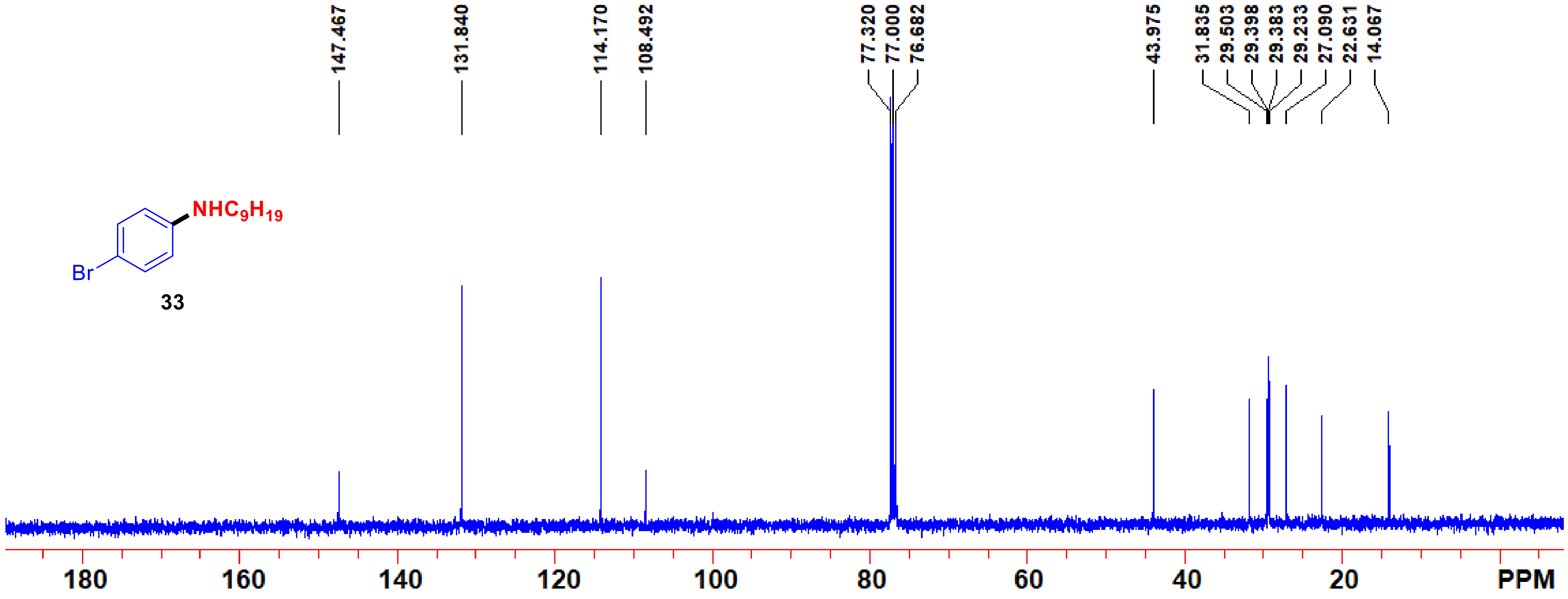

147.467
131.840
114.170
108.492
77.320
77.000
76.682
43.975
31.835
29.503
29.398
29.383
29.233
27.090
22.631
14.067
NHC9H19
Br
33
180
160
140
120
100
80
60
40
20
PPM

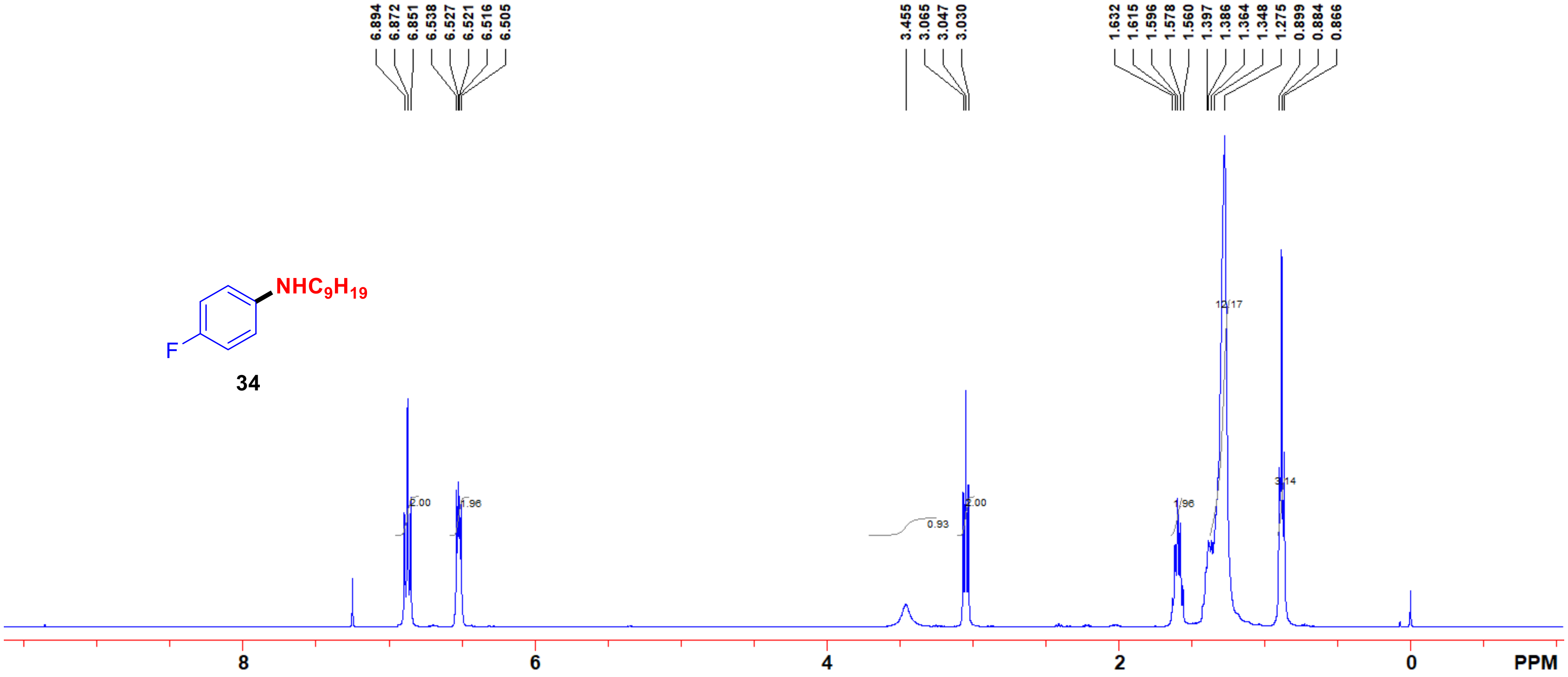

6.894
6.872
6.851
6.538
6.527
6.521
6.516
6.505
3.455
3.065
3.047
3.030
1.632
1.615
1.596
1.578
1.560
1.397
1.386
1.364
1.348
1.275
0.899
0.884
0.866
NHC9H19
F
34
2.00
1.96
0.93
2.00
1.96
12.17
3.14
8
6
4
2
0
PPM

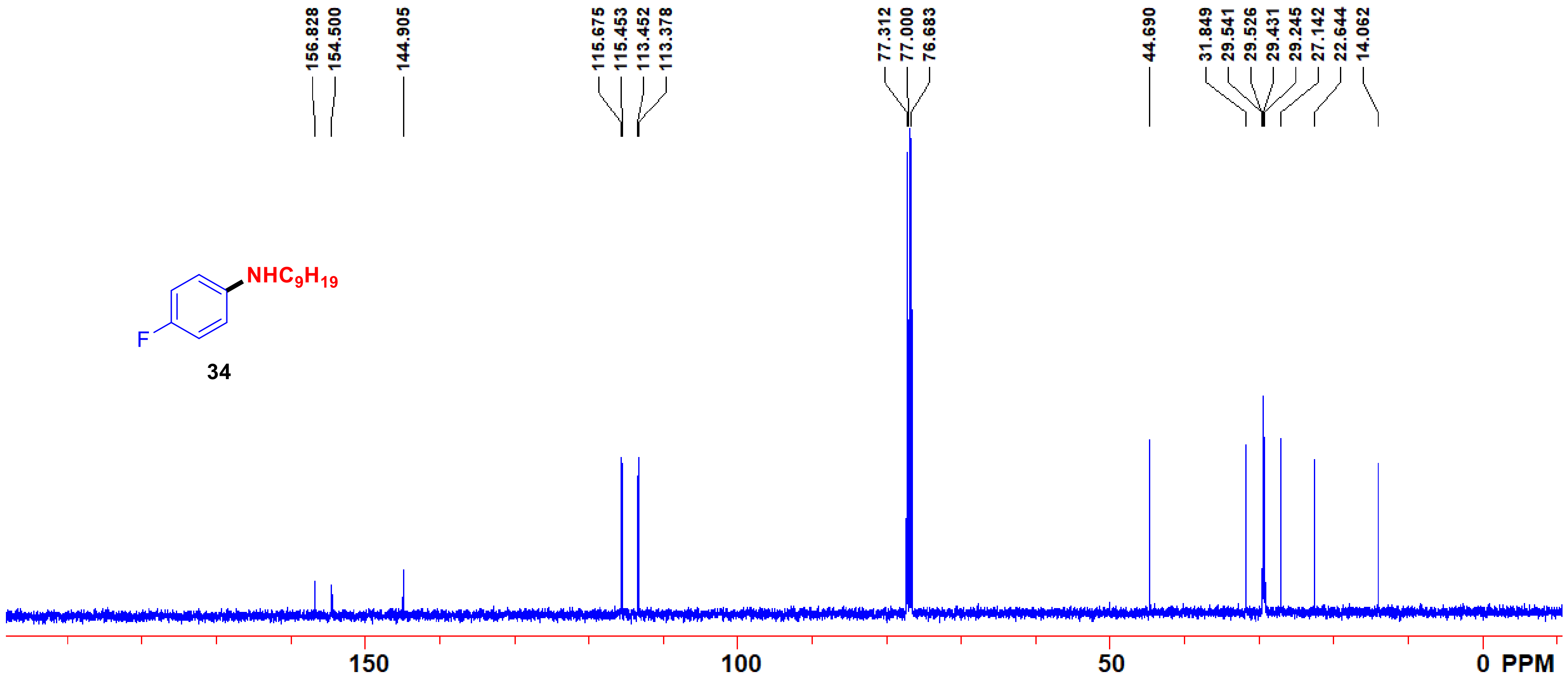

156.828
154.500
144.905
115.675
115.453
113.452
113.378
77.312
77.000
76.683
44.690
31.849
29.541
29.526
29.431
29.245
27.142
22.644
14.062
NHC9H19
F
34
150
100
50
0 PPM

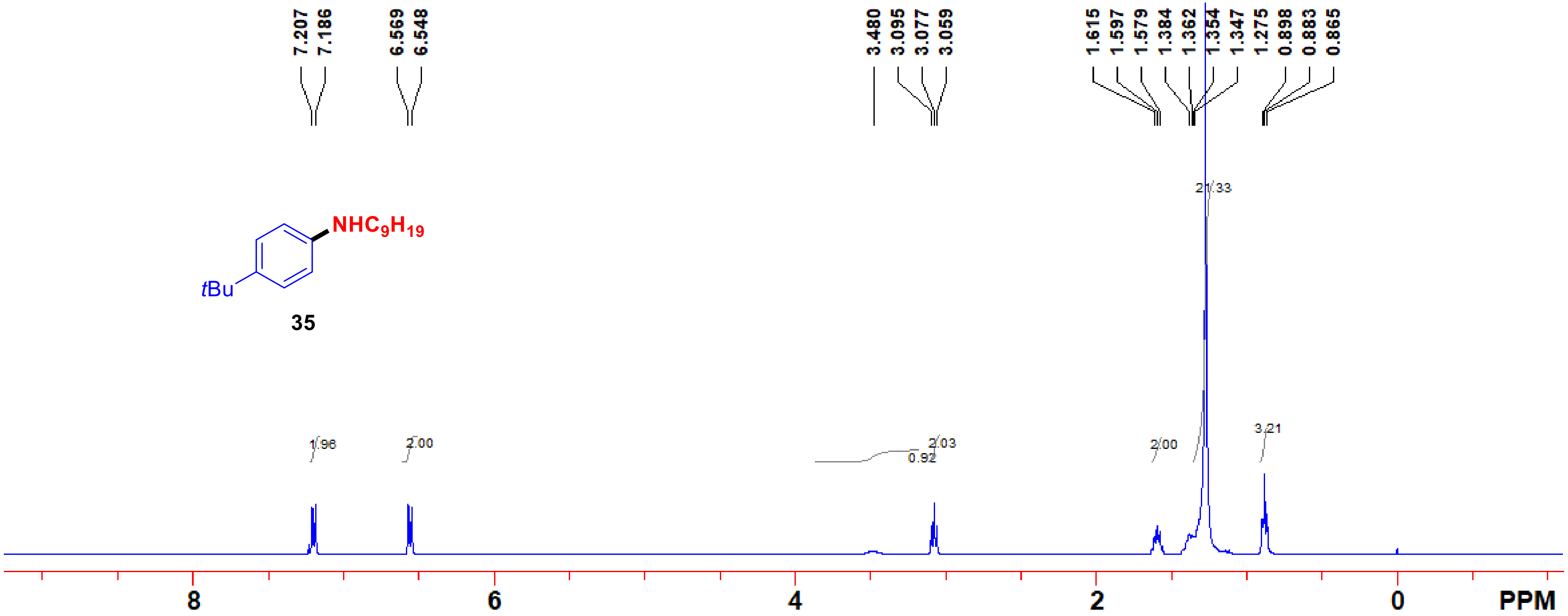
7.207
7.186
6.569
6.548
3.480
3.095
3.077
3.059
1.615
1.597
1.579
1.384
1.362
1.354
1.347
1.275
0.898
0.883
0.865
NHC9H19
tBu
35
1.96
2.00
2.03
0.92
2.00
21.33
3.21
8
6
4
2
0
PPM

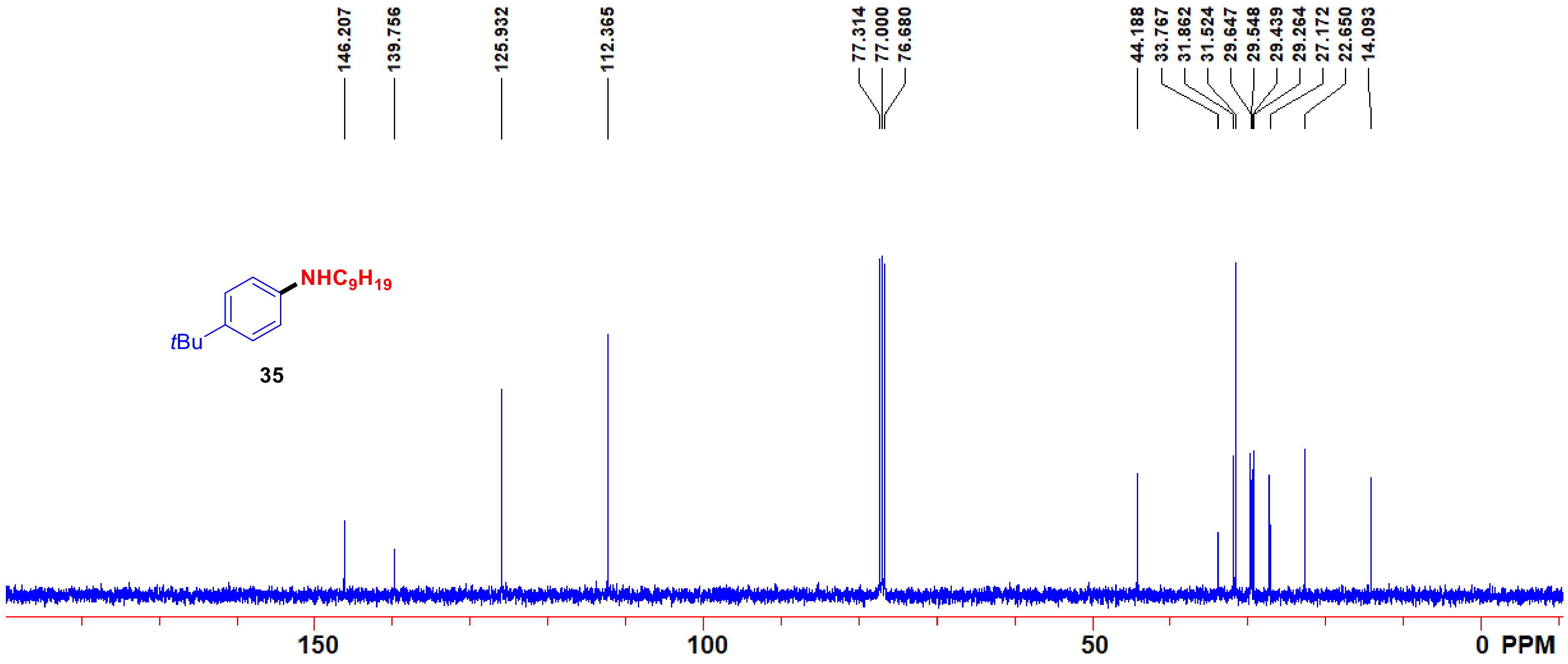
146.207
139.756
125.932
112.365
77.314
77.000
76.680
44.188
33.767
31.862
31.524
29.647
29.548
29.439
29.264
27.172
22.650
14.093
NHC9H19
tBu
35
150
100
50
0 PPM

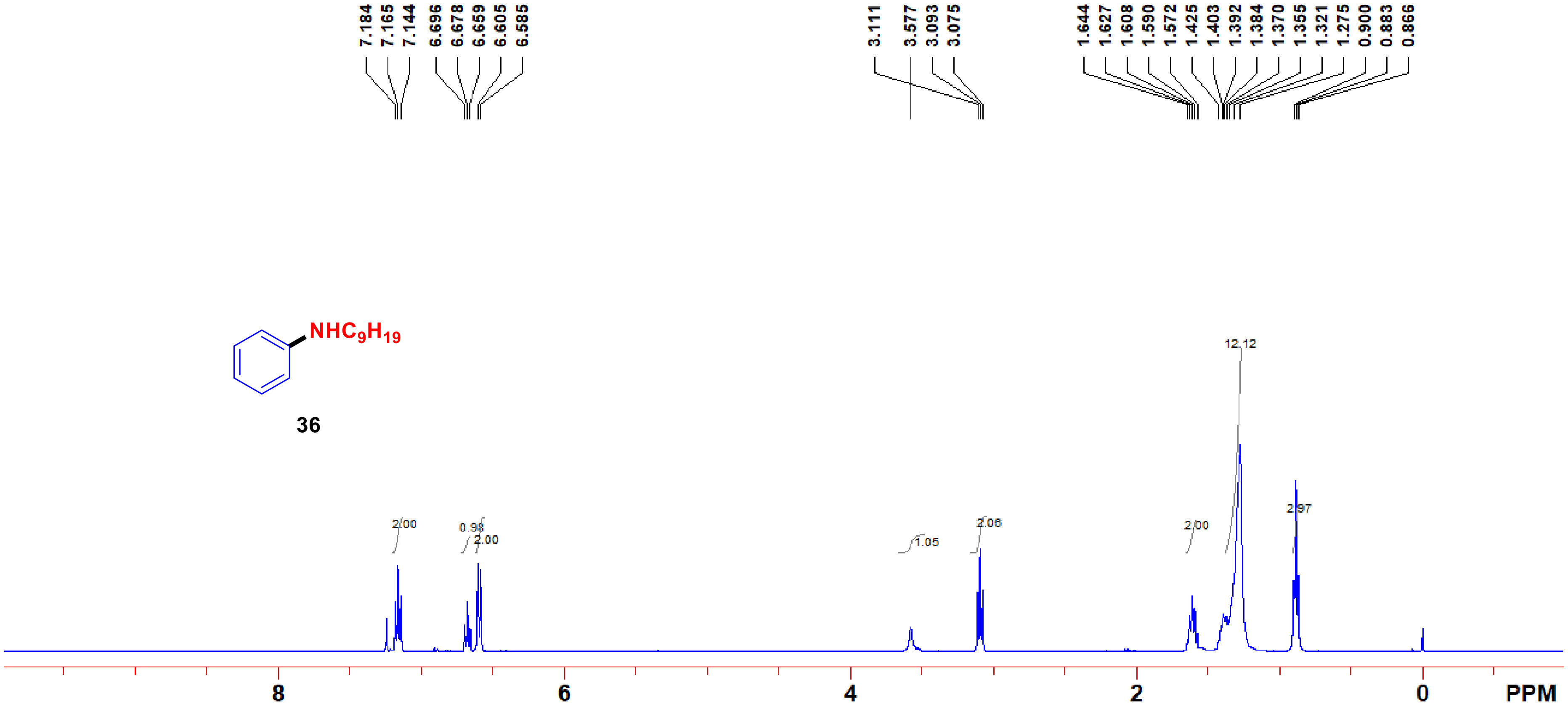

7.184
7.165
7.144
6.696
6.678
6.659
6.605
6.585
3.111
3.577
3.093
3.075
1.644
1.627
1.608
1.590
1.572
1.425
1.403
1.392
1.384
1.370
1.355
1.321
1.275
0.900
0.883
0.866
NHC9H19
36
2.00
0.98
2.00
1.05
2.06
2.00
12.12
2.97
8
6
4
2
0
PPM

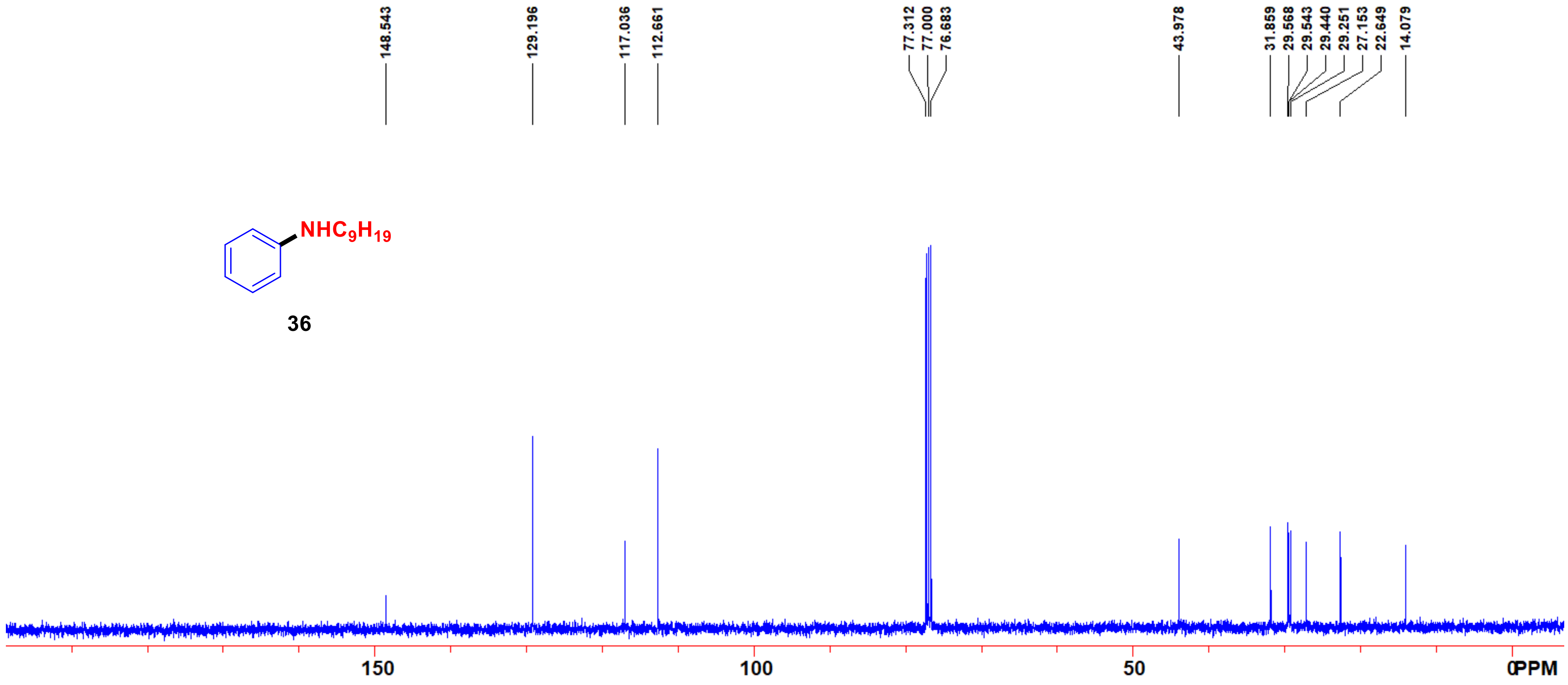

148.543
129.196
117.036
112.661
77.312
77.000
76.683
43.978
31.859
29.568
29.543
29.440
29.251
27.153
22.649
14.079
NHC9H19
36
150
100
50
0PPM

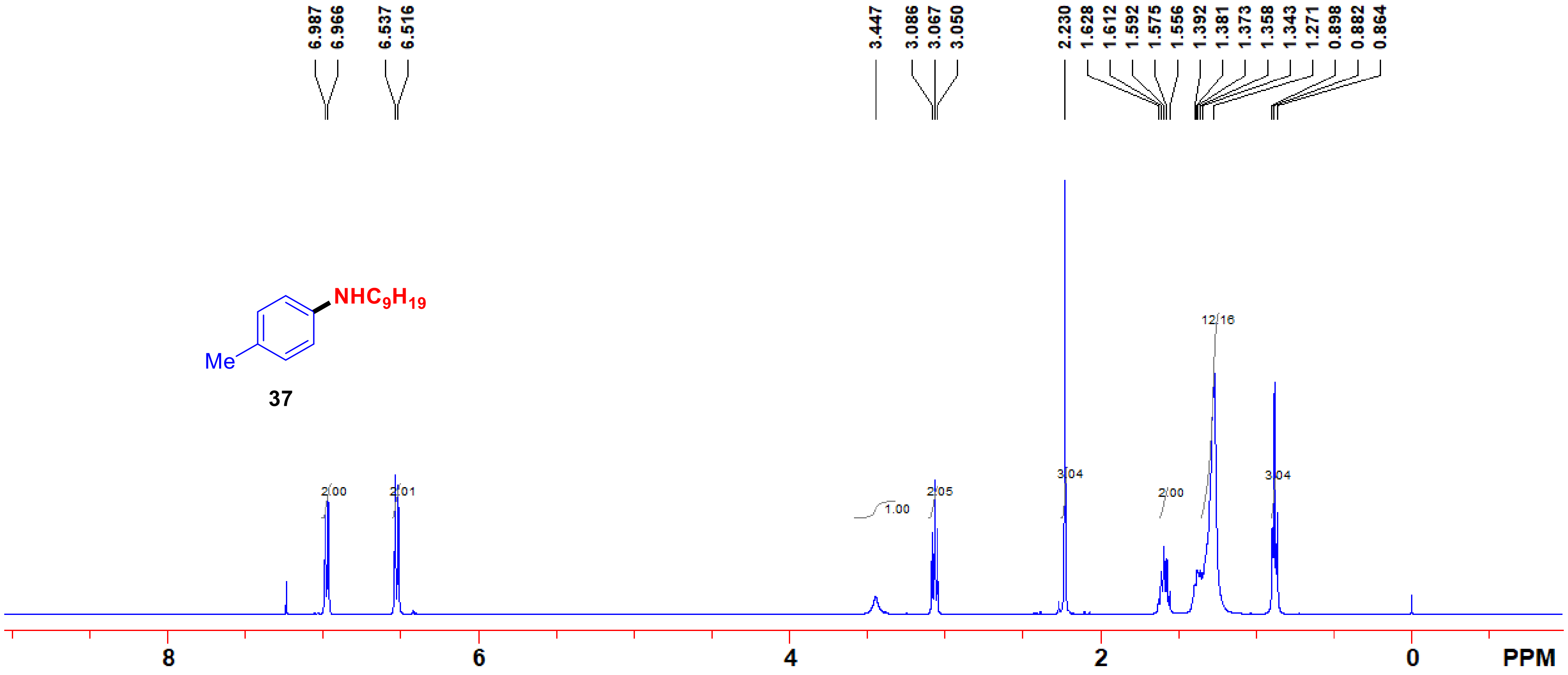

6.987
6.966
6.537
6.516
3.447
3.086
3.067
3.050
2.230
1.628
1.612
1.592
1.575
1.556
1.392
1.381
1.373
1.358
1.343
1.271
0.898
0.882
0.864
NHC9H19
Me
37
2.00
2.01
1.00
2.05
3.04
2.00
12.16
3.04
8
6
4
2
0
PPM

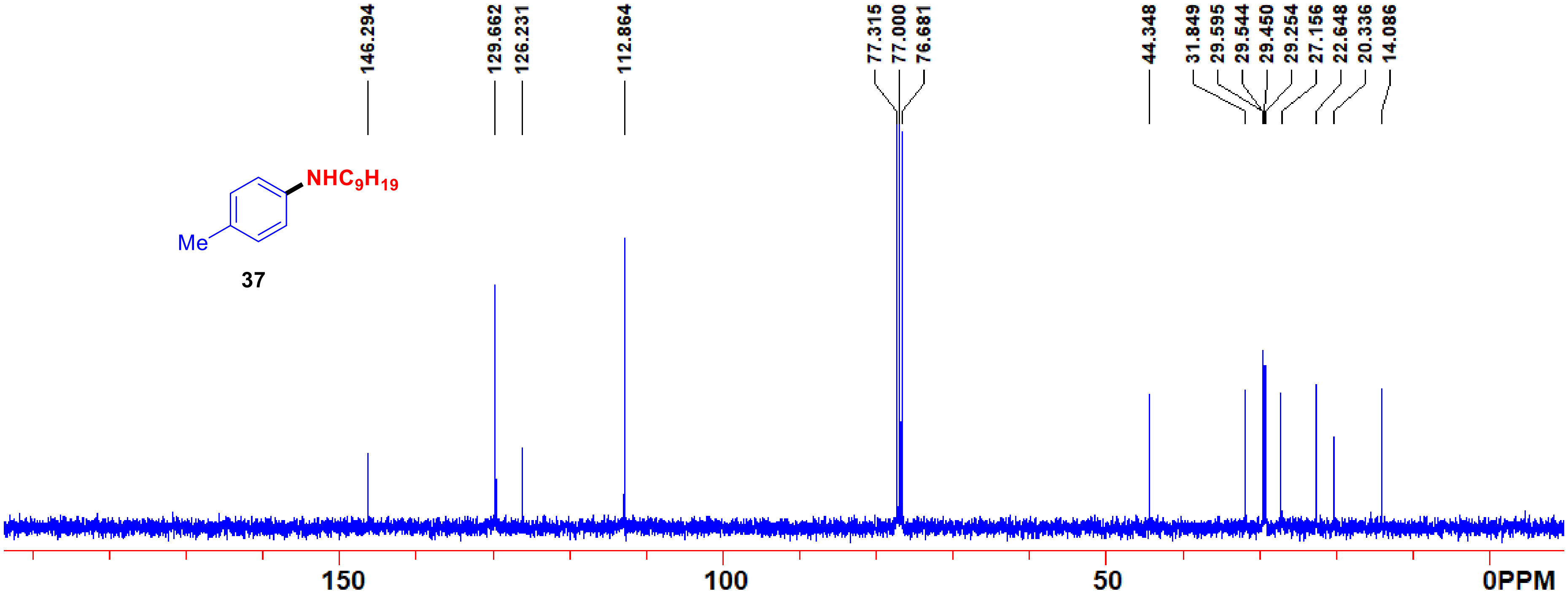
146.294
129.662
126.231
112.864
77.315
77.000
76.681
44.348
31.849
29.595
29.544
29.450
29.254
27.156
22.648
20.336
14.086
NHC9H19
Me
37
150
100
50
0PPM

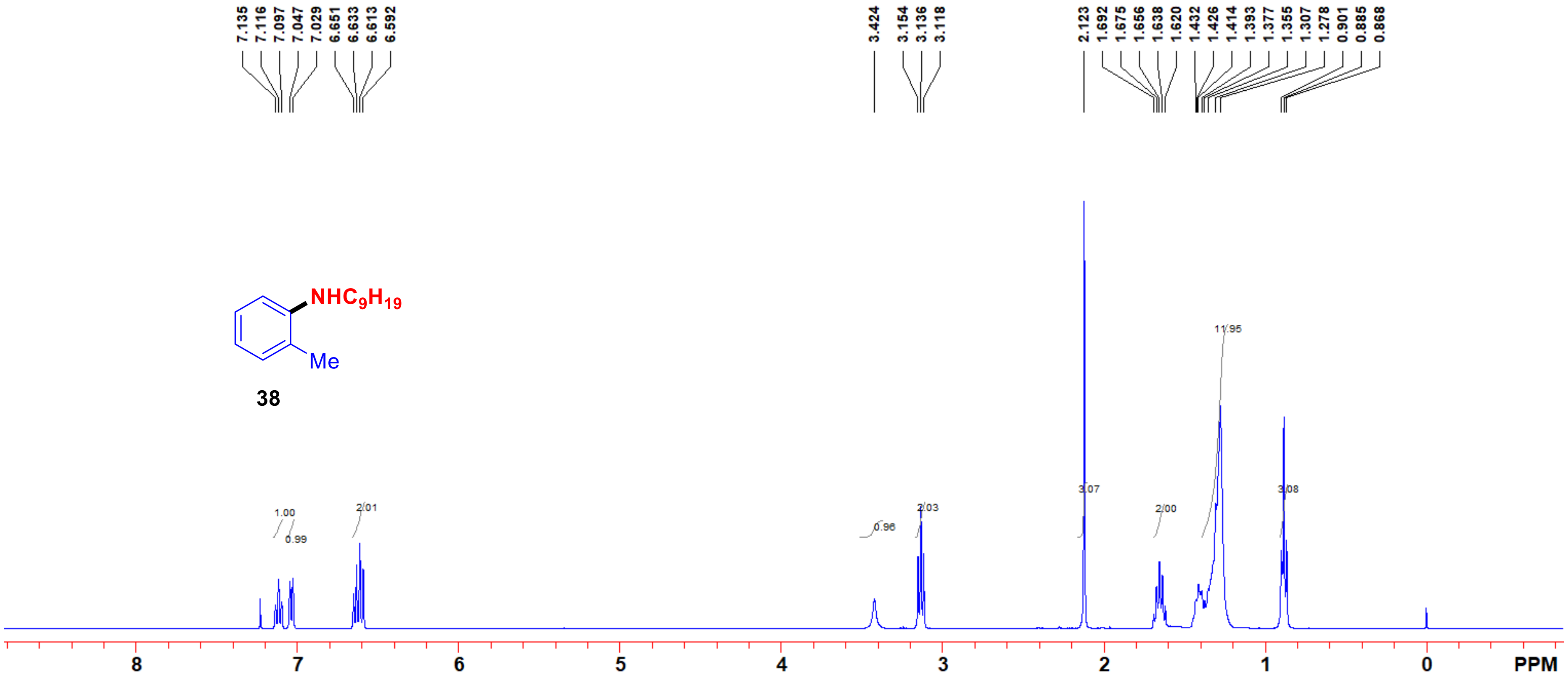
7.135
7.116
7.097
7.047
7.029
6.651
6.633
6.613
6.592
3.424
3.154
3.136
3.118
2.123
1.692
1.675
1.656
1.638
1.620
1.432
1.426
1.414
1.393
1.377
1.355
1.307
1.278
0.901
0.885
0.868
NHC9H19
Me
1.00
0.99
2.01
0.96
2.03
3.07
2.00
11.95
3.08
8
7
6
5
4
3
2
1
0
PPM

38

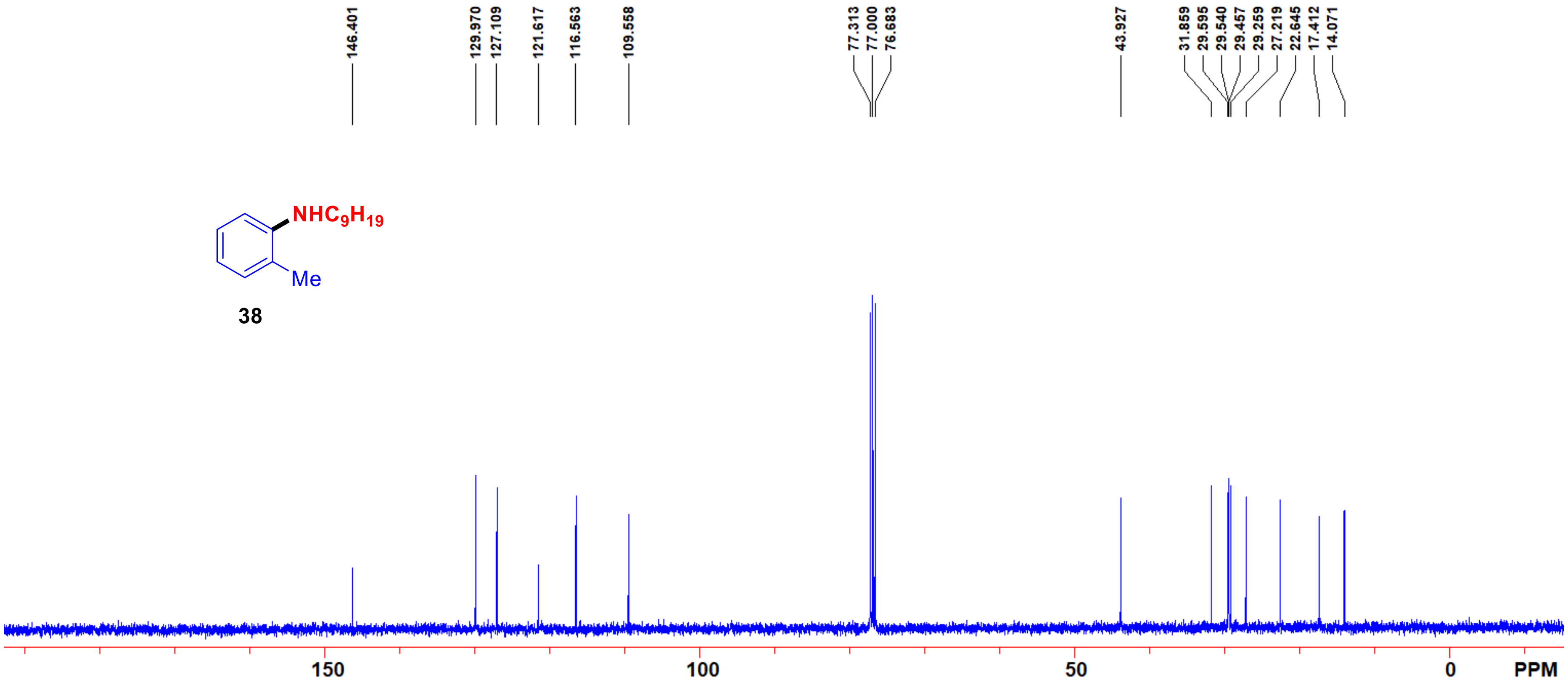

146.401
129.970
127.109
121.617
116.563
109.558
77.313
77.000
76.683
43.927
31.859
29.595
29.540
29.457
29.259
27.219
22.645
17.412
14.071
NHC9H19
Me
150
100
50
0
PPM

38

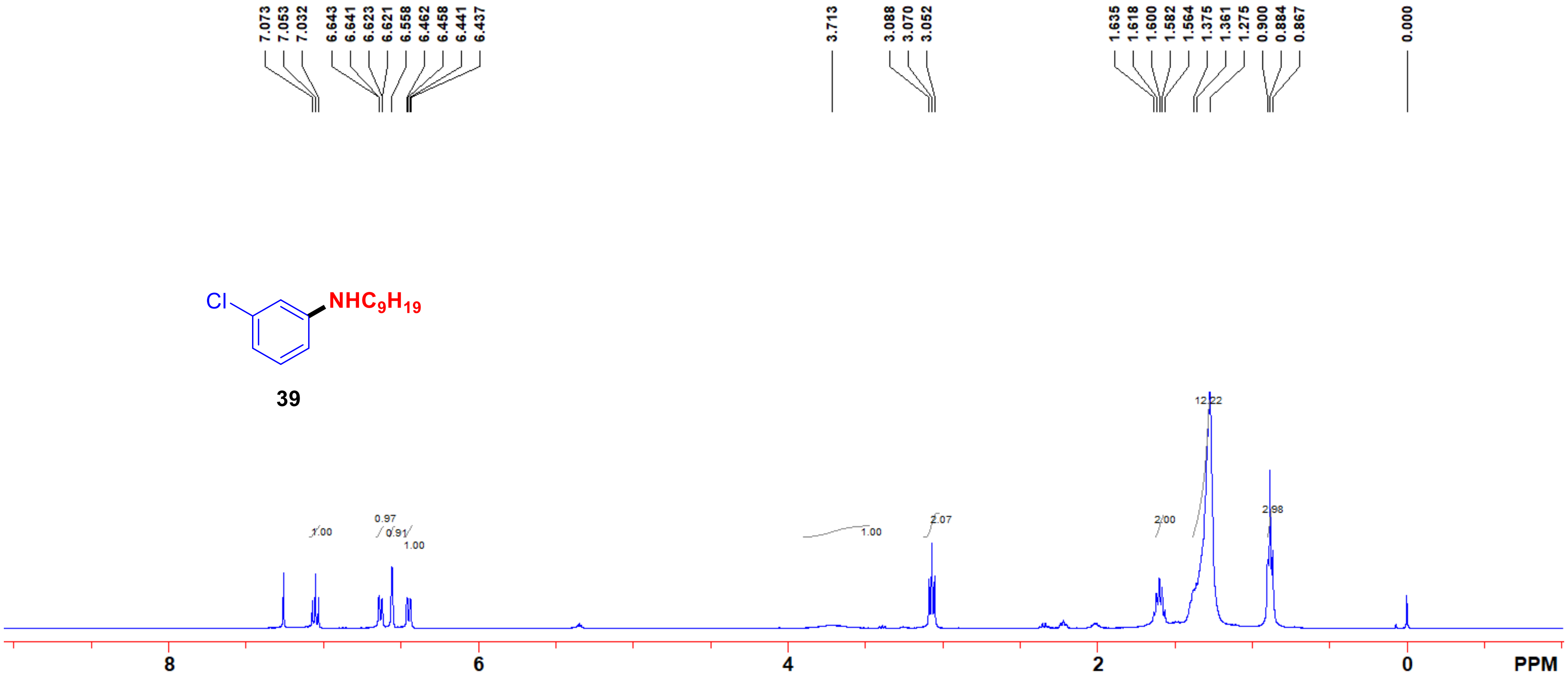
7.073
7.053
7.032
6.643
6.641
6.623
6.621
6.558
6.462
6.458
6.441
6.437
3.713
3.088
3.070
3.052
1.635
1.618
1.600
1.582
1.564
1.375
1.361
1.275
0.900
0.884
0.867
0.000
Cl
NHC9H19
39
1.00
0.97
0.91
1.00
1.00
2.07
2.00
12.22
2.98
8
6
4
2
0
PPM

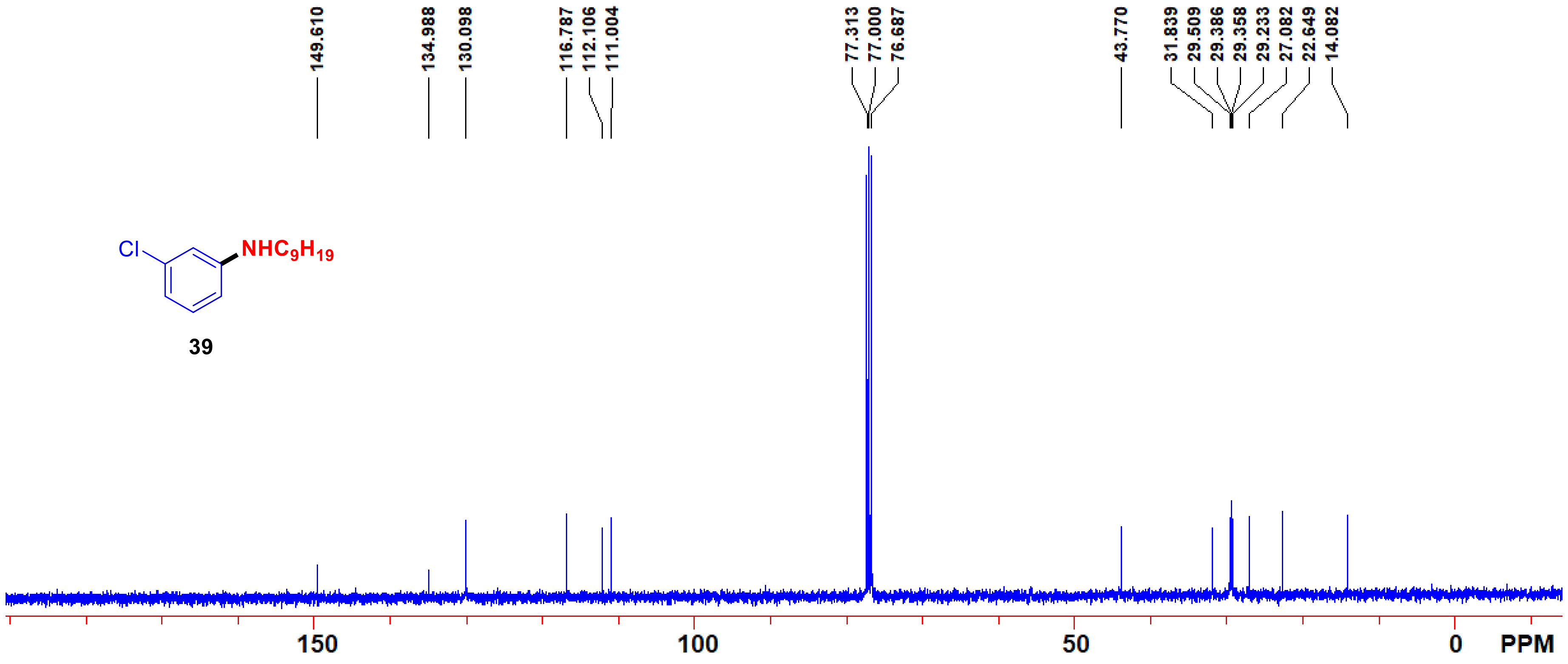
149.610
134.988
130.098
116.787
112.106
111.004
77.313
77.000
76.687
43.770
31.839
29.509
29.386
29.358
29.233
27.082
22.649
14.082
Cl
NHC_9H_{19}
150
100
50
0
PPM

39

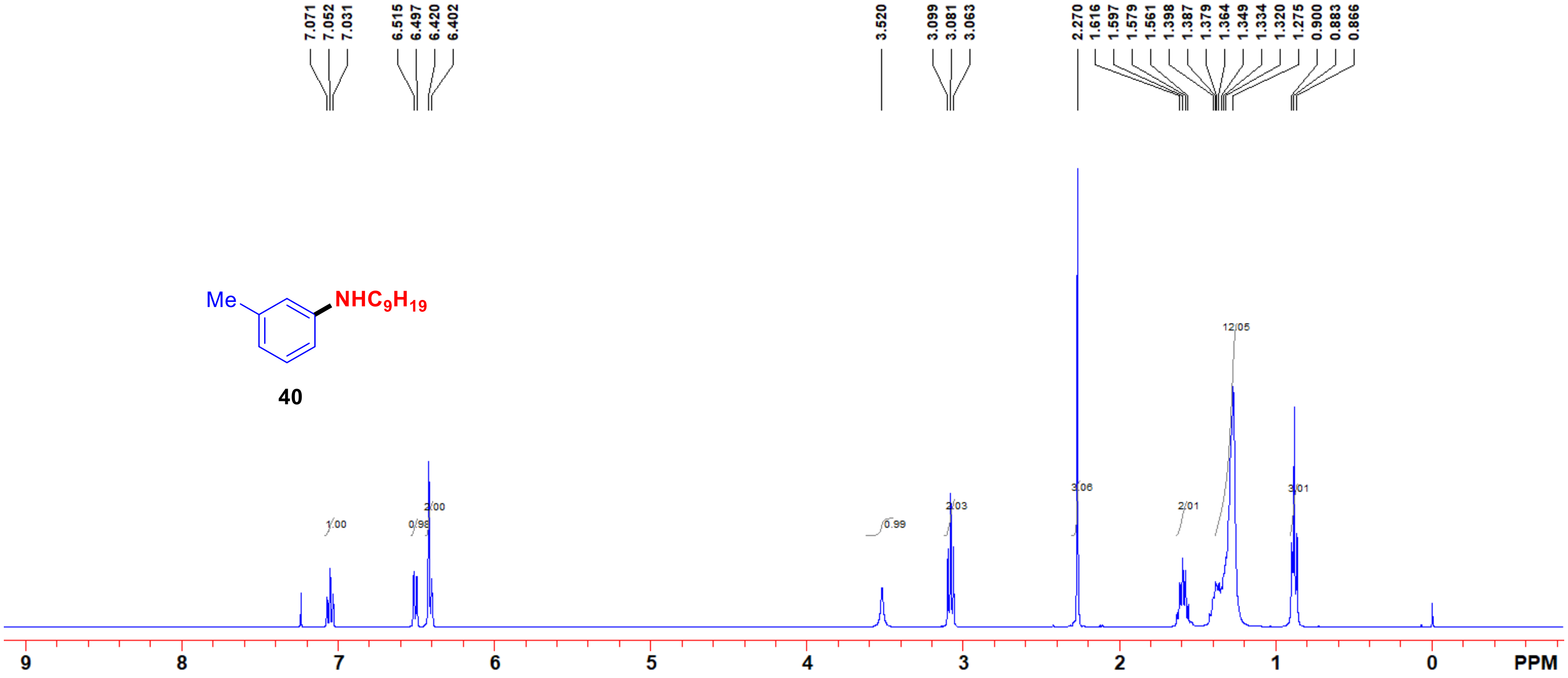
7.071
7.052
7.031
6.515
6.497
6.420
6.402
3.520
3.099
3.081
3.063
2.270
1.616
1.597
1.579
1.561
1.398
1.387
1.379
1.364
1.349
1.334
1.320
1.275
0.900
0.883
0.866
Me
NHC9H19
40
1.00
0.98
2.00
0.99
2.03
3.06
2.01
12.05
3.01
9
8
7
6
5
4
3
2
1
0
PPM

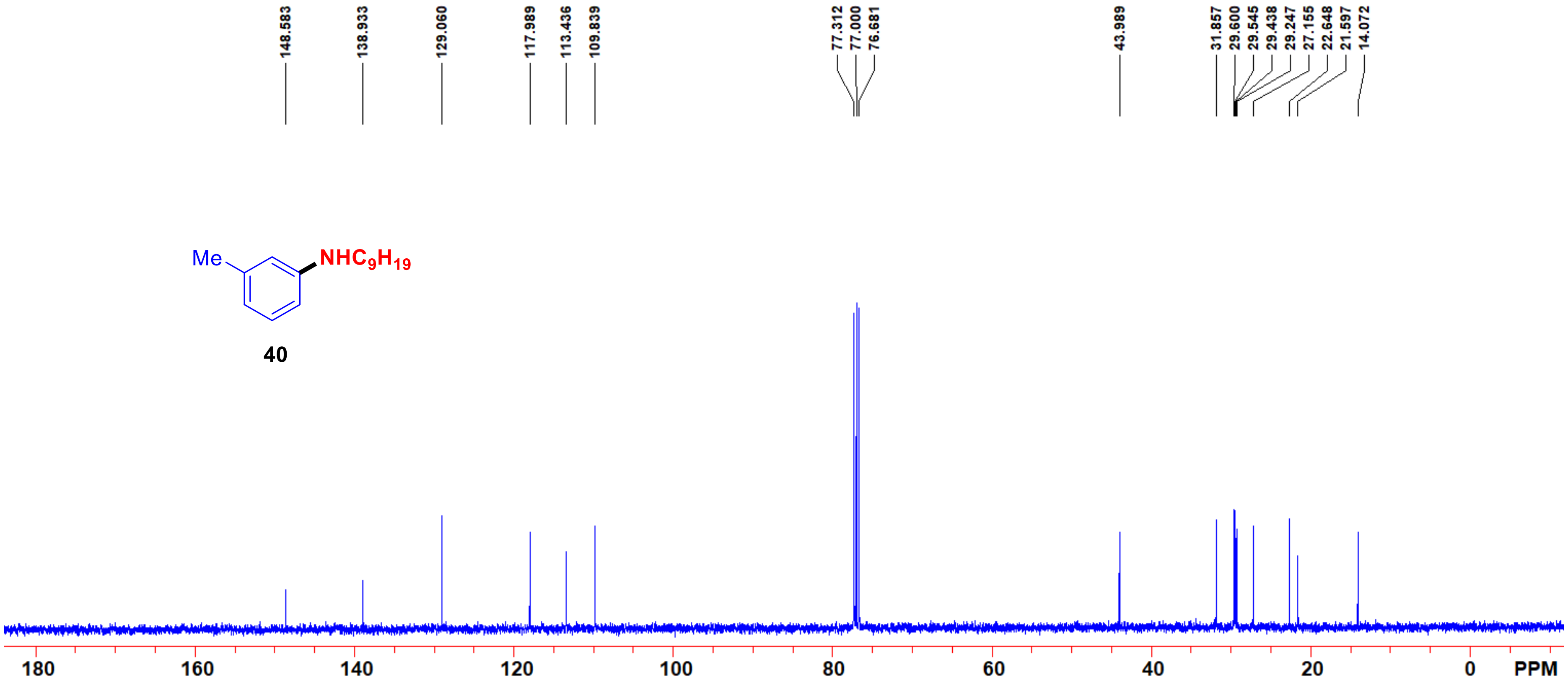
148.583
138.933
129.060
117.989
113.436
109.839
77.312
77.000
76.681
43.989
31.857
29.600
29.545
29.438
29.247
27.155
22.648
21.597
14.072
Me
NHC9H19
40
180
160
140
120
100
80
60
40
20
0
PPM

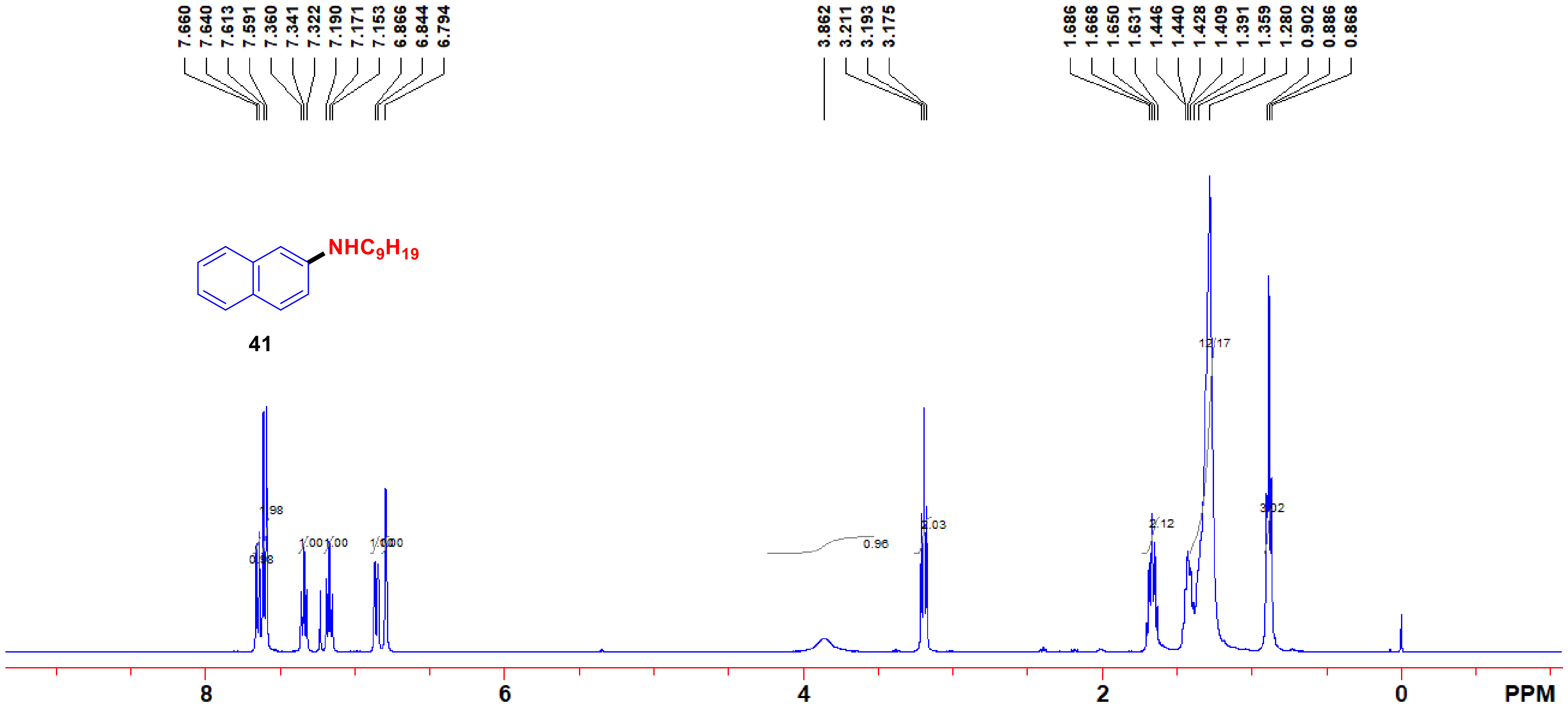
7.660
7.640
7.613
7.591
7.360
7.341
7.322
7.190
7.171
7.153
6.866
6.844
6.794
3.862
3.211
3.193
3.175
1.686
1.668
1.650
1.631
1.446
1.440
1.428
1.409
1.391
1.359
1.280
0.902
0.886
0.868
NHC9H19
41
1.98
0.98
1.00
1.00
0.96
2.03
2.12
13.17
3.02
8
6
4
2
0
PPM

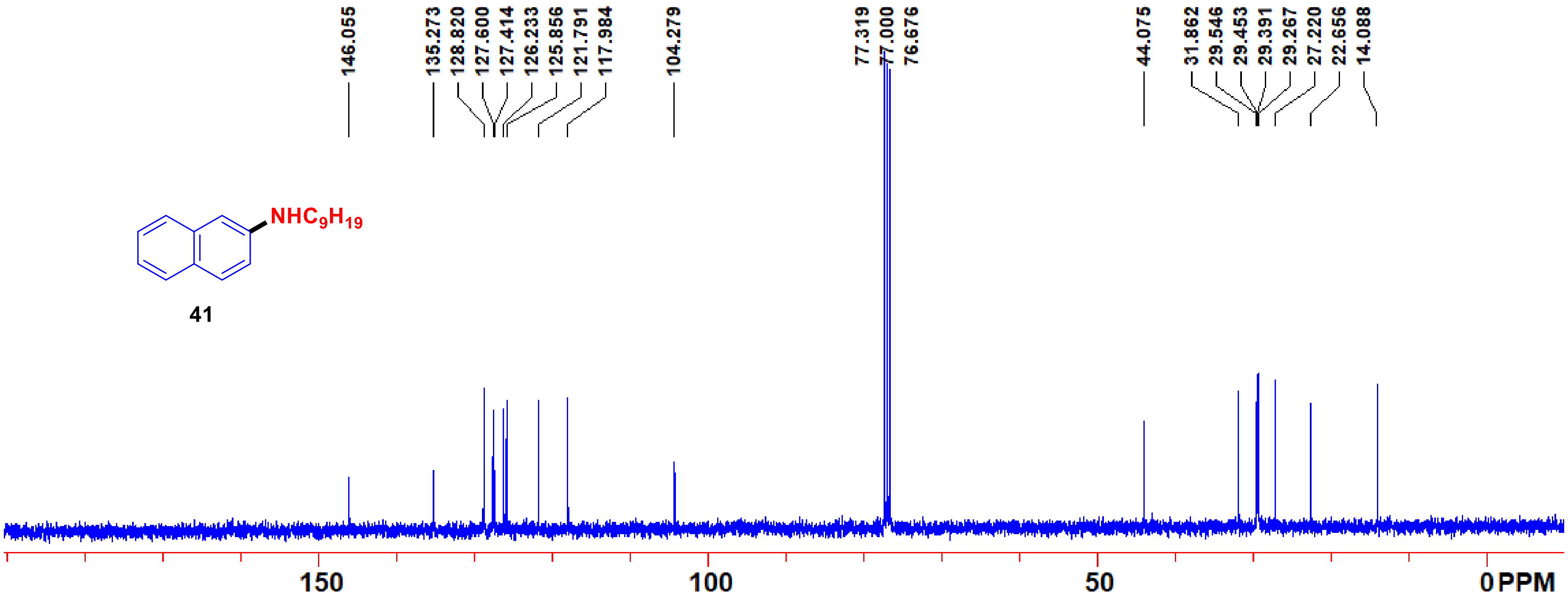

146.055
135.273
128.820
127.600
127.414
126.233
125.856
121.791
117.984
104.279
77.319
77.000
76.676
44.075
31.862
29.546
29.453
29.391
29.267
27.220
22.656
14.088
NHC9H19
41
150
100
50
0 PPM

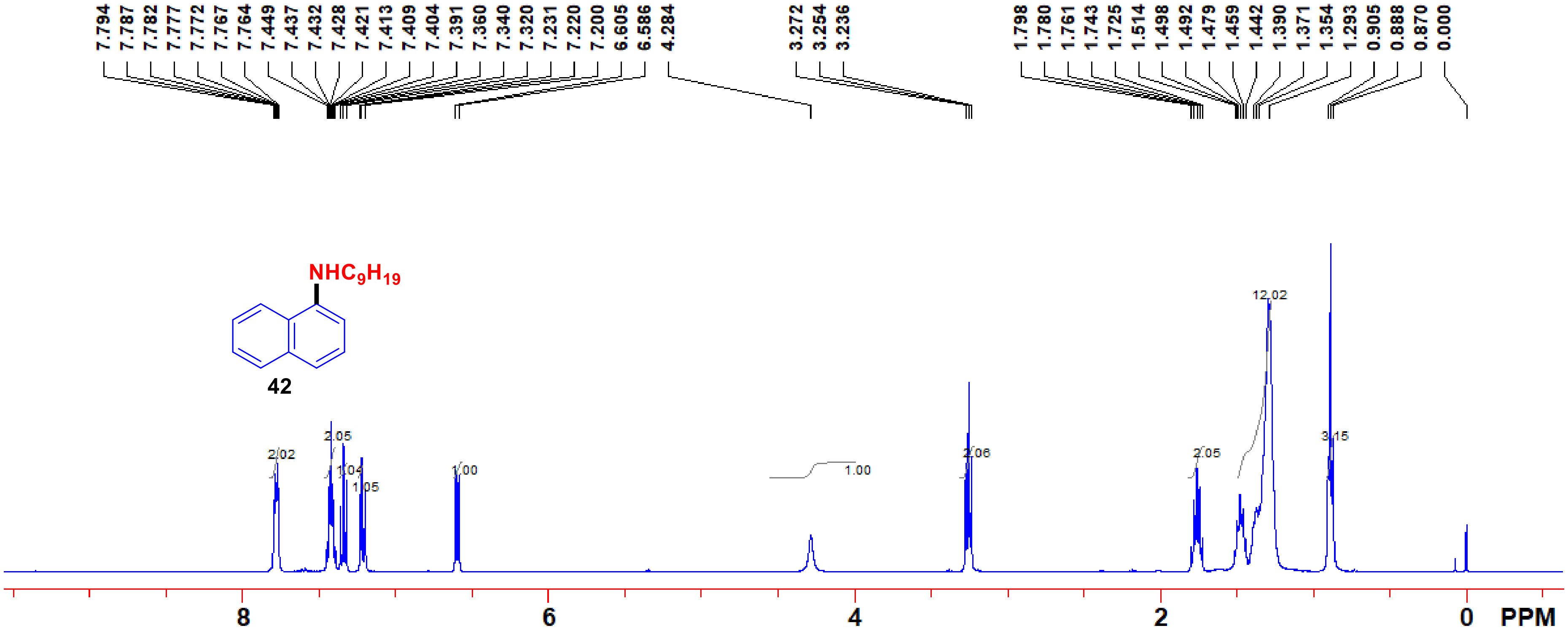
7.794 7.787 7.782 7.777 7.772 7.767 7.764 7.449 7.437 7.432 7.428 7.421 7.413 7.409 7.404 7.391 7.360 7.340 7.320 7.231 7.220 7.200 6.605 6.586 4.284
3.272 3.254 3.236
1.798 1.780 1.761 1.743 1.725 1.514 1.498 1.492 1.479 1.459 1.442 1.390 1.371 1.354 1.293 0.905 0.888 0.870 0.000
NHC9H19
42
2.02
2.05
1.04
1.05
1.00
1.00
2.06
2.05
12.02
3.15
8
6
4
2
0
PPM

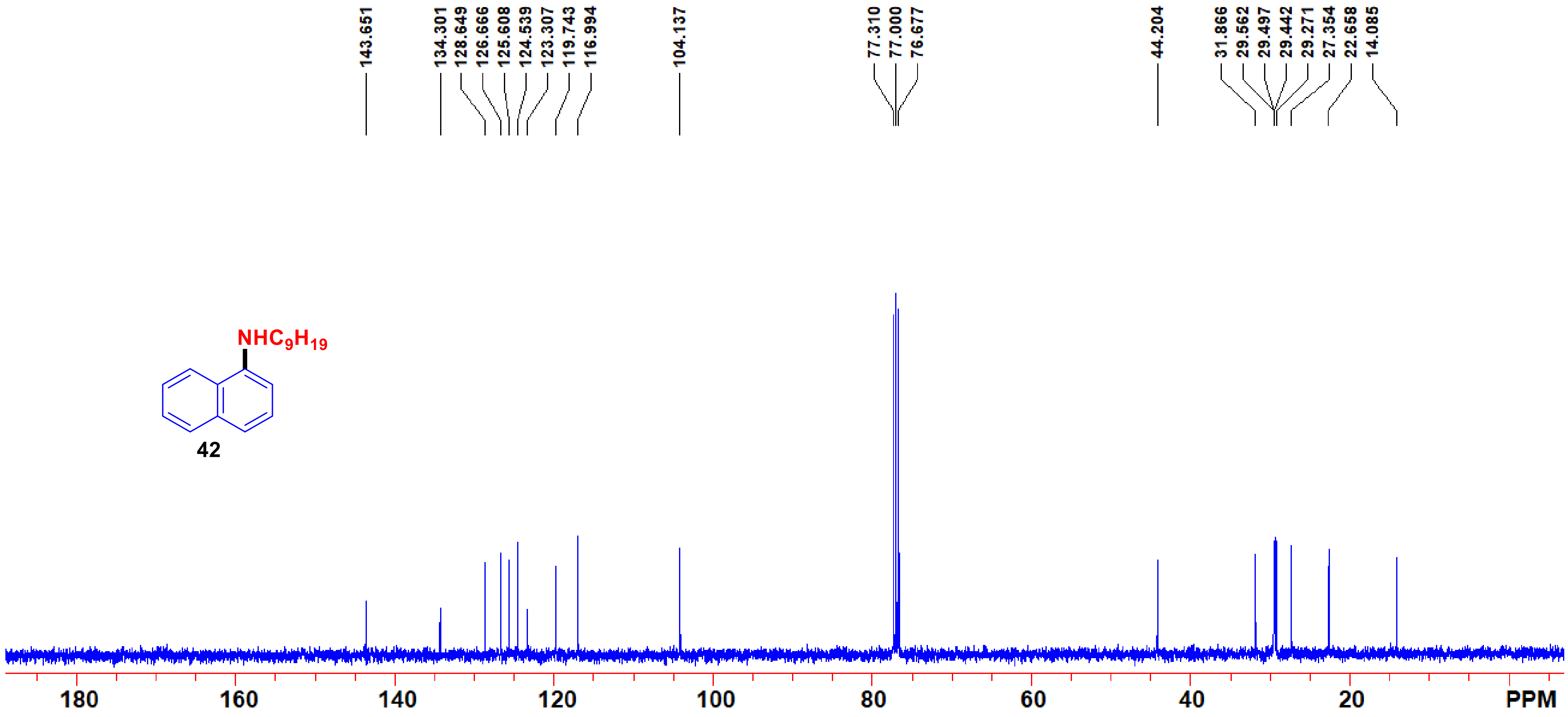
143.651
134.301
128.649
126.666
125.608
124.539
123.307
119.743
116.994
104.137
77.310
77.000
76.677
44.204
31.866
29.562
29.497
29.442
29.271
27.354
22.658
14.085
NHC9H19
42
180
160
140
120
100
80
60
40
20
PPM

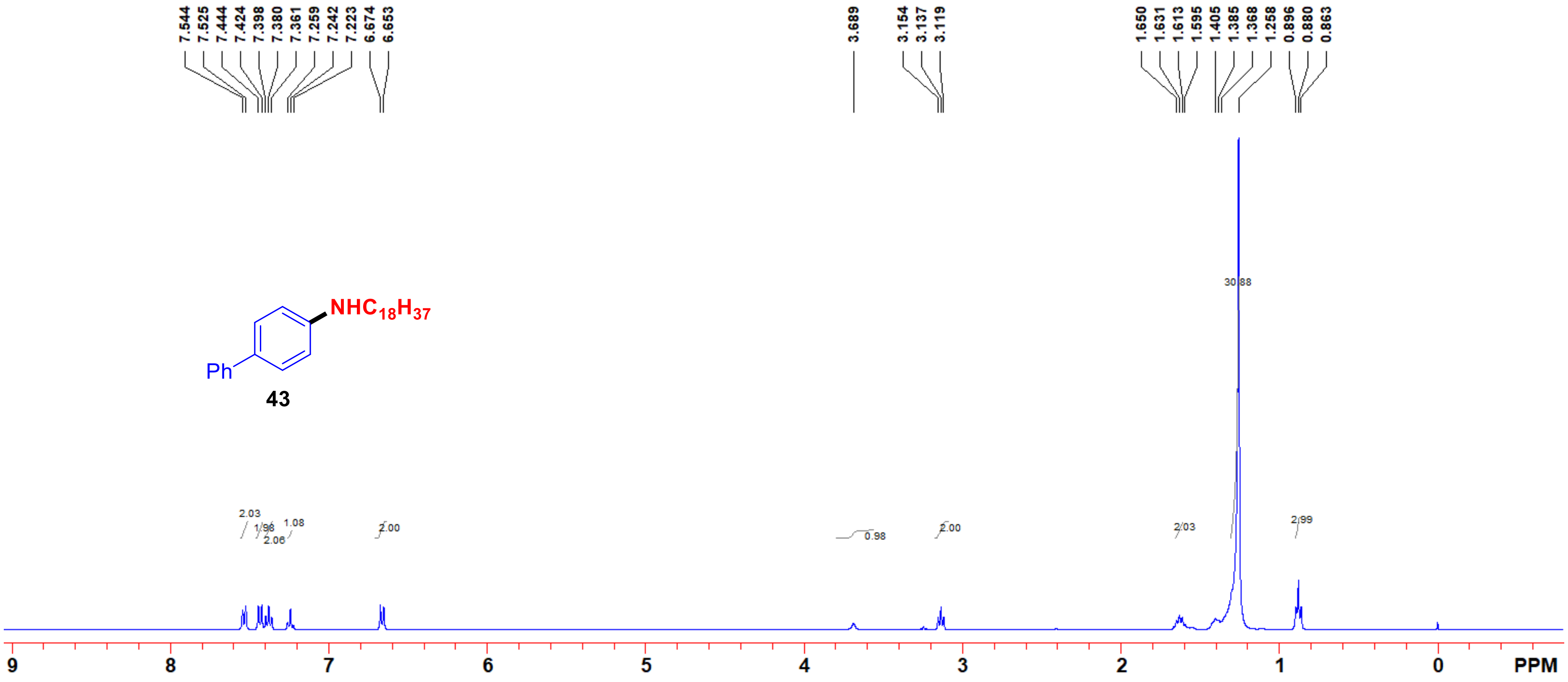
7.544
7.525
7.444
7.424
7.398
7.380
7.361
7.259
7.242
7.223
6.674
6.653
3.689
3.154
3.137
3.119
1.650
1.631
1.613
1.595
1.405
1.385
1.368
1.258
0.896
0.880
0.863
NHC18H37
Ph
43
30.88
2.03
1.98
2.06
1.08
2.00
0.98
2.00
2.03
2.99
9
8
7
6
5
4
3
2
1
0
PPM

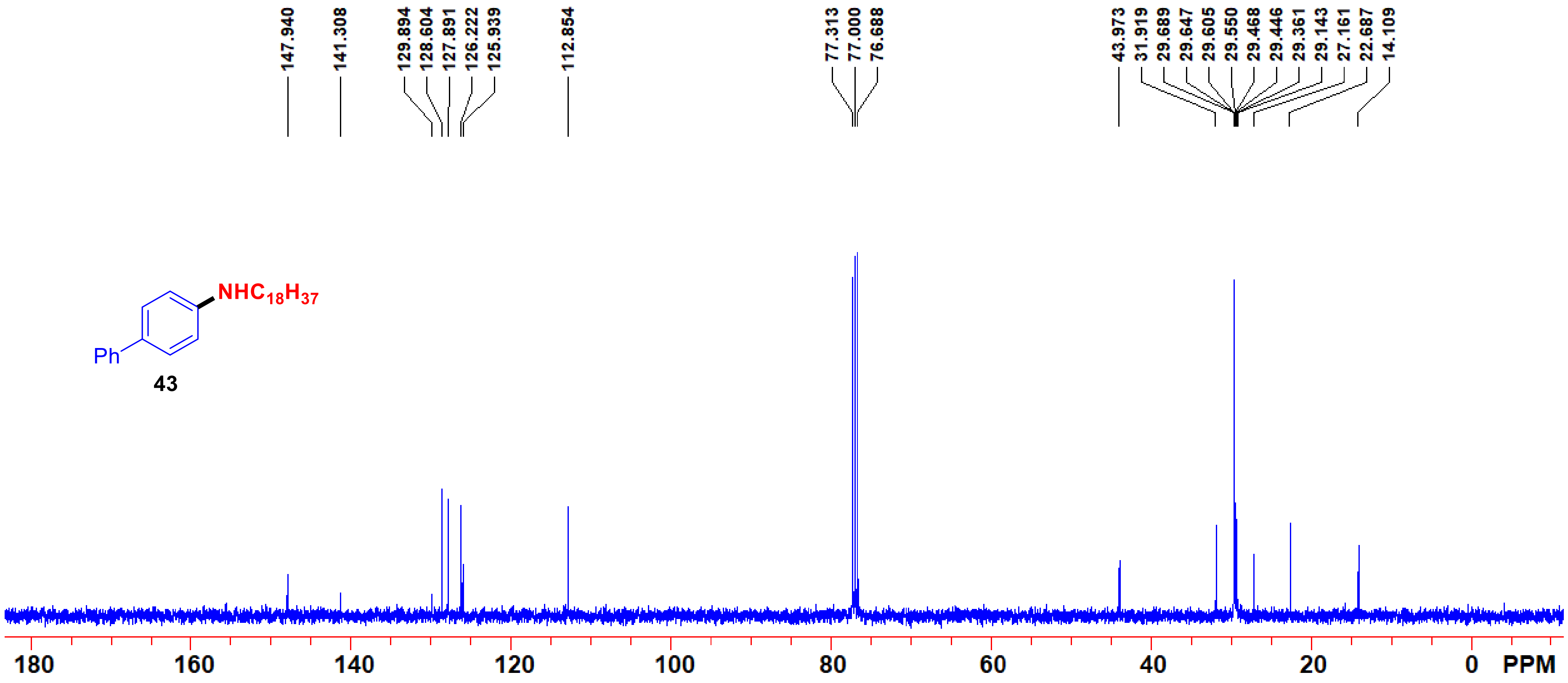
147.940
141.308
129.894
128.604
127.891
126.222
125.939
112.854
77.313
77.000
76.688
43.973
31.919
29.689
29.647
29.605
29.550
29.468
29.446
29.361
29.143
27.161
22.687
14.109
NHC18H37
Ph
43
180
160
140
120
100
80
60
40
20
0
PPM

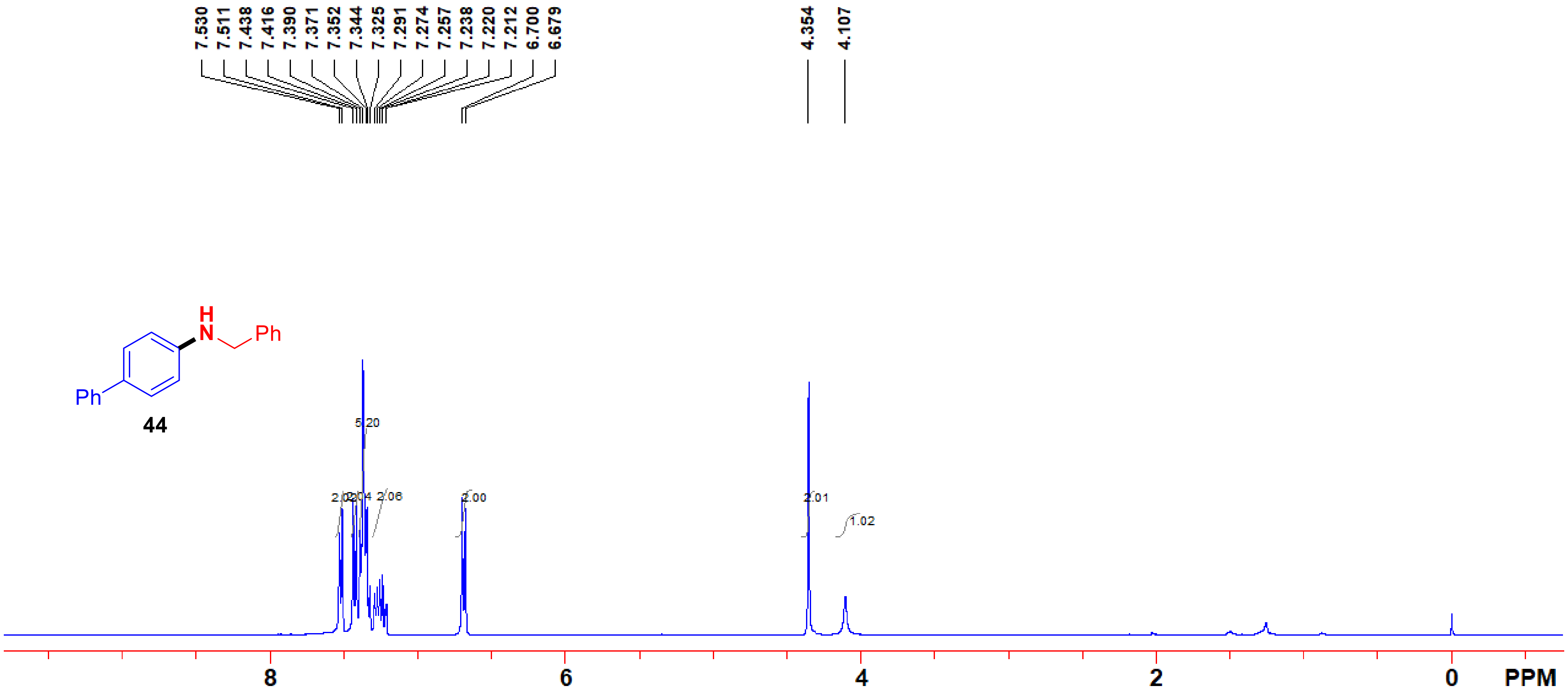

7.530
7.511
7.438
7.416
7.390
7.371
7.352
7.344
7.325
7.291
7.274
7.257
7.238
7.220
7.212
6.700
6.679
4.354
4.107
H
N
Ph
Ph
44
5.20
2.02
2.04
2.06
2.00
2.01
1.02
8
6
4
2
0
PPM

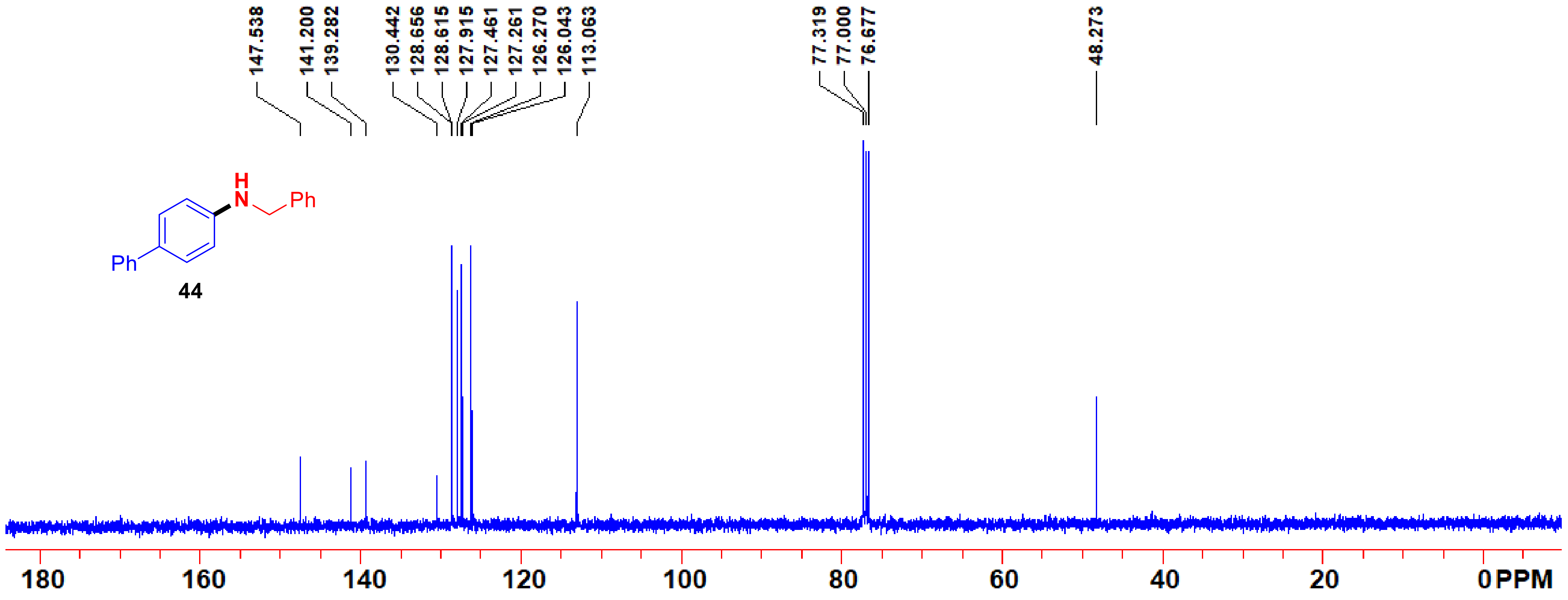
147.538
141.200
139.282
130.442
128.656
128.615
127.915
127.461
127.261
126.270
126.043
113.063
77.319
77.000
76.677
48.273
H
N
Ph
Ph
44
180
160
140
120
100
80
60
40
20
0 PPM

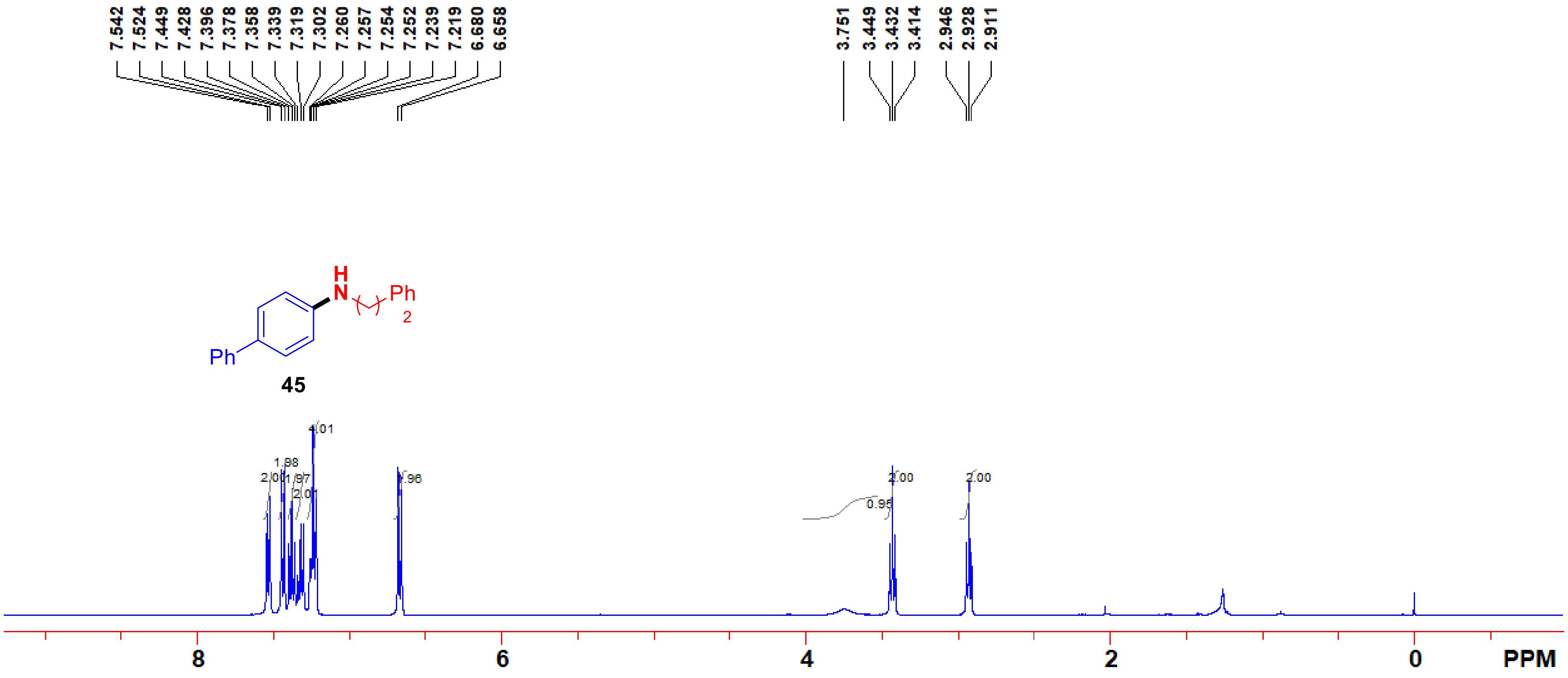
7.542
7.524
7.449
7.428
7.396
7.378
7.358
7.339
7.319
7.302
7.260
7.257
7.254
7.252
7.239
7.219
6.680
6.658
3.751
3.449
3.432
3.414
2.946
2.928
2.911
H
N
Ph
2
Ph
45
2.00
1.98
1.97
2.01
4.01
1.96
0.95
2.00
2.00
8
6
4
2
0
PPM

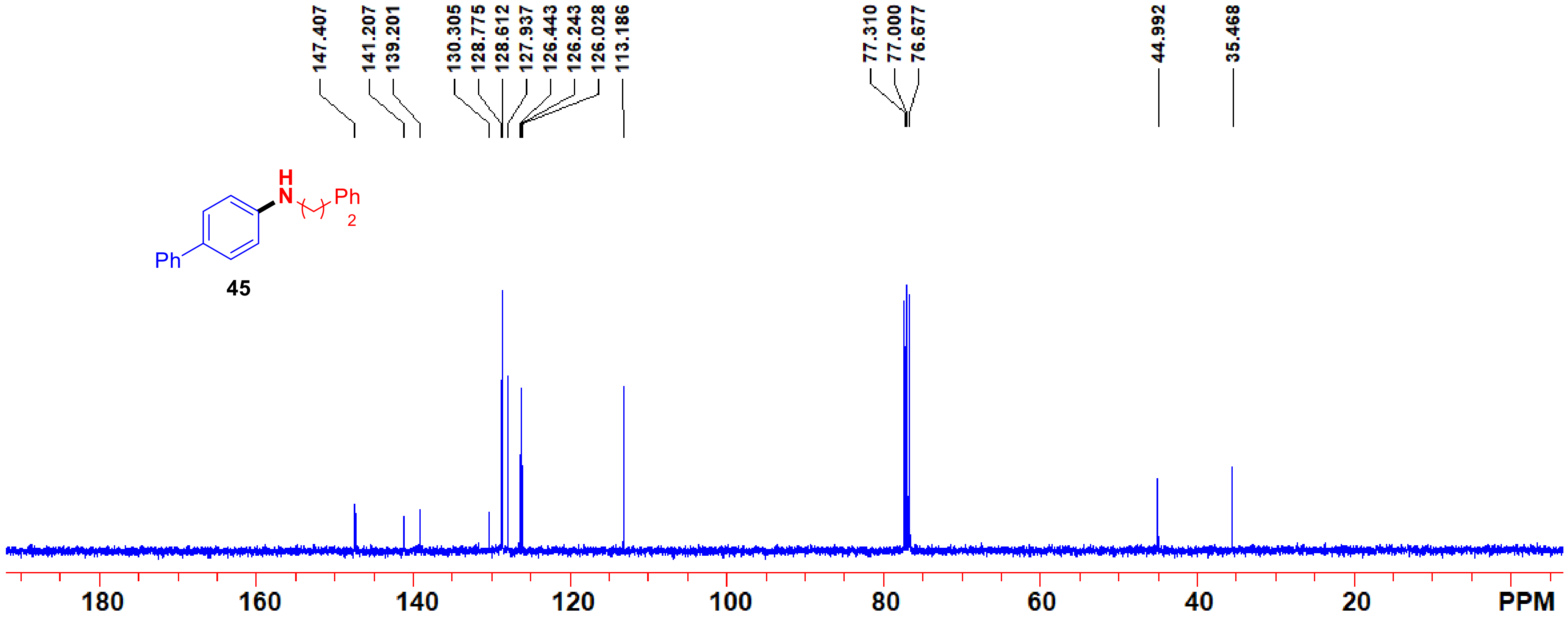

147.407
141.207
139.201
130.305
128.775
128.612
127.937
126.443
126.243
126.028
113.186
77.310
77.000
76.677
44.992
35.468
H
N
Ph
2
Ph
45
180
160
140
120
100
80
60
40
20
PPM

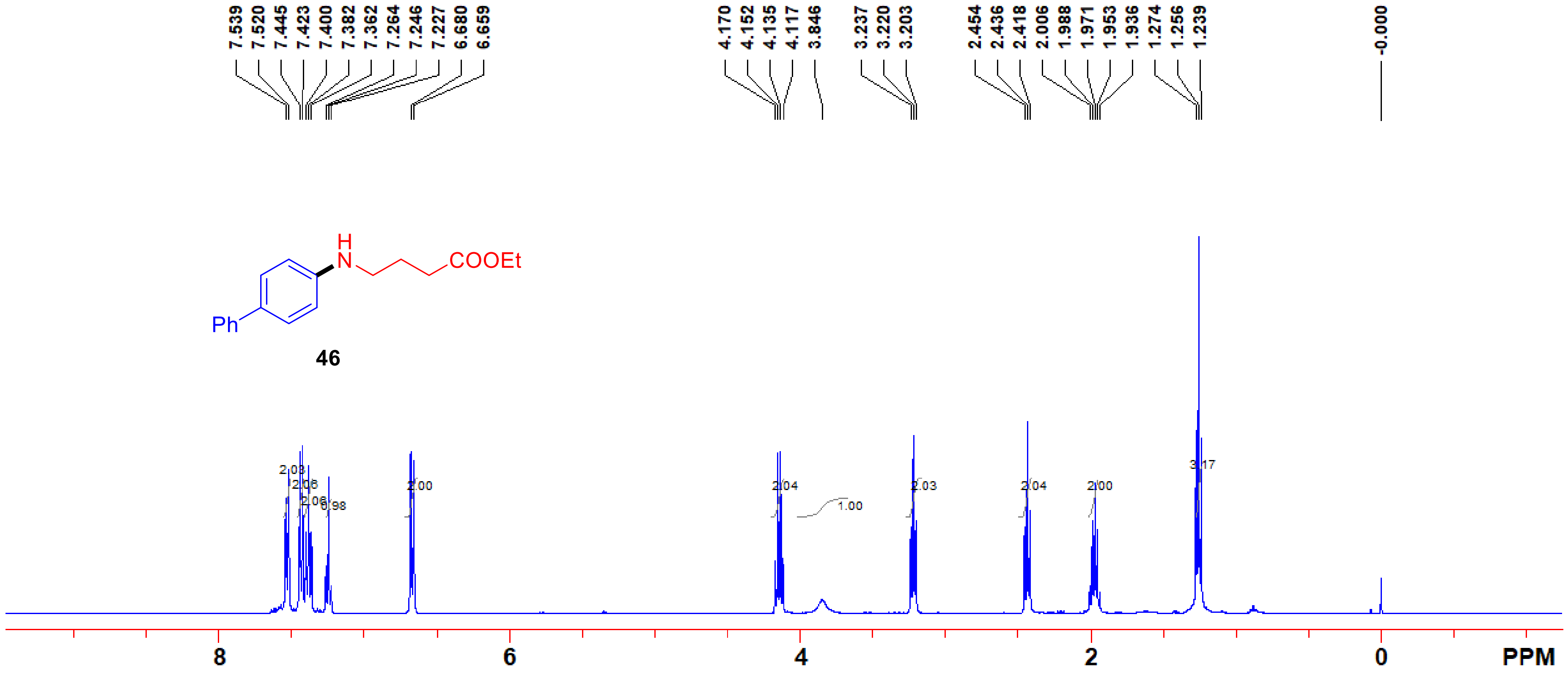
7.539
7.520
7.445
7.423
7.400
7.382
7.362
7.264
7.246
7.227
6.680
6.659
4.170
4.152
4.135
4.117
3.846
3.237
3.220
3.203
2.454
2.436
2.418
2.006
1.988
1.971
1.953
1.936
1.274
1.256
1.239
-0.000
H
N
COOEt
Ph
46
2.03
2.06
2.06
0.98
2.00
2.04
1.00
2.03
2.04
2.00
3.17
8
6
4
2
0
PPM

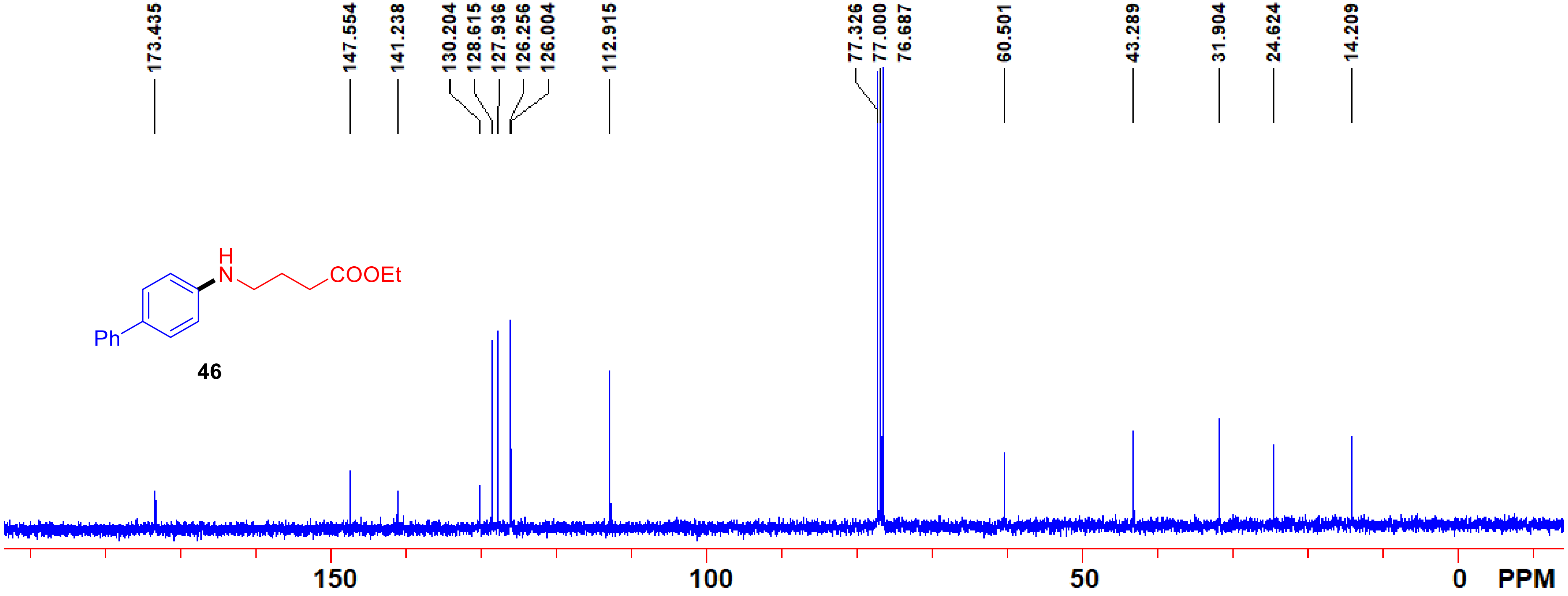
173.435
147.554
141.238
130.204
128.615
127.936
126.256
126.004
112.915
77.326
77.000
76.687
60.501
43.289
31.904
24.624
14.209
H
N
COOEt
Ph
46
150
100
50
0
PPM

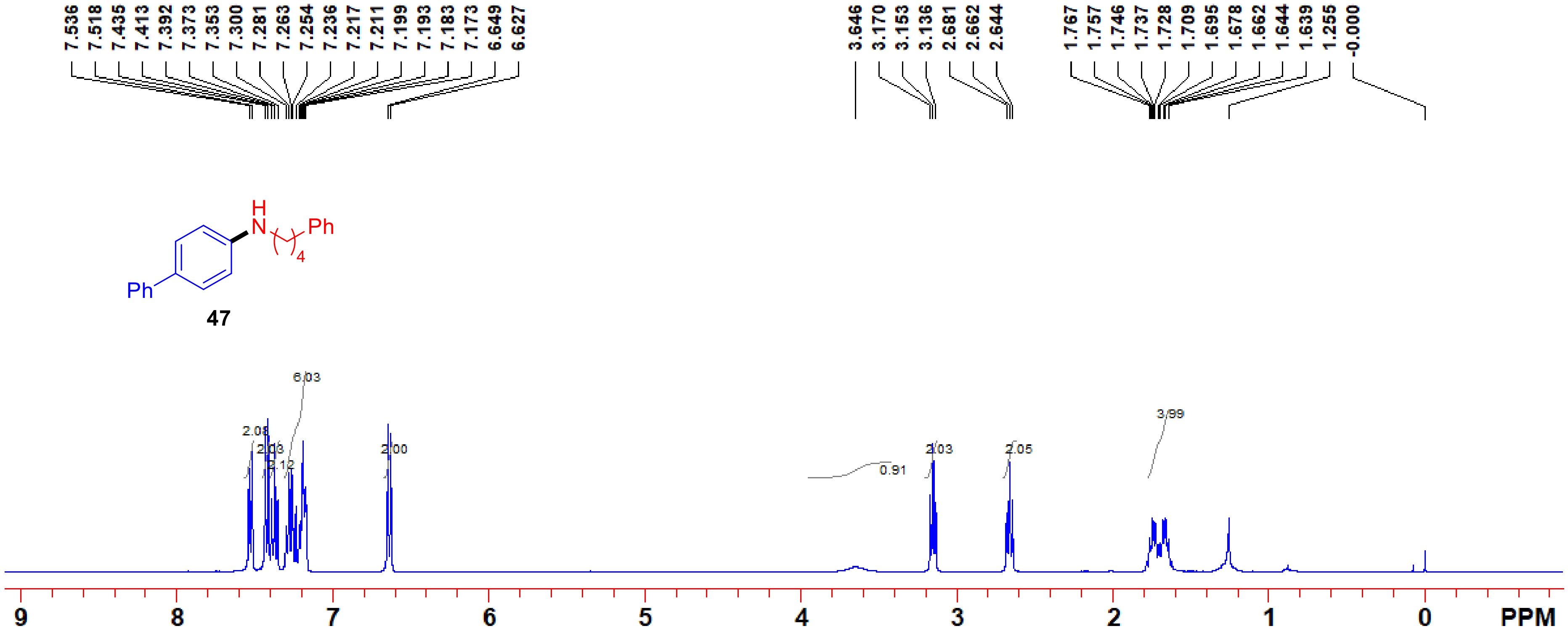
7.536
7.518
7.435
7.413
7.392
7.373
7.353
7.300
7.281
7.263
7.254
7.236
7.217
7.211
7.199
7.193
7.183
7.173
6.649
6.627
3.646
3.170
3.153
3.136
2.681
2.662
2.644
1.767
1.757
1.746
1.737
1.728
1.709
1.695
1.678
1.662
1.644
1.639
1.255
-0.000
Ph
N
H
Ph
4
47
2.08
2.03
2.12
6.03
2.00
0.91
2.03
2.05
3.99
9
8
7
6
5
4
3
2
1
0
PPM

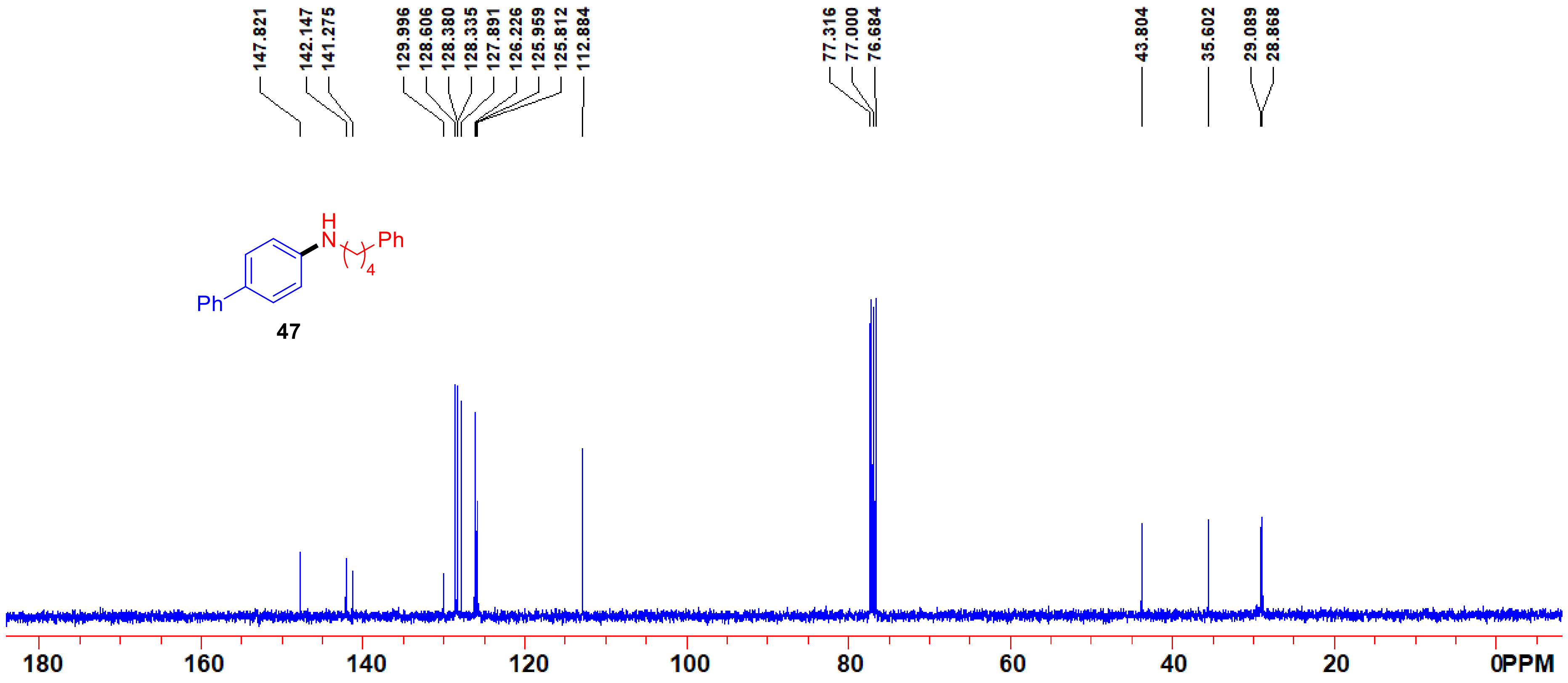
147.821
142.147
141.275
129.996
128.606
128.380
128.335
127.891
126.226
125.959
125.812
112.884
77.316
77.000
76.684
43.804
35.602
29.089
28.868
H
N
Ph
4
Ph
47
180
160
140
120
100
80
60
40
20
0PPM

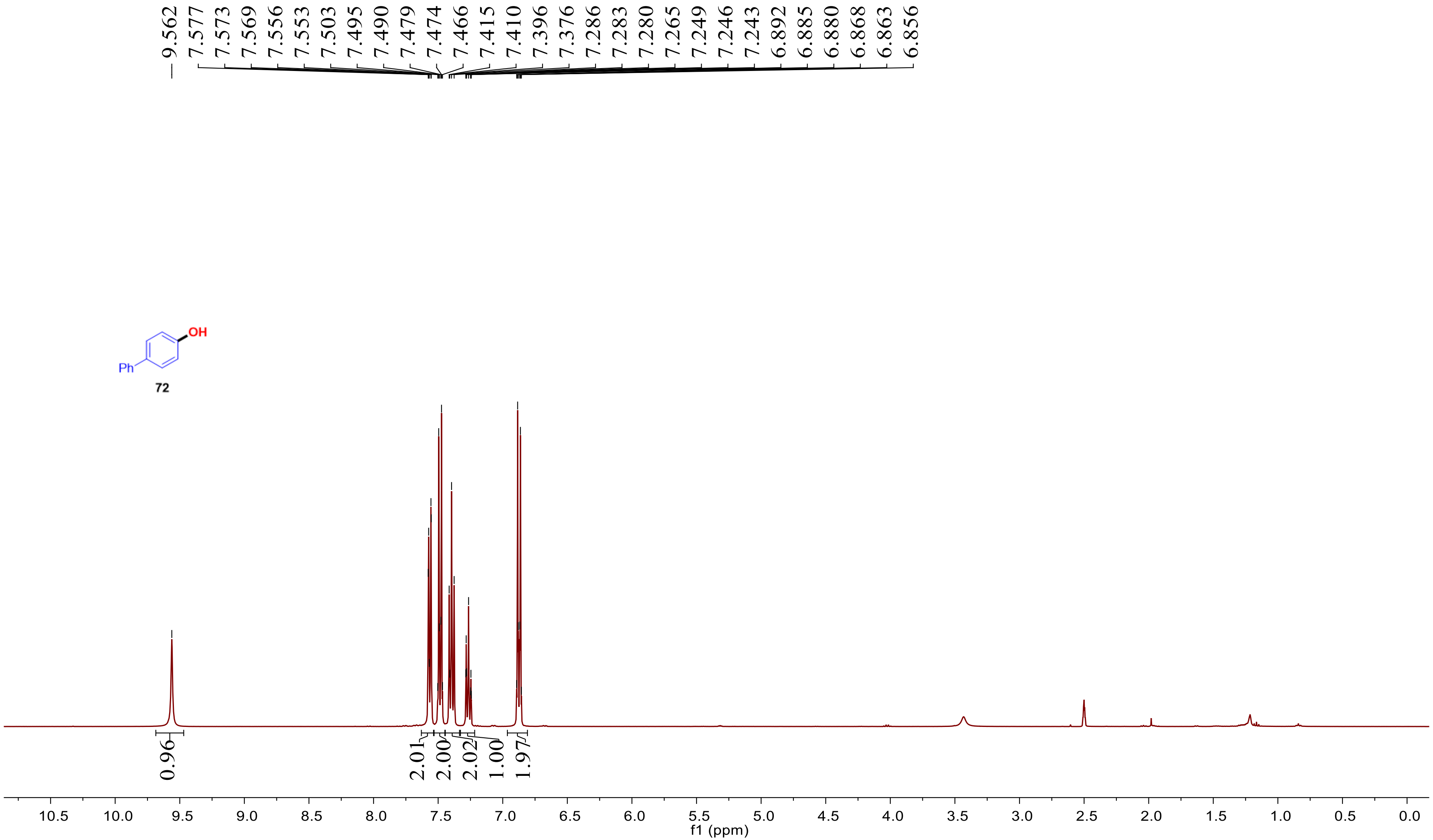
9.562
7.577
7.573
7.569
7.556
7.553
7.503
7.495
7.490
7.479
7.474
7.466
7.415
7.410
7.396
7.376
7.286
7.283
7.280
7.265
7.249
7.246
7.243
6.892
6.885
6.880
6.868
6.863
6.856
OH
Ph
72
0.96
2.01
2.00
2.02
1.00
1.97
10.5
10.0
9.5
9.0
8.5
8.0
7.5
7.0
6.5
6.0
5.5
5.0
4.5
4.0
3.5
3.0
2.5
2.0
1.5
1.0
0.5
0.0
f1 (ppm)

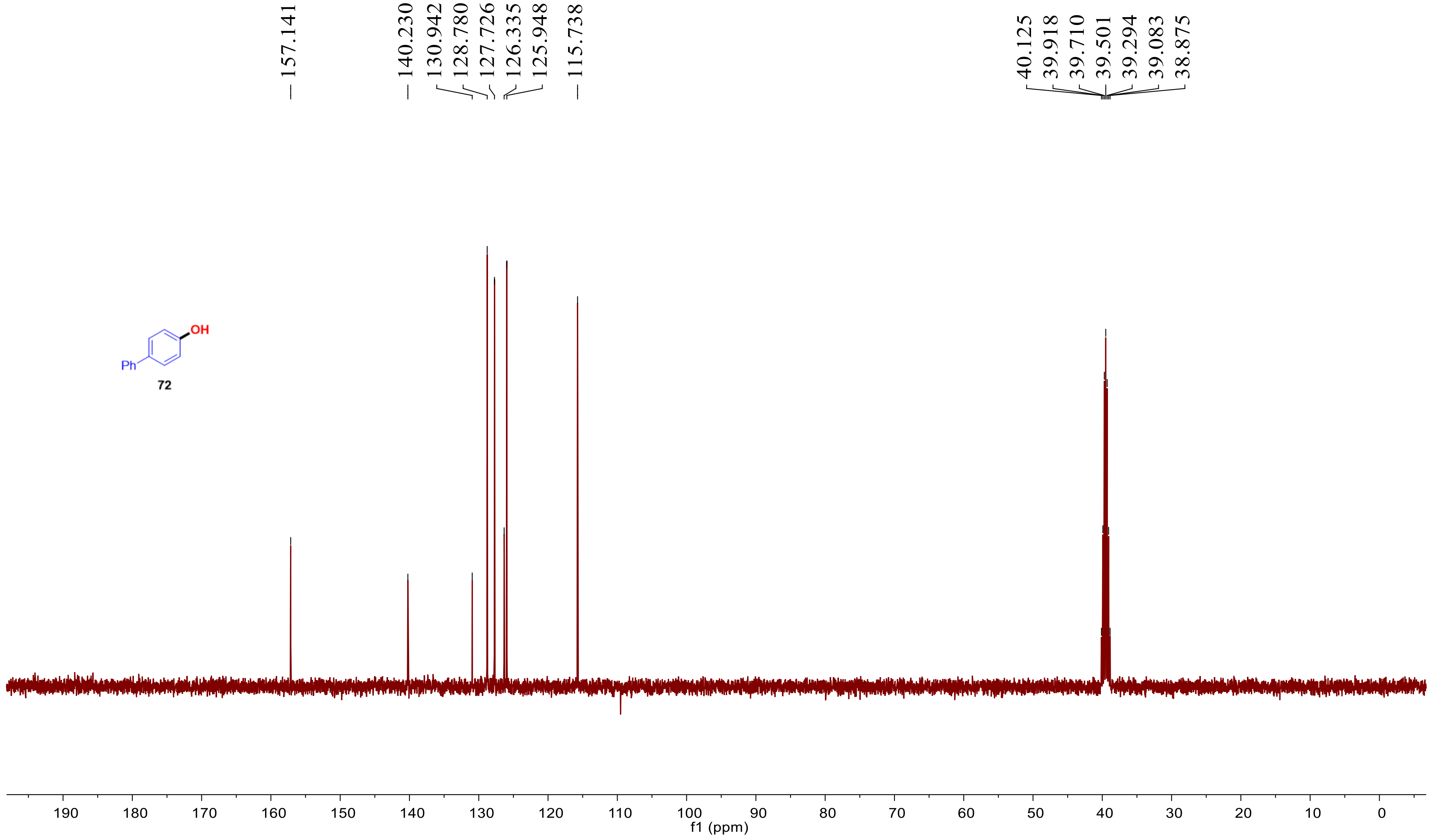
157.141
140.230
130.942
128.780
127.726
126.335
125.948
115.738
40.125
39.918
39.710
39.501
39.294
39.083
38.875
OH
Ph
72
190 180 170 160 150 140 130 120 110 100 90 80 70 60 50 40 30 20 10 0
f1 (ppm)

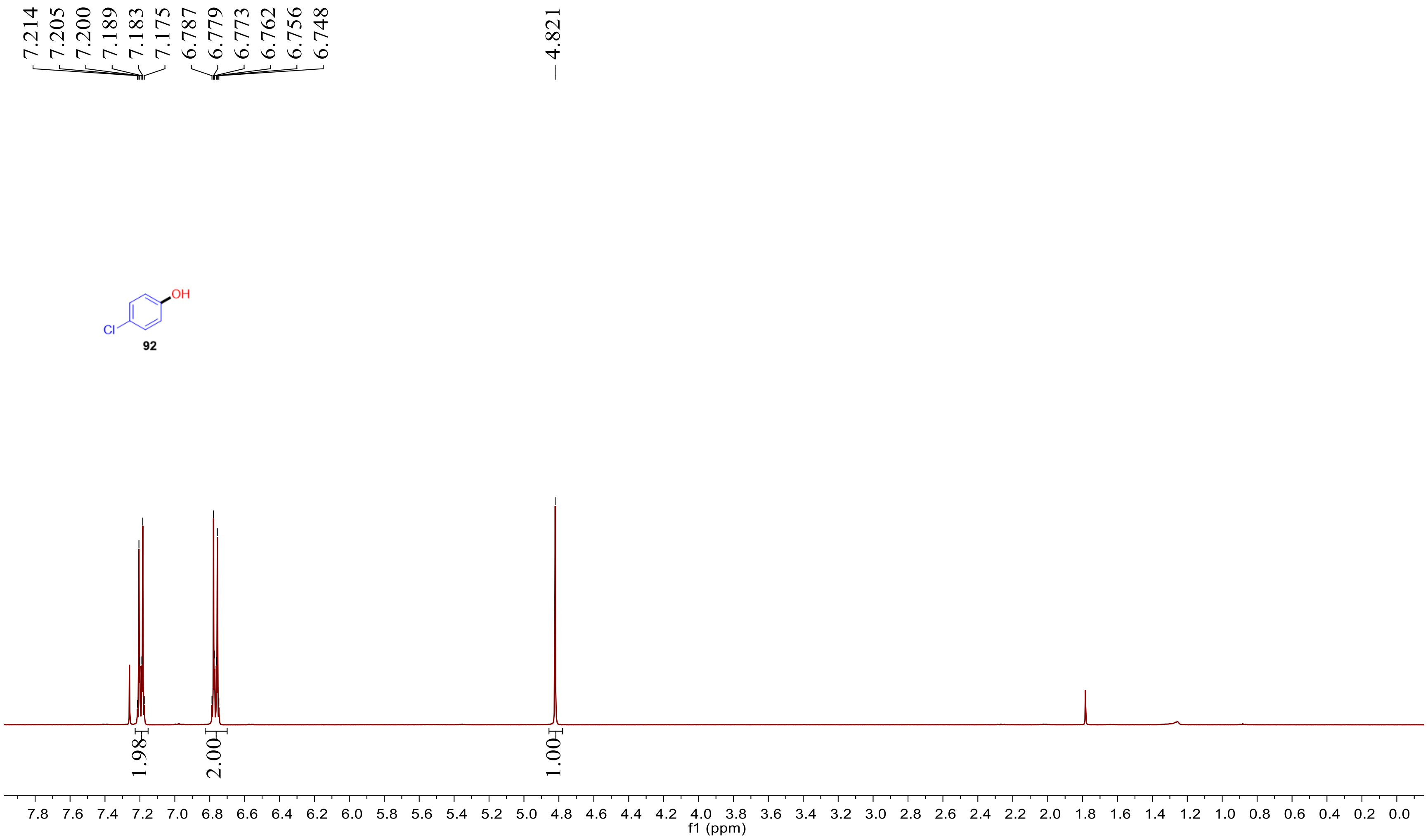
7.214
7.205
7.200
7.189
7.183
7.175
6.787
6.779
6.773
6.762
6.756
6.748
4.821
OH
Cl
92
1.98
2.00
1.00
7.8 7.6 7.4 7.2 7.0 6.8 6.6 6.4 6.2 6.0 5.8 5.6 5.4 5.2 5.0 4.8 4.6 4.4 4.2 4.0 3.8 3.6 3.4 3.2 3.0 2.8 2.6 2.4 2.2 2.0 1.8 1.6 1.4 1.2 1.0 0.8 0.6 0.4 0.2 0.0
f1 (ppm)

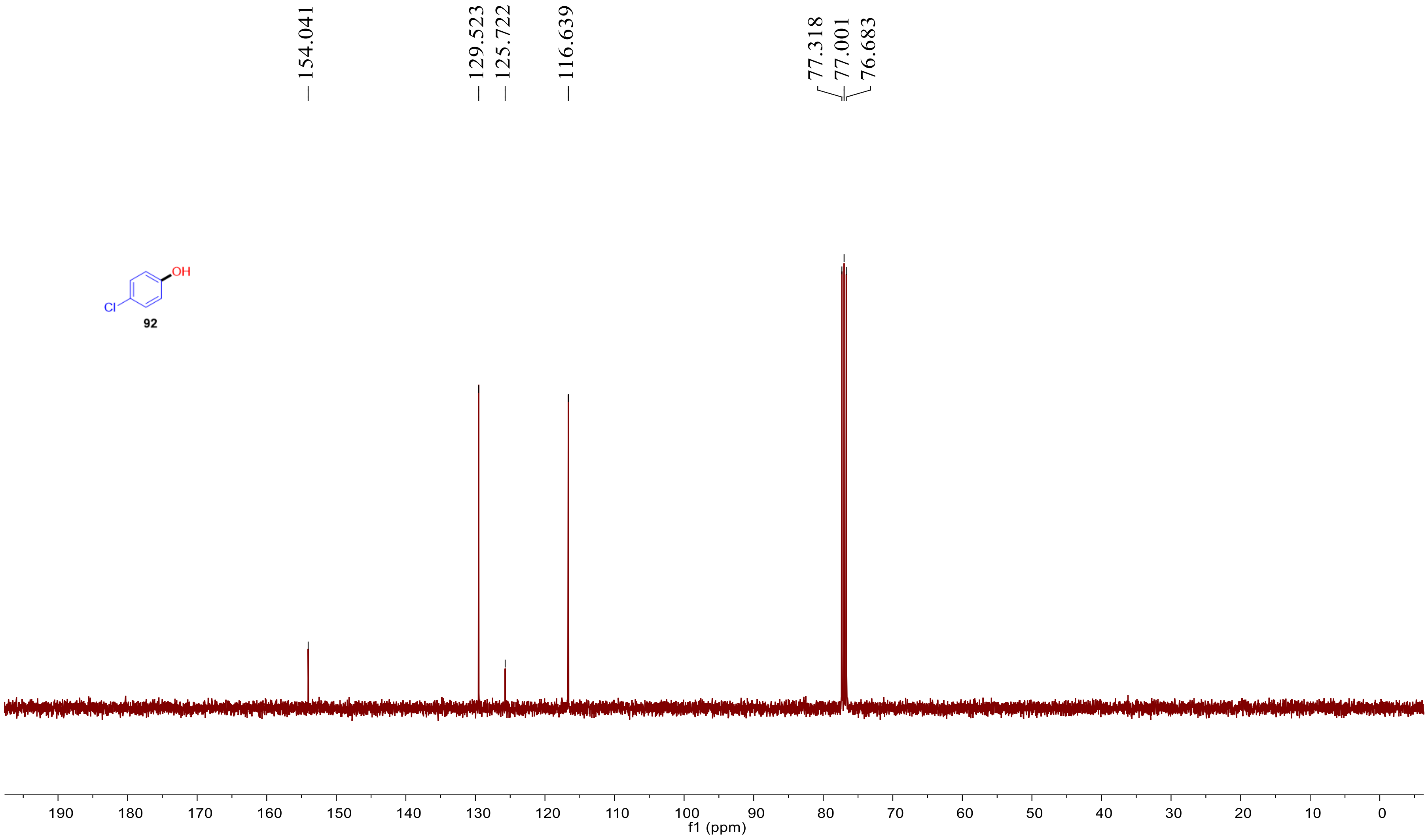

154.041
129.523
125.722
116.639
77.318
77.001
76.683
OH
Cl
190
180
170
160
150
140
130
120
110
100
90
80
70
60
50
40
30
20
10
0
f1 (ppm)

92

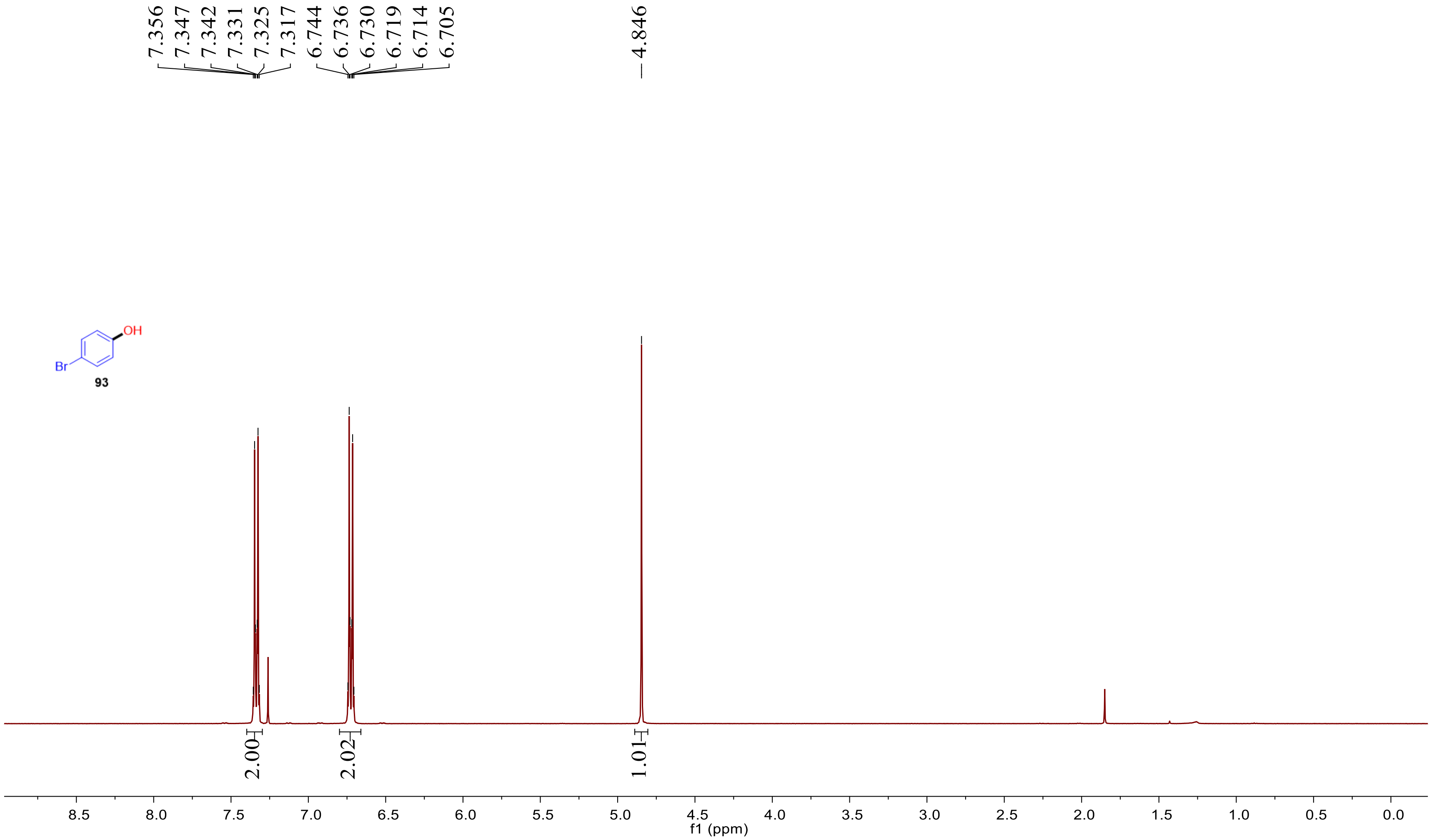
7.356
7.347
7.342
7.331
7.325
7.317
6.744
6.736
6.730
6.719
6.714
6.705
4.846
OH
Br
93
2.00
2.02
1.01
8.5
8.0
7.5
7.0
6.5
6.0
5.5
5.0
4.5
4.0
3.5
3.0
2.5
2.0
1.5
1.0
0.5
0.0
f1 (ppm)

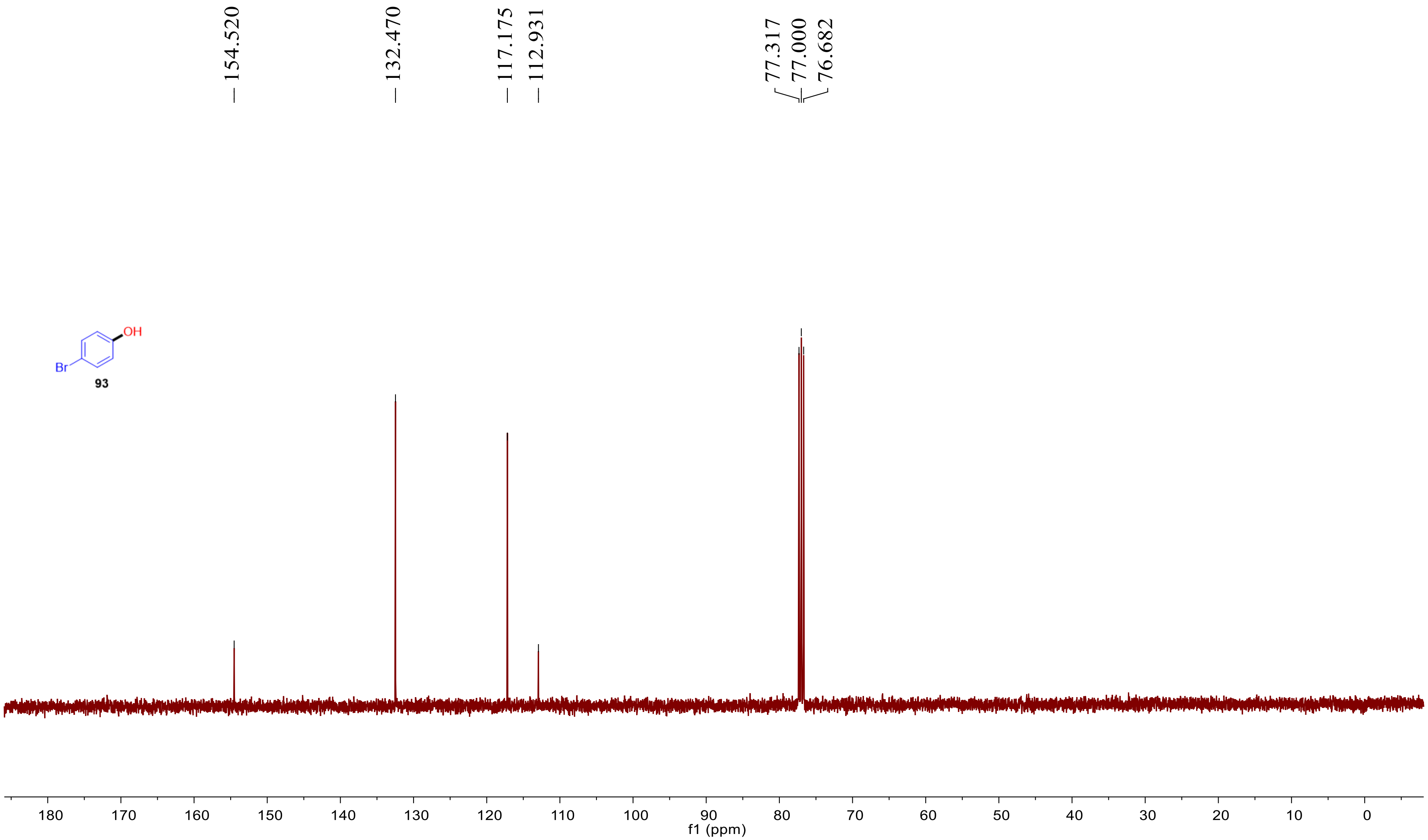

154.520
132.470
117.175
112.931
77.317
77.000
76.682
OH
Br
93
180
170
160
150
140
130
120
110
100
90
80
70
60
50
40
30
20
10
0
f1 (ppm)

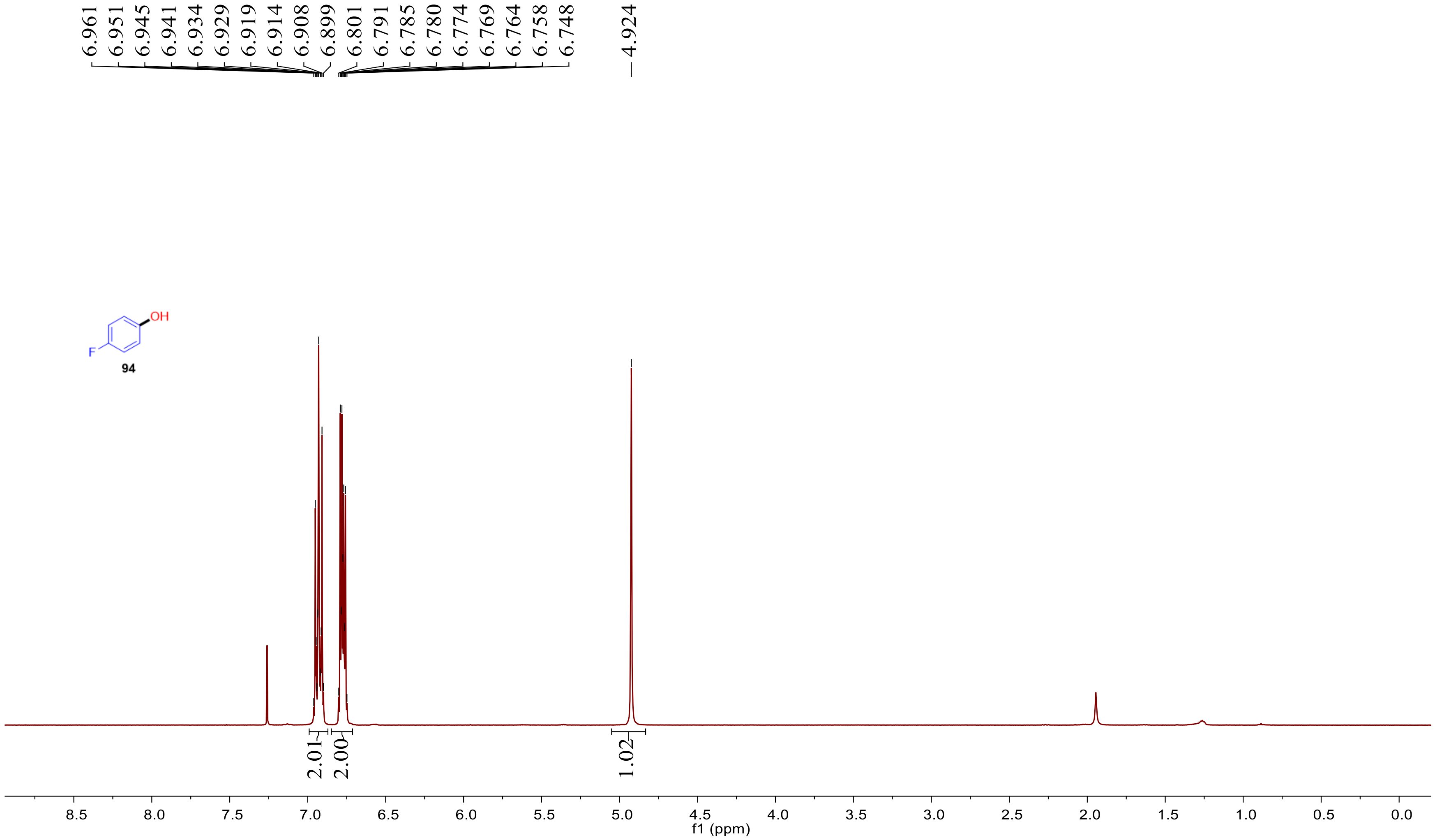
6.961
6.951
6.945
6.941
6.934
6.929
6.919
6.914
6.908
6.899
6.801
6.791
6.785
6.780
6.774
6.769
6.764
6.758
6.748
4.924
OH
F
94
2.01
2.00
1.02
8.5
8.0
7.5
7.0
6.5
6.0
5.5
5.0
4.5
4.0
3.5
3.0
2.5
2.0
1.5
1.0
0.5
0.0
f1 (ppm)

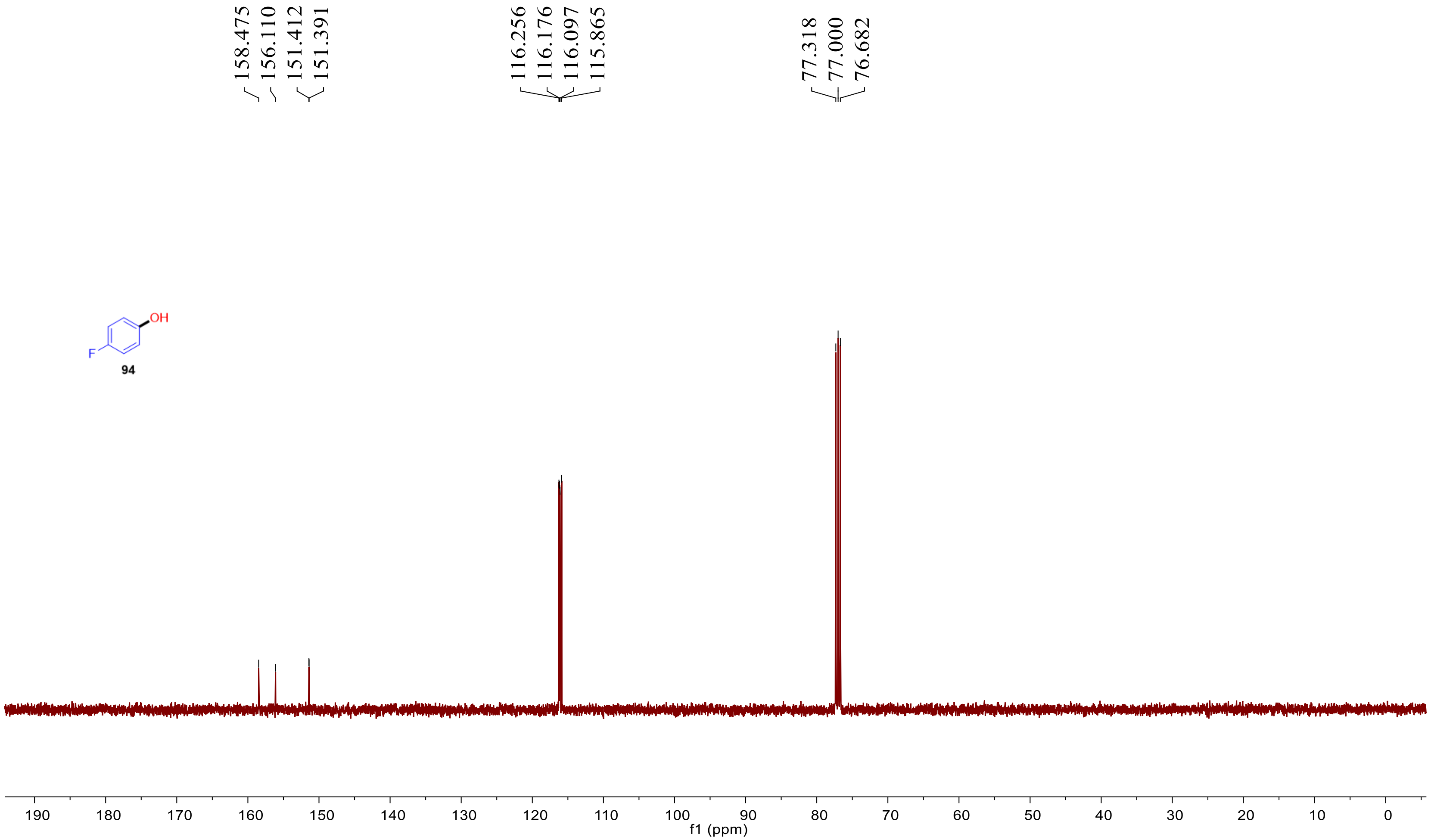

158.475
156.110
151.412
151.391
116.256
116.176
116.097
115.865
77.318
77.000
76.682
OH
F
94
190 180 170 160 150 140 130 120 110 100 90 80 70 60 50 40 30 20 10 0
f1 (ppm)

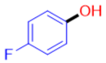

94

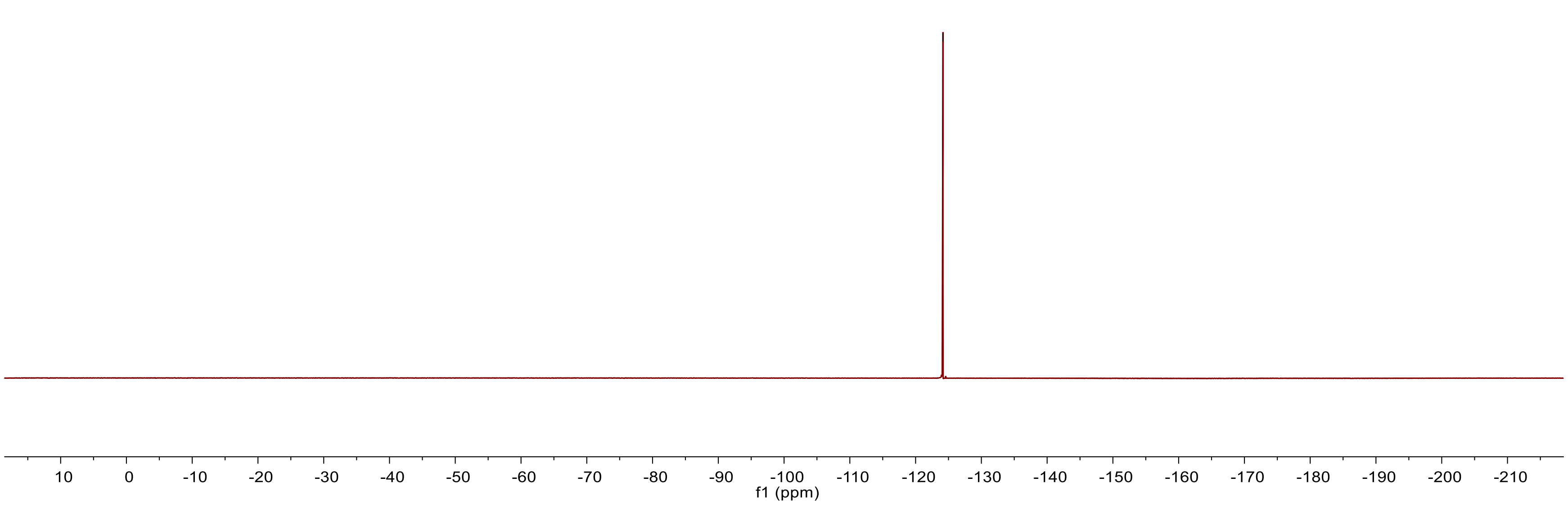

-124.162
10 0 -10 -20 -30 -40 -50 -60 -70 -80 -90 -100 -110 -120 -130 -140 -150 -160 -170 -180 -190 -200 -210
f1 (ppm)

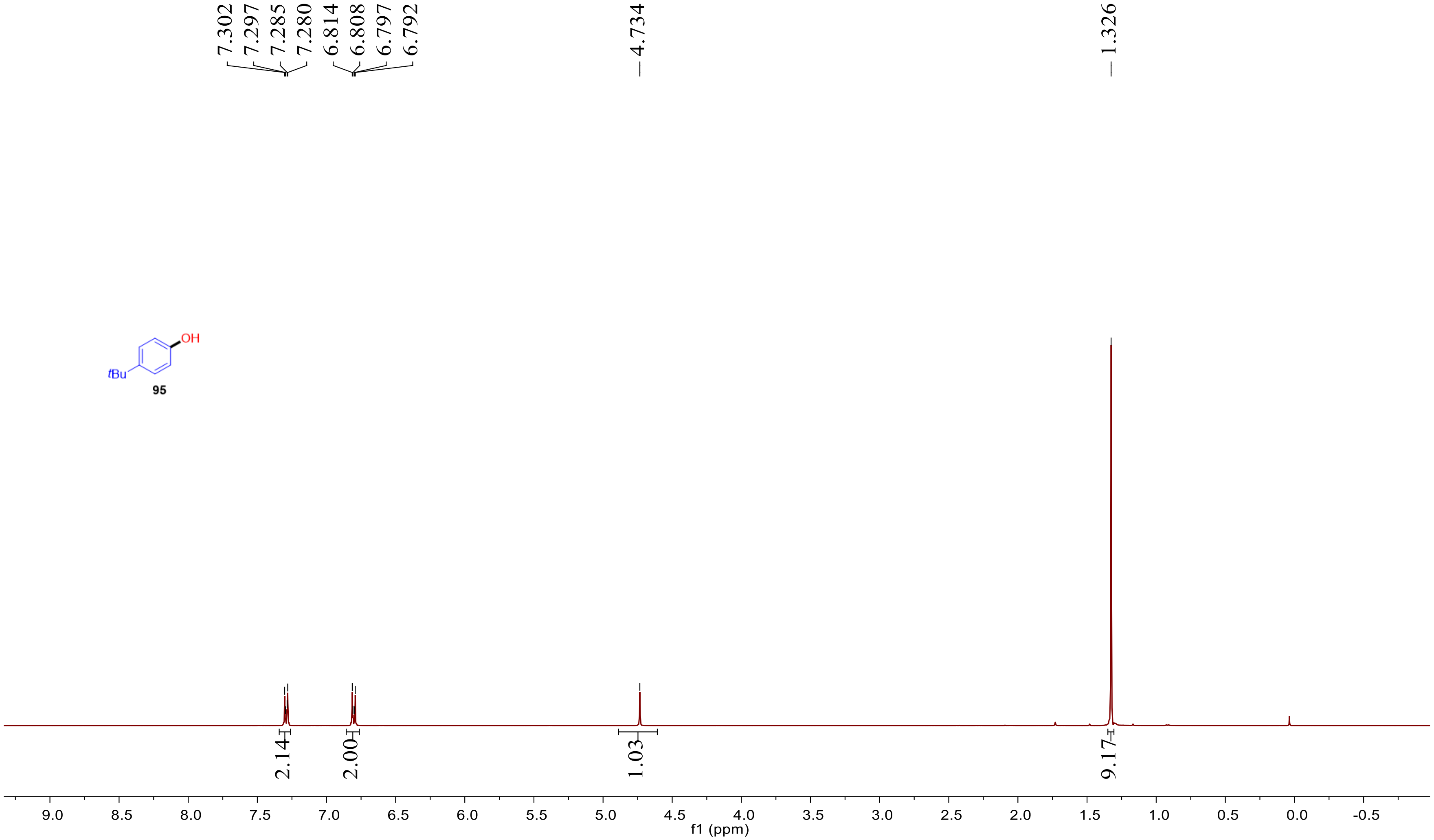
7.302
7.297
7.285
7.280
6.814
6.808
6.797
6.792
4.734
1.326
OH
tBu
95
2.14
2.00
1.03
9.17
9.0 8.5 8.0 7.5 7.0 6.5 6.0 5.5 5.0 4.5 4.0 3.5 3.0 2.5 2.0 1.5 1.0 0.5 0.0 -0.5
f1 (ppm)

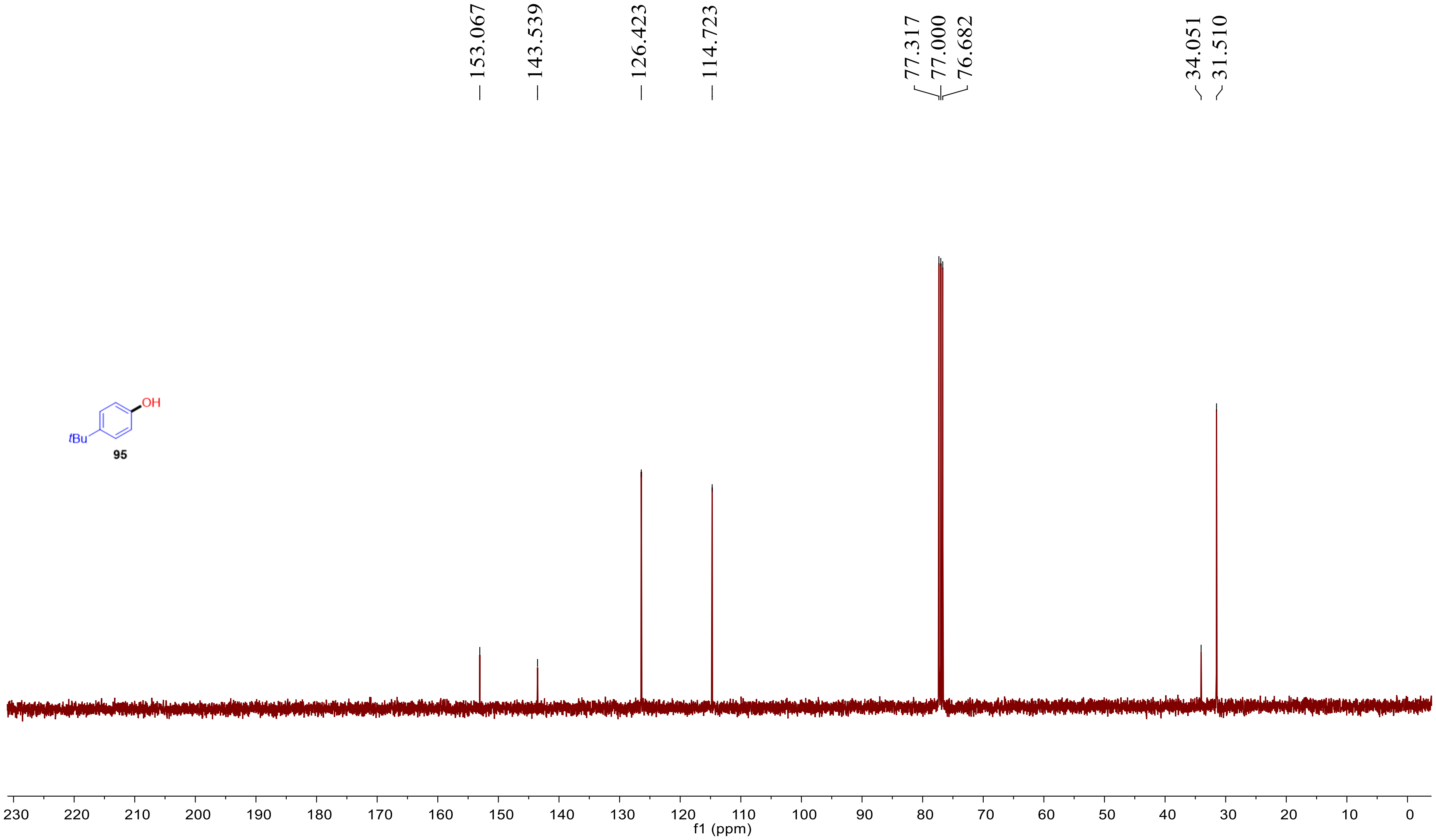
153.067
143.539
126.423
114.723
77.317
77.000
76.682
34.051
31.510
230 220 210 200 190 180 170 160 150 140 130 120 110 100 90 80 70 60 50 40 30 20 10 0
f1 (ppm)
tBu
OH
95

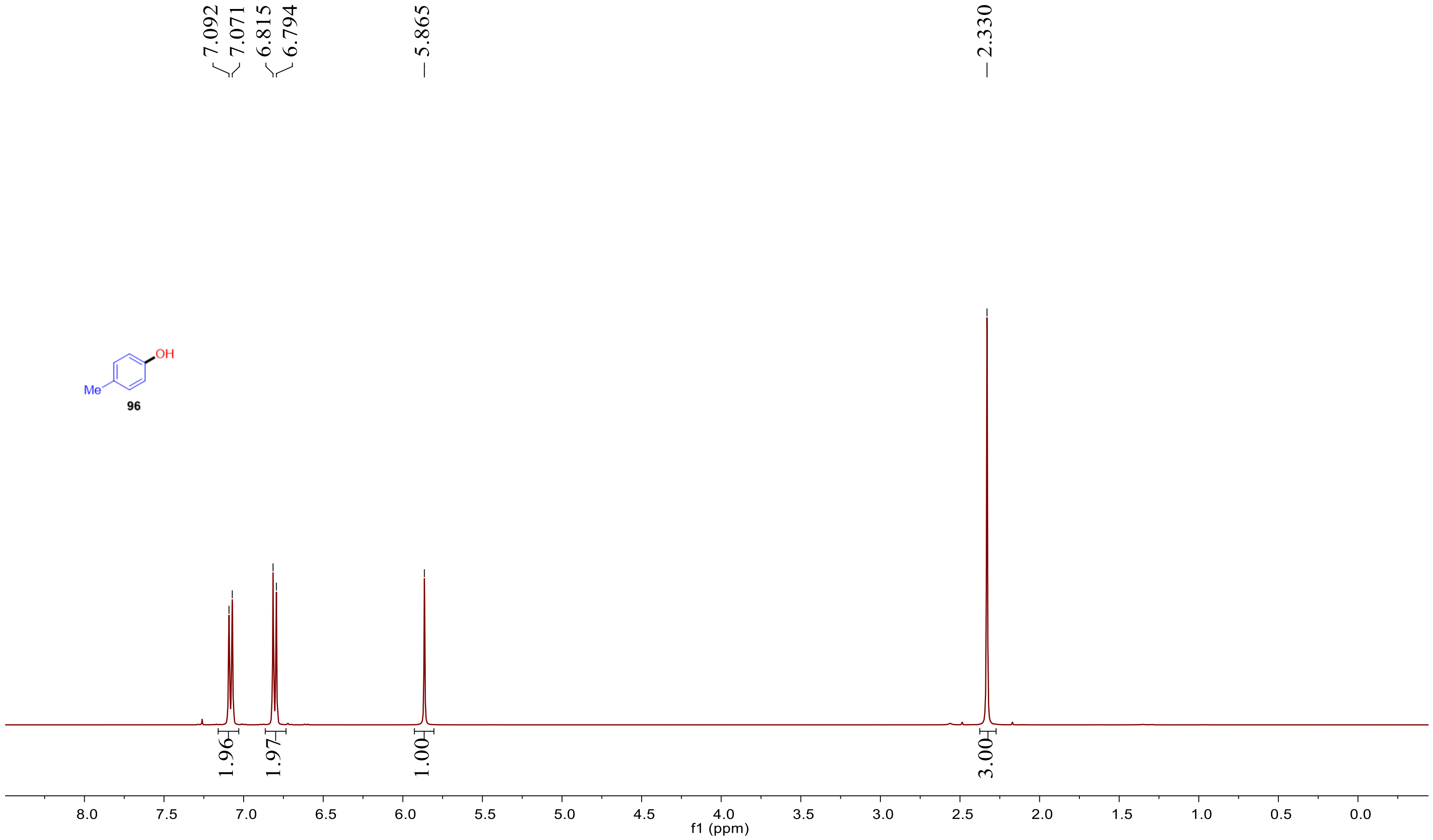
7.092
7.071
6.815
6.794
5.865
2.330
OH
Me
96
1.96
1.97
1.00
3.00
8.0 7.5 7.0 6.5 6.0 5.5 5.0 4.5 4.0 3.5 3.0 2.5 2.0 1.5 1.0 0.5 0.0
f1 (ppm)

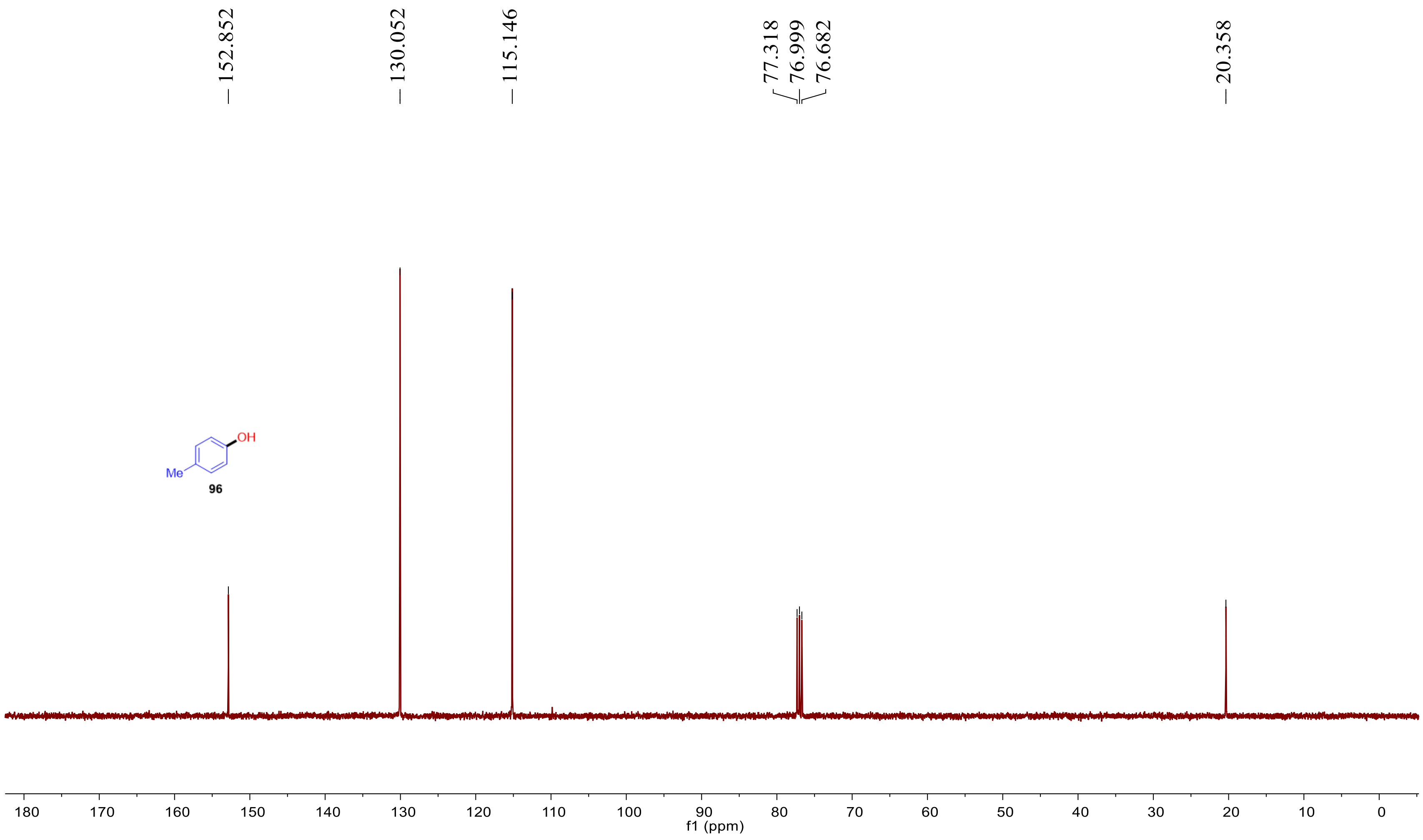

152.852
130.052
115.146
77.318
76.999
76.682
20.358
OH
Me
96
180 170 160 150 140 130 120 110 100 90 80 70 60 50 40 30 20 10 0
f1 (ppm)

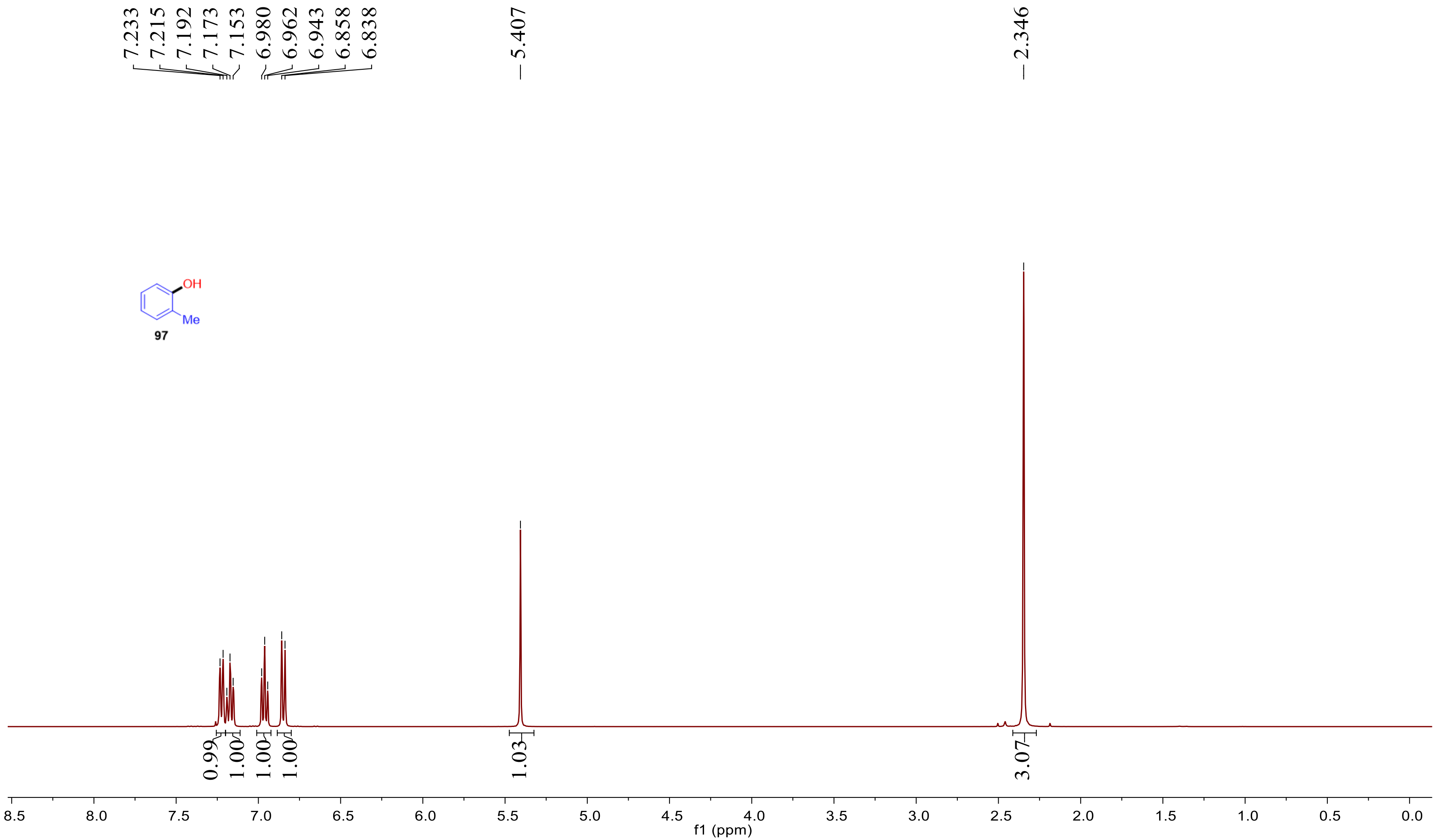
7.233
7.215
7.192
7.173
7.153
6.980
6.962
6.943
6.858
6.838
5.407
2.346
OH
Me
97
0.99
1.00
1.00
1.00
1.03
3.07
8.5
8.0
7.5
7.0
6.5
6.0
5.5
5.0
4.5
4.0
3.5
3.0
2.5
2.0
1.5
1.0
0.5
0.0
f1 (ppm)

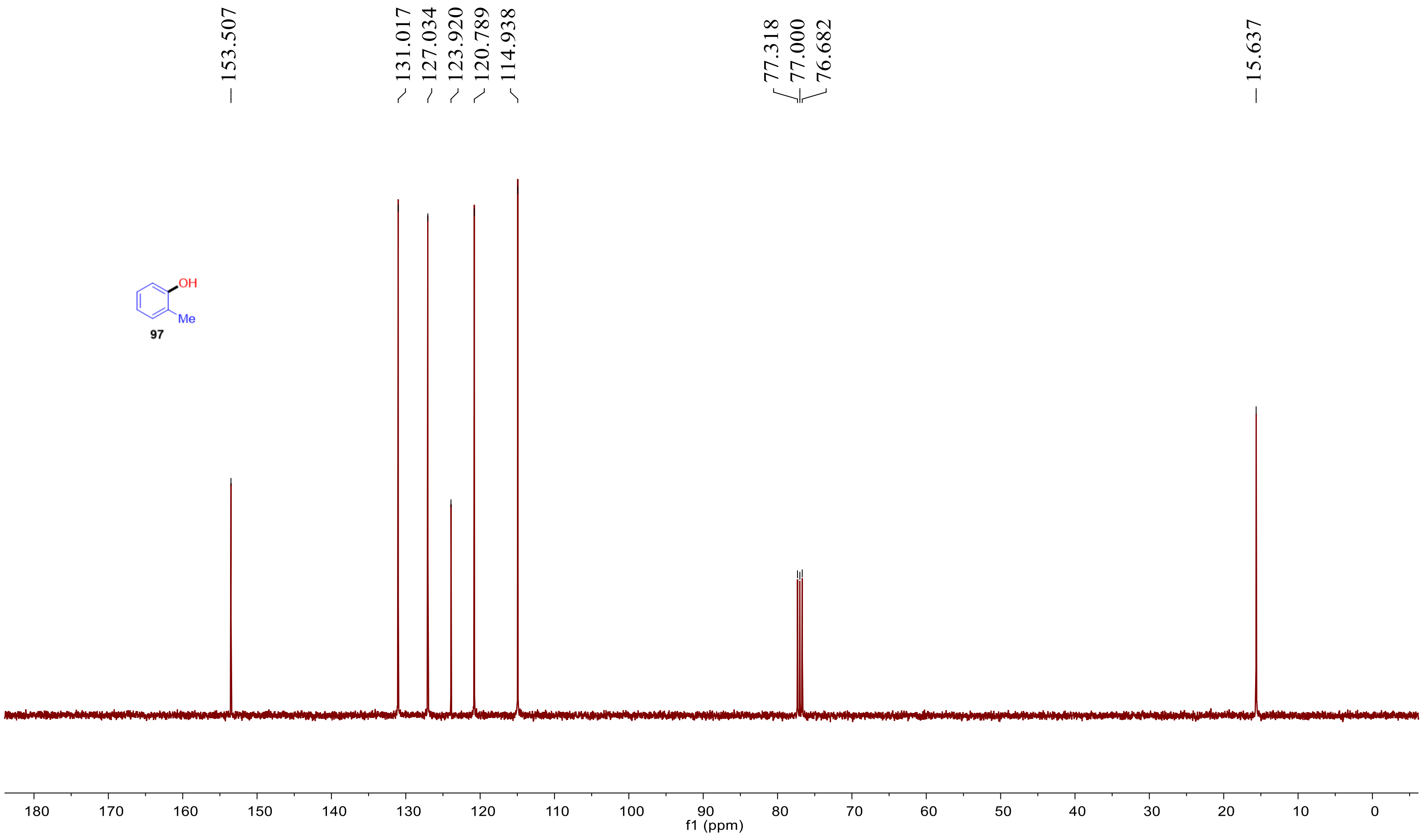
153.507
131.017
127.034
123.920
120.789
114.938
77.318
77.000
76.682
15.637
OH
Me
97
180 170 160 150 140 130 120 110 100 90 80 70 60 50 40 30 20 10 0
f1 (ppm)

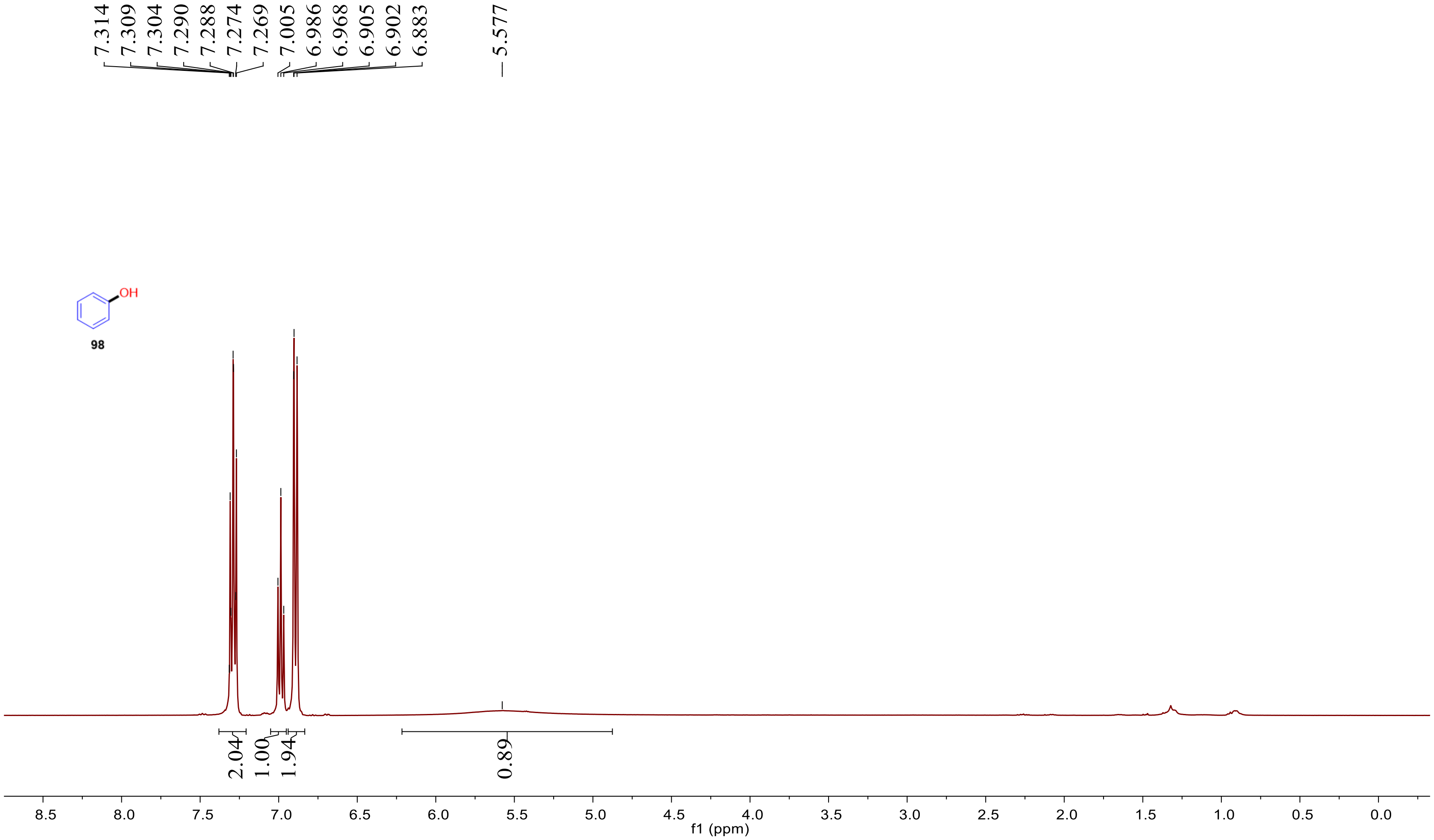
7.314
7.309
7.304
7.290
7.288
7.274
7.269
7.005
6.986
6.968
6.905
6.902
6.883
5.577
OH
98
2.04
1.00
1.94
0.89
8.5
8.0
7.5
7.0
6.5
6.0
5.5
5.0
4.5
4.0
3.5
3.0
2.5
2.0
1.5
1.0
0.5
0.0
f1 (ppm)

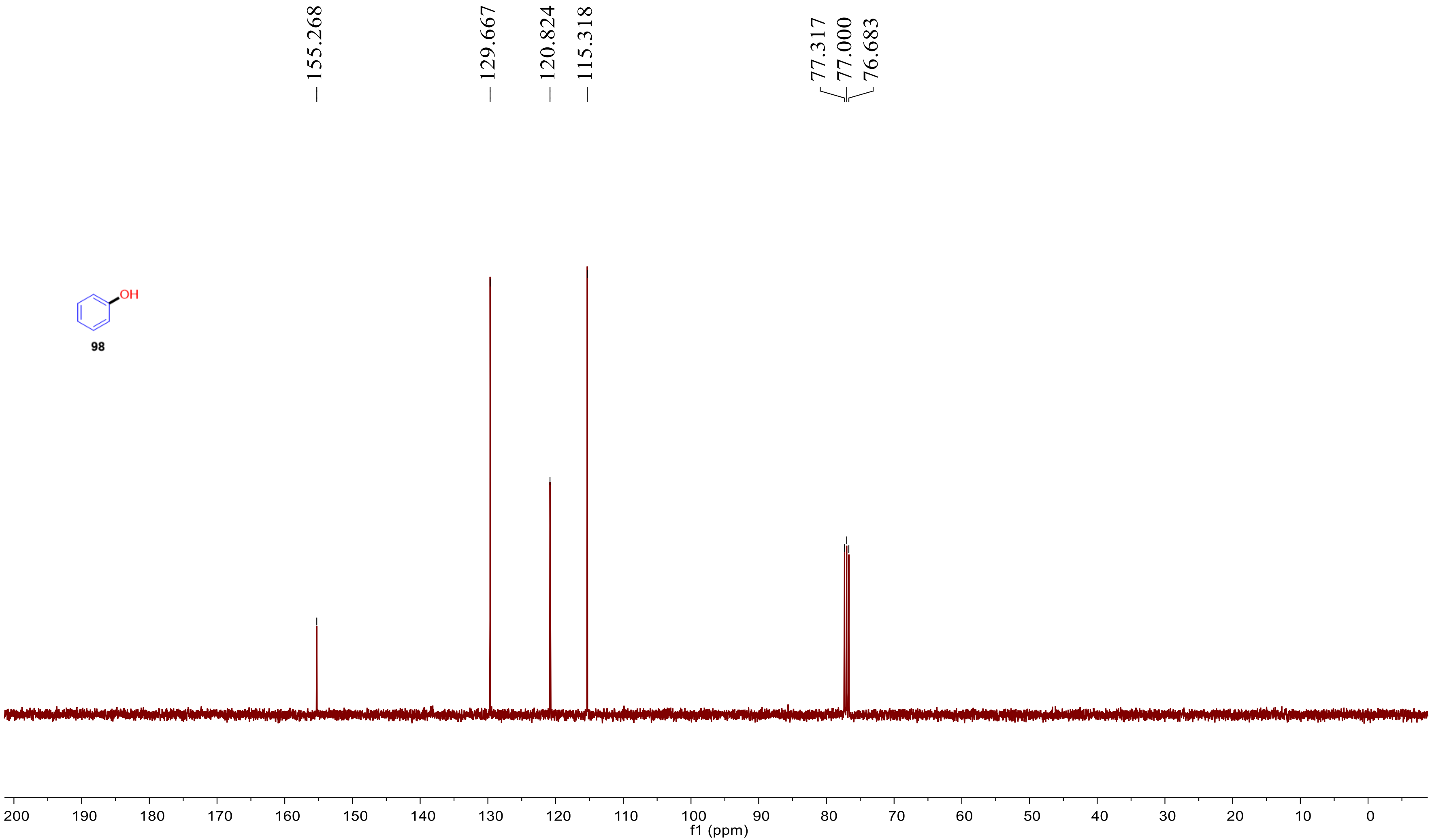

98
155.268
129.667
120.824
115.318
77.317
77.000
76.683
200 190 180 170 160 150 140 130 120 110 100 90 80 70 60 50 40 30 20 10 0
f1 (ppm)

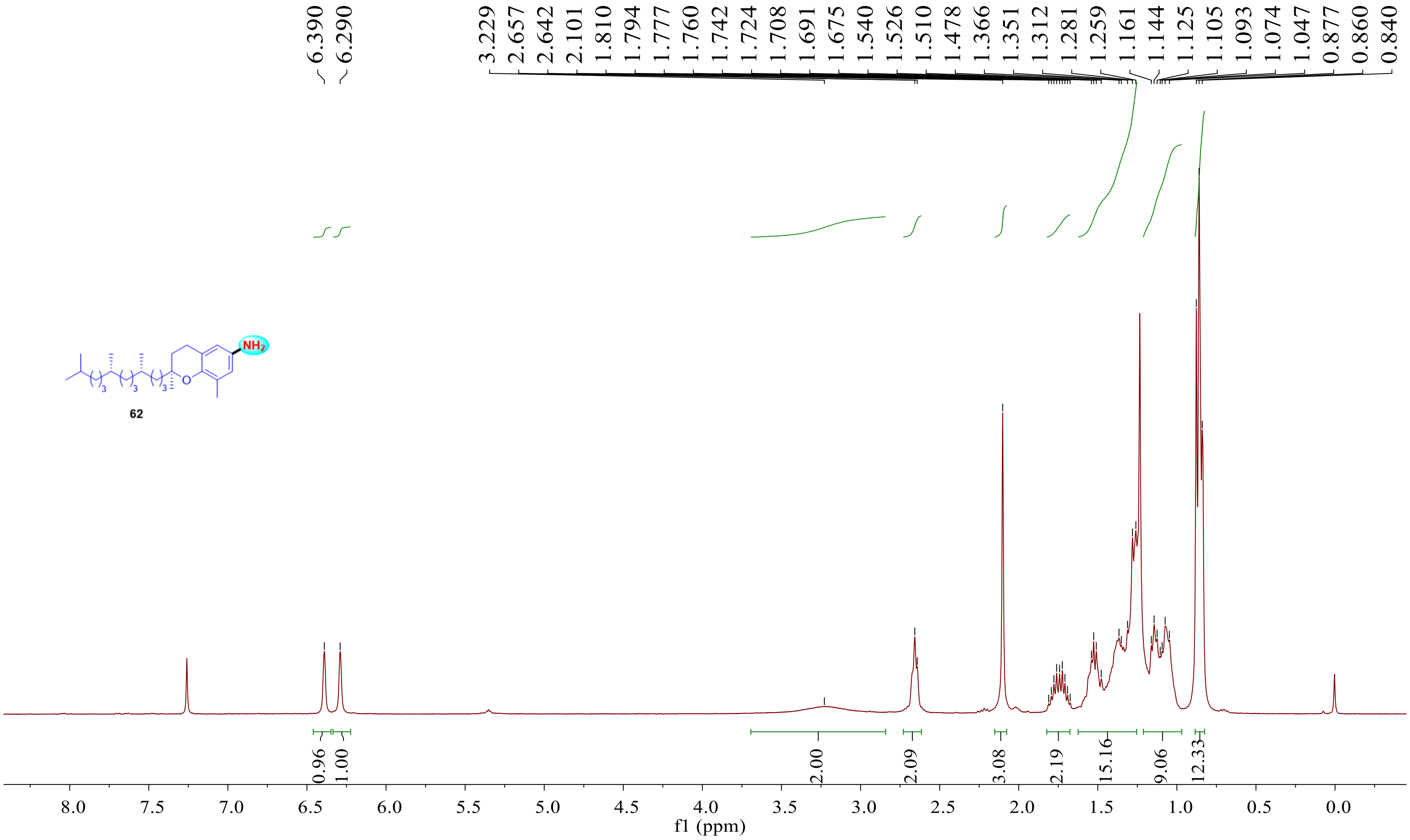
6.390
6.290
3.229
2.657
2.642
2.101
1.810
1.794
1.777
1.760
1.742
1.724
1.708
1.691
1.675
1.540
1.526
1.510
1.478
1.366
1.351
1.312
1.281
1.259
1.161
1.144
1.125
1.105
1.093
1.074
1.047
0.877
0.860
0.840
NH2
O
62
0.96
1.00
2.00
2.09
3.08
2.19
15.16
9.06
12.33
8.0
7.5
7.0
6.5
6.0
5.5
5.0
4.5
4.0
3.5
3.0
2.5
2.0
1.5
1.0
0.5
0.0
f1 (ppm)

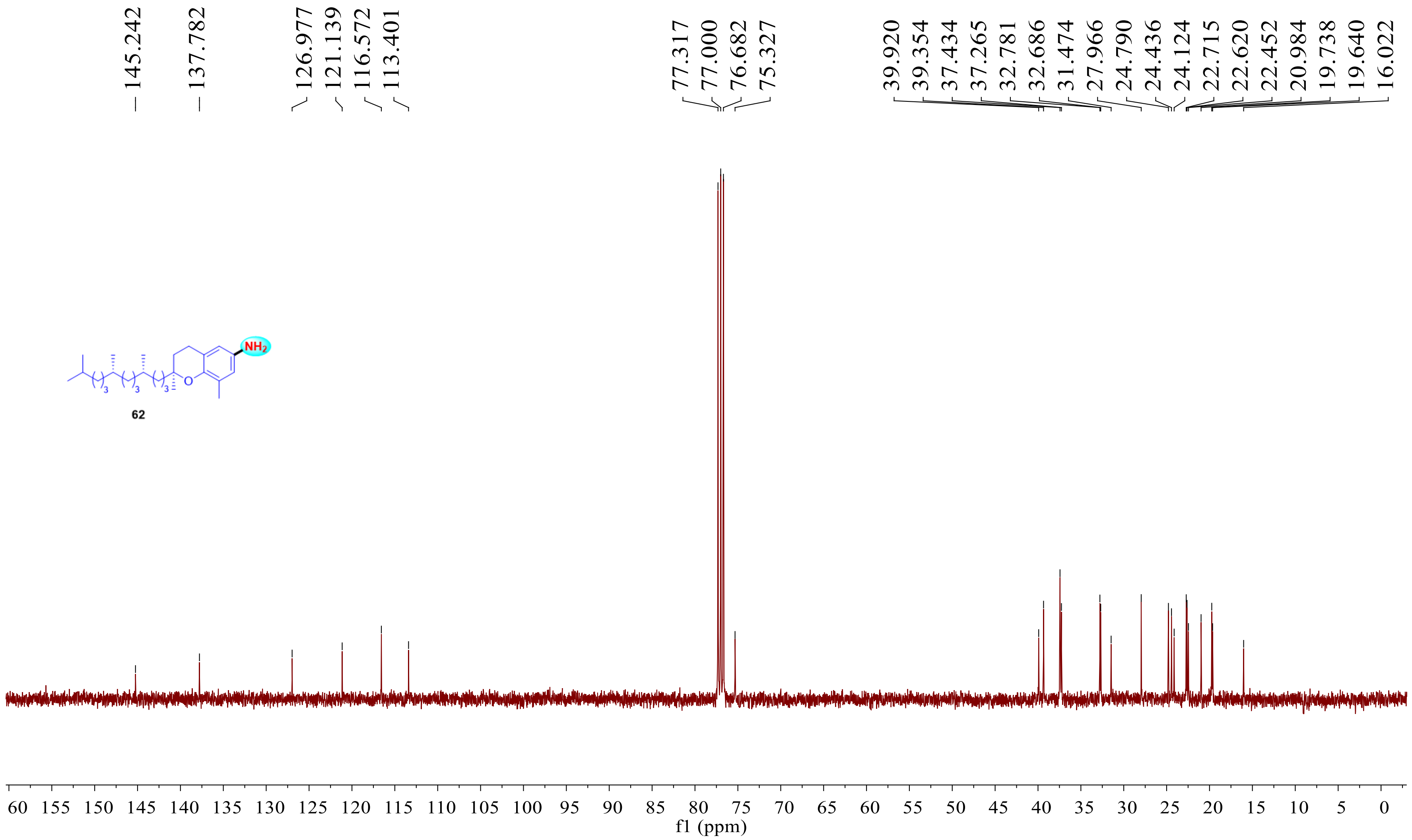
145.242
137.782
126.977
121.139
116.572
113.401
77.317
77.000
76.682
75.327
39.920
39.354
37.434
37.265
32.781
32.686
31.474
27.966
24.790
24.436
24.124
22.715
22.620
22.452
20.984
19.738
19.640
16.022
NH2
O
3
3
3
62
60 155 150 145 140 135 130 125 120 115 110 105 100 95 90 85 80 75 70 65 60 55 50 45 40 35 30 25 20 15 10 5 0
f1 (ppm)

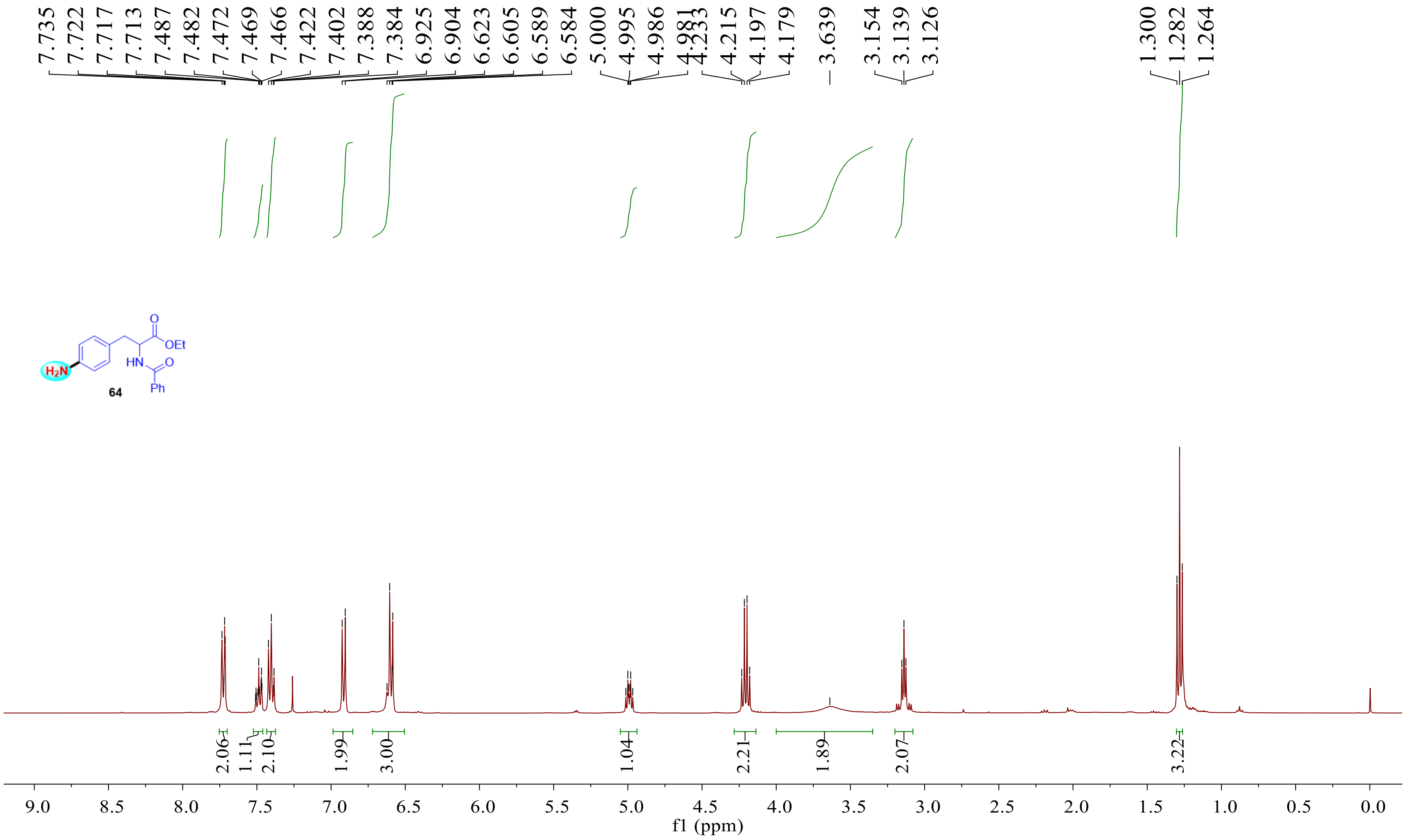
7.735
7.722
7.717
7.713
7.487
7.482
7.472
7.469
7.466
7.422
7.402
7.388
7.384
6.925
6.904
6.623
6.605
6.589
6.584
5.000
4.995
4.986
4.981
4.233
4.215
4.197
4.179
3.639
3.154
3.139
3.126
1.300
1.282
1.264
O
OEt
HN
O
Ph
64
2.06
1.11
2.10
1.99
3.00
1.04
2.21
1.89
2.07
3.22
9.0
8.5
8.0
7.5
7.0
6.5
6.0
5.5
5.0
4.5
4.0
3.5
3.0
2.5
2.0
1.5
1.0
0.5
0.0
f1 (ppm)

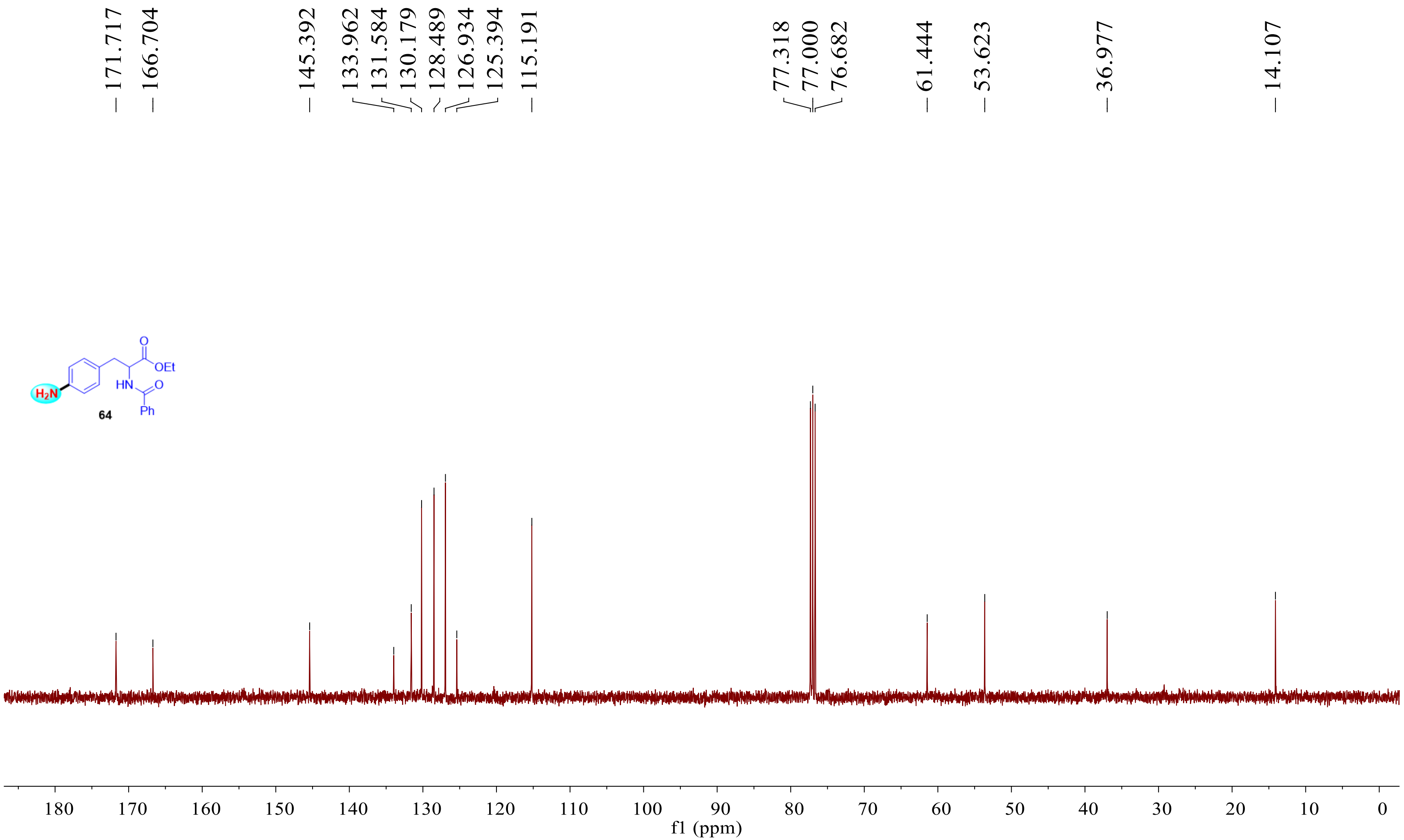

171.717
166.704
145.392
133.962
131.584
130.179
128.489
126.934
125.394
115.191
77.318
77.000
76.682
61.444
53.623
36.977
14.107
O
OEt
HN
O
Ph
H2N
64
180 170 160 150 140 130 120 110 100 90 80 70 60 50 40 30 20 10 0
f1 (ppm)

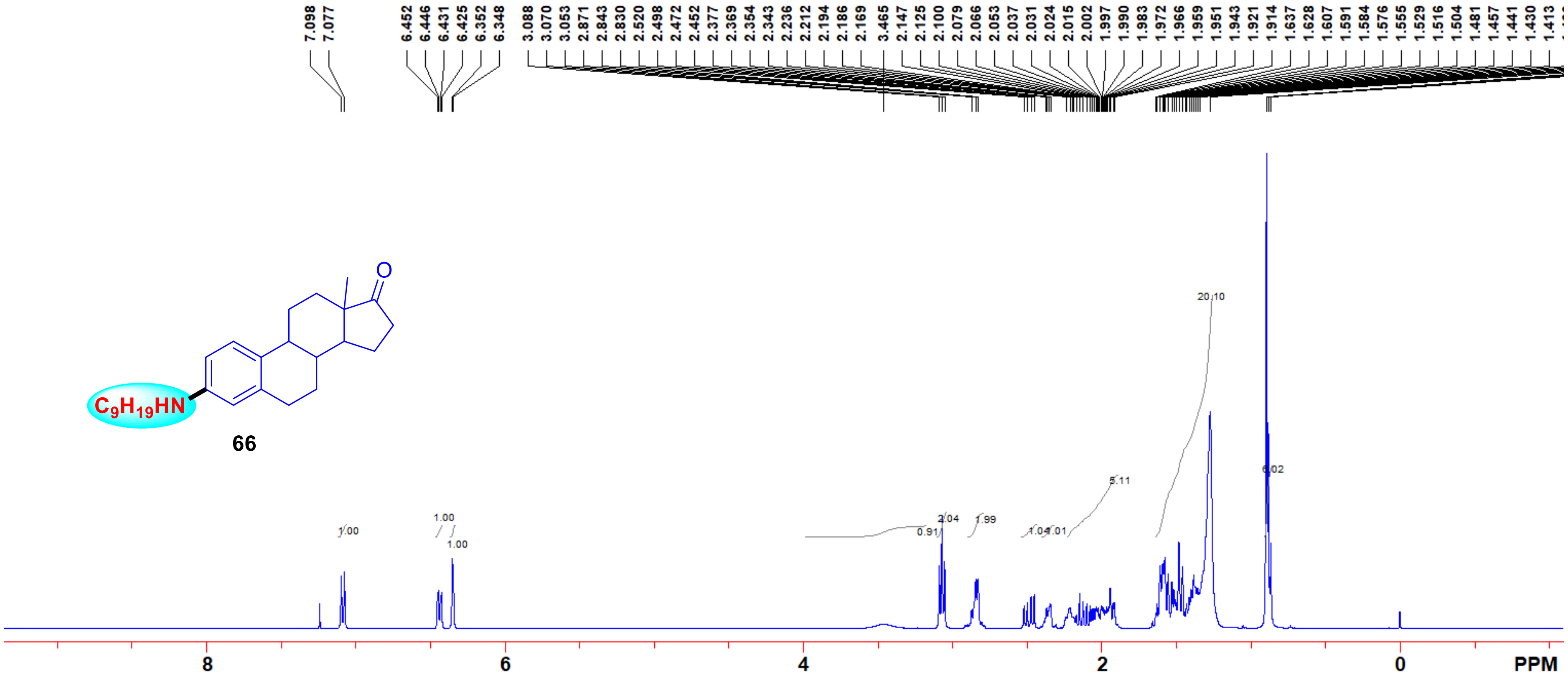

66

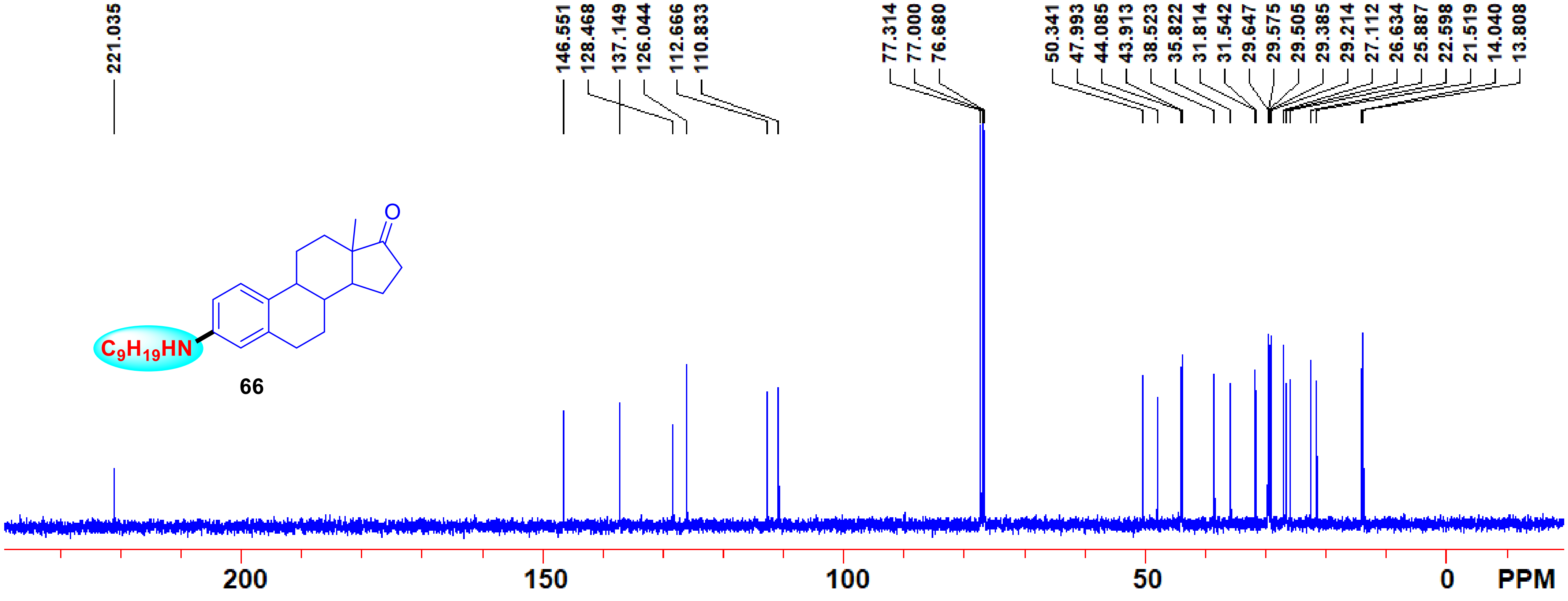

221.035
146.551
128.468
137.149
126.044
112.666
110.833
77.314
77.000
76.680
50.341
47.993
44.085
43.913
38.523
35.822
31.814
31.542
29.647
29.575
29.505
29.385
29.214
27.112
26.634
25.887
22.598
21.519
14.040
13.808
C9H19HN
66
200
150
100
50
0
PPM

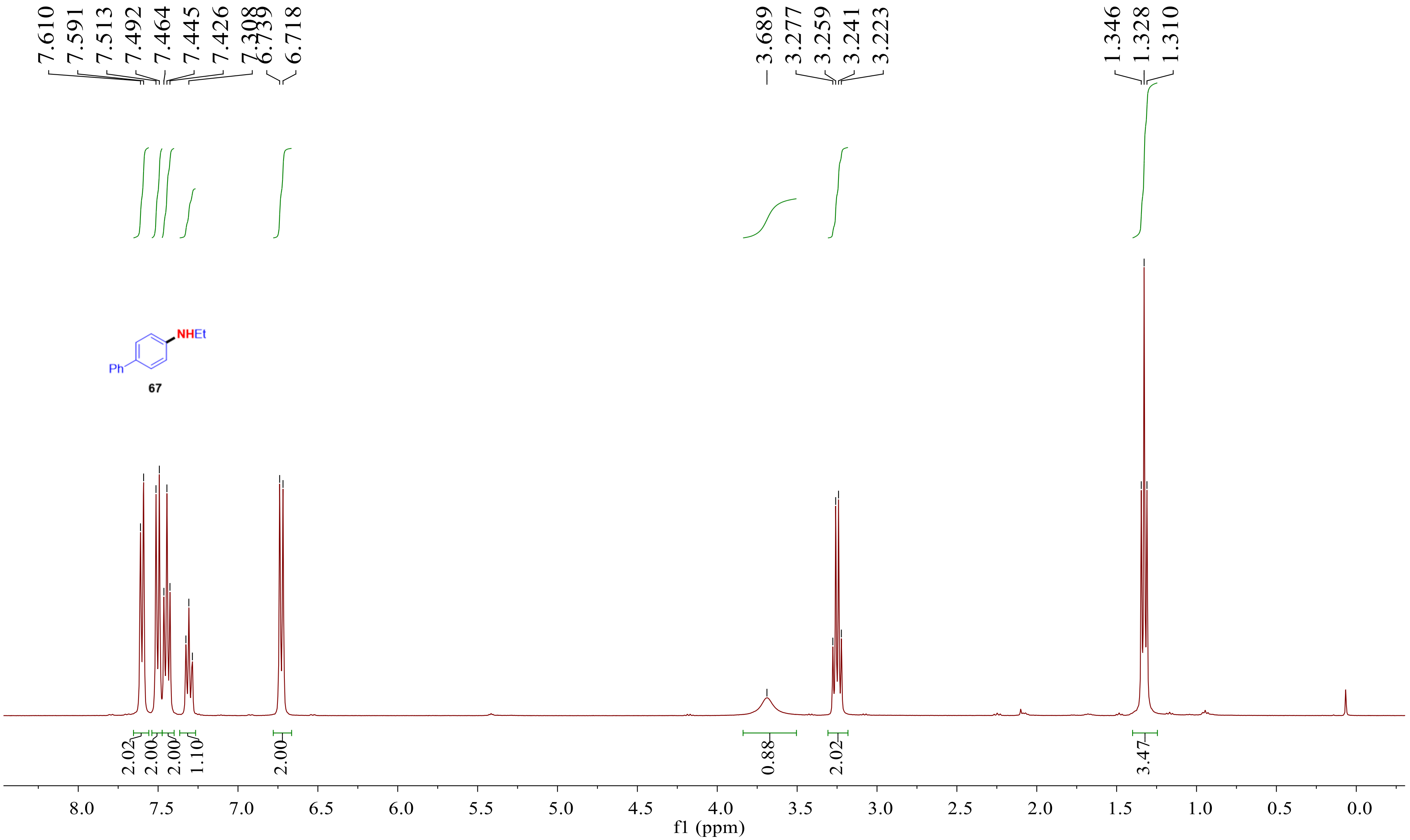
7.610
7.591
7.513
7.492
7.464
7.445
7.426
7.308
6.739
6.718
3.689
3.277
3.259
3.241
3.223
1.346
1.328
1.310
NHEt
Ph
67
2.02
2.00
2.00
1.10
2.00
0.88
2.02
3.47
8.0
7.5
7.0
6.5
6.0
5.5
5.0
4.5
4.0
3.5
3.0
2.5
2.0
1.5
1.0
0.5
0.0
f1 (ppm)

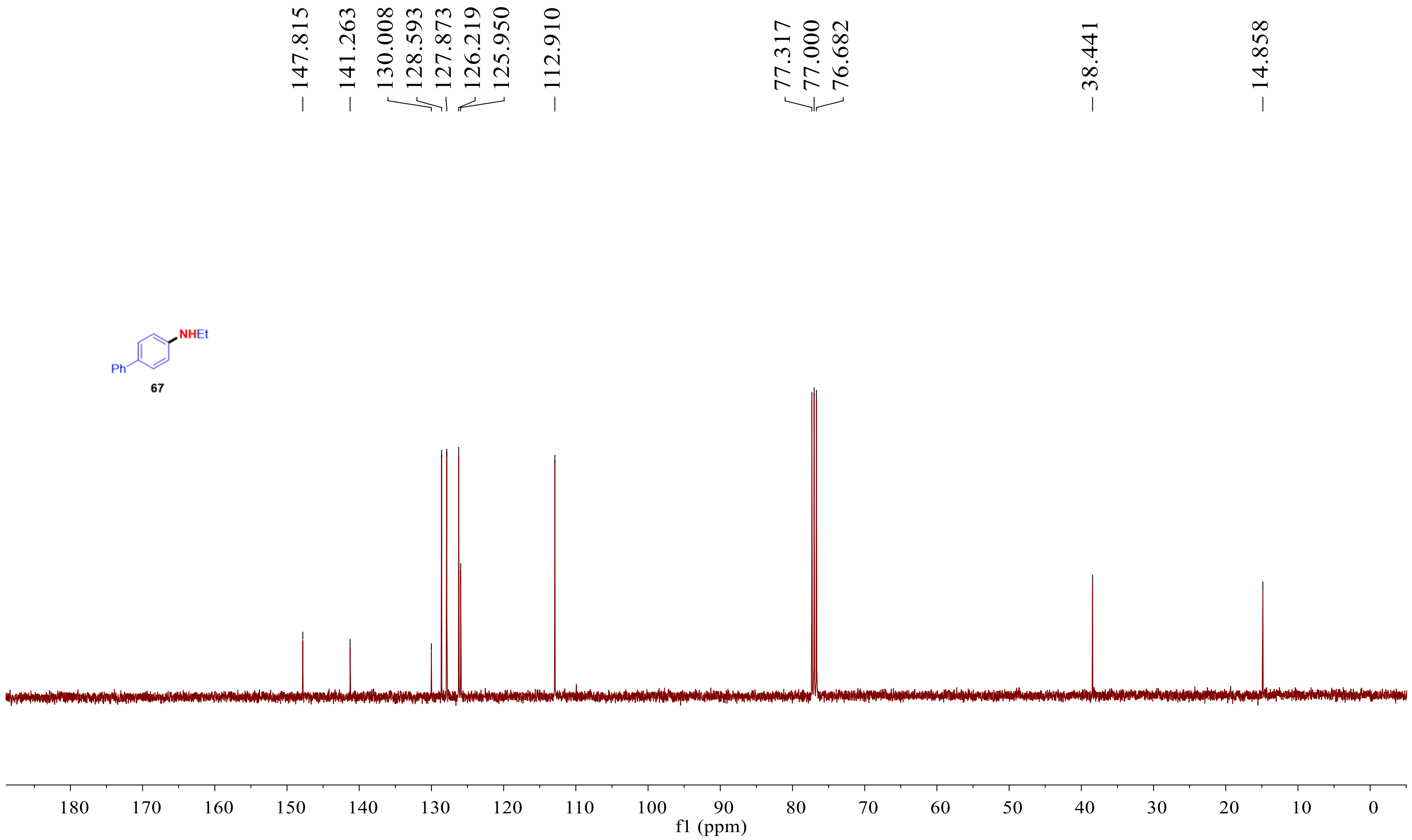
147.815
141.263
130.008
128.593
127.873
126.219
125.950
112.910
77.317
77.000
76.682
38.441
14.858
NHEt
Ph
67
180
170
160
150
140
130
120
110
100
90
80
70
60
50
40
30
20
10
0
f1 (ppm)

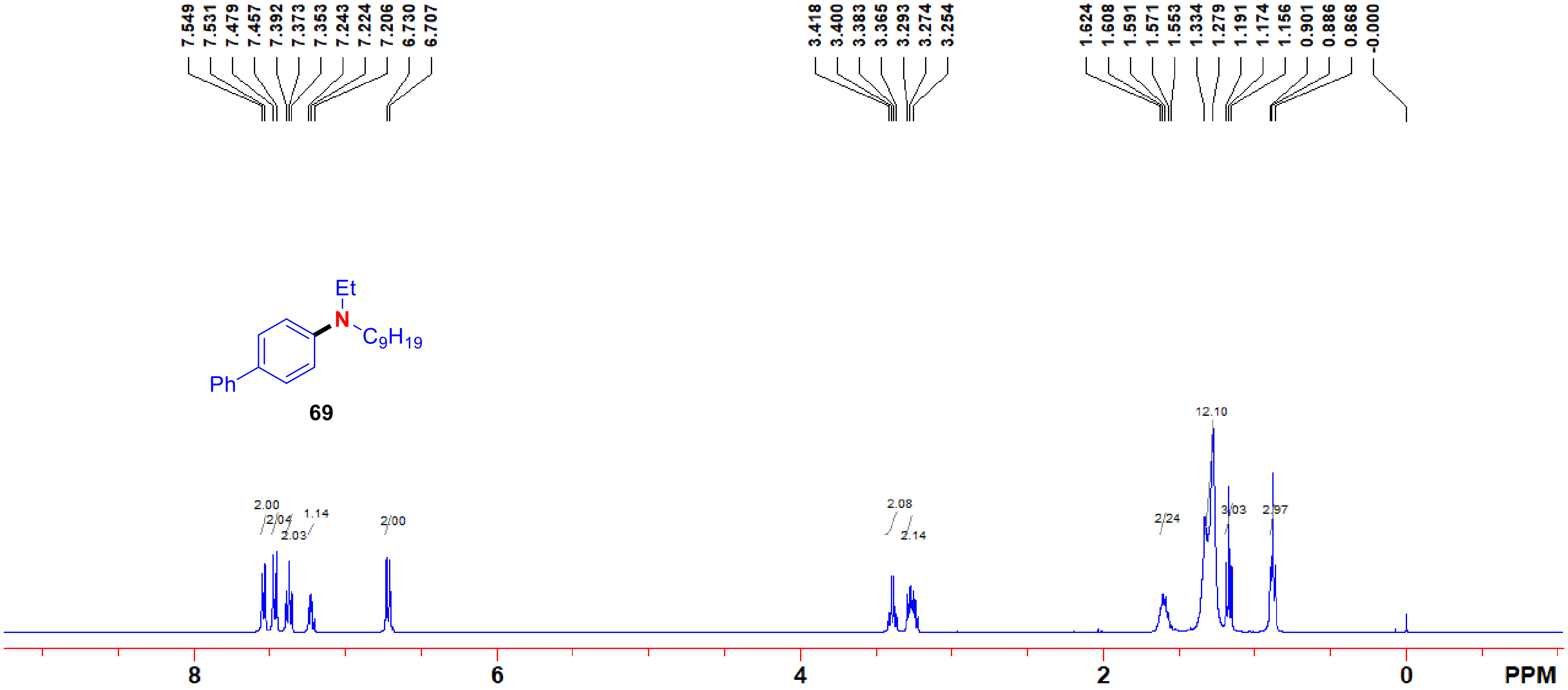

7.549
7.531
7.479
7.457
7.392
7.373
7.353
7.243
7.224
7.206
6.730
6.707
3.418
3.400
3.383
3.365
3.293
3.274
3.254
1.624
1.608
1.591
1.571
1.553
1.334
1.279
1.191
1.174
1.156
0.901
0.886
0.868
-0.000
Et
N
C_9H_{19}
Ph
69
2.00
2.04
2.03
1.14
2.00
2.08
2.14
2.24
12.10
3.03
2.97
8
6
4
2
0
PPM

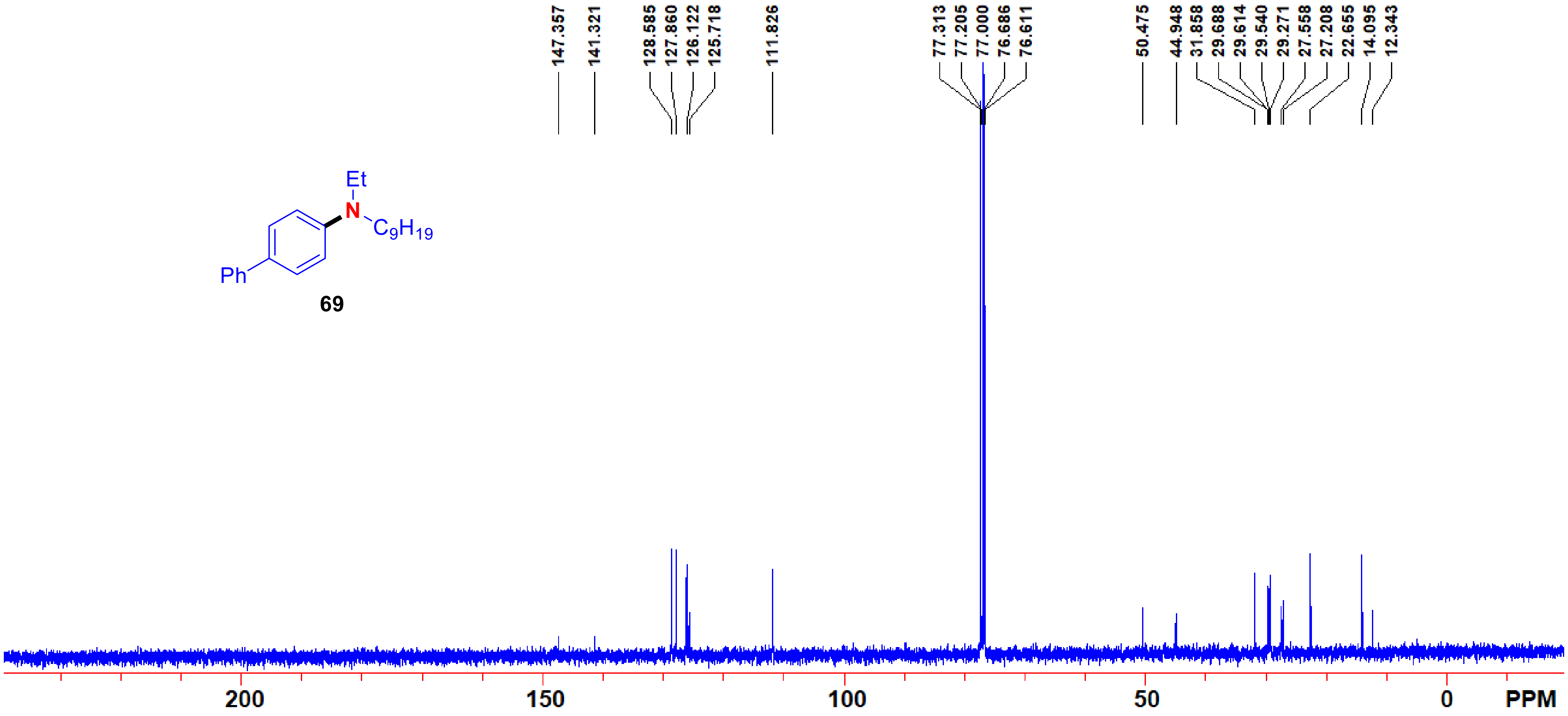
147.357
141.321
128.585
127.860
126.122
125.718
111.826
77.313
77.205
77.000
76.686
76.611
50.475
44.948
31.858
29.688
29.614
29.540
29.271
27.558
27.208
22.655
14.095
12.343
Et
N
C9H19
Ph
69
200
150
100
50
0
PPM
